# DNA methylation signatures of duplicate gene evolution in angiosperms

**DOI:** 10.1093/plphys/kiad220

**Published:** 2023-04-15

**Authors:** Sunil K Kenchanmane Raju, Marshall Ledford, Chad E Niederhuth

**Affiliations:** Department of Plant Biology, Michigan State University, East Lansing, MI 48824, USA; Biology Department, Vassar College, Poughkeepsie, NY 12604, USA; Department of Plant Biology, Michigan State University, East Lansing, MI 48824, USA; AgBioResearch, Michigan State University, East Lansing, MI 48824, USA

## Abstract

Gene duplication is a source of evolutionary novelty. DNA methylation may play a role in the evolution of duplicate genes (paralogs) through its association with gene expression. While this relationship has been examined to varying extents in a few individual species, the generalizability of these results at either a broad phylogenetic scale with species of differing duplication histories or across a population remains unknown. We applied a comparative epigenomic approach to 43 angiosperm species across the phylogeny and a population of 928 Arabidopsis (*Arabidopsis thaliana*) accessions, examining the association of DNA methylation with paralog evolution. Genic DNA methylation was differentially associated with duplication type, the age of duplication, sequence evolution, and gene expression. Whole-genome duplicates were typically enriched for CG-only gene body methylated or unmethylated genes, while single-gene duplications were typically enriched for non-CG methylated or unmethylated genes. Non-CG methylation, in particular, was a characteristic of more recent single-gene duplicates. Core angiosperm gene families were differentiated into those which preferentially retain paralogs and “duplication-resistant” families, which convergently reverted to singletons following duplication. Duplication-resistant families that still have paralogous copies were, uncharacteristically for core angiosperm genes, enriched for non-CG methylation. Non-CG methylated paralogs had higher rates of sequence evolution, higher frequency of presence–absence variation, and more limited expression. This suggests that silencing by non-CG methylation may be important to maintaining dosage following duplication and be a precursor to fractionation. Our results indicate that genic methylation marks differing evolutionary trajectories and fates between paralogous genes and have a role in maintaining dosage following duplication.

## Teaser

Genic DNA methylation marks different evolutionary fates of duplicate genes in angiosperms and may have a role in maintaining dosage following duplication.

## Introduction

Gene and genome duplication increases organismal gene content, generating a repertoire for functional novelty (Bridges 1935; Ohno 1970; Flagel and Wendel 2009). Whole-genome duplication (WGD) increases the entire gene content (Soltis et al. 2015) and is more pervasive in plants than in other eukaryotes (Otto and Whitton 2000; Van de Peer et al. 2017; Cheng et al. 2018a). Small-scale and single-gene duplications (SGDs) are a continuous process with ongoing gene birth and death contributing substantially to gene content (Lynch and Conery 2000; Maere et al. 2005; Panchy et al. 2016). The subsequent retention, divergence, and fractionation (loss) of paralogs are biased depending on the duplication type and gene function (Freeling 2009; De Smet et al. 2013).

Factors determining the evolutionary fate of paralogs are an area of intense study, and DNA methylation is thought to be a contributing factor due to its association with gene expression (Rodin and Riggs 2003; Wang, Wang, et al. 2013). Cytosine methylation at CG dinucleotides is found throughout eukaryotes, while methylation of the non-CG trinucleotide CHG and CHH (H = A, T, or C) contexts is limited to plants (Feng et al. 2010; Zemach et al. 2010). Plant genes have several distinct patterns of DNA methylation within coding regions (henceforth “genic methylation”) that are associated with gene expression (Niederhuth and Schmitz 2017). Genes characterized by CG-only methylation in coding regions are referred to as gene body methylated (gbM) (Tran et al. 2005; Zhang et al. 2006) and are frequently conserved between orthologous genes. gbM genes are typically broadly expressed and evolve more slowly (Takuno and Gaut 2012, 2013; Niederhuth et al. 2016; Takuno et al. 2016). Some genes are methylated similar to transposable elements (TEs), having both CG methylation and non-CG methylation within coding regions. This TE-like methylation (teM) is rarely conserved between orthologs and results in transcriptional silencing (Seymour et al. 2014; Niederhuth et al. 2016; El Baidouri et al. 2018). Most genes, however, are unmethylated (unM) and exhibit variable expression across tissues and conditions (Takuno and Gaut 2012; Niederhuth et al. 2016).

DNA methylation could serve to buffer the genome against changes in gene dosage by modulating gene expression and facilitating functional divergence. Tissue-specific silencing of paralogs might lead to “epigenetic complementation,” through subfunctionalization of expression and paralog retention (Adams et al. 2003; Rodin and Riggs 2003). Alternatively, silencing may contribute to pseudogenization and subsequent fractionation (Hua et al. 2013; El Baidouri et al. 2018). Studies in plants (Hua et al. 2013; Wang, Wang, et al. 2013; Wang, Marowsky, and Fan 2014; Kim et al. 2015; Wang et al. 2015, 2017; El Baidouri et al. 2018; Wang et al. 2018; Xu et al. 2018) and animals (Chang and Liao 2012; Keller and Yi 2014) have found that increasing DNA methylation differences between paralogs are associated with divergence in sequence evolution and expression. In soybean (*Glycine max*), gene transposition to heterochromatic regions resulted in silencing by non-CG methylation, increased sequence divergence, and likely pseudogenization (El Baidouri et al. 2018). In the highly duplicated F-box family of *Arabidopsis thaliana*, silencing by DNA methylation and trimethylation of lysine 27 on histone H3 protein (H3K27me3) was associated with increased sequence divergence and was proposed to have a role in maintaining dosage balance (Hua et al. 2013).

Past studies of DNA methylation and gene duplication have been limited to individual species, focused primarily on WGDs, and often ignore the contextual differences of genic methylation. Lineage-specific variation in DNA methylation (Niederhuth et al. 2016), histories of gene duplication (Qiao et al. 2019), and differences in analysis have precluded an overarching understanding of the relationship between DNA methylation and paralog evolution. To address these issues, we analyze genic methylation contexts across 43 angiosperm species and a population of 928 *A. thaliana* ecotypes. We find overarching trends and relationships between genic methylation, the type and age of duplication, gene family, and paralog evolution. This work provides a broad phylogenetic and population-scale understanding of the role of DNA methylation in plant duplicate gene evolution and suggests that DNA methylation may have a role in maintaining dosage prior to fractionation.

## Results

### Genic methylation across duplication types

**Figure 1.**
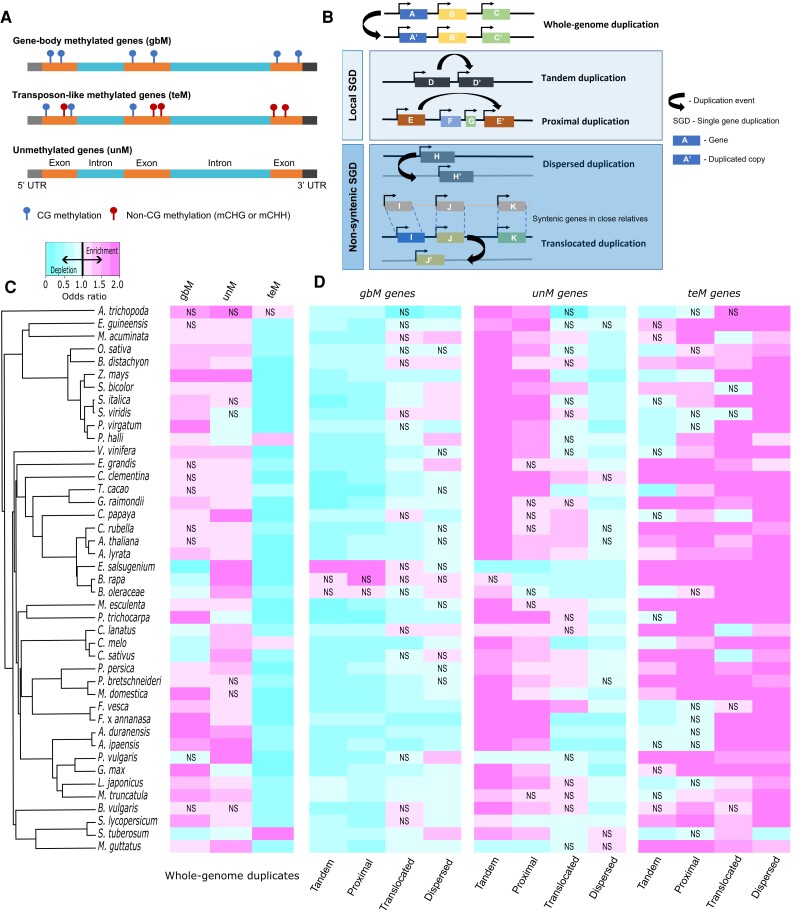
Patterns of genic methylation across different types of gene duplicates. **A)** Schematic representation of genic methylation classification. Coding regions of gbM genes are methylated in the CG context only, teM genes in both CG and non-CG (CHG and CHH) contexts, and unM genes are unmethylated. **B)** Classification of duplicate genes into WGDs and different types of SGDs (tandem, proximal, dispersed, and translocated). **C**, **D)** Enrichment or depletion of each genic methylation class (gbM, unM, and teM) for **C)** WGDs and **D)** different types of SGDs. A Fisher's exact test odds ratio of <1 represents depletion and >1 indicates enrichment. Unless indicated, all associations are statistically significant at an FDR-corrected *P* < 0.05. “NS” indicates no statistical significance.

We analyzed genic methylation and gene duplication for 43 angiosperm species (Supplemental Table S1). Genes were classified as gbM, unM, or teM based on DNA methylation in coding regions (Fig. 1A and Supplemental Table S2). Gene duplicates were identified and classified (Supplemental Table S3) as either WGDs or 1 of 4 types of SGDs: tandem, proximal, translocated, or dispersed (Fig. 1B). Tandem duplicates occur through unequal crossing-over, resulting in adjacent paralogous copies (Zhang 2003). Proximal duplicates are separated by several intervening genes and arose either through local transposition or interruption of ancient tandem duplicates (Zhao et al. 1998; Freeling et al. 2008). Translocated duplicates (also known as “transposed”) are distally located pairs in which 1 of the genes is syntenic and the other is nonsyntenic (Wang, Li, and Paterson 2013; Qiao et al. 2019) and can arise either by retrotransposition or DNA-based duplication (Cusack and Wolfe 2006). Finally, dispersed duplicates are pairs that fit none of the above criteria and can arise through multiple mechanisms (Ganko et al. 2007; Freeling 2009; Qiao et al. 2019).

We hypothesized that different duplication types would differ in genic methylation. Each duplication type was tested for enrichment or depletion of gbM, unM, and teM in each species (Fig. 1, C and D, and Supplemental Table S4). Across angiosperms, WGDs were more frequently enriched for gbM (27/43 enriched and 7/43 depleted) and unM (32/43 enriched and 5/43 depleted) and depleted for teM (3/43 enriched and 39/43 depleted). Notable exceptions include 3 Brassicaceae species [cabbage (*Brassica oleracea*), field mustard (*Brassica rapa*), and saltwater cress (*Eutrema salsugineum*)], 3 Cucurbitaceae species [watermelon (*Citrullus lanatus*), muskmelon (*Cucumis melo*), and cucumber (*Cucumis sativus*)], and potato (*Solanum tuberosum*). The 3 Brassicaceae species are known to be depleted of gbM genome wide (Bewick et al. 2016). No known depletion of gbM is documented in the Cucurbitaceae. While *C. melo* WGDs are depleted of gbM and enriched in teM, *S. tuberosum* is the only species showing depletion of both gbM and unM and enrichment of teM in WGDs. “Local” tandem and proximal SGDs are more similar in enrichment/depletion to each other compared with “distal” translocated and dispersed SGDs. Local SGDs are depleted of gbM (tandem and proximal: 40/43 depleted and 1/43 enriched) in all species except for the 3 gbM-deficient Brassicaceae species and are enriched for unM in the majority of species (tandem: 39/43 enriched and 3/43 depleted; proximal: 31/43 enriched and 5/43 depleted). Tandem and proximal duplicates differed more in teM (tandem: 19/43 enriched and 14/43 depleted; proximal: 31/43 enriched and 1/43 depleted), with proximal duplicates showing more species enriched for teM than tandem. Like local SGDs, distal SGDs were more frequently depleted for gbM (translocated: 0/43 enriched and 27/43 depleted; dispersed: 11/43 enriched and 21/43 depleted), although dispersed SGDs were most frequently enriched for gbM. Translocated and dispersed duplicates differ for unM; translocated duplicates have similar numbers of enriched and depleted species (13/43 enriched and 14/43 depleted), while dispersed duplicates are depleted in most species (0/43 enriched and 36/43 depleted). Distal SGDs are more frequently enriched for teM than local SGDs (translocated: 34/43 enriched and 4/43 depleted; dispersed: 42/43 enriched and 1/43 depleted). Increasing teM frequency from tandem to proximal to distal SGDs suggests that teM becomes more common as genes duplicate to increasingly different sequences or chromatin environments.

### Effect of gene family on genic methylation and duplication

Gene families differ in their duplicability and retention. Past work has revealed “duplication-resistant” gene families that repeatedly return to single-copy status (Paterson et al. 2006; De Smet et al. 2013; Li et al. 2016), while other gene families retain duplicates over long evolutionary timescales (Conant et al. 2014). To see how gene family composition affects the relationship between genic methylation and duplication, we identified orthogroups for the 43 species with methylome data and additional 15 species included as outgroups (Supplemental Table S1 and Fig. S1). Orthogroups showed a bimodal distribution (Supplemental Fig. S2), with the majority of orthogroups present in either a few species or conserved across most species (Li et al. 2016). Orthogroups represented in ≥51 species were classified as “core angiosperm” genes and further divided as “core:single-copy” (duplication-resistant) if represented by a single-copy in ≥70% species (Li et al. 2016) and the remainder as “core:multicopy.” The remaining orthogroups were classified based on increasing lineage specificity: “cross family” if present in multiple plant families, “family specific” if restricted to a single family, or “species/lineage specific” if limited to a single species. Genic methylation shows more consistent enrichment and depletion across species for orthogroups than for duplication type (Supplemental Figs. S3 and S4 and Table S5). gbM genes are enriched in core angiosperm orthogroups (multicopy and single-copy) and depleted in noncore orthogroups. The only exceptions were the gbM-depleted species field mustard and saltwater cress. teM genes have the opposite pattern and are depleted in core angiosperm orthogroups and enriched in noncore orthogroups, suggesting a more recent evolutionary origin for gene families enriched for teM. unM genes are variably represented across orthogroups but are more frequently enriched in cross-family (32/43 enriched and 4/43 depleted) and depleted in core:single-copy (2/43 enriched and 41/43 depleted) orthogroups.

We next tested enrichment or depletion of duplicate types in each orthogroup category (Supplemental Fig. S5 and Table S6). SGDs are more frequently enriched in cross-family and family-specific orthogroups and also in core:multicopy genes, except for proximal duplicates, which is the only duplication type depleted in core:multicopy genes in most species (4/43 enriched and 29/43 depleted). Every duplication type was depleted in core:single-copy orthogroups with the exception of enrichment of dispersed duplicates in soybean and WGDs in strawberry (*Fragaria* × *ananassa*), soybean, apple (*Malus* × *domestica*), and switchgrass (*Panicum virgatum*). The greatest enrichment was in the extant polyploids *F.* × *ananassa* (octoploid) and *P. virgatum* (tetraploid), suggesting insufficient time since WGD to revert to singletons. WGDs were enriched in core:multicopy orthogroups and depleted in noncore orthogroups with the exception of *C. melo* WGDs, which showed enrichment in cross-family orthogroups, and *S. tuberosum* WGDs, which is enriched in cross-family, family-specific, and species/lineage-specific orthogroups. As noted in the previous section, WGDs of these 2 species are depleted for gbM and enriched for teM. The enrichment of WGDs in noncore orthogroups for these species may explain why WGDs differ in their enrichment/depletion of genic methylation. *S. tuberosum* is also the only extant autopolyploid in our data set and is of relatively recent origin (Potato Genome Sequencing Consortium et al. 2011; Wang et al. 2018), which could result in overrepresentation of more lineage-specific genes that are more likely to be teM. Collectively, these results indicate that gene family composition is a driving factor in the relationship between gene duplication and genic methylation.

### Methylation divergence between paralogs

**Figure 2.**
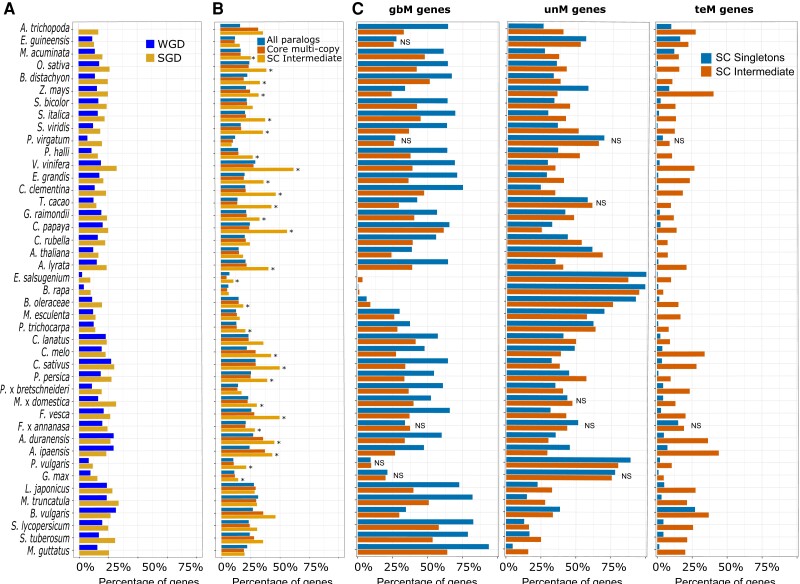
Genic methylation patterns in duplicate paralogs and single-copy orthologs. **A)** Proportion of duplicate pairs with divergence in methylation between the two paralogs in WGDs and SGDs. **B)** Proportion of duplicate pairs with divergent genic methylation profiles in core:multicopy, core:single-copy (SC) intermediates, and all duplicate pairs. A two-proportion *Z*-test was used to compare core:multi-copy and SC intermediates. Statistically significant differences are indicated by “*”. **C**) Proportions of gbM, unM, and teM in SC-singletons and SC-intermediates. A two-proportion *Z*-test was used to compare SC-intermediates and SC-singletons. Unless indicated, all comparisons are statistically significant at an FDR-corrected *P* < 0.05. “NS” indicates no statistical significance.

Changes in genic methylation might facilitate functional divergence between paralogs and mark different evolutionary trajectories, so we determined the extent of genic methylation differences between paralogs (Fig. 2A and Supplemental Table S7). WGD pairs have the highest similarity in genic methylation (same: ∼69% to 97%, median: 84%; different: ∼3% to 31%, median: 16%), followed by tandem (same: ∼69% to 93%, median: 82%; different: ∼7% to 31%, median: 18%), proximal (same: ∼66% to 90%, median: 77%; different: ∼10% to 34%, median: 23%), and dispersed (same: ∼65% to 92%, median: 76%; different: ∼8% to 35%, median: 24%). Translocated duplicates had the broadest range and the greatest proportion of pairs differing in genic methylation (same: ∼57% to 90%, median: 74%; different: ∼10% to 42%, median: 25%) (Supplemental Fig. S6 and Table S7). Amborella (*Amborella trichopoda*) is an outlier in this analysis due to the small number of genes classified as WGD or translocated. The direction of genic methylation changes cannot typically be discerned. However, for translocated duplicates, 1 paralog is syntenic and considered parental locus and the translocated gene the daughter locus (Wang, Li, and Paterson 2013). Translocated copies had higher teM proportions in 34/43 species and lower gbM proportions in 24/43 species (Supplemental Table S8). Assuming that parental locus methylation is the original state, we can determine the directionality of methylation changes in the translocated copy. Switching to unM was the most common in 22/43 species, switching to teM in 19/43 species, and switching to gbM in only 2/43 species (Supplemental Table S9). This shows that while most pairs retain the same genic methylation status, changes are not infrequent, becoming more common as duplicates move to increasingly distal locations and that changes are predominantly to unM and teM, rarely to gbM.

Core:single-copy orthogroups are also duplicated during WGD but are quickly eliminated by fractionation and are thus depleted in WGDs in nearly every species (Supplemental Fig. S5 and Table S6). These “duplication-resistant” pairs are dosage sensitive, and we hypothesized that DNA methylation has a role in maintaining dosage of these genes following duplication. Core:single-copy genes are predominantly gbM or unM, with few teM genes (Supplemental Fig. S3). gbM and unM core:single-copy genes differ in functional enrichment (Supplemental Table S10). GbM genes are enriched for cellular response to stress and metabolic processes, while unM are enriched in processes like photosynthesis, cell redox homeostasis, and cell cycle checkpoint signaling. Although most core:single-copy genes are singletons (SC-singletons) within a species, duplicates persist in varying numbers across angiosperms. We termed these “single-copy intermediates” (SC-intermediates). SC-intermediate genes differed in their genic methylation at a much higher frequency than both core:multicopy pairs and duplicate pairs as a whole (Fig. 2B). SC-singletons are enriched for gbM genes compared with SC-intermediates (Fig. 2C), while SC-intermediates have significantly more teM genes than SC-singletons. These results support our hypothesis and indicate a potential role for DNA methylation in maintaining dosage of single-copy core angiosperm genes following duplication and prior to fractionation.

### Genic methylation marks paralog age and sequence evolution

**Figure 3.**
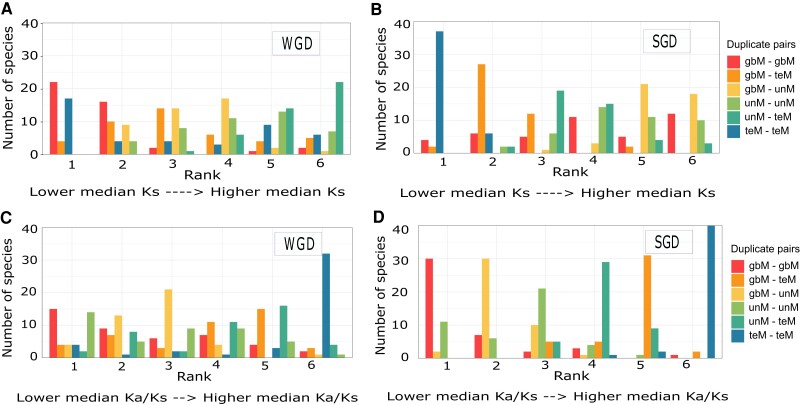
Relationship between genic methylation, age of duplication, and sequence evolution of duplicate paralogs. The number of species in each of the duplicate pair genic methylation classifications (gbM–gbM, gbM–teM, teM–teM, unM–unM, gbM–unM, and unM–teM) ranked based on median Ks values (synonymous substitutions) for WGDs **A)** and SGDs **B)**. The number of species in each of the duplicate pair genic methylation classifications (gbM–gbM, gbM–teM, teM–teM, unM–unM, gbM–unM, and unM–teM) ranked based on median Ka/Ks values (ratio of Ka, nonsynonymous substitutions to Ks, synonymous substitutions) for WGDs **C)** and SGDs **D)**.

We next examined the relationship between genic methylation and paralog evolution. Synonymous substitutions (Ks) are assumed to accumulate neutrally with time, and Ks distributions have been used to date duplication events (Lynch and Conery 2000; Maere et al. 2005). The number and timing of WGD events differ across angiosperms, so we did not expect to find any shared trends for WGD Ks. Contrary to this expectation, WGD gbM-containing pairs tended to have lower Ks values and unM-containing pairs higher Ks values (Fig. 3A and Supplemental Table S11). No clear trend was observed for teM-containing WGD pairs. This may be due to the depletion and lower numbers of teM in WGDs. In contrast to WGD, SGD is a continuous process with constant gene birth and death (Lynch 2002). SGD teM-containing pairs typically had lower Ks values than those with only gbM or unM paralogs (Figs. 3B and S7). This is most evident for teM–teM pairs, but gbM–teM and unM–teM also have lower Ks values. This suggests that teM paralogs tend to be evolutionarily younger. We confirmed this using a method independent of Ks for translocated genes. As the syntenic gene is assumed to be parental in translocated genes, the daughter gene can be parsed into different periods (epochs) at each node of the species tree (Supplemental Table S12) by sequential exclusion to the closest outgroup (Wang, Li, and Paterson 2013). More recent translocated duplicates were enriched in teM paralogs, while more ancient translocated duplicates were enriched for gbM and unM paralogs (Supplemental Fig. S8 and Table S13), supporting our observations from the Ks analysis. These results also fit with the observation that evolutionarily younger lineage-specific orthogroups are enriched for teM genes. We also compared Ks distributions for SC-intermediates and core:multicopy genes but observed no difference suggesting similar evolutionary ages (Supplemental Fig. S9).

The ratio of nonsynonymous (Ka) to synonymous (Ks) substitutions (Ka/Ks) is indicative of sequence evolution (Miyata and Yasunaga 1980; Yang and Bielawski 2000). A Ka/Ks < 1 is indicative of purifying selection, Ka/Ks = 0 indicates neutral selection, and Ka/Ks > 1 is indicative of diversifying selection. We calculated Ka/Ks ratios for each duplicate pair and examined their distributions (Supplemental Fig. S10 and Table S14). The majority of pairs have a Ka/Ks < 1, regardless of duplication type or genic methylation, indicating purifying selection. However, there are differences in the distribution based on genic methylation. For both WGD and SGD genes (Fig. 3, C and D), gbM-containing pairs have lower Ka/Ks, teM-containing pairs have higher Ka/Ks, while unM-containing pairs are intermediate in distribution. This suggests that teM paralogs are under relaxed selective constraints compared with gbM and unM paralogs. A number of pairs had a Ka/Ks > 1 indicating diversifying selection. These were enriched for SGD and depleted for WGD in almost every species except *S. tuberosum* (Supplemental Table S15) and enriched in teM-containing pairs, in particular teM–teM pairs (Supplemental Table S16). We hypothesized that SC-intermediates would be under relaxed selective constraints as these are enriched for teM. Indeed, SC-intermediates have higher Ka/Ks values compared with core:multicopy pairs (Supplemental Fig. S11). Increased nonsynonymous substitutions in SC-intermediates could lead to their pseudogenization and facilitate fractionation to singleton status.

Ongoing gene duplication and differential fractionation within a species can create presence–absence variation (PAV). We used published lists of PAVs in *B. oleracea*, maize (*Zea mays*), tomato (*Solanum lycopersicum*), and *S. tuberosum* (Hirsch et al. 2014; Golicz et al. 2016; Hardigan et al. 2016; Gao et al. 2019) to examine the relationship between PAVs, genic methylation, and gene duplication (Supplemental Fig. S12 and Table S17). PAVs are enriched for teM genes in all 4 species and depleted for gbM in 3 species, except gbM-deficient *B. oleracea*. PAVs are depleted for unM genes in 3 species and enriched for unM in *S. lycopersicum*. Results are identical whether tested for all genes or duplicated genes only. Association between PAVs and teM could result from targeting of lineage-specific SGDs or incomplete fractionation of teM duplicates in the population. An example of the latter would be fractionation of core:single-copy orthogroups following WGD. In all 4 species, SC-intermediates had higher frequencies of PAV compared with core:multicopy orthogroups and a higher frequency compared with all genes in maize (Supplemental Fig. S13), supporting the hypothesis that teM silencing is an intermediate to fractionation for single-copy core angiosperm genes.

### Genic methylation and paralog expression

**Figure 4.**
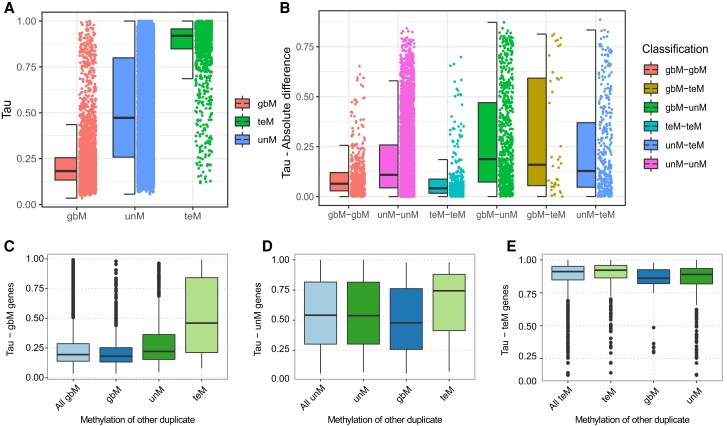
Gene expression specificity of *A. thaliana* duplicate genes. Tissue specificity index (*τ*), ranges from 0 (broadly expressed) to 1 (narrowly expressed). **A)** Tissue specificity of genes based on genic methylation classification (gbM, unM, and teM). Each dot represents *τ* value of a gene. Center line in the half boxplot represents the median *τ* while the box limits represent 25% and 75% percentile of the interquartile range; whiskers represent 1.5 times above or below the interquartile range. **B)** Absolute difference in tissue specificity index (*τ*) between pairs of duplicate genes with similar or divergent methylation. Tissue specificity index (*τ*) of **C)** gbM, **D)** unM, and **E)** teM genes when the other duplicate pair has the same or a different genic methylation status. For example, for **C)** gbM genes *τ* was plotted for all gbM genes and the gbM paralog in gbM–gbM, gbM–teM, and gbM–unM pairs. Similarly, *τ* of only the unM paralog is plotted for **D)** unM genes and *τ* of the teM paralog for **E)** teM genes. Dots represent outliers 1.5 times above or below the interquartile range.

Divergent expression between paralogs is proposed to be the first step in functional diversification, enabling paralogs to subfunctionalize in expression and increasing the odds of retention (Ohno 1970; Ferris and Whitt 1979; Li et al. 2005). We used gene expression atlases in *A. thaliana*, *G. max*, common bean (*Phaseolus vulgaris*), and sorghum (*Sorghum bicolor*) (O’Rourke et al. 2014; Klepikova et al. 2016; McCormick et al. 2018; Wang et al. 2019) to explore the relationship between genic methylation and paralog expression. For each gene, we calculated the expression specificity (*τ*), a measure of the number of conditions in which a gene is expressed (Yanai et al. 2005). The value of *τ* ranges from “0” (broad expression) to “1” (narrow expression). Typically, gbM genes have the broadest expression, teM the narrowest, while unM genes have a wide range of expression specificity (Figs. 4A and S14). Core angiosperm genes are more broadly expressed, expression specificity becoming narrower with increasing lineage specificity (Supplemental Fig. S15). This trend persists when broken down by genic methylation. gbM, unM, and teM genes become more narrowly expressed with increasing lineage specificity. By duplication type, WGDs have broader expression and SGDs narrower expression (Supplemental Fig. S16). Distal SGDs have broader expressions than local SGDs, even though distal SGDs can place duplicates in new chromatin contexts and are more frequently enriched for teM. Similar to what was observed for orthogroups, differences between duplication types persist even when comparing genes of like genic methylation (Supplemental Fig. S16). WGD teM genes had overall broader expression than distal teM SGD teM genes, which had broader expression than local SGD teM genes. This same trend was observed for gbM and unM. Both orthogroup and duplication type therefore exert an effect beyond the genic methylation, and global differences between both orthogroups and duplication types are not fully explained by differential enrichment of genic methylation.

We next examined expression divergence between duplicate pairs, first calculating the expression correlation of each pair (Supplemental Fig. S17). Pairs with the same genic methylation had higher correlation than pairs differing in genic methylation, gbM–gbM pairs having the highest overall correlation. Fitting this, duplicate pairs differing in genic methylation have a greater overall absolute difference in expression specificity than pairs with the same genic methylation (Figs. 4B and S18 and Supplemental Table S18). Surprisingly, the expression specificity differed for genes of the same genic methylation based on the methylation of its duplicate pair (Figs. 4, C to E, and S19). gbM genes that are part of gbM–gbM pairs tend to have broader expression specificity compared with the gbM genes in gbM–unM and gbM–teM pairs. For unM genes, those in unM–gbM pairs had broader expression specificity than those in unM–unM pairs, and those in unM–teM pairs had narrower expression specificity than either. Finally, teM genes in teM–gbM pairs had broader expression specificity than those in teM–unM pairs, which in turn had broader expression specificity than those in teM–teM pairs. This suggests a potential relationship between the parental locus expression and the expression of the duplicate copy. Perhaps, certain genes may be predisposed to genic methylation changes by their expression.

### Transposons and chromatin environment associations

**Figure 5.**
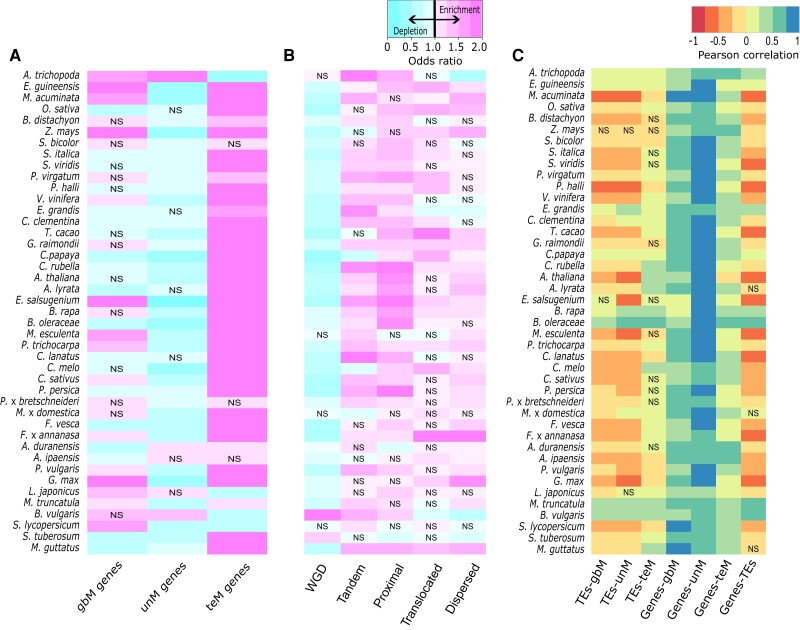
Association of TEs and chromatin environment with duplicate genes. Enrichment and depletion of TEs with duplicate genes based on **A)** genic methylation classification and **B)** type of duplication in each species. TEs within 1-kb upstream, downstream, or within the gene body were considered associated with that gene. A Fisher's exact test odds ratio of <1 represents depletion and >1 indicates enrichment. Unless indicated, all associations are statistically significant at an FDR-corrected *P* < 0.05. “NS” indicates no statistical significance. **C)** Correlation of gbM, unM, and teM genes with chromatin environment. The number of genes and number of TEs were calculated in 100-kb sliding windows with a 50-kb step size and used as a proxy for regions of euchromatin and heterochromatin. Unless indicated, all associations are statistically significant at an FDR-corrected *P* < 0.05. “NS” indicates no statistical significance.

Non-CG methylation is often associated with Transposons (TEs), and TEs can alter the gene chromatin and expression (Hirsch and Springer 2017; Raju et al. 2019). We identified TEs in or within 1 kb (Fig. 5A and Supplemental Table S19) for each paralog. teM paralogs are enriched (36/43 enriched and 4/43 depleted) and unM paralogs are depleted (3/43 enriched and 34/43 depleted) for TEs in the majority of species. gbM was enriched (15/43) and depleted (15/43) for TEs in an equal number of species. Examining duplication type (Fig. 5B and Supplemental Table S20), WGDs are depleted (2/43 enriched and 37/43 depleted) and all 4 SGDs enriched (tandem: 30/43 enriched and 3/43 depleted; proximal: 33/43 enriched and 2/43 depleted; translocated: 21/43 enriched and 2/43 depleted; dispersed: 27/43 enriched and 4/43 depleted) for TEs in the majority of species. Enrichment of TEs in SGDs may partly explain enrichment of teM in SGDs. We compared TE presence/absence for duplicate pairs differing in genic methylation (Supplemental Table S21), hypothesizing that the teM paralog would associate with TEs more frequently than its unM or gbM pair. This was true for *A. thaliana* and lyrate rockcress (*Arabidopsis lyrata*), but for most species, both paralogs are associated with a TE in gbM–teM and unM–teM pairs, suggesting a more complex relationship than simple TE presence/absence in switching of genic methylation states. Differences in TE location or the TE family may be relevant, as was shown with TEs and heterochromatin spreading in *Z. mays* (Eichten et al. 2012).

Location and chromatin environment can also affect genic methylation of duplicate genes. In *G. max*, translocation of paralogs to TE-rich pericentromeric regions often resulted in teM acquisition (El Baidouri et al. 2018). We used gene number, TE number, and fraction of TE base pairs in sliding windows across the genome as a proxy for regions of euchromatin and heterochromatin and correlated these with the number of gbM/unM/teM paralogs (Fig. 5C and Supplemental Table S22). gbM, unM, and teM duplicates are positively correlated with gene number, except *A. thaliana*, where teM has a very weak negative correlation [Pearson's *r* = −0.05, false discovery rate (FDR)-corrected *P* = 0.004]. This may be due to *A. thaliana* genomic organization, which has the smallest genome and the strongest negative correlation between total gene number and TEs (Pearson's *r* = −0.71, FDR-corrected *P* < 0.001) in our data. In most species, the distribution of gbM and unM genes is negatively correlated (gbM: 8/43 positive and 33/43 negative; unM: 10/43 positive and 31/43 negative) and teM genes positively correlated with TEs (24/43 positive and 8/43 negative). This supports the hypothesis that duplication of genes to heterochromatic regions can lead to teM acquisition; however, this does not explain cases such as SC-intermediates.

### Epiallele frequency and paralog evolution within a population

**Figure 6.**
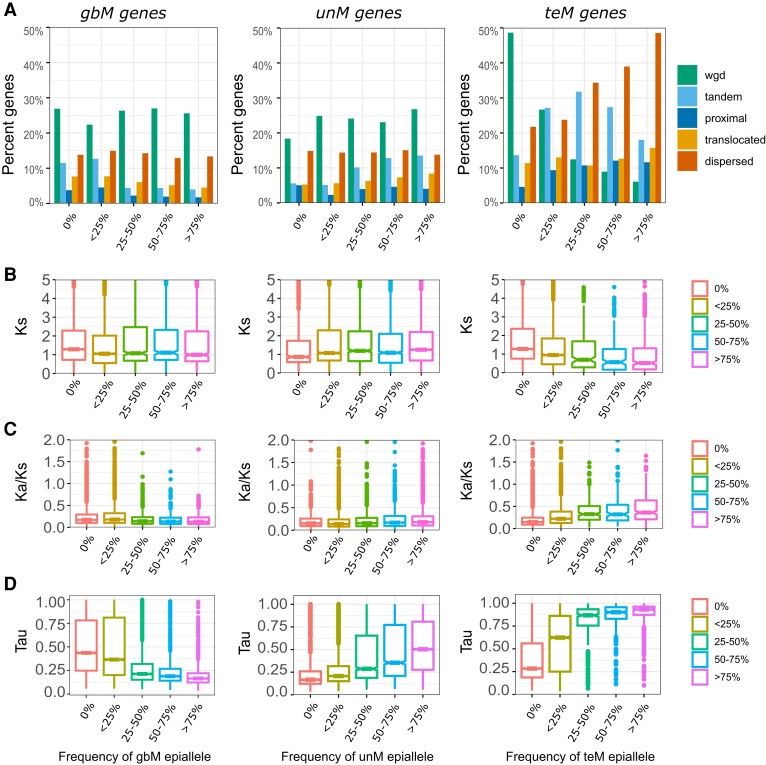
Genic methylation frequency within a population is associated with duplication type, age, sequence evolution, and expression specificity. **A)** The proportion of each type of duplication at different population frequencies of gbM, unM, and teM (0%, <25%, 25% to 50%, 50% to 75%, and >75%) in 928 *A. thaliana* accessions. **B, C, D)** The distribution of **B)** Ks (synonymous substitutions), **C)** Ka/Ks (ratio of Ka, nonsynonymous substitutions to Ks, synonymous substitutions), **D)** and tissue specificity index (*τ*) for genes at different population frequencies of gbM, unM, and teM. Center line in the boxplot represents the median Ks value and Ka/Ks ratio, while the box limits represent 25% and 75% percentile of the interquartile range; whiskers represent 1.5 times above or below the interquartile range and dots represent outliers.

DNA methylation varies across a population (Becker and Weigel 2012). The relationship between this variation and paralog evolution is unknown. To address this, genes were classified based on genic methylation and binned according to the frequency of gbM/unM/teM across 928 *A. thaliana* accessions (Kawakatsu et al. 2016). We examined the proportion of duplication type (Fig. 6A) and orthogroup (Supplemental Fig. S20) for each bin, predicting that there would be a corresponding change according to the frequency of genic methylation. This was true only in some instances. gbM showed a slight decrease in SGDs at higher frequencies, this being the most evident for tandem SGDs, while tandem SGDs continually increased with unM frequency. WGD decreased and proximal SGDs increased with increasing teM frequency, while tandem SGDs peaked at ∼25% to 50% before declining. As observed across species, a stronger association was observed for orthogroups. As the gbM frequency increases, the frequency of core:multicopy orthogroups increases, and the frequency of cross-family, family-specific, and species/lineage-specific orthogroups decreases. We observed an opposite trend with increasing teM frequency. As the unM frequency increases in the population, we observe an increase in cross-family orthogroups and a decrease in the frequency of core single-copy genes.

We examined Ks (Fig. 6B) and Ka/Ks (Fig. 6C) at different epiallele frequencies. Both gbM and unM show little variation in Ks at different frequencies, while Ks steadily decreases with increasing teM frequency. This suggests a higher frequency of teM in evolutionarily more recent paralogs. Ka/Ks decreased with increasing gbM frequency and increased with increasing teM frequency, fitting the expectation of gbM genes being under greater purifying selection and teM genes being under relaxed selective constraints. Ka/Ks increased slightly with higher unM frequency. We hypothesized that this may be related to gene expression. Supporting this hypothesis, we observed that *τ* increased (more tissue specific) at higher population frequencies of both unM and teM and decreased with increasing frequency of gbM (Fig. 6D).

## Discussion

DNA methylation has been proposed to have a role in paralog evolution (Rodin and Riggs 2003; Wang, Wang, et al. 2013; Keller and Yi 2014; Wang, Marowsky, and Fan 2014). However, this has not been examined at either a broad phylogenetic level or within a population, leaving the generalizability of results from individual species unresolved. To address this, we examined DNA methylation and paralog evolution across 43 angiosperms and a population of 928 *A. thaliana* accessions. Across the phylogeny, WGDs are broadly enriched for gbM and unM genes and depleted for teM genes. There is further differentiation between “local” SGDs (tandem and proximal) and “distal” SGDs (translocated and dispersed). Both are more frequently depleted in gbM, local duplicates are more frequently enriched for unM, while there is an increasing frequency of teM from tandem to proximal to translocated to dispersed SGDs. There are notable exceptions to these trends. For 3 Brassicaceae species (*B. oleracea*, *B. rapa*, and *E. salsugineum*), divergence from these patterns is explained by a known depletion of gbM (Bewick et al. 2016). The Cucurbitaceae and *S. tuberosum* were also depleted of WGD gbM, despite no known depletion of gbM in these species, and need further investigation. We observe an even more consistent association of genic methylation with different types of orthogroups, and this may drive patterns observed between genic methylation and gene duplication. This appears to be the case for *S. tuberosum*, a relatively recent autopolyploid (Potato Genome Sequencing Consortium et al. 2011). Unlike other species, *S. tuberosum* WGDs are enriched for increasingly lineage-specific orthogroups which explains the depletion of gbM and unM and enrichment of teM in WGDs.

gbM is characteristic of evolutionarily conserved genes (Takuno and Gaut 2012, 2013; Bewick et al. 2016; Takuno et al. 2016). Fitting this, gbM is enriched in core angiosperm genes, and gbM–gbM duplicate pairs are more conserved in sequence and expression. In contrast, teM genes are evolutionarily younger, increasing in enrichment with greater lineage specificity, and predominantly found in recent SGDs. teM paralogs have narrower expression, higher Ka/Ks ratios, and enrichment in PAV, suggesting that most are on the path to pseudogenization. This process would lead to their depletion in more ancient WGDs, while continual duplication in SGDs would result in their enrichment. unM genes are seemingly intermediate between gbM and teM in most aspects. unM might be considered the “default” state and spans from more gbM-like to more teM-like genes. In gbM-depleted species, the gbM ortholog is unM (Bewick et al. 2016). unM is the largest group and broadly represented across both core angiosperm orthogroups and more lineage-specific orthogroups. Many transcription factors and kinases are retained following WGD and have tissue-specific expression characteristic of unM (Pophaly and Tellier 2015). At the same time, many tandem and proximal duplications are associated with environmental adaptation (Freeling 2009). This would favor retention of unM in both WGDs and local SGDs. Unexpectedly, unM-containing WGD pairs typically have a higher Ks than gbM-containing pairs. We speculate that this could result from unM-containing pairs being derived from more ancient WGD events or differences in the mutation rates of gbM and unM genes.

Within a population, paralog evolution is associated with genic methylation frequency. This is especially true for teM genes, where increasing teM frequency is associated with evolutionary younger genes, narrower expression, and greater sequence divergence. Differences are also observed in gbM and unM genes and appear to be driven by expression as more narrowly expressed unM genes have a higher Ka/Ks ratio. To achieve high frequency in a population, the simplest explanation is that a genic methylation state was established early following duplication and spread with expansion of the population. Low-frequency states would reflect either relict populations (Kawakatsu et al. 2016) or cases of recent acquisition. A deeper analysis taking into account the structure and relationship of accessions in the population will provide further insight into the establishment of genic methylation states and paralog evolution.

Gene family is also an important factor, as indicated by our orthogroup analyses, and should be accounted for when trying to understand the role of DNA methylation in paralog evolution. Gene families differ in susceptibility to fractionation, some being preferentially retained, while others convergently revert to singletons. Both are thought to be dosage sensitive. The former retains duplicate copies to maintain relative dosage to other genes in the genome as explained by the gene balance hypothesis (Birchler and Veitia 2010) and is characteristic of what we termed “core:multicopy” genes. The latter “duplication-resistant” genes we have termed “core:single-copy” are thought to be under selective pressure to maintain singleton status (Paterson et al. 2006; De Smet et al. 2013; Li et al. 2016). gbM and unM mark different functional sets of core:single-copy genes and likely reflect differences in expression. While core:single-copy genes are predominantly singletons across angiosperms, in each species, some duplicate copies still persist (SC-intermediates). By contrasting these SC-intermediates to core:multicopy genes, we found that SC-intermediates have more frequent differences in genic methylation between duplicate pairs and a higher frequency of teM compared with core:multicopy genes and often duplicate pairs as a whole. SC-intermediates are predominantly syntenic genes resulting from WGD and do not differ in evolutionary age from core:multicopy genes. As such, they are unlikely to have been silenced prior to WGD, and the gain of teM would not have occurred via movement to heterochromatic regions. Despite being from conserved gene families, SC-intermediates have higher Ka/Ks ratios indicating relaxed selection and are associated with PAV. We propose that SC-intermediates are in the process of fractionation and that silencing by DNA methylation has a role in maintaining dosage and is a first step to their fractionation. However, it is unclear how these conserved genes would become silenced. Experimental approaches, including use of resynthesized or synthetic polyploids (Edger et al. 2017), may be necessary to capture this process.

It has been proposed that silencing by DNA methylation can result in retention of paralogs and their functional divergence (e.g. epigenetic complementation) (Adams et al. 2003; Rodin and Riggs 2003; Chang and Liao 2012). Alternatively, silencing may lead to pseudogenization and gene loss (Hua et al. 2013; El Baidouri et al. 2018). Neither hypothesis is necessarily wrong, and cases of both likely exist within a genome. Our results suggest that pseudogenization and loss are the predominant consequences. This is most evident for SGDs and core:single-copy angiosperm genes. However, there is suggestive evidence for epigenetic complementation in the divergence of expression states, and many teM genes are still expressed under limited conditions. Epigenetic complementation could also occur through silencing by other chromatin marks, like H3K27me3. Furthermore, many teM-containing duplicates have a Ka/Ks > 1, possibly indicating positive selection. Rapid functional divergence of SGDs was observed in grasses; many of these have characteristics similar to teM SGDs (Jiang and Assis 2019), but this will require further analysis. Our data show the genic DNA methylation marks differing evolutionary fates of duplicate genes and may have a role in maintaining dosage following gene duplication.

## Materials and methods

### Genome and methylome data

We used genomes and annotations for 58 angiosperm species (Tuskan et al. 2006; Jaillon et al. 2007; Ming et al. 2008; Sato et al. 2008; The International Brachypodium Initiative 2010; Schmutz et al. 2010; Hu et al. 2011; Bennetzen et al. 2012; D’Hont et al. 2012; Garcia-Mas et al. 2012; Lamesch et al. 2012; Paterson et al. 2012; Amborella Genome Project 2013; Guo et al. 2013; Hellsten et al. 2013; Kawahara et al. 2013; Ming et al. 2013; Motamayor et al. 2013; Sharma et al. 2013; Singh et al. 2013; Slotte et al. 2013; Yang et al. 2013; Dohm et al. 2014; Liu et al. 2014; Parkin et al. 2014; Schmutz et al. 2014; Tang et al. 2014; Wang, Haberer, et al. 2014; Bartholomé et al. 2015; VanBuren et al. 2015; Bertioli et al. 2016; Bombarely et al. 2016; Bredeson et al. 2016; Cheng et al. 2017; Daccord et al. 2017; Harkess et al. 2017; Jiao et al. 2017; Verde et al. 2017; Xu et al. 2017; Edger et al. 2018; Filiault et al. 2018; Hibrand Saint-Oyant et al. 2018; Hulse-Kemp et al. 2018; Lovell et al. 2018; McCormick et al. 2018; VanBuren et al. 2018; Wu et al. 2018; Xue et al. 2018; Barchi et al. 2019; Colle et al. 2019; Edger et al. 2019; Li et al. 2019; Valliyodan et al. 2019; Hosmani et al. 2019; Mamidi et al. 2019; Lovell et al. 2021), including 43 species (Supplemental Table S1) with whole-genome bisulfite sequencing (WGBS) data (Amborella Genome Project 2013; Seymour et al. 2014; Kim et al. 2015; Ong-Abdullah et al. 2015; Secco et al. 2015; Bertioli et al. 2016; Bewick et al. 2016; Niederhuth et al. 2016; Daccord et al. 2017; Dong et al. 2017; Picard and Gehring 2017; Song et al. 2017; Turco et al. 2017; Lü et al. 2018; Wang et al. 2018; Cheng et al. 2018b; Noshay et al. 2019; Yang et al. 2019) and additional 15 species included as outgroups (Supplemental Table S1). Genes were filtered to remove putative misannotated TEs as previously described (Bowman et al. 2017) with slight modifications. First, genes were searched against Pfam-A using hmmscan (Potter et al. 2018) filtering genes matching a curated list of TE domains (https://github.com/Childs-Lab/GC_specific_MAKER) with an *e*-value < 1e−5. Next, genes were searched against a set of transposase sequences (www.hrt.msu.edu/uploads/535/78637/Tpases020812.gz) using DIAMOND blastp (Buchfink et al. 2015) and hits with an *e*-value < 1e−10 removed.

### DNA methylation analyses

WGBS from 43 angiosperm species (Supplemental Table S1) was mapped to their respective genomes using methylpy v1.2.9 (Schultz et al. 2015). Genes were classified as gbM, teM, and unM as previously done with slight modification (Takuno and Gaut 2012; Niederhuth et al. 2016). First, a background rate was calculated for CG, CHG, CHH, and non-CG (combined CHG and CHH) methylation by averaging the percentage of methylated sites in that context across primary transcript coding regions (CDS feature) of all species. Each gene was tested for enrichment of CG, CHG, CHH, or non-CG in its primary transcript coding region against this background rate using a binomial test and *P*-values corrected for FDR by the Benjamini–Hochberg (BH) procedure (Benjamini and Hochberg 1995). Genes enriched for CG methylation with ≥10 CG sites and nonsignificant CHG or CHH methylation were classified as gbM. Genes enriched for CHG, CHH, or non-CG and ≥10 sites in that context were classified as teM. Genes with ≤1 methylated site in any context or a weighted methylation (Schultz et al. 2012) ≤2% for all contexts (CG, CHG, or CHH) were classified as unM. Genes lacking DNA methylation data were classified “missing” and those with intermediate DNA methylation levels not fitting the above criteria “unclassified.”

### Gene duplication classification

Each species was blasted against itself and Amborella (*A. trichopoda*) (outgroup) using double index alignment of next-generation sequencing data (DIAMOND) blastp (Buchfink et al. 2015). *A. thaliana* was used as the outgroup for *A. trichopoda*. Hits from the same orthogroup and an *e*-value cutoff < 1e−5 were retained. Paralogs were classified by *DupGen_finder-unique* (Qiao et al. 2019), requiring ≥5 genes for collinearity and ≤10 intervening genes to classify as “proximal” duplicates. *MCScanX-transposed* (Wang, Li, and Paterson 2013) was used to detect translocated duplicates at different epochs since species divergence (Supplemental Table S12). Genic methylation enrichment in duplication types was determined by a 2-sided Fisher's exact test (Fisher 1934) with FDR correction by BH and plotted using *heatmap.2* in *gplots*. The phylogenetic tree was generated using “V.PhyloMaker” (Jin and Qian 2019) and “phytools” (Revell 2012). To avoid overcounting, if a gene had more than 1 potential paralog, we retained the pair with the lowest *e*-value.

### Orthogroup analyses

Orthogroups were identified for protein sequences of 58 angiosperm species (Supplemental Table S1) using Orthofinder v2.5.2 (Emms and Kelly 2015; Emms and Kelly 2019), with the options “-M dendroblast -S diamond_ultra_sens, -I 1.3.” Orthogroups represented in ≥51 species (∼87.9%; Supplemental Fig. S2) were classified as “core angiosperm” orthogroups. This accounts for missing annotations and is equivalent to cutoffs in past work (Li et al. 2016). Following Li et al., we classified core angiosperm orthogroups as core:single-copy if represented by a single gene in ≥70% species and the remainder as core:multicopy. Remaining orthogroups were classified based on increasing lineage specificity: “cross family” if present in multiple plant families, “family specific” if found in a single plant family, or “species/lineage specific” if limited to a single species. Within each species, a subset of core:single-copy orthogroups still retained duplicate copies and were classified as SC-intermediates and those represented by only a single gene as SC-singletons. A 2-proportion *Z*-test was used to test differences in genic methylation between SC-intermediates and SC-singletons.

### Sequence evolution

The calculate_Ka_Ks_pipeline.pl (Qiao et al. 2019) was used to determine nonsynonymous (Ka) and synonymous substitutions (Ks) for duplicate pairs. Protein sequences are aligned by MAFFT (v7.402) (Katoh and Standley 2013), converted to a codon alignment with PAL2NAL (Suyama et al. 2006), and KaKs_Calculator 2.0 used to calculate Ka, Ks, and Ka/Ks with the γ-MYN method (Wang et al. 2010; Qiao et al. 2019). PAV variants were downloaded for *B. oleracea* (Golicz et al. 2016), *S. lycopersicum* (Gao et al. 2019), *S. tuberosum* (Hardigan et al. 2016), and *Z. mays* (Hirsch et al. 2014). For *S. tuberosum* and *Z. mays*, genes with an average read coverage of <0.2 in ≥1 accession were considered PAV. Enrichment was tested using a 2-sided Fisher's exact test with FDR correction by BH.

### Gene expression

Expression data for *A. thaliana*, *G. max*, *P. vulgaris*, and *S. bicolor* are from published expression atlases (O’Rourke et al. 2014; Klepikova et al. 2016; McCormick et al. 2018; Wang et al. 2019). *A. thaliana* reads were downloaded from NCBI SRA (PRJNA314076 and PRJNA324514), mapped with STAR (Dobin et al. 2013), and normalized for library size in DESeq2 (Love et al. 2014). *G. max*, *P. vulgaris*, and *S. bicolor* normalized data were downloaded from Phytozome (Goodstein et al. 2012). The tissue specificity index (*τ*) was calculated in R for each gene as previously described (Yanai et al. 2005). Genes not expressed under any conditions were excluded as *τ* could not be calculated. Pearson correlation coefficients were calculated for each duplicate pair in R.

### Transposons and genomic distribution

TEs were annotated de novo for all species using extensive de novo TE annotator (EDTA) (Ou et al. 2019). We calculated the total number of genes, genes belonging to each of the genic methylation classes, the number of TEs, and number of TE base pairs in 100-kb sliding windows with 50-kb steps. Pearson correlation coefficients were calculated using the “rcorr” function in “corrplot” (Wei and Viliam).

### Arabidopsis diversity

WGBS data for 928 *A. thaliana* accessions, previously aligned by methylpy (Kawakatsu et al. 2016), were downloaded from the Gene Expression Omnibus (GEO Accession GSE43857). Genes were classified as before and the frequency of gbM/unM/teM in the population calculated for each gene.

### Accession numbers

NCBI SRA accession numbers for the data sets used in the study are listed in Supplemental Table S1.

## Supplementary Material

kiad220_Supplementary_Data

## Data Availability

Raw data sources are listed in Supplemental Table S1. Formatted genomes and data are available at DataDryad https://doi.org/10.5061/dryad.n8pk0p30v. Code and scripts used in these analyses are available at: https://github.com/niederhuth/DNA-methylation-signatures-of-duplicate-gene-evolution-in-angiosperms.

## Supplemental data

The following materials are available in the online version of this article.

**Supplemental Figure S1**. Schematic representation of different orthogroup classifications.

**Supplemental Figure S2**. Distribution of orthogroups across 58 angiosperm species.

**Supplemental Figure S3**. Distribution of orthogroups in genic methylation classes.

**Supplemental Figure S4**. Enrichment or depletion of different genic methylation classes (gbM, unM, and teM) for each orthogroup category (core:multicopy, core:single-copy, cross-family, family-specific, and lineage/species-specific).

**Supplemental Figure S5**. Enrichment or depletion of different types of duplicates (whole-genome and different types of SGDs) for each orthogroup category (core:multicopy, core:single-copy, cross-family, family-specific, and lineage/species-specific).

**Supplemental Figure S6**. Proportion of paralogs with similar and divergent DNA methylation profiles.

**Supplemental Figure S7**. Distribution of genic methylation classified genes based on synonymous substitution (Ks) across different types of gene duplicate pairs.

**Supplemental Figure S8**. Proportion of different genic methylation in transposed duplicates across different epochs.

**Supplemental Figure S9**. Distribution of core:multicopy and core:single-copy (intermediate) paralogs based on synonymous substitutions (Ks).

**Supplemental Figure S10**. Distribution of genic methylation classified genes based on the ratio of nonsynonymous substitution (Ka), with synonymous substitutions (Ks) across different types of gene duplicate pairs.

**Supplemental Figure S11**. Distribution of core:multicopy and core:single-copy (intermediate) paralogs based on the ratio of nonsynonymous substitution (Ka), with synonymous substitutions (Ks).

**Supplemental Figure S12**. Percentage of total (all genes), gbM, teM, and unM genes with known PAV.

**Supplemental Figure S13**. Proportion of SC-singletons and SC-intermediates in PAV genes in *B. oleracea*, *S. lycopersicum*, *S. tuberosum*, and *Z. mays*.

**Supplemental Figure S14**. Tau specificity of gbM, teM, and unM genes in *G. max*, *P. vulgaris*, and *S. bicolor*.

**Supplemental Figure S15**. Tau specificity of gbM, teM, and unM genes for each orthogroup category in *A. thaliana*, *G. max*, *P. vulgaris*, and *S. bicolor*.

**Supplemental Figure S16**. Tau specificities of different types of duplicate genes in *A. thaliana*, *G. max*, *P. vulgaris*, and *S. bicolor*.

**Supplemental Figure S17**. Half plots showing gene expression correlations of duplicate pairs based on genic methylation (gbM–gbM, gbM–teM, teM–teM, unM–unM, gbM–unM, and unM–teM) in *A. thaliana*, *G. max*, *P. vulgaris*, and *S. bicolor*.

**Supplemental Figure S18**. Absolute differences in tau specificity between duplicate pairs in *G. max*, *P. vulgaris*, and *S. bicolor*.

**Supplemental Figure S19**. Distribution of tau specificities for gbM, unM, and teM genes separated based on the methylation of their duplicate pair for *G. max*, *P. vulgaris*, and *S. bicolor*.

**Supplemental Figure S20**. Proportion of different orthogroup classifications and gbM/unM/teM epiallele frequency within *A. thaliana* population.

**Supplemental Table S1**. Genomes, methylomes, and mapping statistics for data used in the study.

**Supplemental Table S2**. Classification of genic methylation of all genes in each species.

**Supplemental Table S3**. Number of genes derived from different types of duplications in each species.

**Supplemental Table S4**. Enrichment and depletion of genic methylation classifications across different types of gene duplicates.

**Supplemental Table S5**. Enrichment and depletion of genic methylation classified genes across different orthogroup classifications.

**Supplemental Table S6**. Enrichment and depletion of different types of gene duplicates across each orthogroup category.

**Supplemental Table S7**. Number of duplicate gene pairs with different or the same genic methylation status for each type of duplication in each species.

**Supplemental Table S8**. Proportions of genes in each genic methylation class for the parental and daughter copies of translocated genes for each species.

**Supplemental Table S9**. Number of genes with similar or divergent methylation profiles between parental and translocated duplicates.

**Supplemental Table S10**. Top 5 functional categories for each GO enrichment comparison for gbM core:single-copy genes and unM core:single-copy genes across 33 species.

**Supplemental Table S11**. Duplicate pair classifications ranked based on median Ks values for SGDs and WGDs.

**Supplemental Table S12**. Outgroup species used for each epoch as part of MCscanX-transposed.

**Supplemental Table S13**. Enrichment and depletion of genic methylation classifications across different epochs of transposed duplicates for all species.

**Supplemental Table S14**. Duplicate pair classifications ranked based on median Ka/Ks values for SGDs and WGDs.

**Supplemental Table S15**. Enrichment and depletion of different types of duplicate genes with Ka/Ks ratio > 1.0.

**Supplemental Table S16**. Enrichment and depletion of different classifications of duplicate gene pairs with Ka/Ks ratio > 1.0.

**Supplemental Table S17**. Enrichment and depletion of known presence–absence variants for gbM, teM, and unM genes.

**Supplemental Table S18**. Differences in the distribution of absolute difference in tau for duplicate gene pairs with similar or divergence in methylation.

**Supplemental Table S19**. Enrichment and depletion of TEs with gbM, teM, and unM paralogs in each species.

**Supplemental Table S20**. Enrichment and depletion of TEs with different types of duplication in each species.

**Supplemental Table S21**. Presence/absence of TEs in the gene body and 1-kb upstream and 1-kb downstream for duplicate paralogs differing in their genic methylation.

**Supplemental Table S22**. Correlations between genic methylation classes (gbM, teM, and unM) and genomic features (number of genes, TEs, and TE base pairs) in 100-kb sliding windows with a 50-kb step size.
