# Supplementary material for: DNA methylation signatures of duplicate gene evolution in angiosperms
Source: Plant Physiol. 2023 Apr 15;192(4):2883–901. doi: 10.1093/plphys/kiad220 (PMC10400039; doi:10.1093/plphys/kiad220)

Supplemental Figure S1: Schematic representation of different orthogroup classification.

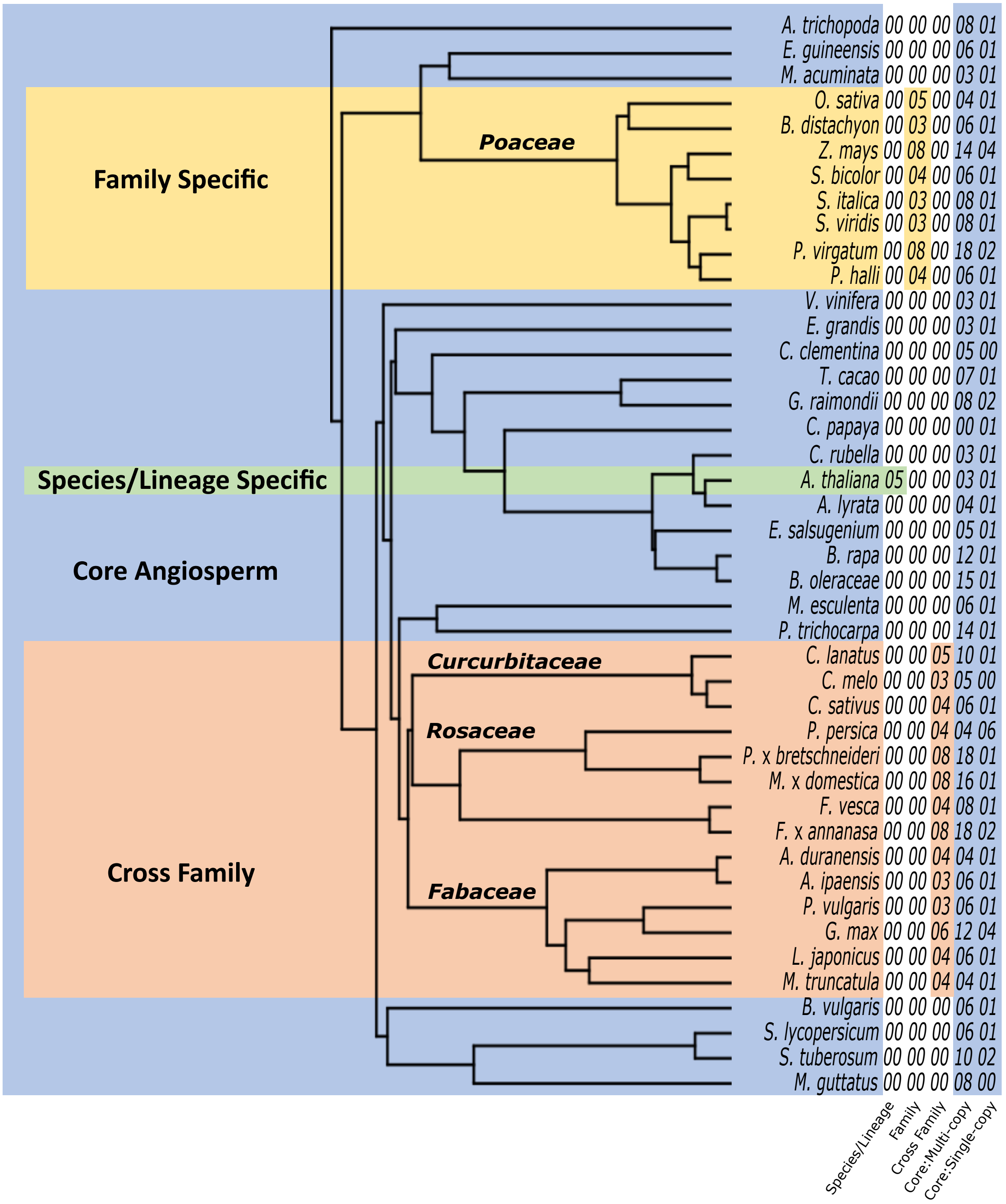

Supplemental Figure S2: Distribution of orthogroups across 58 angiosperm species.
Histogram showing the number of orthogroups represented in 1 to 58 species (A) and the same plot zoomed into species with 2 to 58 species (B). Orange colored bars represent those orthogroups classified as ‘core angiosperm’.

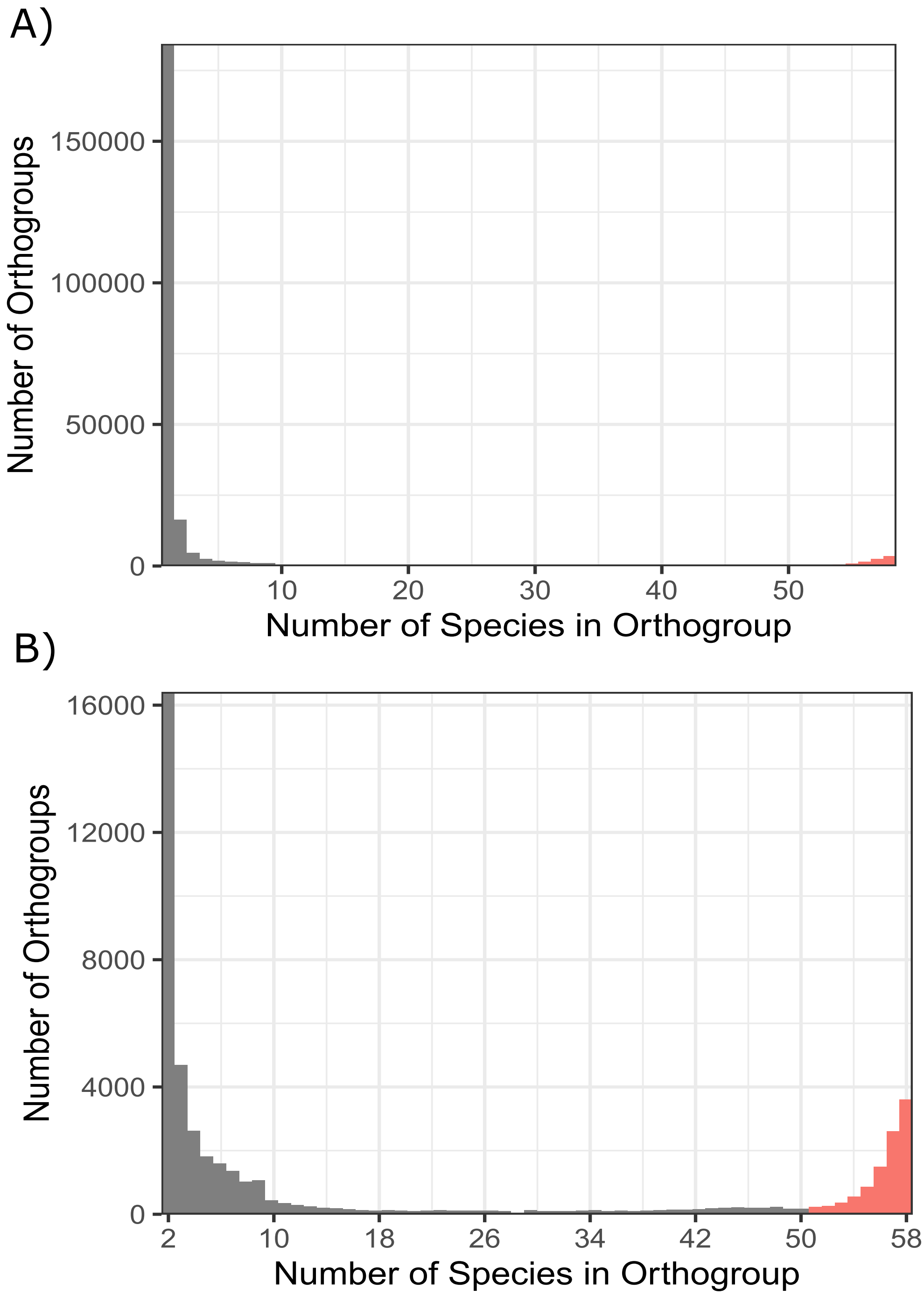

Supplemental Figure S3: Distribution of orthogroups in genic methylation classes.
A) For each species, the percentage of genes classified into different orthogroup categories (core:single-copy, core: multi-copy, cross-family, family-specific, and species/lineage-specific) in each of the three genic methylation classification (gbM, teM, and unM genes).
B) Distribution of genes classified as gbM, unM, teM, unclassified, and 'missing methylation data' across different orthogroup classifications (Core:single copy, core:multi-copy, cross-family, family-specific, and species/lineage specific)

A)

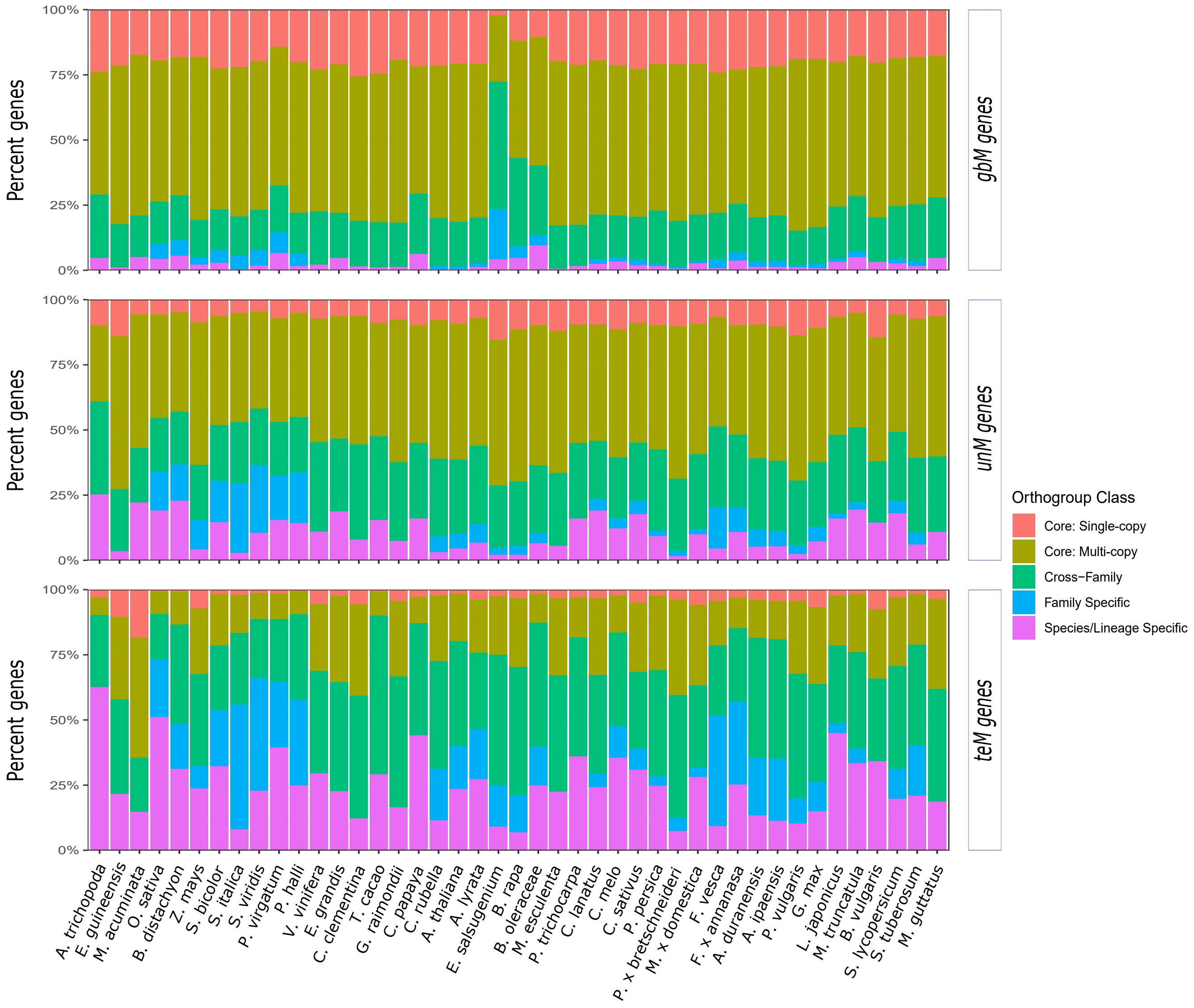

Supplemental Figure S3: Distribution of orthogroups in genic methylation classes.
A) For each species, the percentage of genes classified into different orthogroup categories (core:single-copy, core: multi-copy, cross-family, family-specific, and species/lineage-specific) in each of the three genic methylation classification (gbM, teM, and unM genes).
B) Distribution of genes classified as gbM, unM, teM, unclassified, and 'missing methylation data' across different orthogroup classifications (Core:single copy, core:multi-copy, cross-family, family-specific, and species/lineage specific)

B)

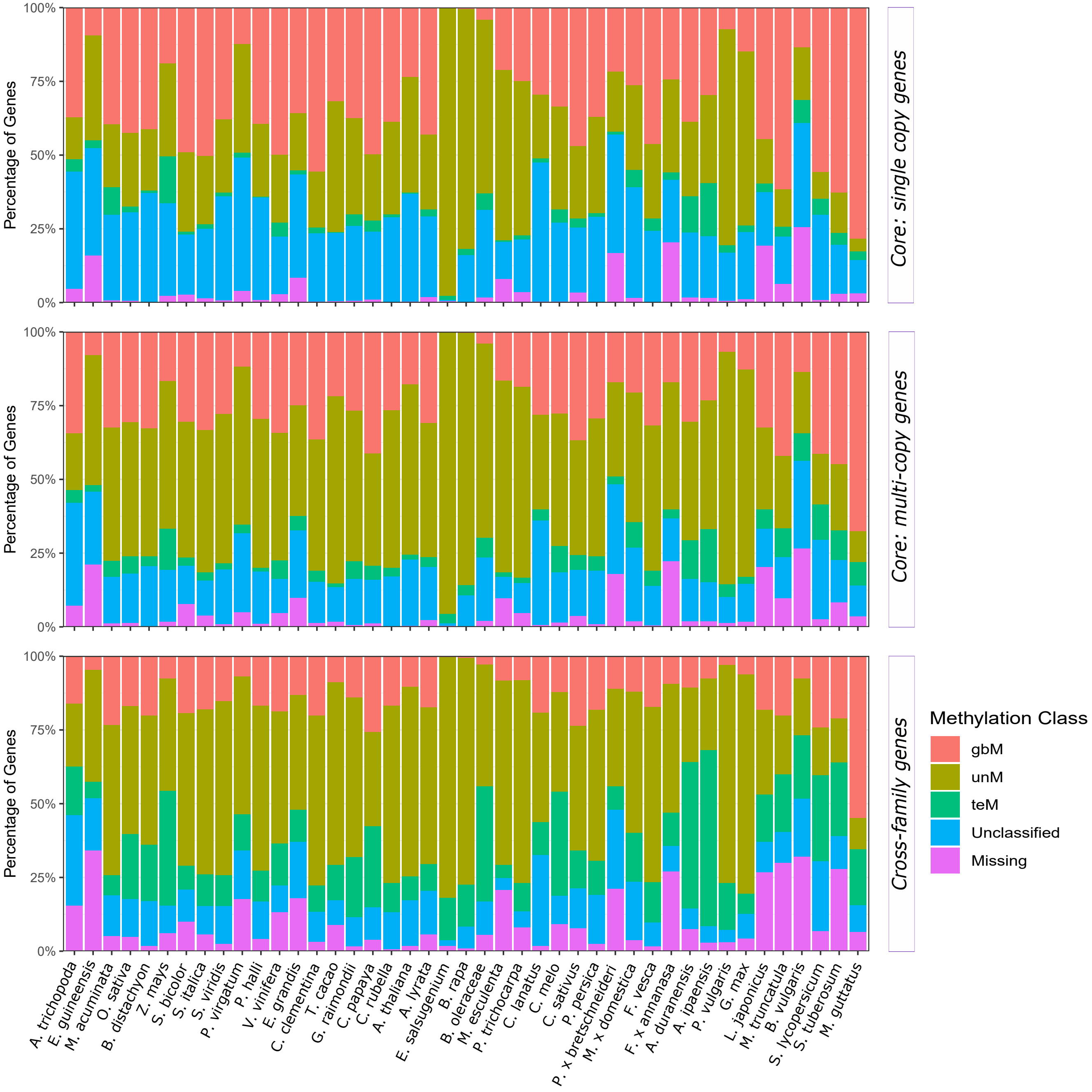

Supplemental Figure S3: Distribution of orthogroups in genic methylation classes.
A) For each species, the percentage of genes classified into different orthogroup categories (core:single-copy, core: multi-copy, cross-family, family-specific, and species/lineage-specific) in each of the three genic methylation classification (gbM, teM, and unM genes).
B) Distribution of genes classified as gbM, unM, teM, unclassified, and 'missing methylation data' across different orthogroup classifications (Core:single copy, core:multi-copy, cross-family, family-specific, and species/lineage specific)

B)

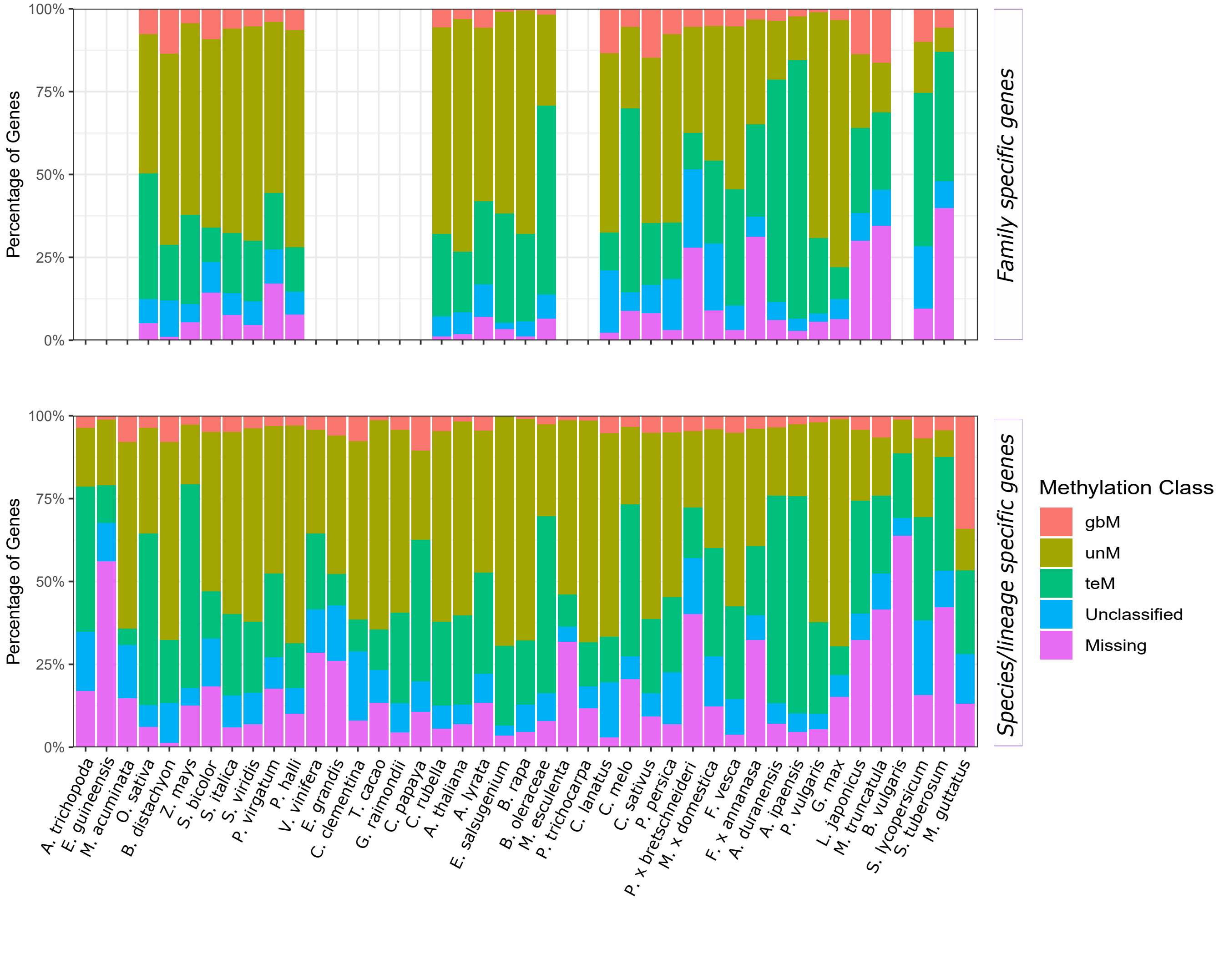

Supplemental Figure S4: **Enrichment or depletion of different genic methylation classes (gbM, unM, and teM) for each orthogroup category (core:multi-copy, core:single-copy, cross-family, family-specific, and lineage/species-specific).** Increasing shades of cyan indicates greater depletion, while increasing shades of magenta represents greater enrichment. Unless indicated, all associations are statistically significant at a FDR-corrected p-value < 0.05. 'NS' indicates no statistical significance.

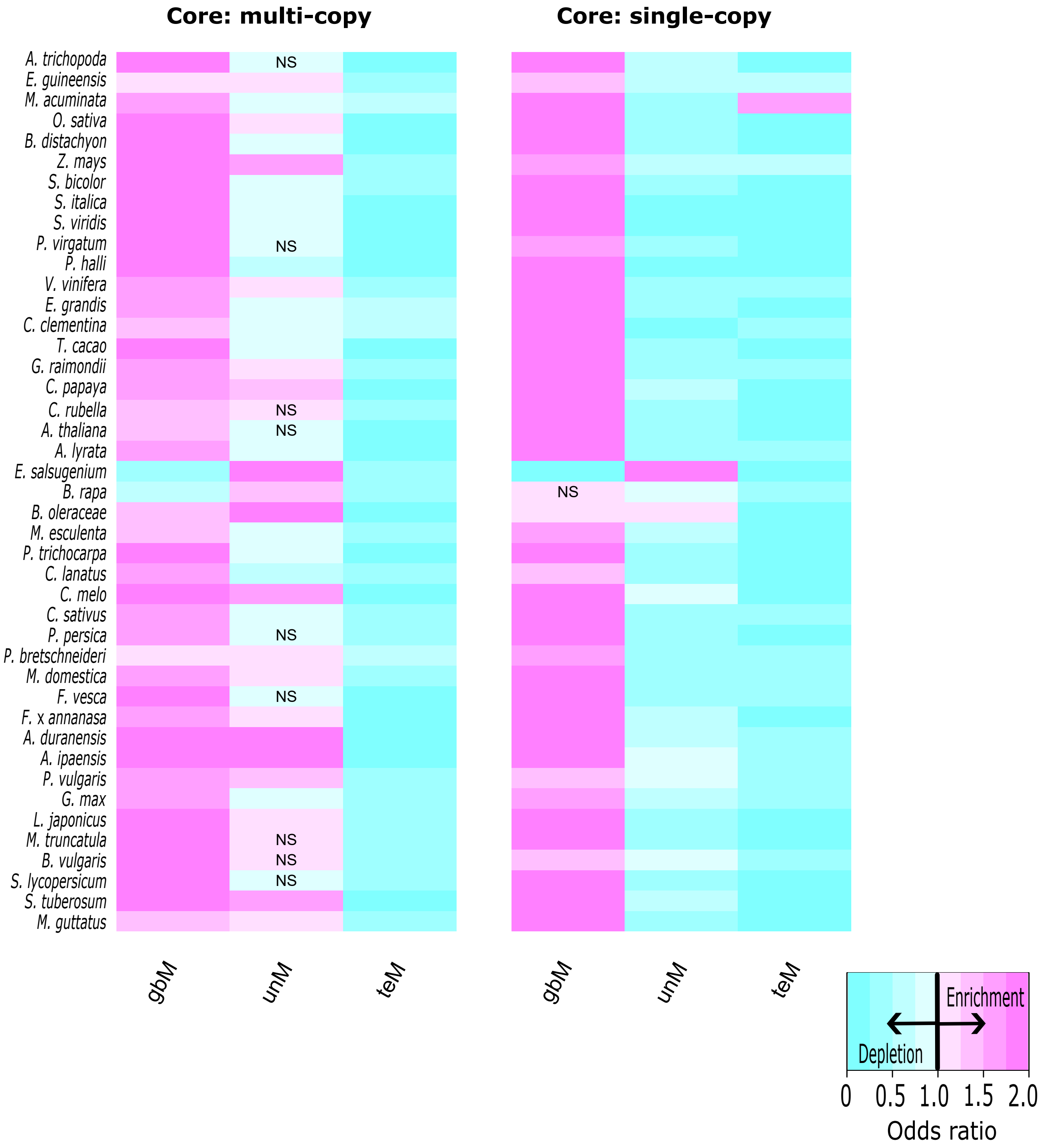

Supplemental Figure S4: **Enrichment or depletion of different genic methylation classes (gbM, unM, and teM) for each orthogroup category (core:multi-copy, core:single-copy, cross-family, family-specific, and lineage/species-specific).** Increasing shades of cyan indicates greater depletion, while increasing shades of magenta represents greater enrichment. Unless indicated, all associations are statistically significant at a FDR-corrected p-value < 0.05. 'NS' indicates no statistical significance.

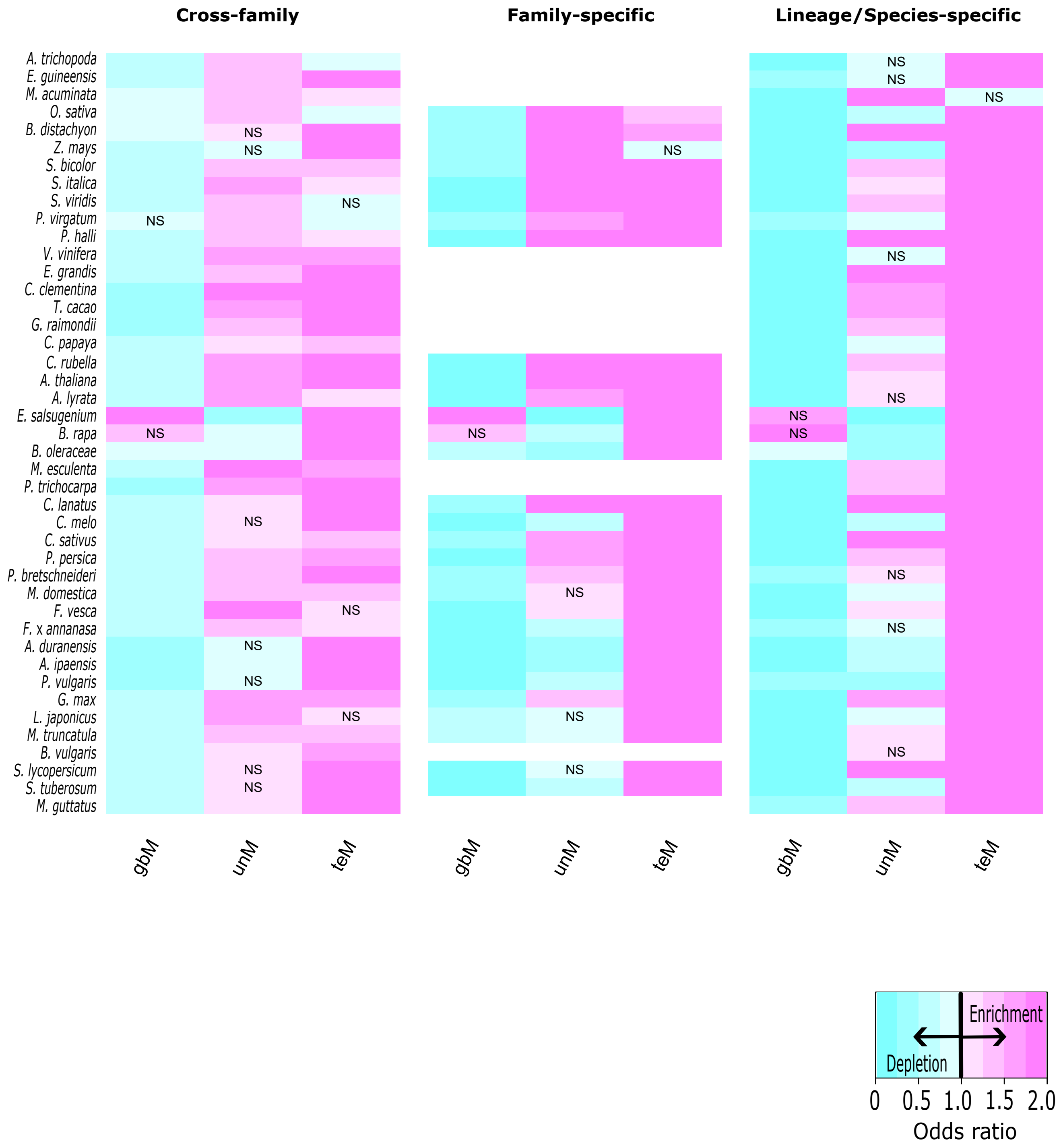

Supplemental Figure S5: **Enrichment or depletion of different types of duplicates (whole-genome, and four different types of single gene duplicates) for each orthogroup category (core:multi-copy, core:single-copy, cross-family, family-specific, and lineage/species-specific)**. Increasing shades of cyan indicates greater depletion, while increasing shades of magenta represents greater enrichment. Unless indicated, all associations are statistically significant at a FDR-corrected p-value < 0.05. 'NS' indicates no statistical significance.

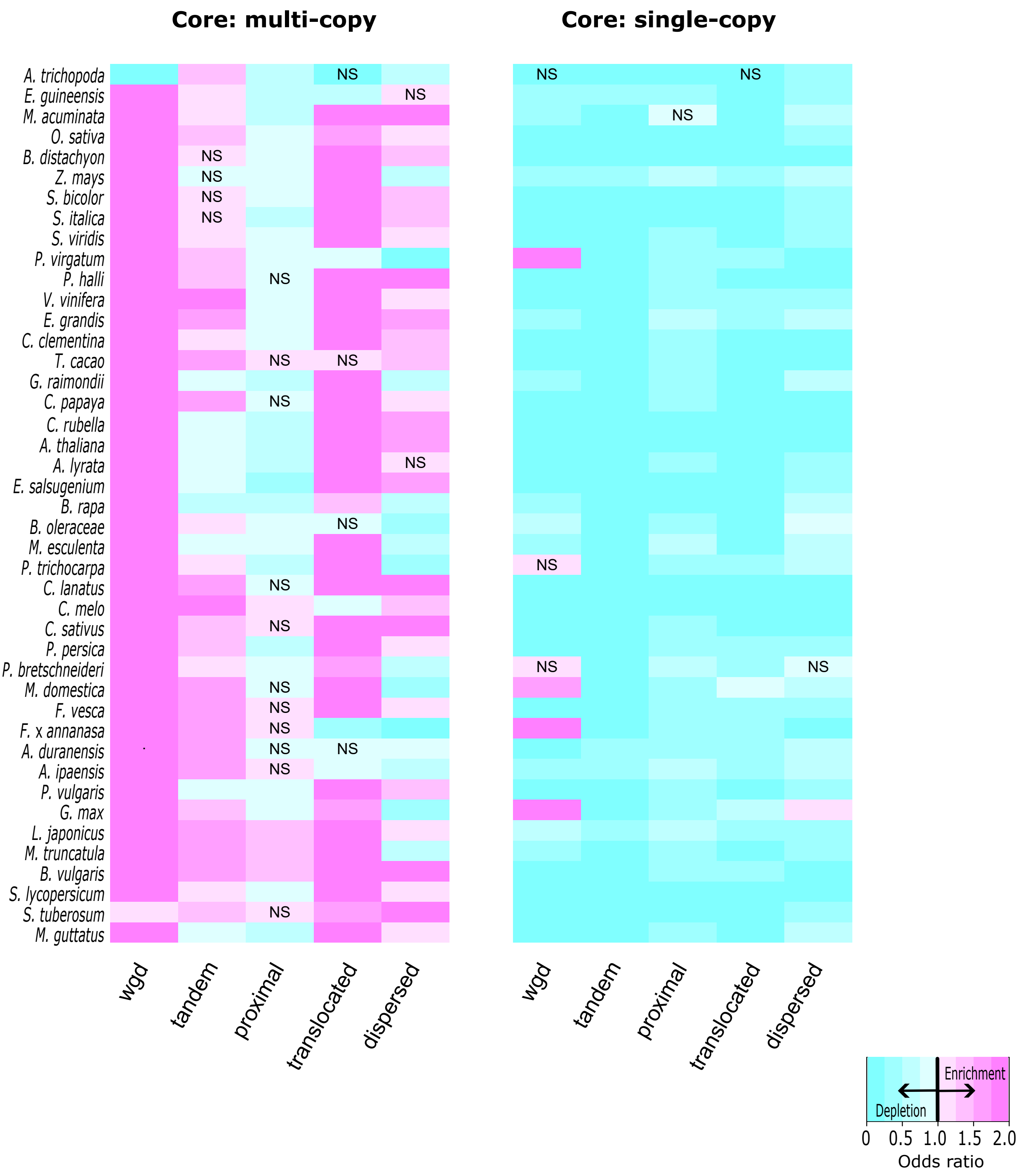

Supplemental Figure S5: **Enrichment or depletion of different types of duplicates (whole-genome, and four different types of single gene duplicates) for each orthogroup category (core:multi-copy, core:single-copy, cross-family, family-specific, and lineage/species-specific)**. Increasing shades of cyan indicates greater depletion, while increasing shades of magenta represents greater enrichment. Unless indicated, all associations are statistically significant at a FDR-corrected p-value < 0.05. 'NS' indicates no statistical significance.

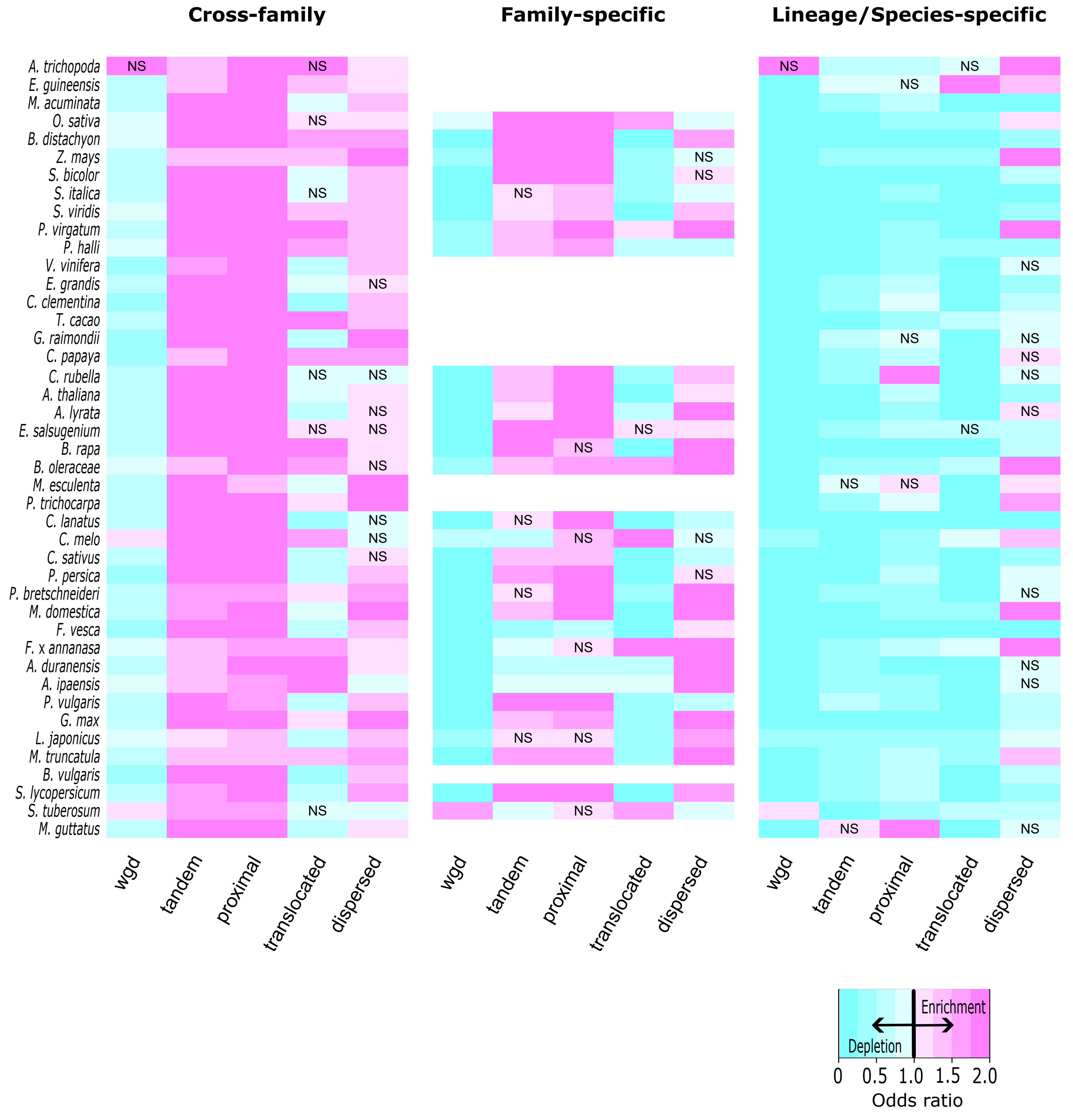

Supplemental Figure S6: **Proportion of paralogs with similar and divergent DNA methylation profiles.** The proportion of duplicate pairs with similar DNA methylation profiles among different types of duplicate genes (Whole-genome duplicates - WGD, Single-gene duplicates - tandem, proximal, translocated, and dispersed) are shown in Blue. Yellow bars represent proportion of duplicate pairs with divergent DNA methylation profiles. Grey bars represent cases where DNA methylation status of at least one of the duplicate pairs was 'undetermined'.

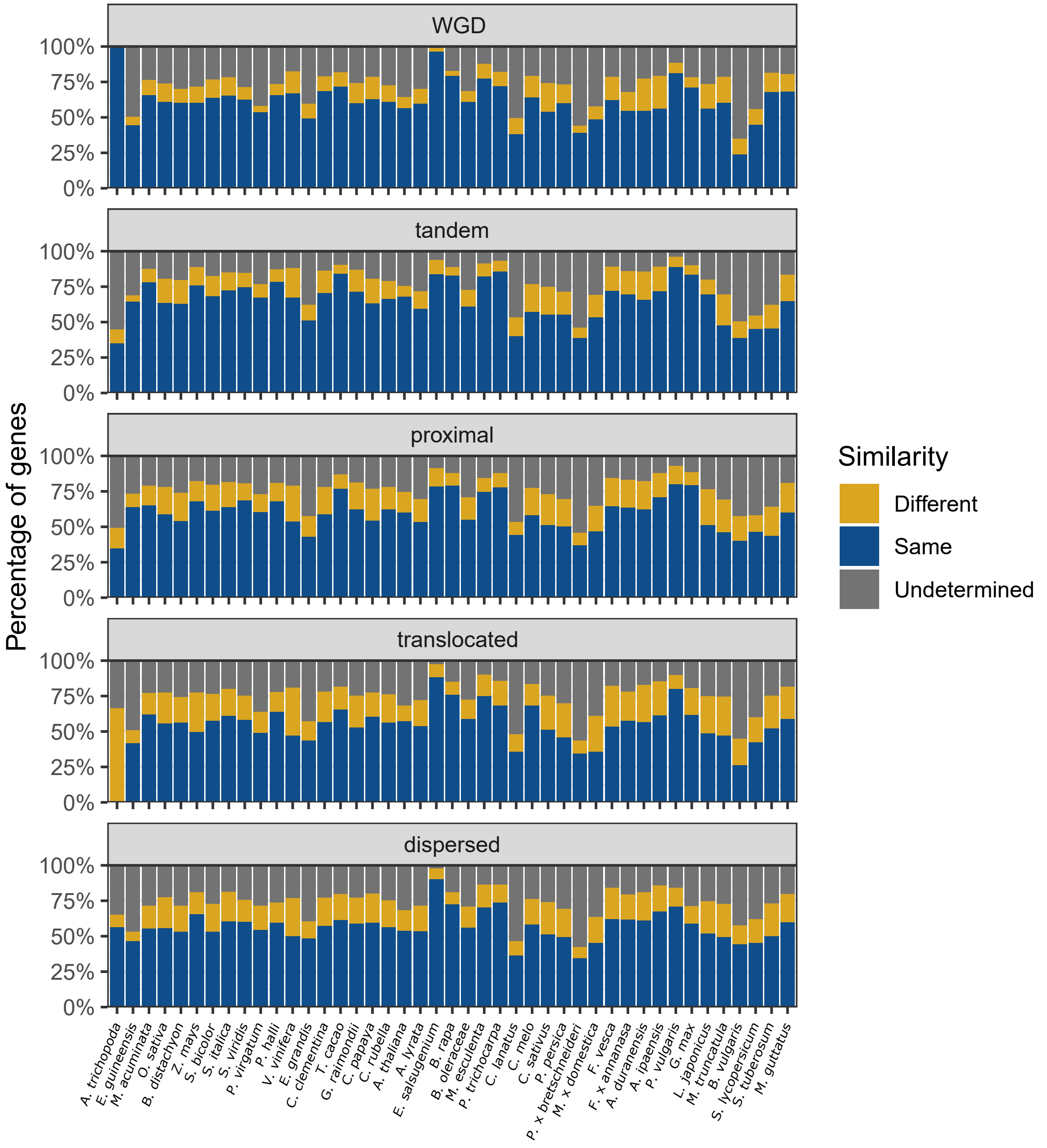

Supplemental Figure S7: **Distribution of genic methylation classified genes based on synonymous substitution (Ks) across different types of gene duplicate pairs.** Whole-genome duplicates - WGD, Single-gene duplicates - SGD (combined data from tandem, proximal, translocated, and dispersed duplicates). Center line in the boxplot represents the median Ks values, while the box limits represent 25% and 75% percentile of the interquartile range, whiskers represent 1.5 times above or below the interquartile range and dots represents outliers.

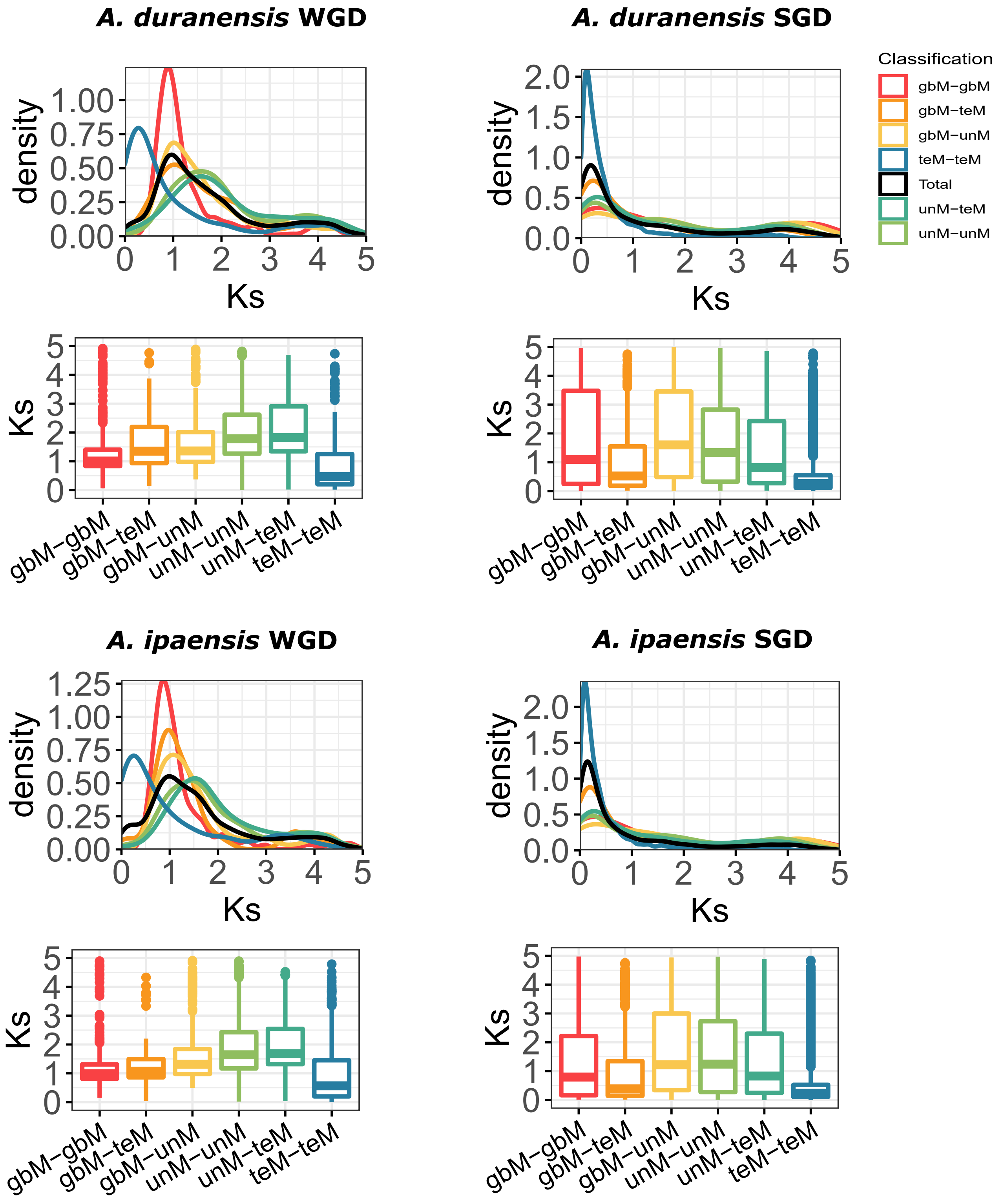

Supplemental Figure S7: **Distribution of genic methylation classified genes based on synonymous substitution (Ks) across different types of gene duplicate pairs.** Whole-genome duplicates - WGD, Single-gene duplicates - SGD (combined data from tandem, proximal, translocated, and dispersed duplicates). Center line in the boxplot represents the median Ks values, while the box limits represent 25% and 75% percentile of the interquartile range, whiskers represent 1.5 times above or below the interquartile range and dots represents outliers.

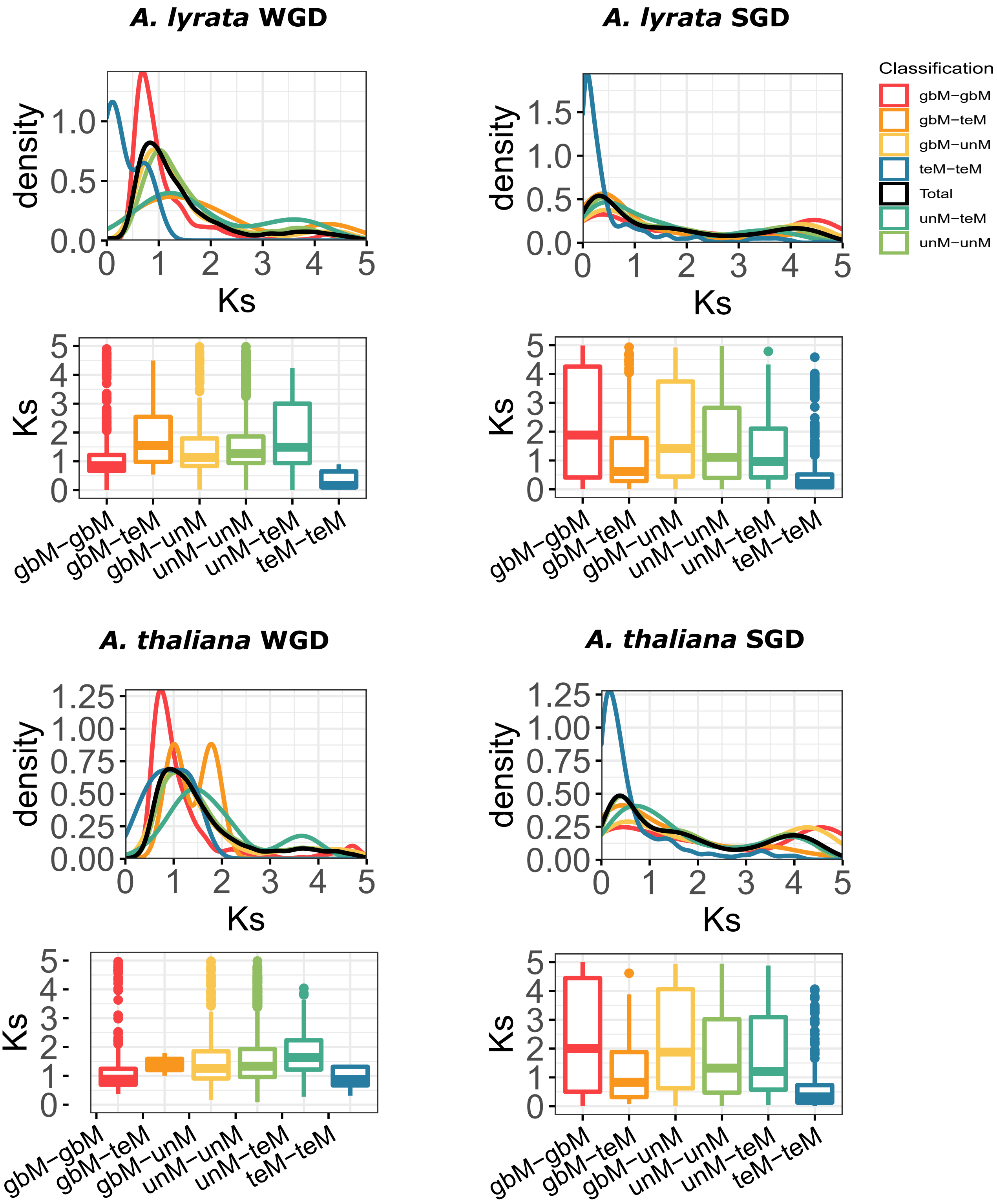

Supplemental Figure S7: **Distribution of genic methylation classified genes based on synonymous substitution (Ks) across different types of gene duplicate pairs.** Whole-genome duplicates - WGD, Single-gene duplicates - SGD (combined data from tandem, proximal, translocated, and dispersed duplicates). Center line in the boxplot represents the median Ks values, while the box limits represent 25% and 75% percentile of the interquartile range, whiskers represent 1.5 times above or below the interquartile range and dots represents outliers.

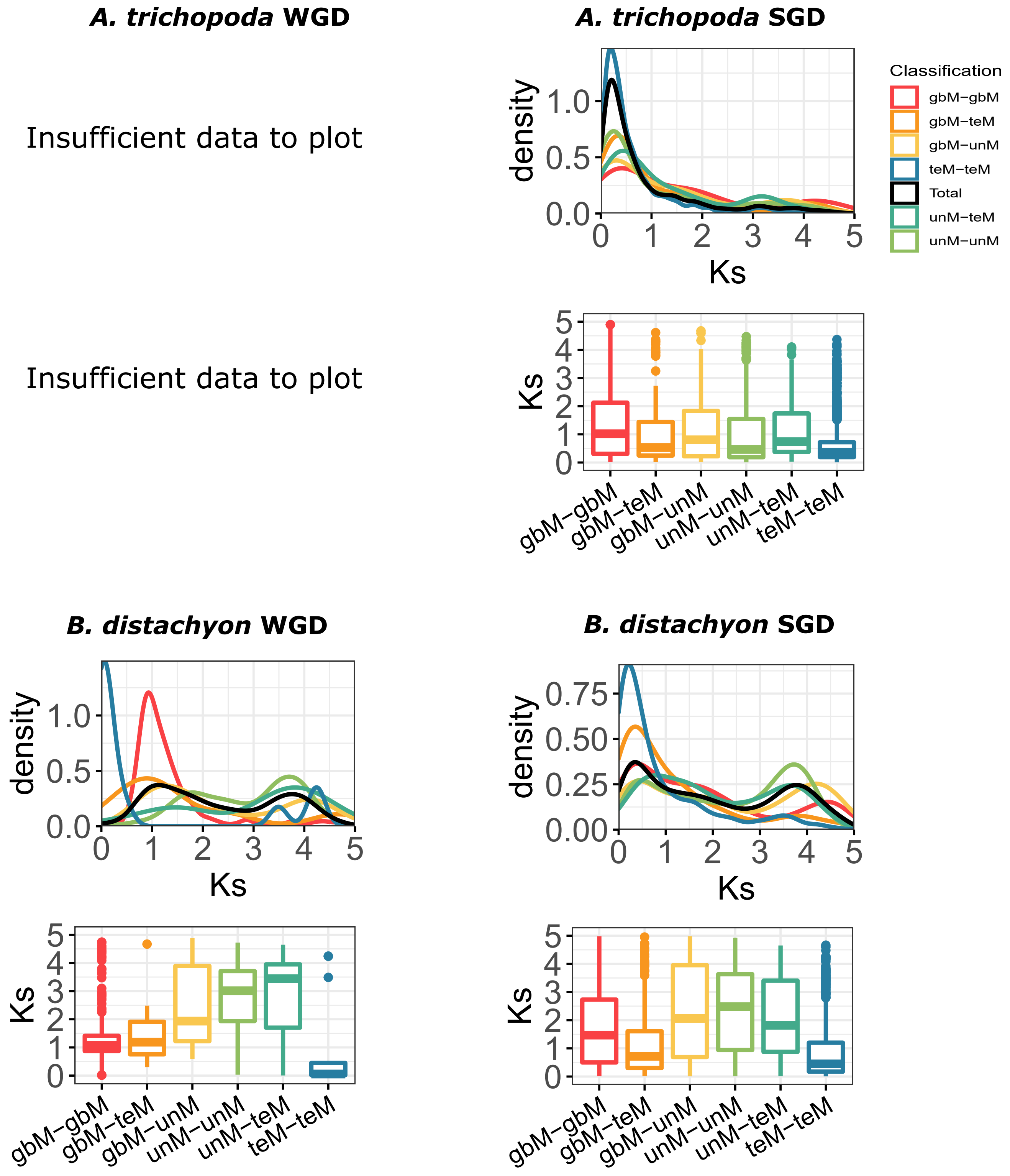

Supplemental Figure S7: **Distribution of genic methylation classified genes based on synonymous substitution (Ks) across different types of gene duplicate pairs.** Whole-genome duplicates - WGD, Single-gene duplicates - SGD (combined data from tandem, proximal, translocated, and dispersed duplicates). Center line in the boxplot represents the median Ks values, while the box limits represent 25% and 75% percentile of the interquartile range, whiskers represent 1.5 times above or below the interquartile range and dots represents outliers.

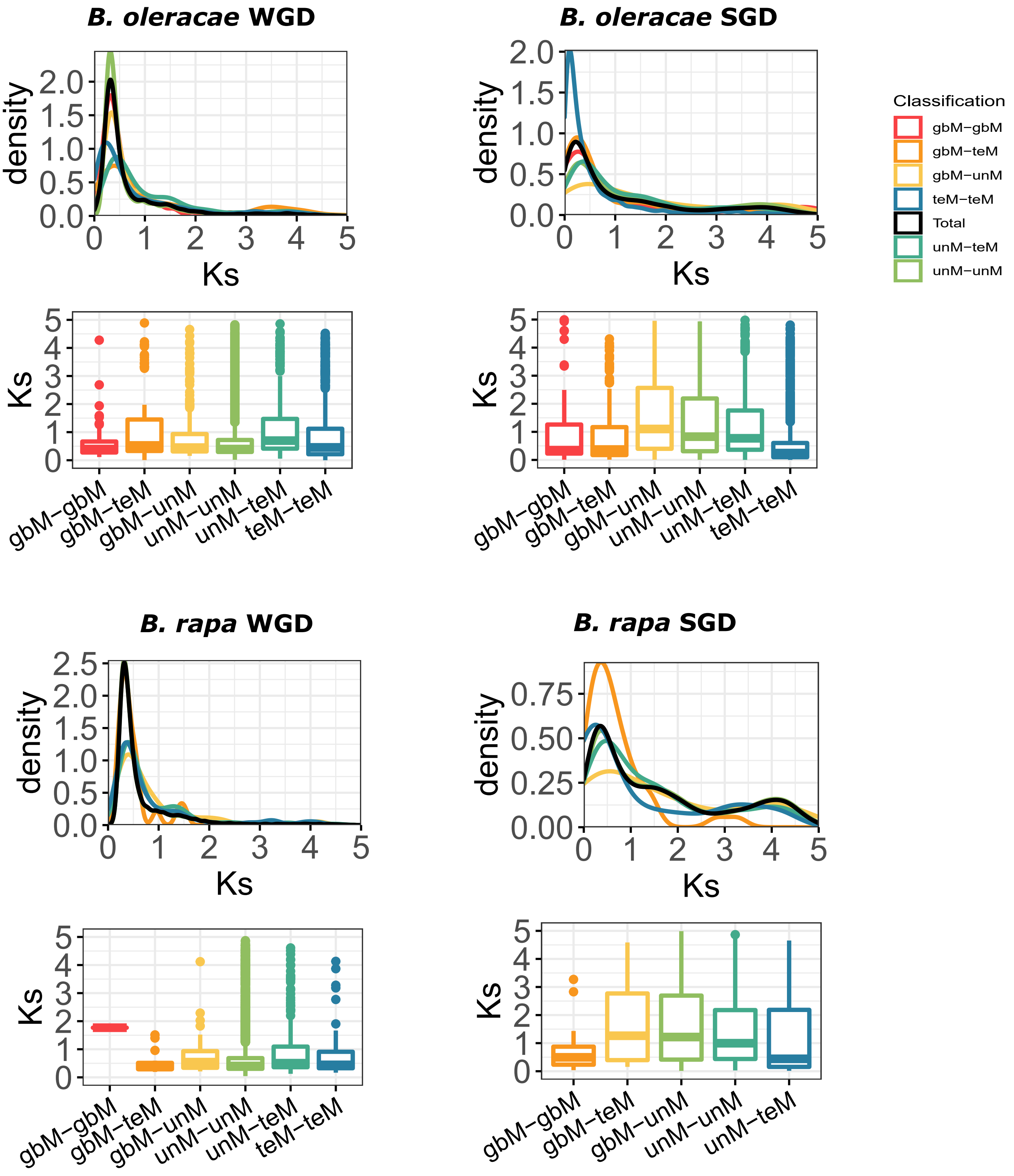

Supplemental Figure S7: **Distribution of genic methylation classified genes based on synonymous substitution (Ks) across different types of gene duplicate pairs.** Whole-genome duplicates - WGD, Single-gene duplicates - SGD (combined data from tandem, proximal, translocated, and dispersed duplicates). Center line in the boxplot represents the median Ks values, while the box limits represent 25% and 75% percentile of the interquartile range, whiskers represent 1.5 times above or below the interquartile range and dots represents outliers.

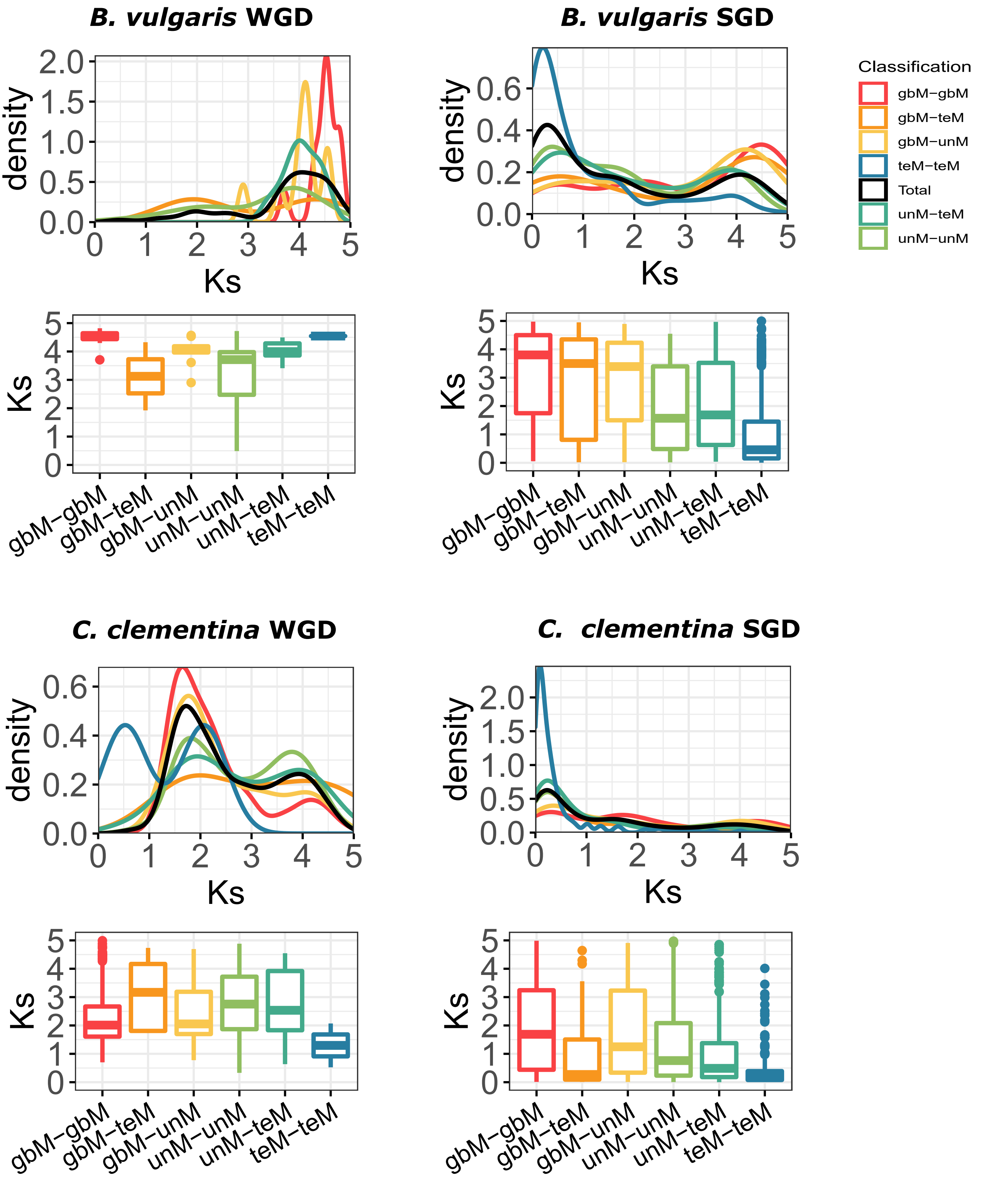

Supplemental Figure S7: **Distribution of genic methylation classified genes based on synonymous substitution (Ks) across different types of gene duplicate pairs.** Whole-genome duplicates - WGD, Single-gene duplicates - SGD (combined data from tandem, proximal, translocated, and dispersed duplicates). Center line in the boxplot represents the median Ks values, while the box limits represent 25% and 75% percentile of the interquartile range, whiskers represent 1.5 times above or below the interquartile range and dots represents outliers.

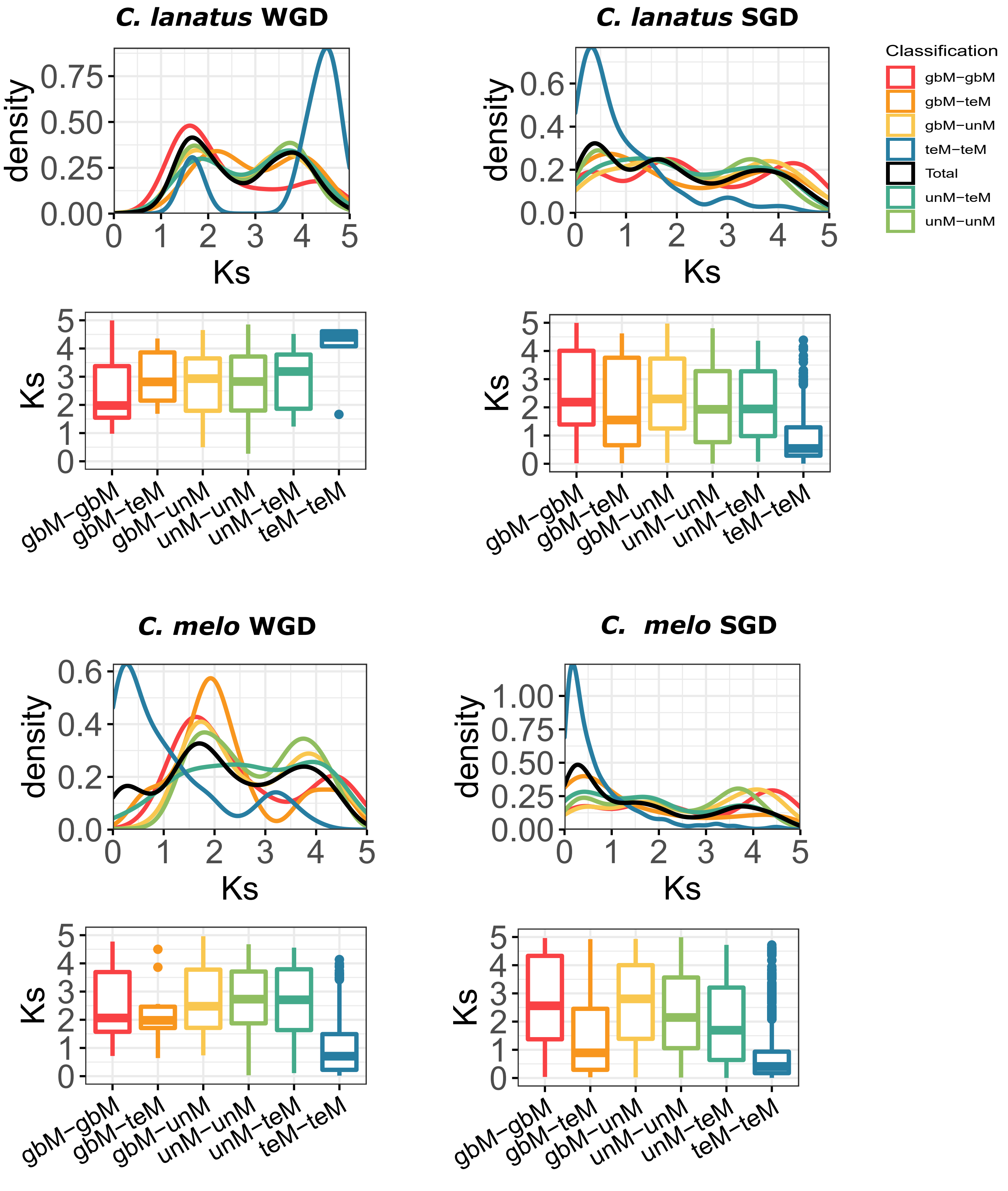

Supplemental Figure S7: **Distribution of genic methylation classified genes based on synonymous substitution (Ks) across different types of gene duplicate pairs.** Whole-genome duplicates - WGD, Single-gene duplicates - SGD (combined data from tandem, proximal, translocated, and dispersed duplicates). Center line in the boxplot represents the median Ks values, while the box limits represent 25% and 75% percentile of the interquartile range, whiskers represent 1.5 times above or below the interquartile range and dots represents outliers.

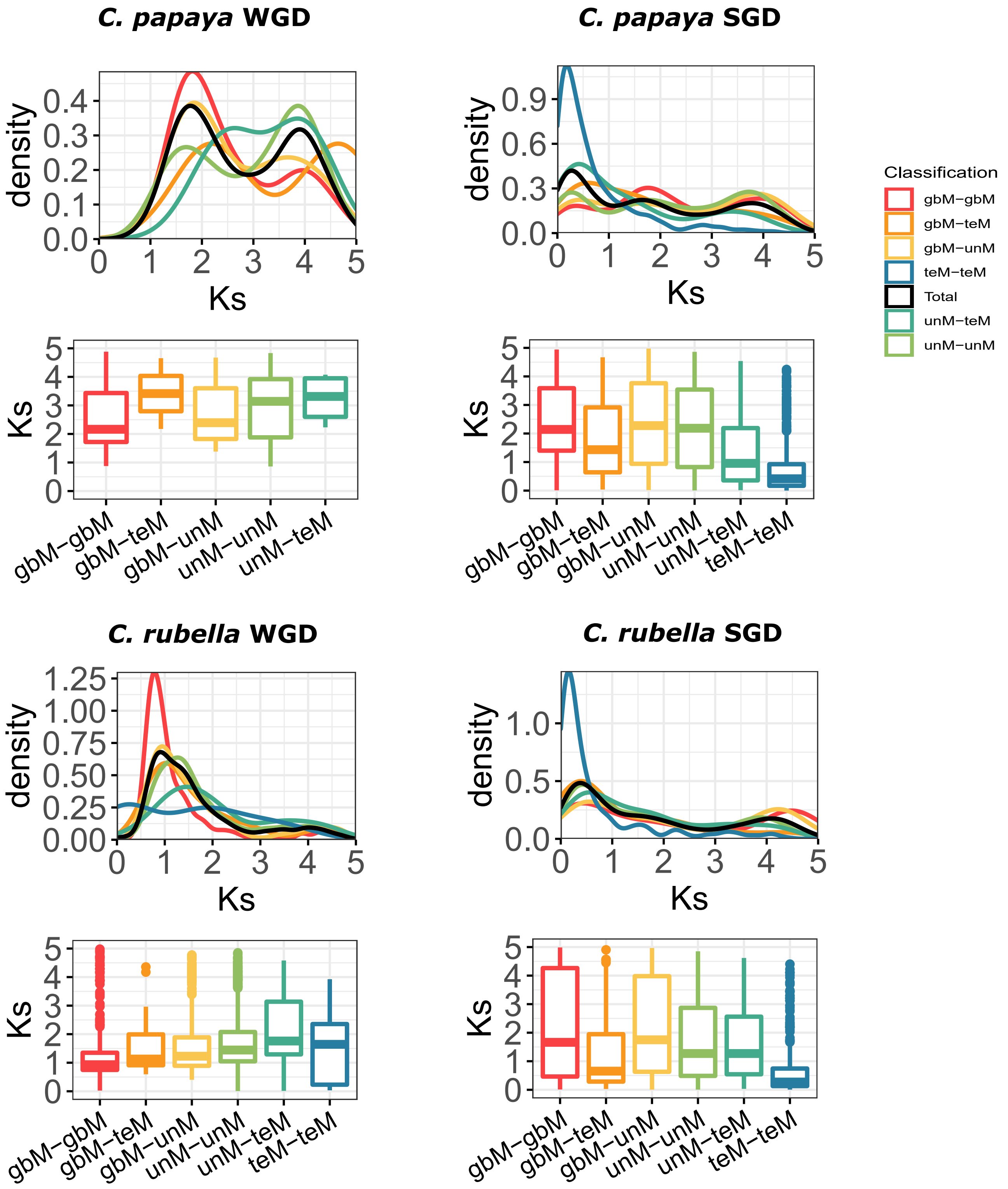

Supplemental Figure S7: **Distribution of genic methylation classified genes based on synonymous substitution (Ks) across different types of gene duplicate pairs.** Whole-genome duplicates - WGD, Single-gene duplicates - SGD (combined data from tandem, proximal, translocated, and dispersed duplicates). Center line in the boxplot represents the median Ks values, while the box limits represent 25% and 75% percentile of the interquartile range, whiskers represent 1.5 times above or below the interquartile range and dots represents outliers.

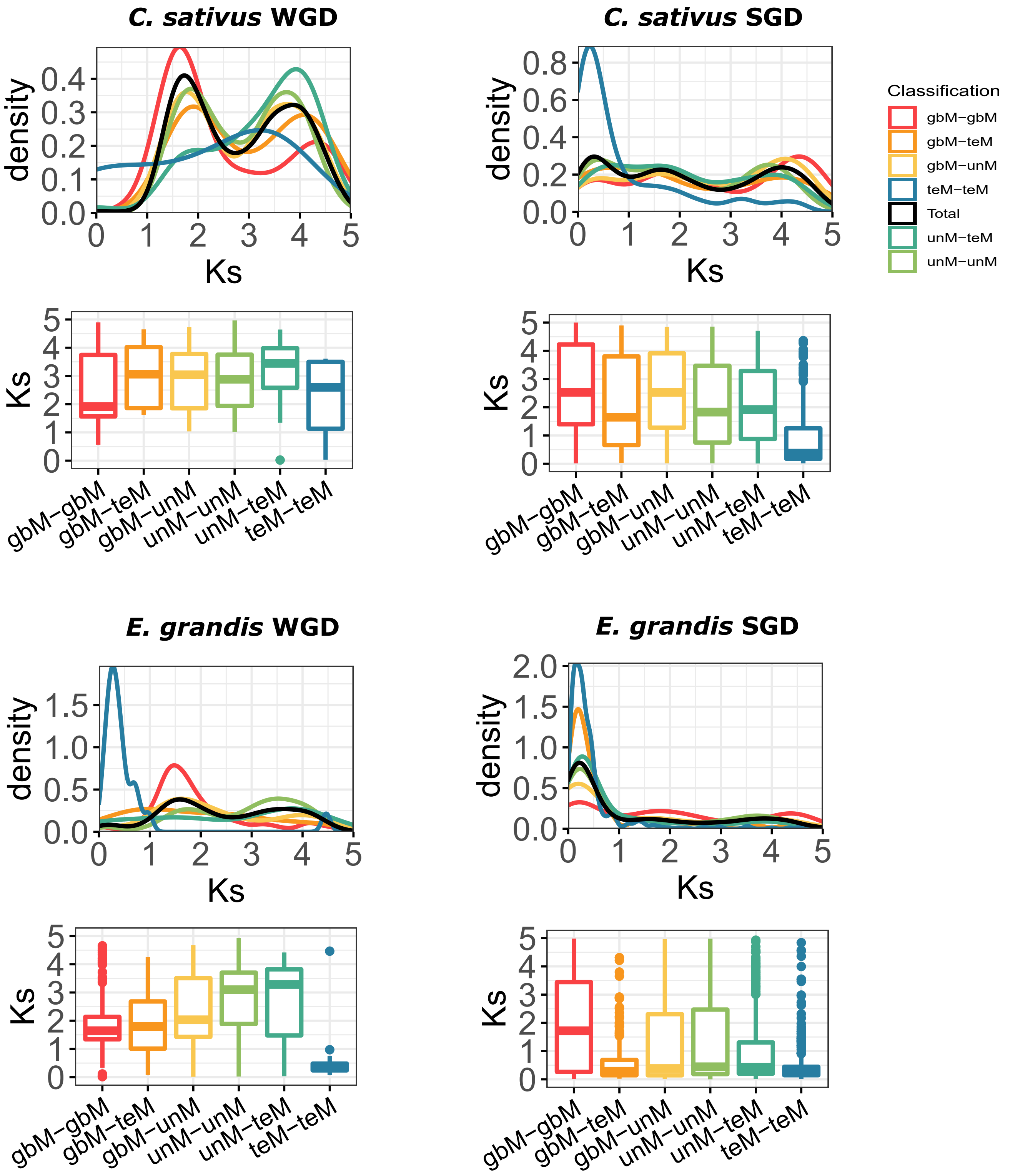

Supplemental Figure S7: **Distribution of genic methylation classified genes based on synonymous substitution (Ks) across different types of gene duplicate pairs.** Whole-genome duplicates - WGD, Single-gene duplicates - SGD (combined data from tandem, proximal, translocated, and dispersed duplicates). Center line in the boxplot represents the median Ks values, while the box limits represent 25% and 75% percentile of the interquartile range, whiskers represent 1.5 times above or below the interquartile range and dots represents outliers.

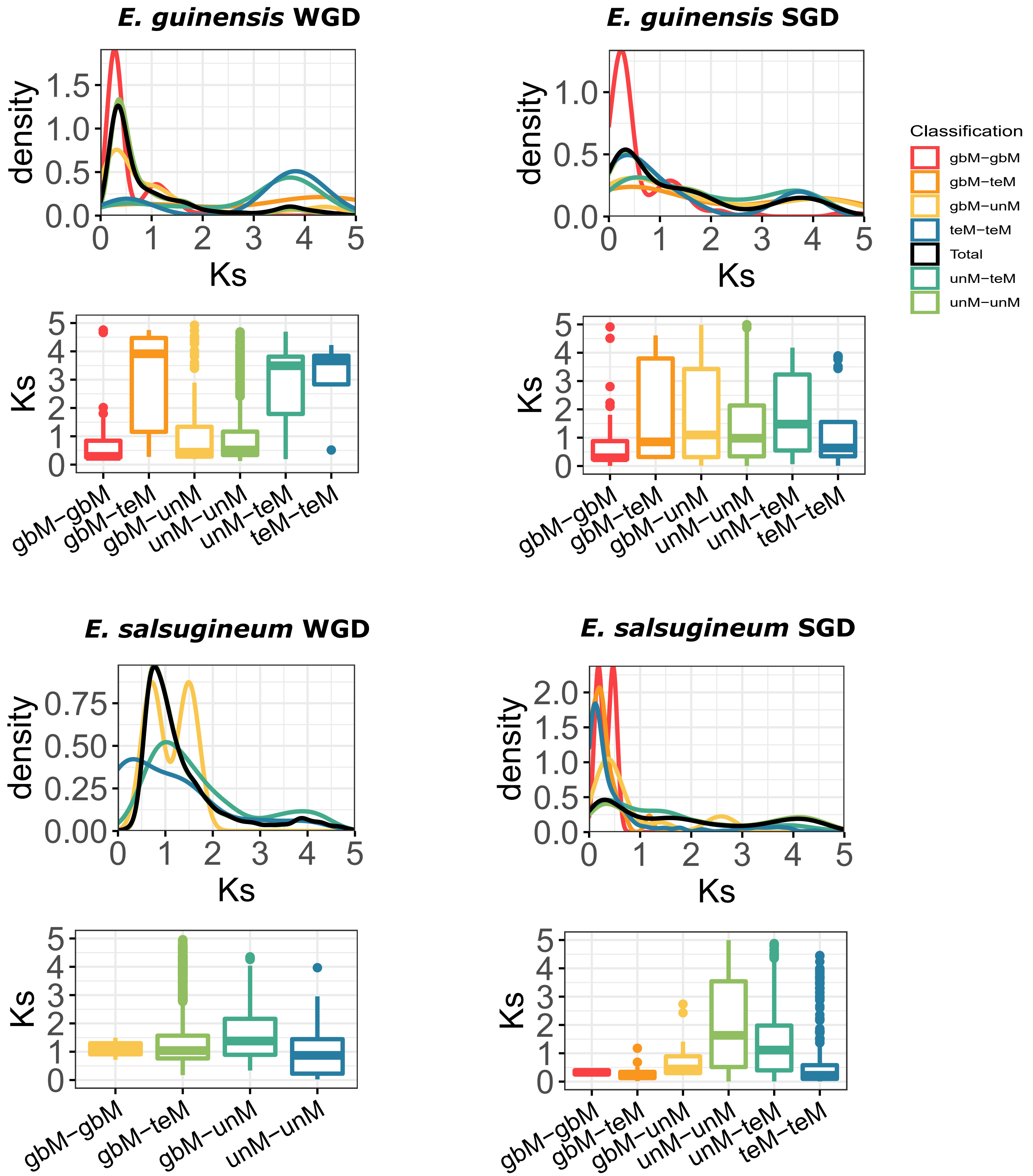

Supplemental Figure S7: **Distribution of genic methylation classified genes based on synonymous substitution (Ks) across different types of gene duplicate pairs.** Whole-genome duplicates - WGD, Single-gene duplicates - SGD (combined data from tandem, proximal, translocated, and dispersed duplicates). Center line in the boxplot represents the median Ks values, while the box limits represent 25% and 75% percentile of the interquartile range, whiskers represent 1.5 times above or below the interquartile range and dots represents outliers.

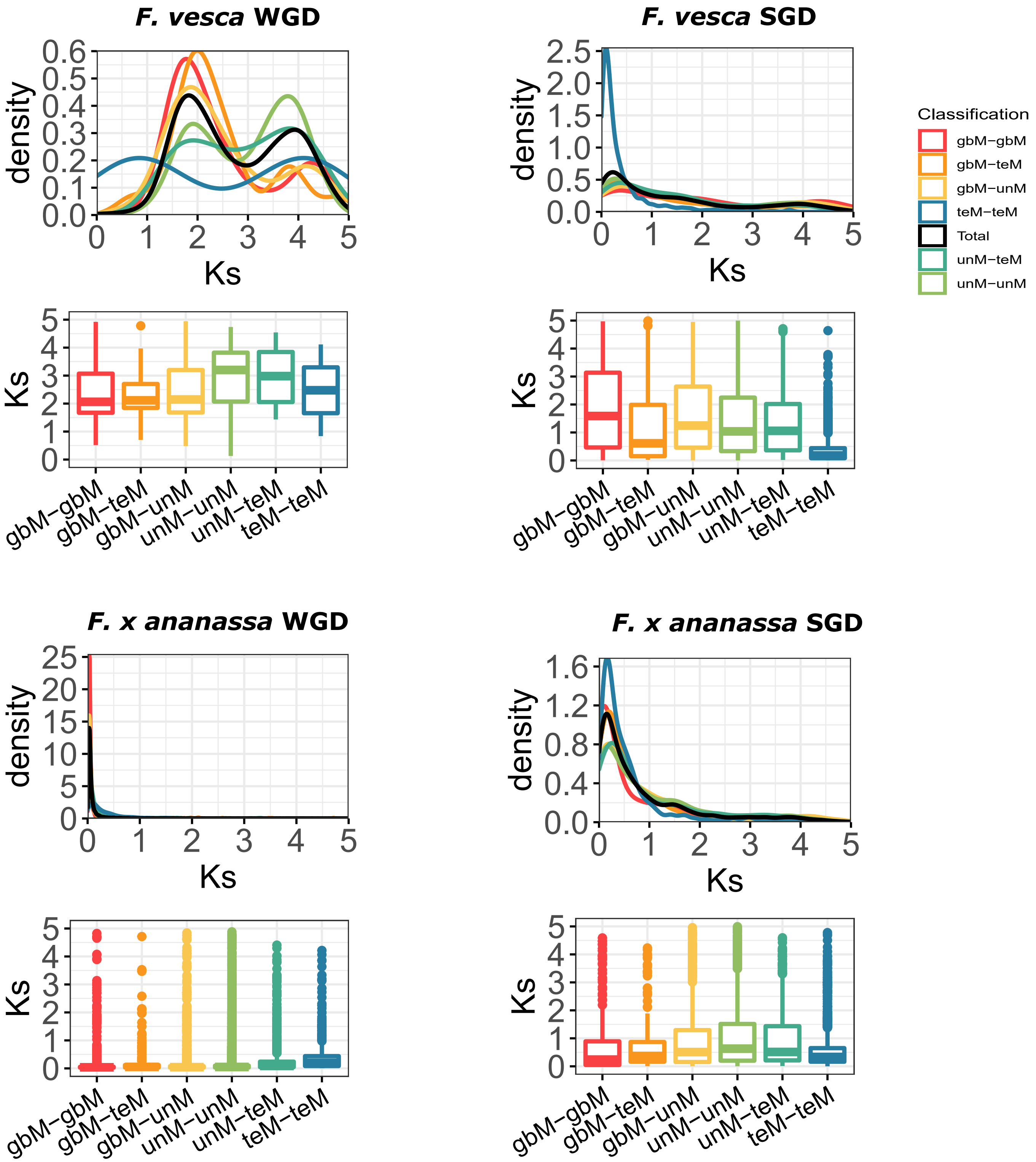

Supplemental Figure S7: **Distribution of genic methylation classified genes based on synonymous substitution (Ks) across different types of gene duplicate pairs.** Whole-genome duplicates - WGD, Single-gene duplicates - SGD (combined data from tandem, proximal, translocated, and dispersed duplicates). Center line in the boxplot represents the median Ks values, while the box limits represent 25% and 75% percentile of the interquartile range, whiskers represent 1.5 times above or below the interquartile range and dots represents outliers.

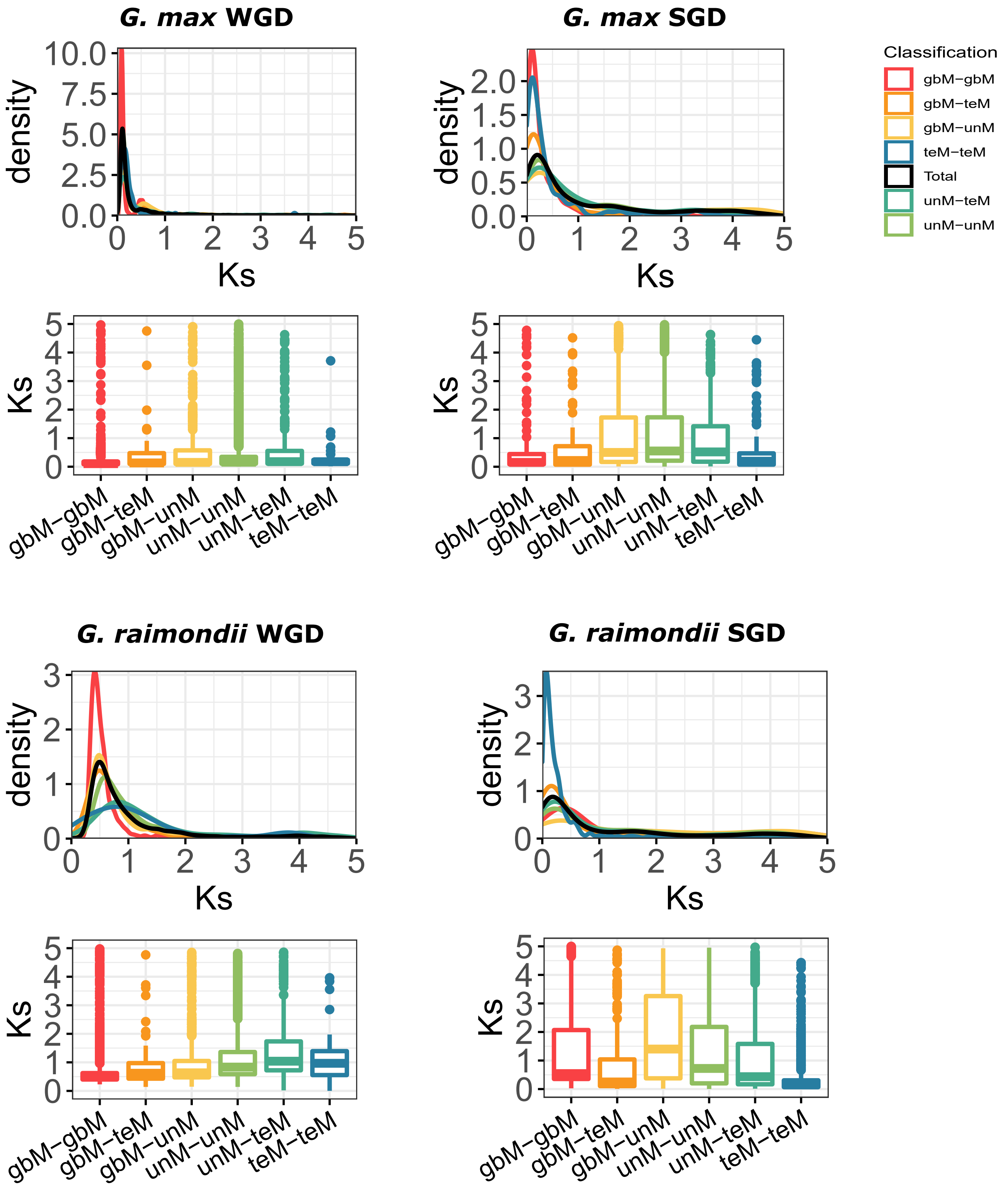

Supplemental Figure S7: **Distribution of genic methylation classified genes based on synonymous substitution (Ks) across different types of gene duplicate pairs.** Whole-genome duplicates - WGD, Single-gene duplicates - SGD (combined data from tandem, proximal, translocated, and dispersed duplicates). Center line in the boxplot represents the median Ks values, while the box limits represent 25% and 75% percentile of the interquartile range, whiskers represent 1.5 times above or below the interquartile range and dots represents outliers.

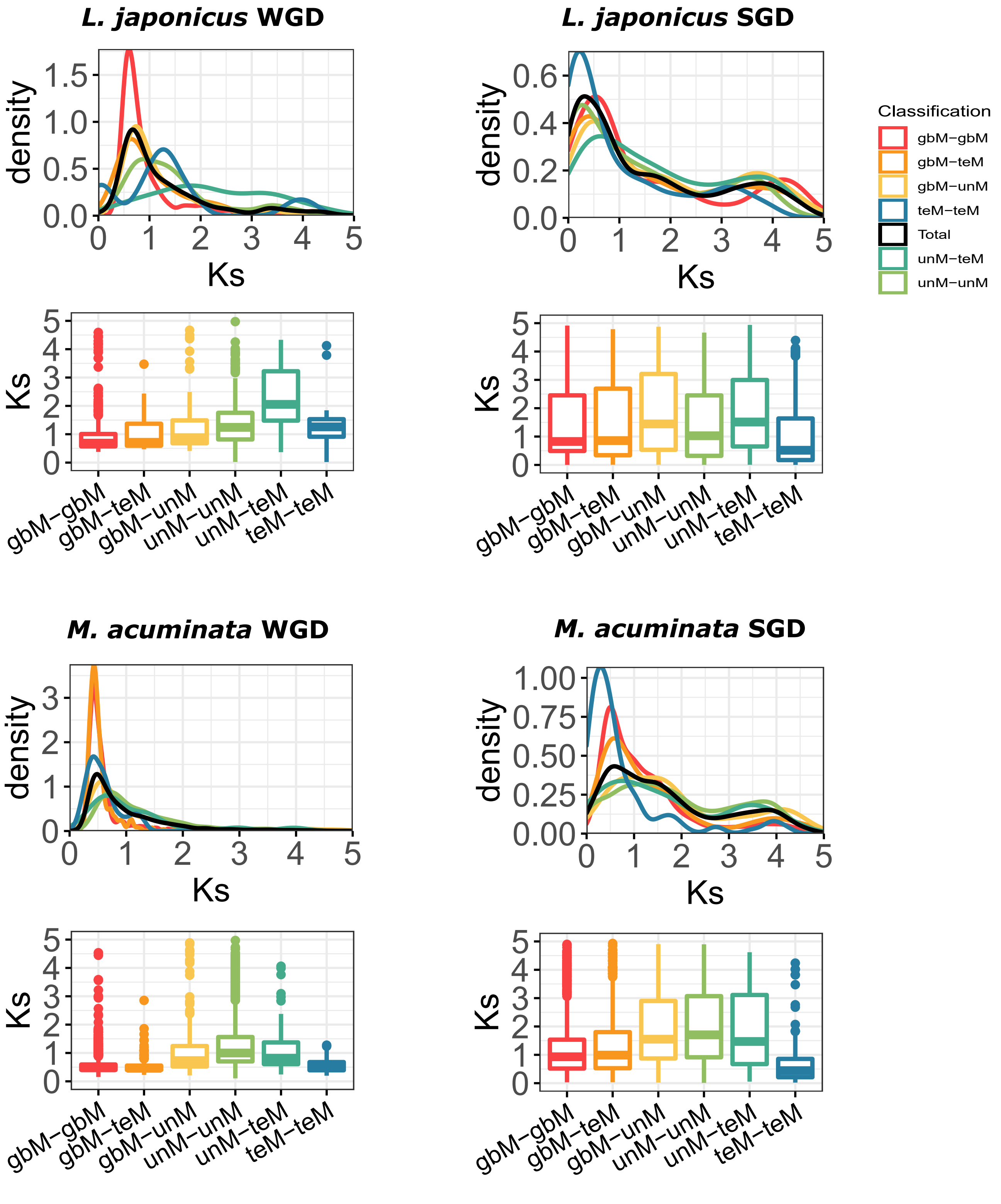

Supplemental Figure S7: **Distribution of genic methylation classified genes based on synonymous substitution (Ks) across different types of gene duplicate pairs.** Whole-genome duplicates - WGD, Single-gene duplicates - SGD (combined data from tandem, proximal, translocated, and dispersed duplicates). Center line in the boxplot represents the median Ks values, while the box limits represent 25% and 75% percentile of the interquartile range, whiskers represent 1.5 times above or below the interquartile range and dots represents outliers.

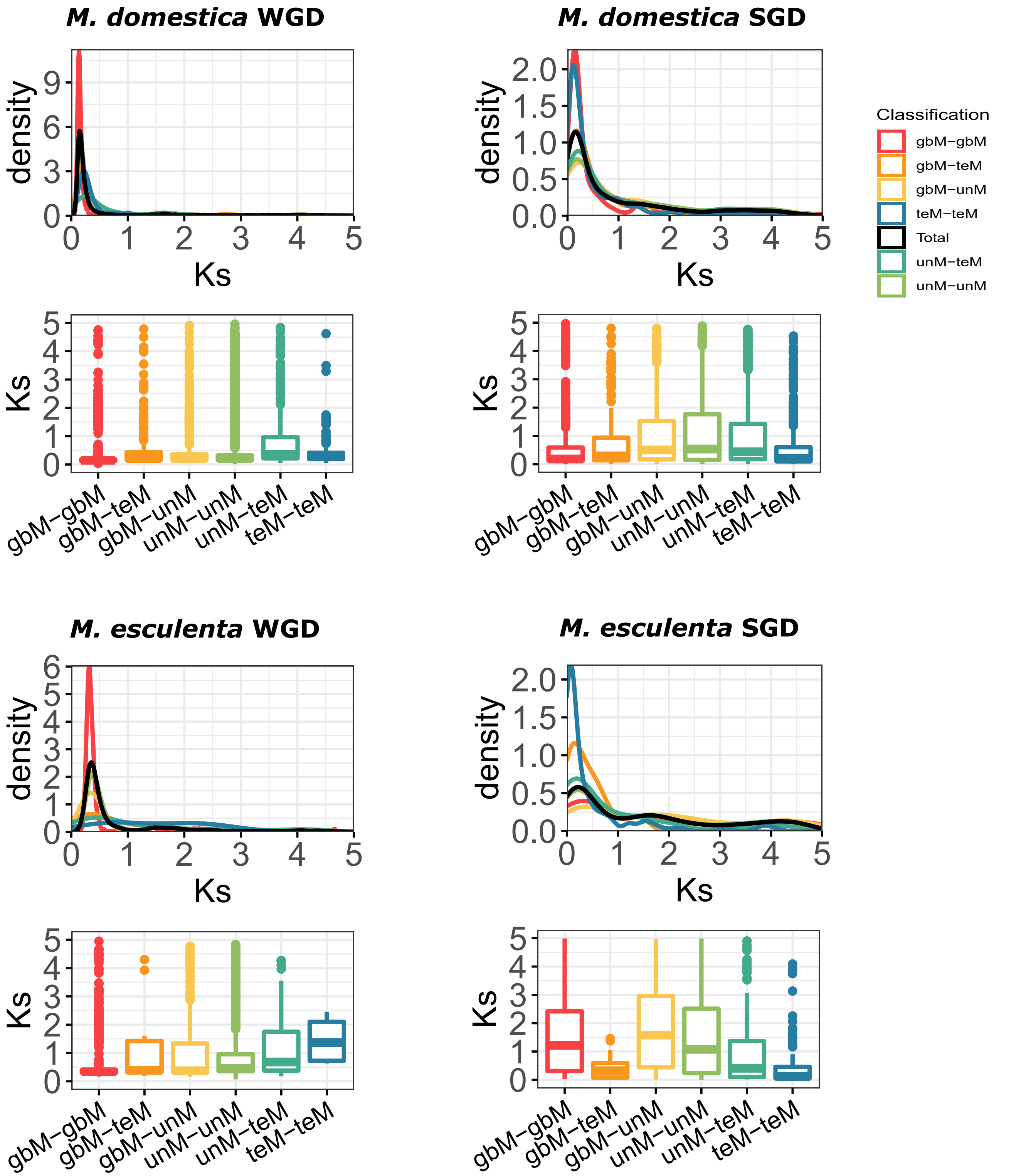

Supplemental Figure S7: **Distribution of genic methylation classified genes based on synonymous substitution (Ks) across different types of gene duplicate pairs.** Whole-genome duplicates - WGD, Single-gene duplicates - SGD (combined data from tandem, proximal, translocated, and dispersed duplicates). Center line in the boxplot represents the median Ks values, while the box limits represent 25% and 75% percentile of the interquartile range, whiskers represent 1.5 times above or below the interquartile range and dots represents outliers.

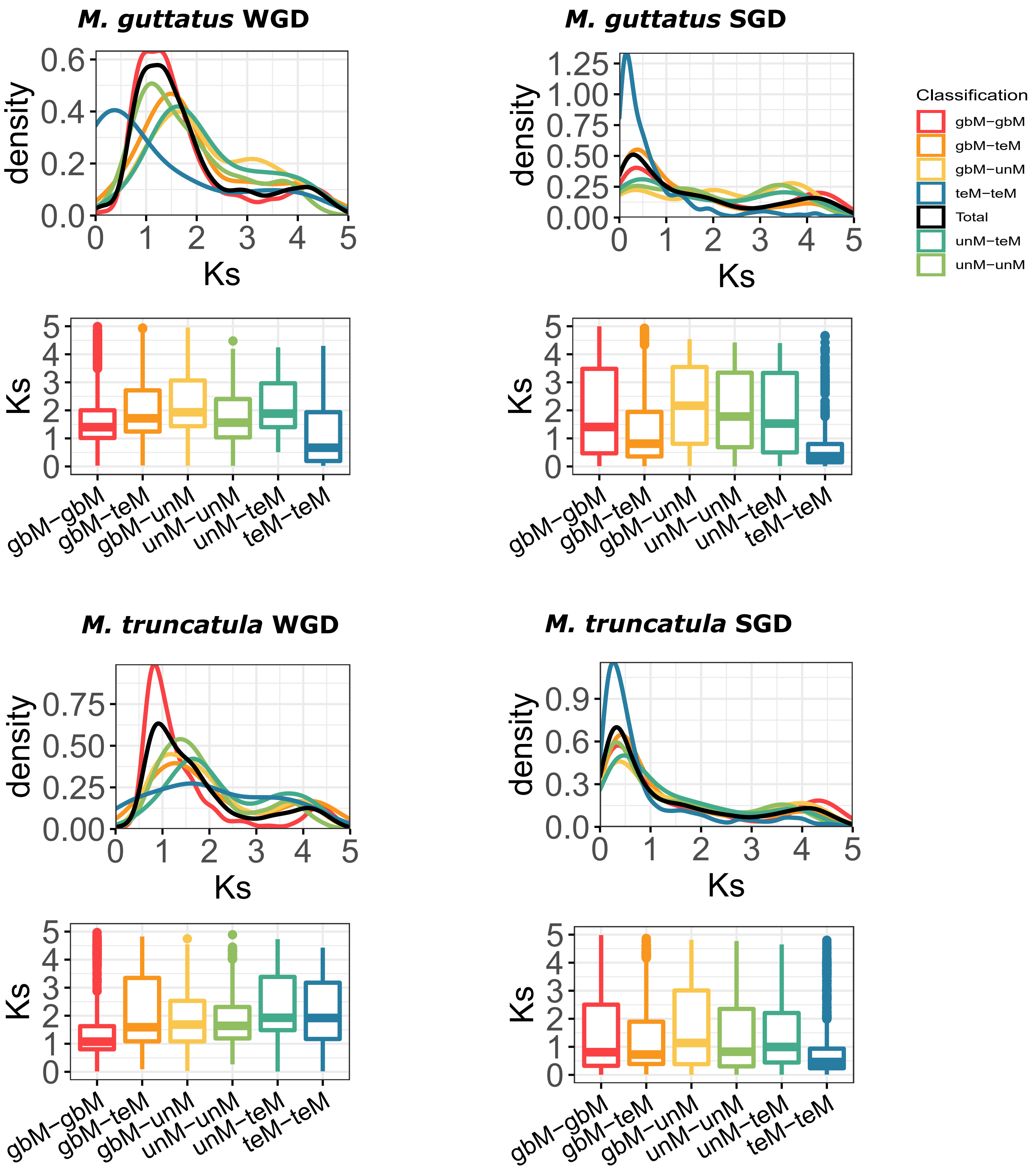

Supplemental Figure S7: **Distribution of genic methylation classified genes based on synonymous substitution (Ks) across different types of gene duplicate pairs.** Whole-genome duplicates - WGD, Single-gene duplicates - SGD (combined data from tandem, proximal, translocated, and dispersed duplicates). Center line in the boxplot represents the median Ks values, while the box limits represent 25% and 75% percentile of the interquartile range, whiskers represent 1.5 times above or below the interquartile range and dots represents outliers.

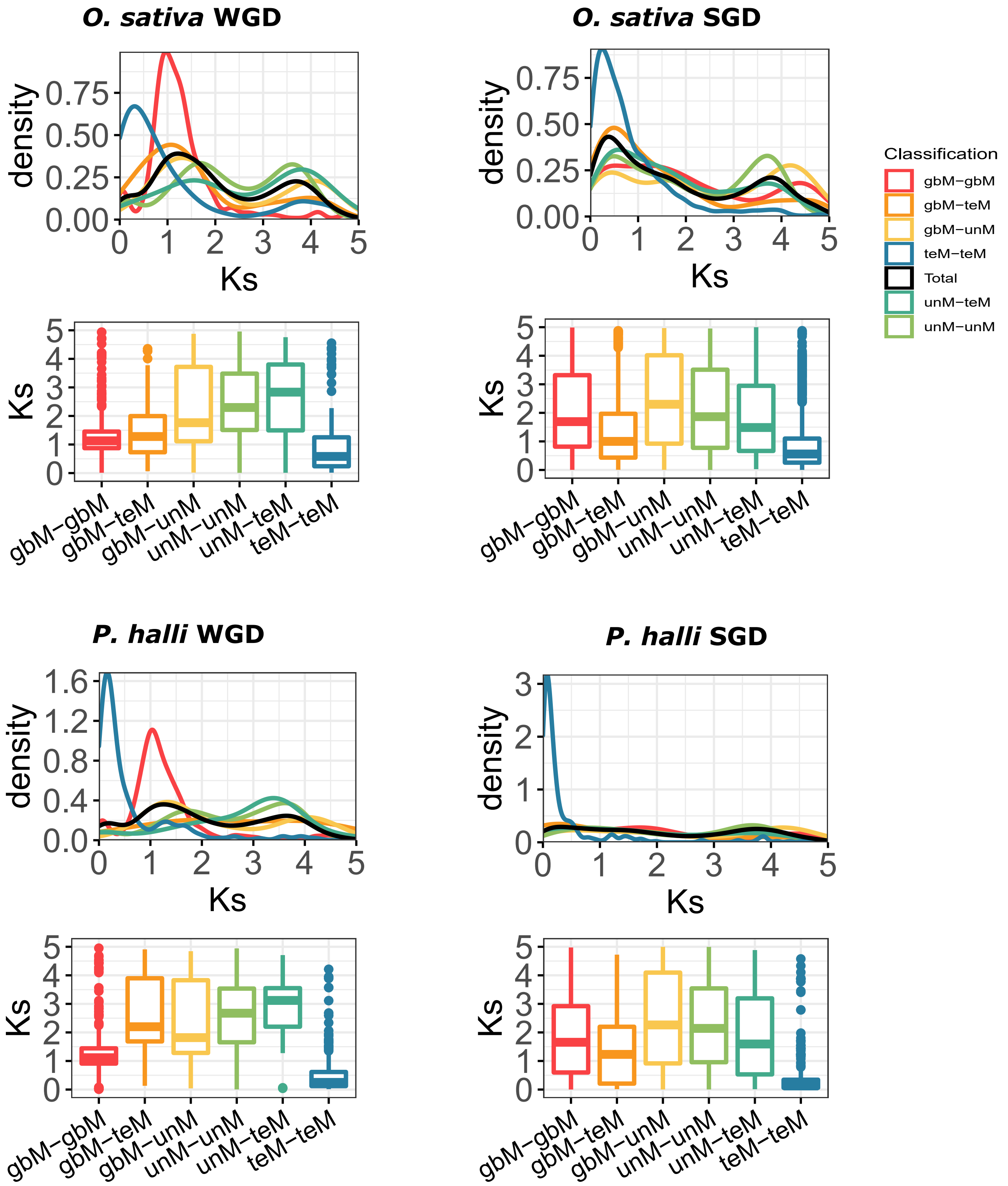

Supplemental Figure S7: **Distribution of genic methylation classified genes based on synonymous substitution (Ks) across different types of gene duplicate pairs.** Whole-genome duplicates - WGD, Single-gene duplicates - SGD (combined data from tandem, proximal, translocated, and dispersed duplicates). Center line in the boxplot represents the median Ks values, while the box limits represent 25% and 75% percentile of the interquartile range, whiskers represent 1.5 times above or below the interquartile range and dots represents outliers.

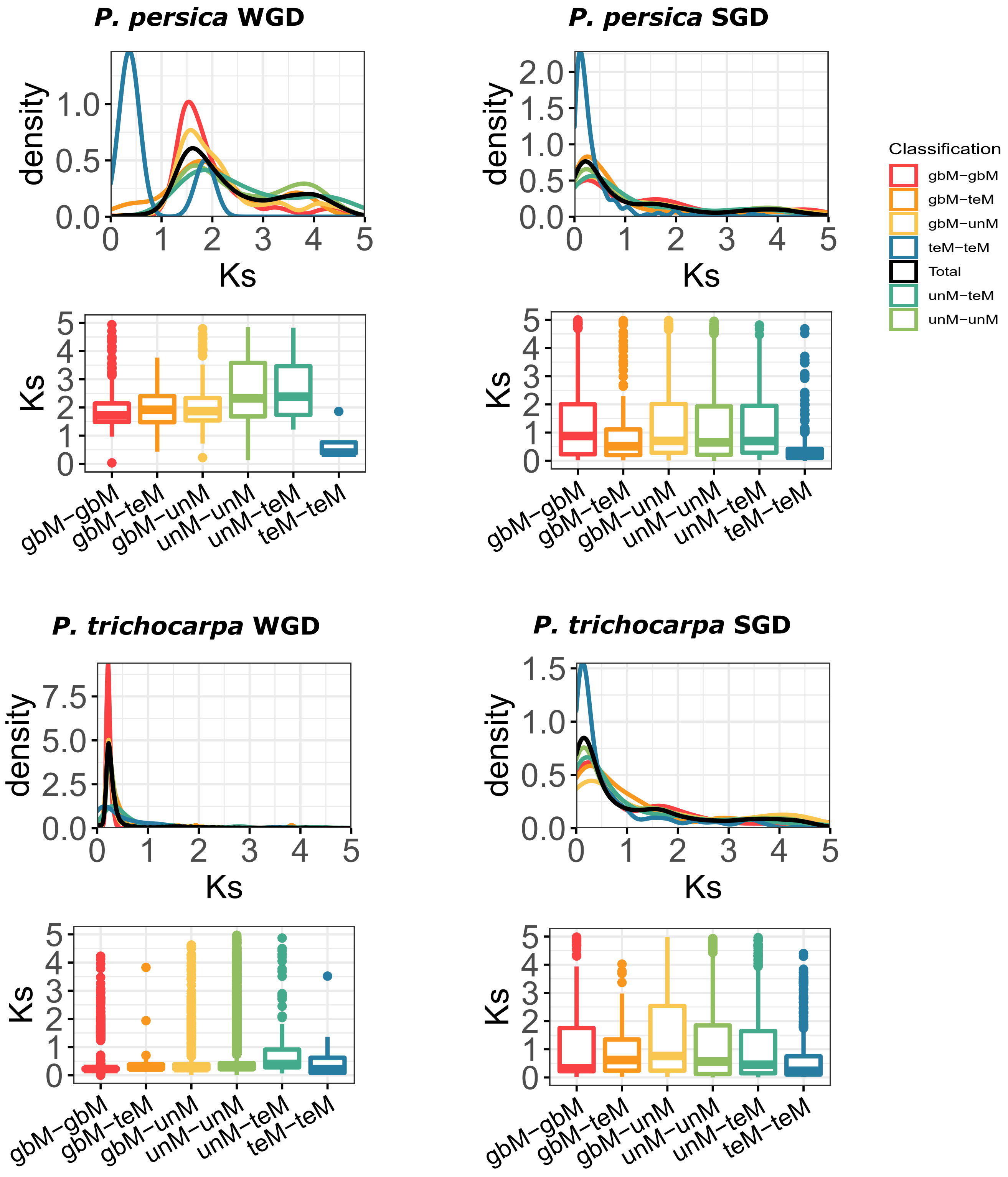

Supplemental Figure S7: **Distribution of genic methylation classified genes based on synonymous substitution (Ks) across different types of gene duplicate pairs.** Whole-genome duplicates - WGD, Single-gene duplicates - SGD (combined data from tandem, proximal, translocated, and dispersed duplicates). Center line in the boxplot represents the median Ks values, while the box limits represent 25% and 75% percentile of the interquartile range, whiskers represent 1.5 times above or below the interquartile range and dots represents outliers.

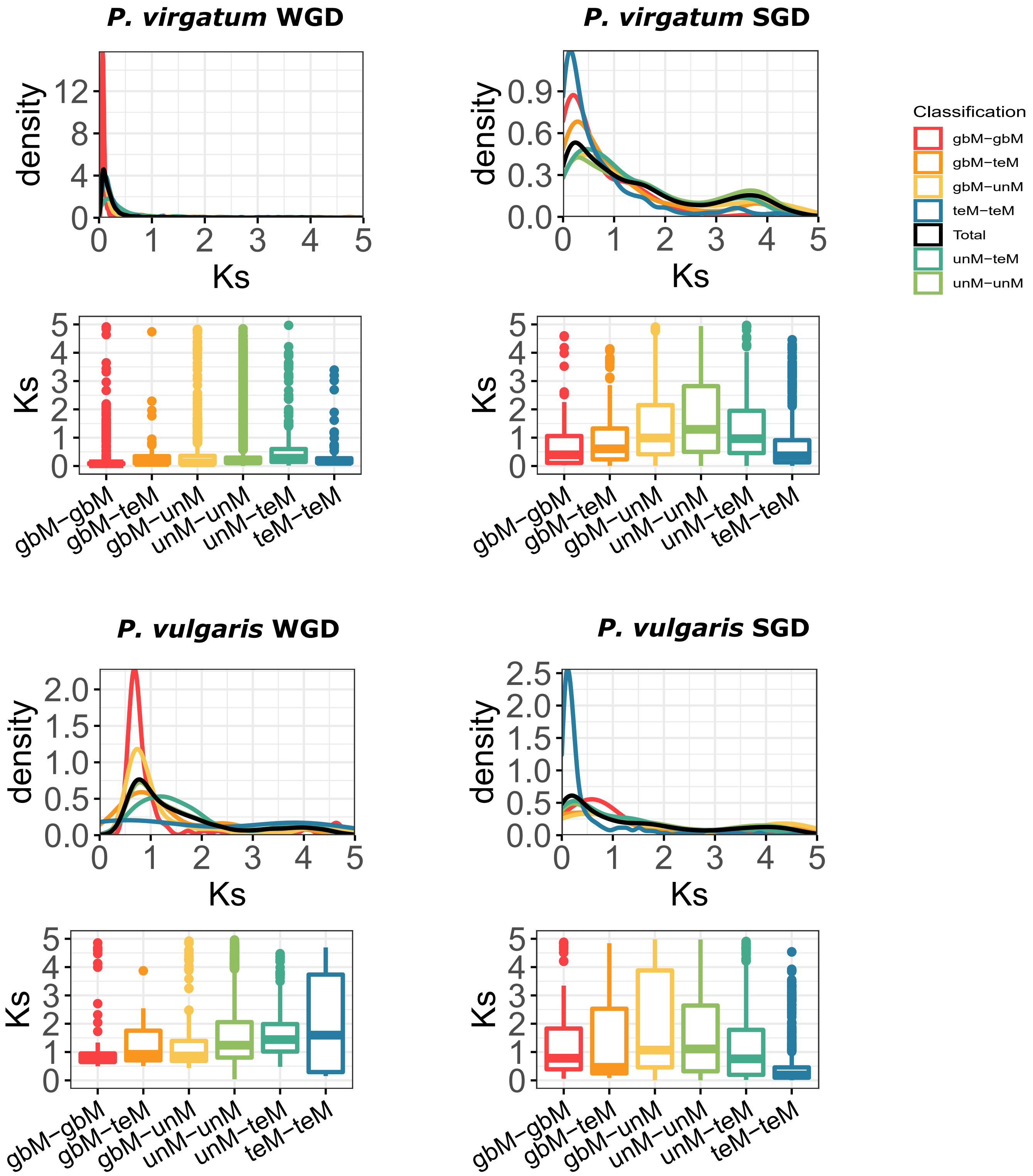

Supplemental Figure S7: **Distribution of genic methylation classified genes based on synonymous substitution (Ks) across different types of gene duplicate pairs.** Whole-genome duplicates - WGD, Single-gene duplicates - SGD (combined data from tandem, proximal, translocated, and dispersed duplicates). Center line in the boxplot represents the median Ks values, while the box limits represent 25% and 75% percentile of the interquartile range, whiskers represent 1.5 times above or below the interquartile range and dots represents outliers.

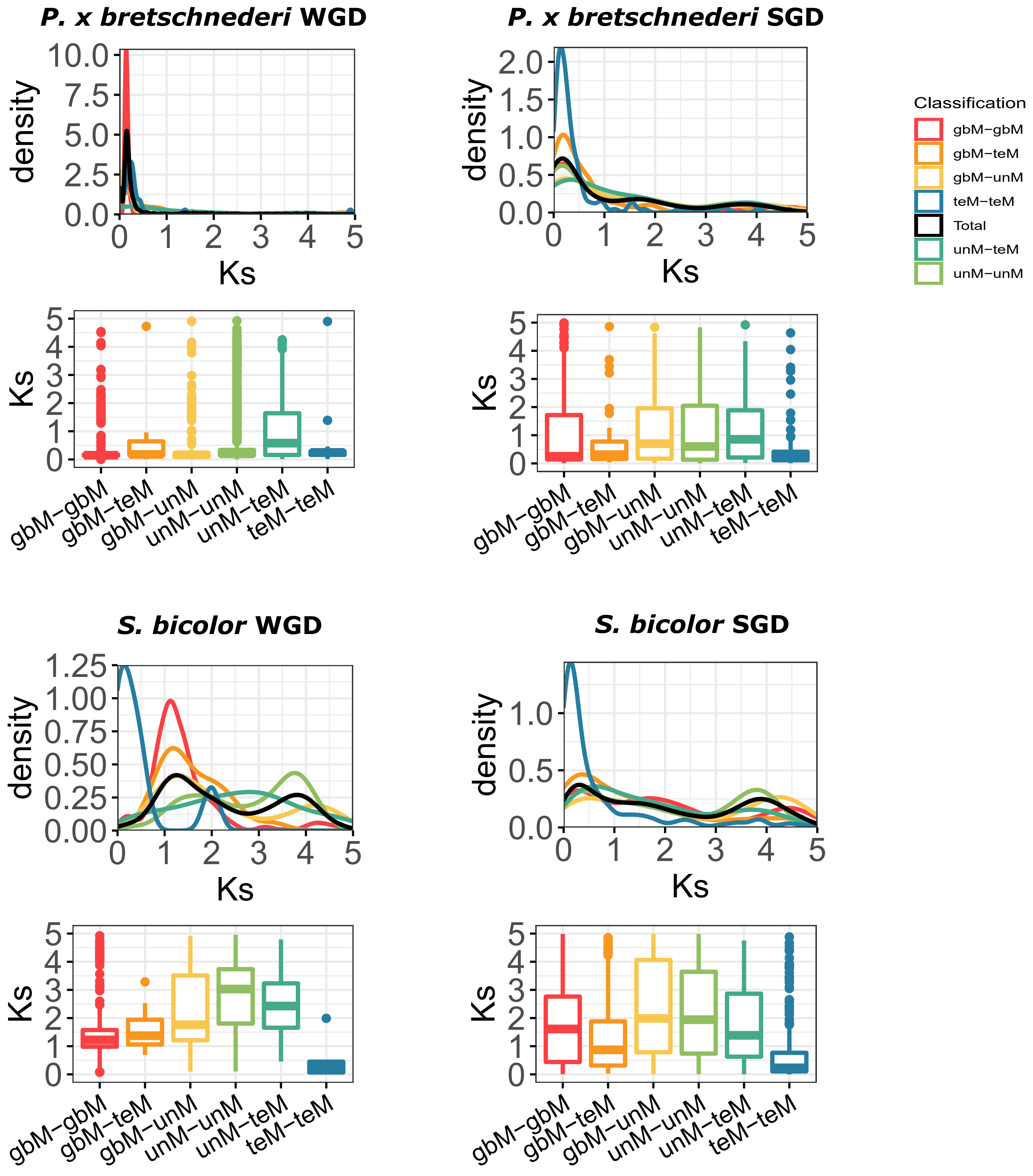

Supplemental Figure S7: **Distribution of genic methylation classified genes based on synonymous substitution (Ks) across different types of gene duplicate pairs.** Whole-genome duplicates - WGD, Single-gene duplicates - SGD (combined data from tandem, proximal, translocated, and dispersed duplicates). Center line in the boxplot represents the median Ks values, while the box limits represent 25% and 75% percentile of the interquartile range, whiskers represent 1.5 times above or below the interquartile range and dots represents outliers.

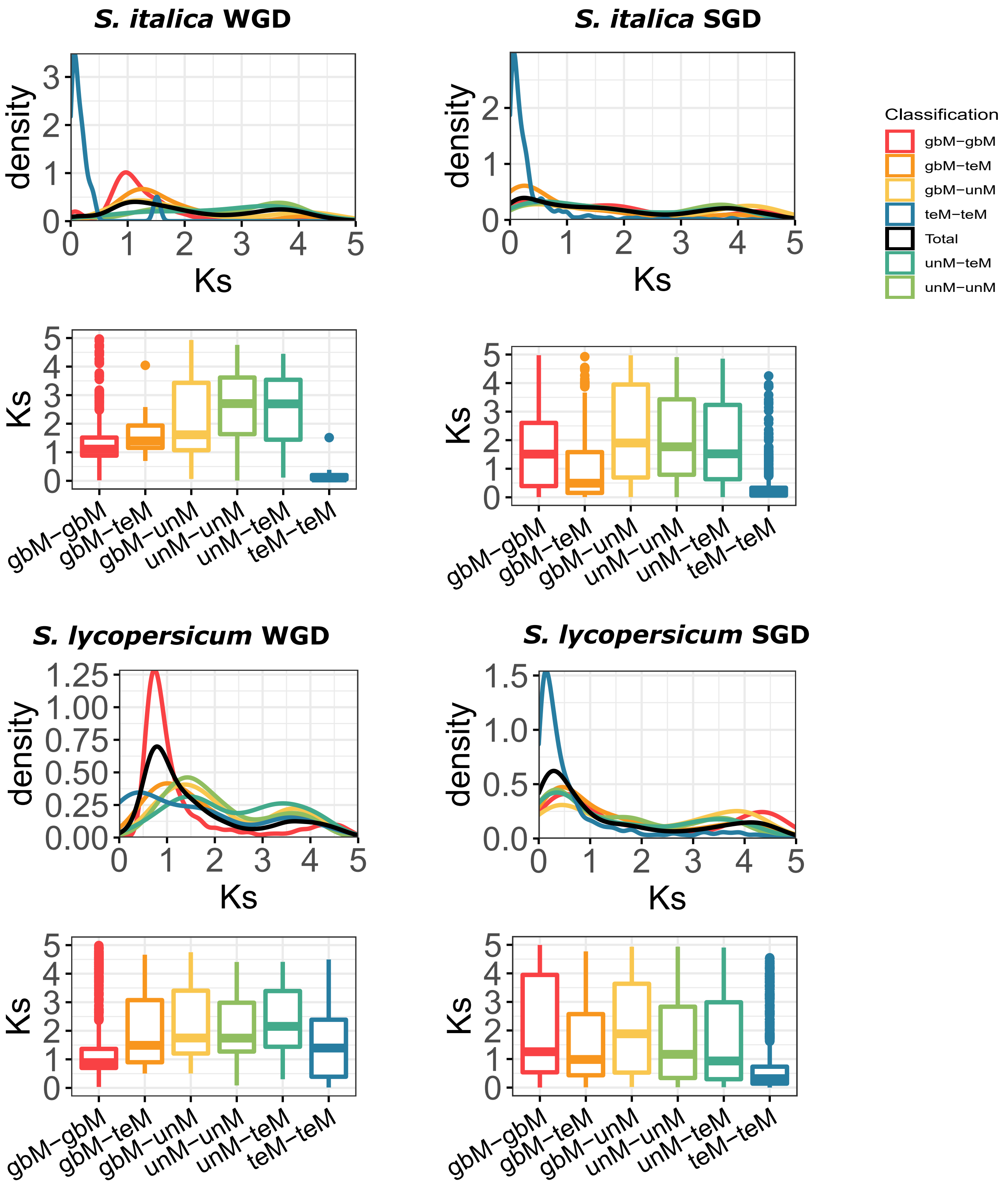

Supplemental Figure S7: **Distribution of genic methylation classified genes based on synonymous substitution (Ks) across different types of gene duplicate pairs.** Whole-genome duplicates - WGD, Single-gene duplicates - SGD (combined data from tandem, proximal, translocated, and dispersed duplicates). Center line in the boxplot represents the median Ks values, while the box limits represent 25% and 75% percentile of the interquartile range, whiskers represent 1.5 times above or below the interquartile range and dots represents outliers.

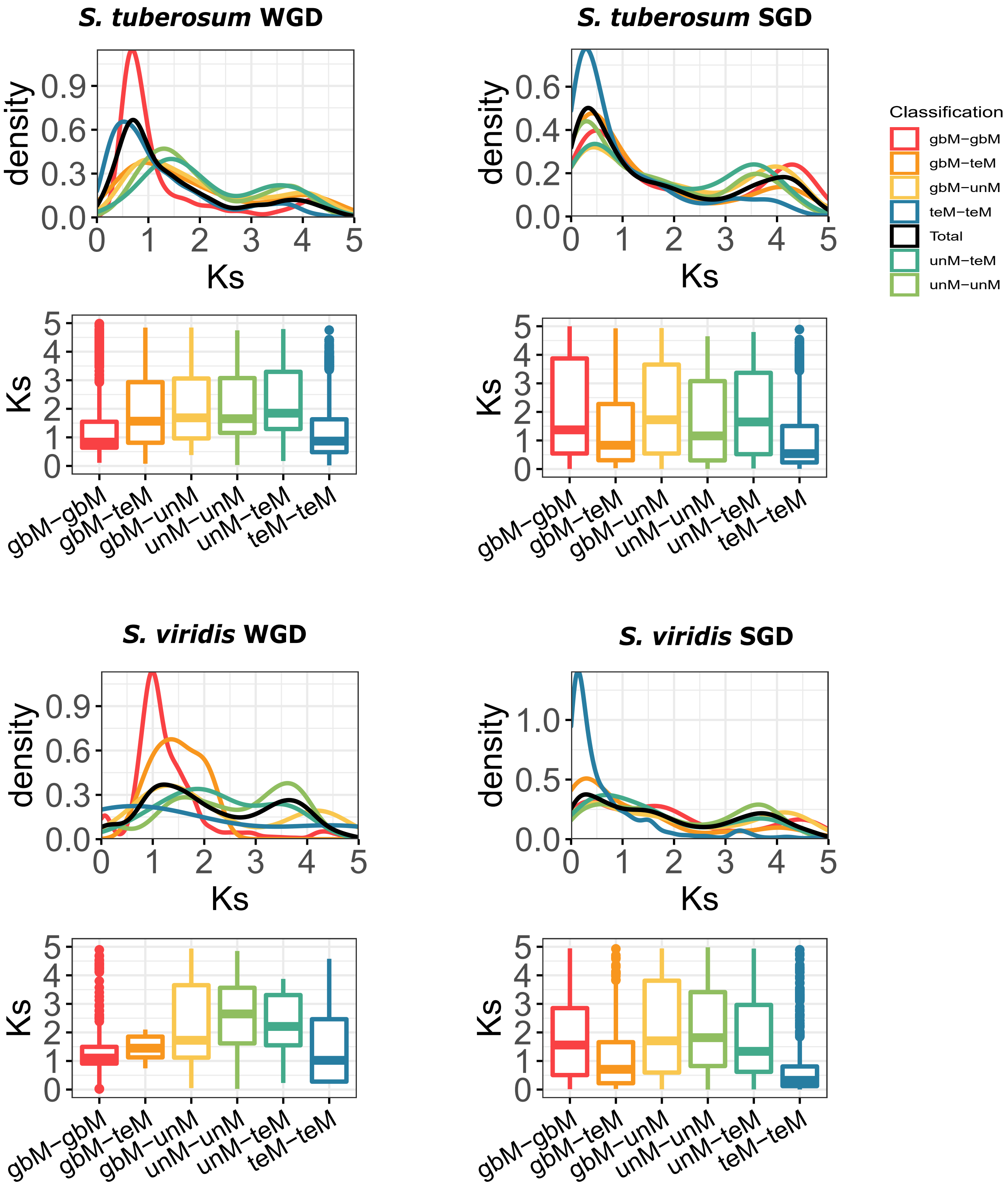

Supplemental Figure S7: **Distribution of genic methylation classified genes based on synonymous substitution (Ks) across different types of gene duplicate pairs.** Whole-genome duplicates - WGD, Single-gene duplicates - SGD (combined data from tandem, proximal, translocated, and dispersed duplicates). Center line in the boxplot represents the median Ks values, while the box limits represent 25% and 75% percentile of the interquartile range, whiskers represent 1.5 times above or below the interquartile range and dots represents outliers.

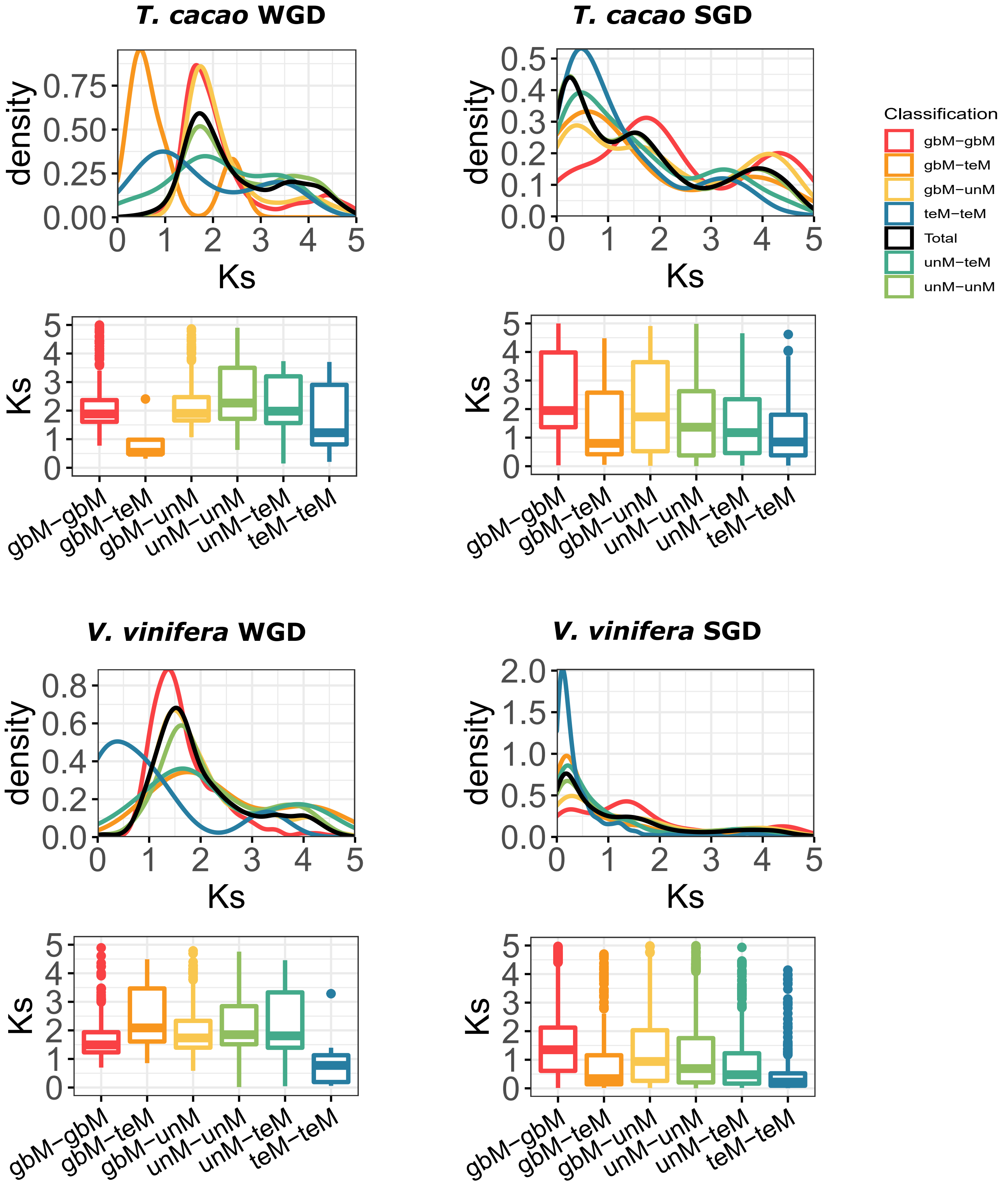

Supplemental Figure S7: **Distribution of genic methylation classified genes based on synonymous substitution (Ks) across different types of gene duplicate pairs.** Whole-genome duplicates - WGD, Single-gene duplicates - SGD (combined data from tandem, proximal, translocated, and dispersed duplicates). Center line in the boxplot represents the median Ks values, while the box limits represent 25% and 75% percentile of the interquartile range, whiskers represent 1.5 times above or below the interquartile range and dots represents outliers.

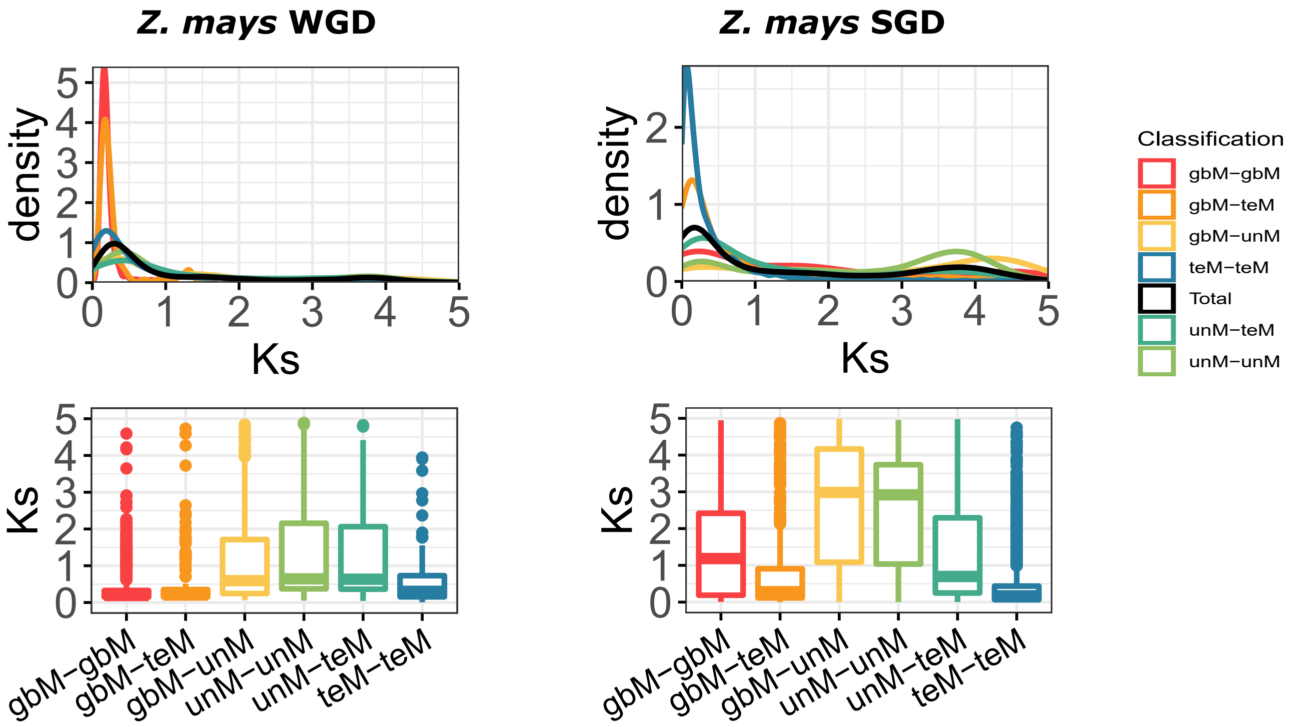

Supplemental Figure S8: Proportion of different genic methylation in transposed duplicates across different epochs. For example, in *A. duranensis* translocated genes that have duplicated since *A. duranensis* diverged from *A. ipaensis* are shown on the x-axis under *A. ipaensis*. Those shown under *G. max*, duplicated in the period since the common ancestor of *A. duranensis* and *A. ipaensis* diverged from their common ancestor with *G. max*, but before the divergence of *A. duranensis* and *A. ipaensis.* Horizontal dotted lines indicate the percentage of each genic methylation class in all translocated duplicates. Bars above this line indicate enrichment, below this line depletion.

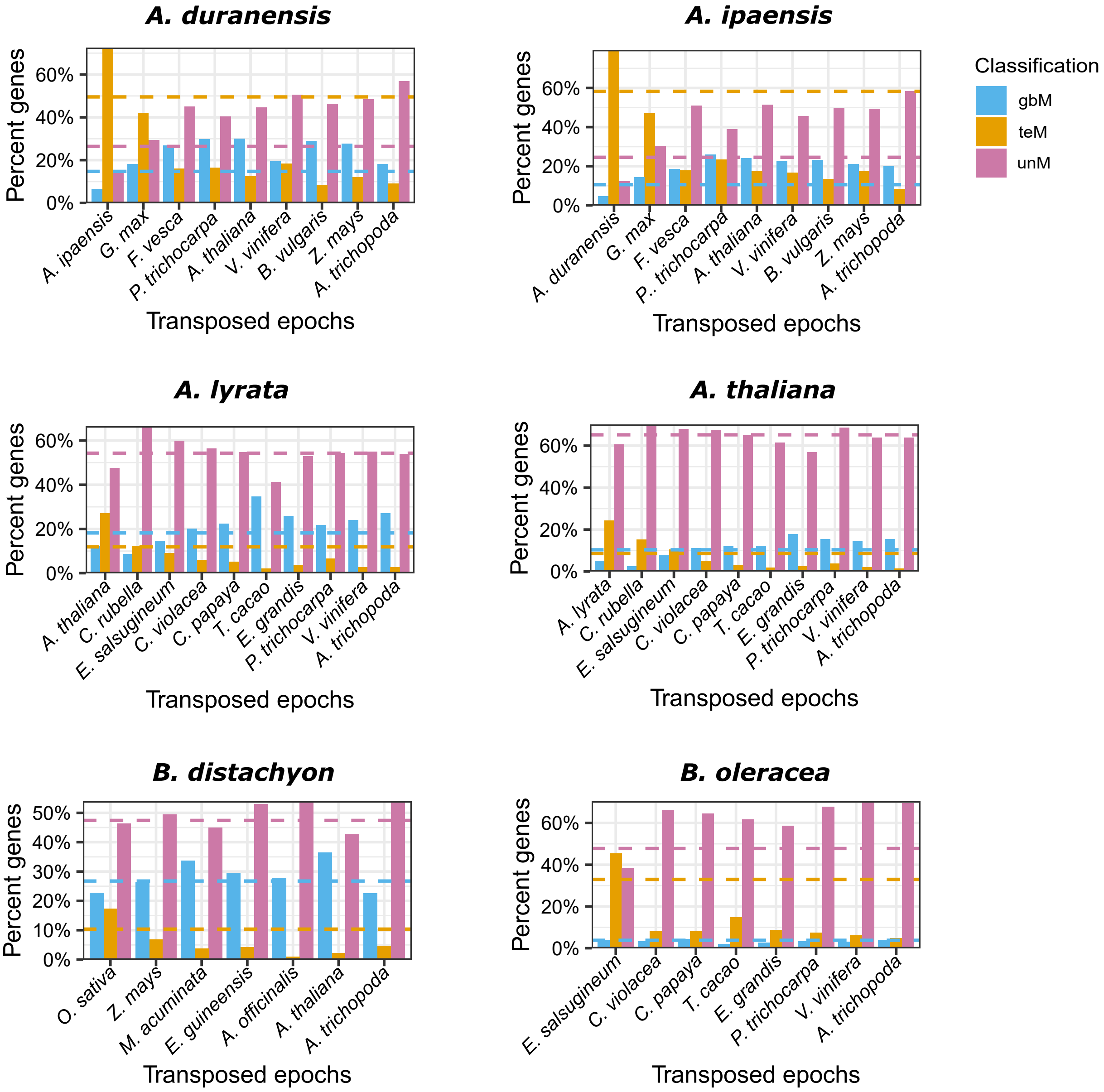

Supplemental Figure S8: Proportion of different genic methylation in transposed duplicates across different epochs. For example, in *A. duranensis* translocated genes that have duplicated since *A. duranensis* diverged from *A. ipaensis* are shown on the x-axis under *A. ipaensis*. Those shown under *G. max*, duplicated in the period since the common ancestor of *A. duranensis* and *A. ipaensis* diverged from their common ancestor with *G. max*, but before the divergence of *A. duranensis* and *A. ipaensis.* Horizontal dotted lines indicate the percentage of each genic methylation class in all translocated duplicates. Bars above this line indicate enrichment, below this line depletion.

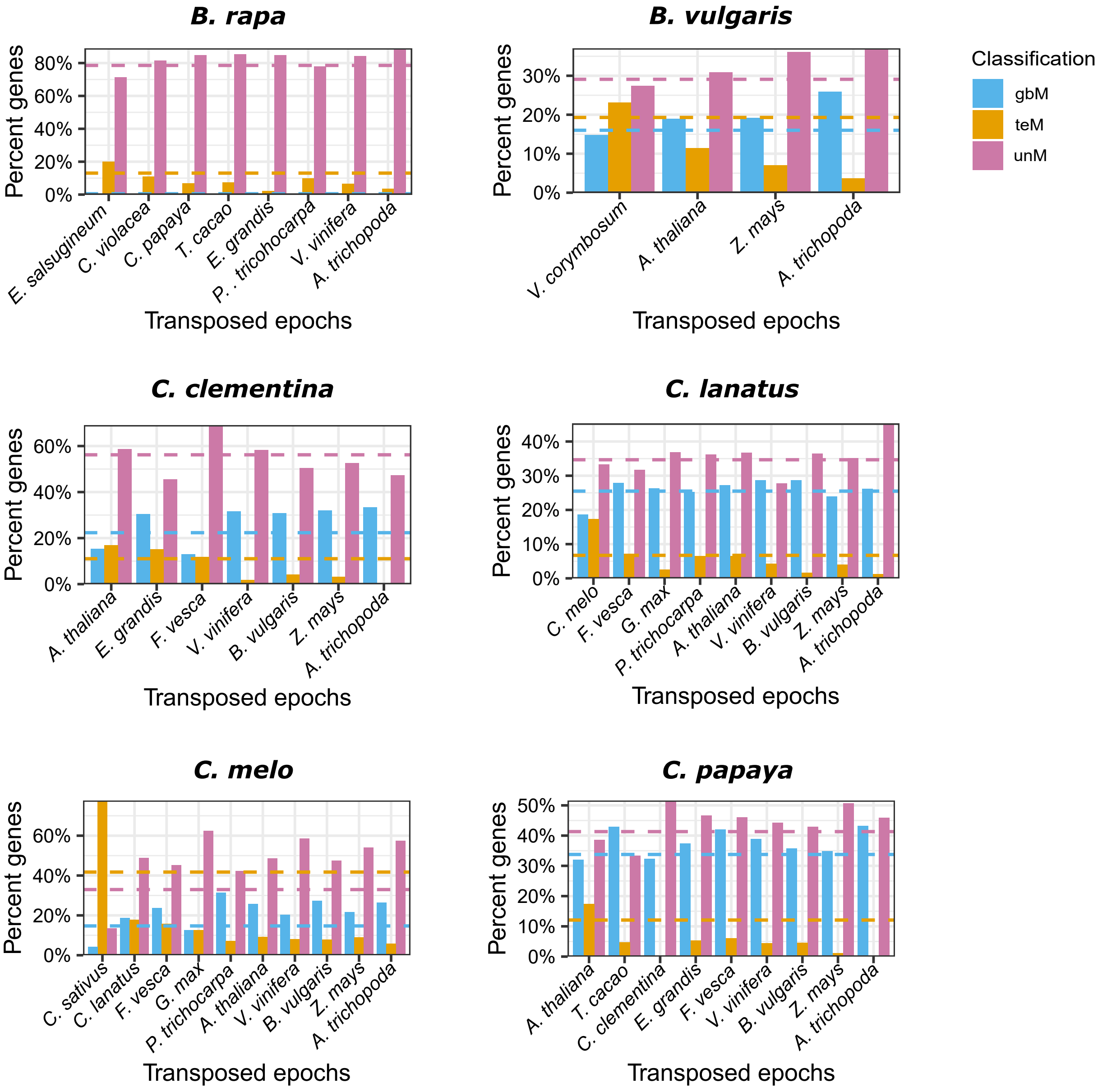

Supplemental Figure S8: Proportion of different genic methylation in transposed duplicates across different epochs. For example, in *A. duranensis* translocated genes that have duplicated since *A. duranensis* diverged from *A. ipaensis* are shown on the x-axis under *A. ipaensis*. Those shown under *G. max*, duplicated in the period since the common ancestor of *A. duranensis* and *A. ipaensis* diverged from their common ancestor with *G. max*, but before the divergence of *A. duranensis* and *A. ipaensis.* Horizontal dotted lines indicate the percentage of each genic methylation class in all translocated duplicates. Bars above this line indicate enrichment, below this line depletion.

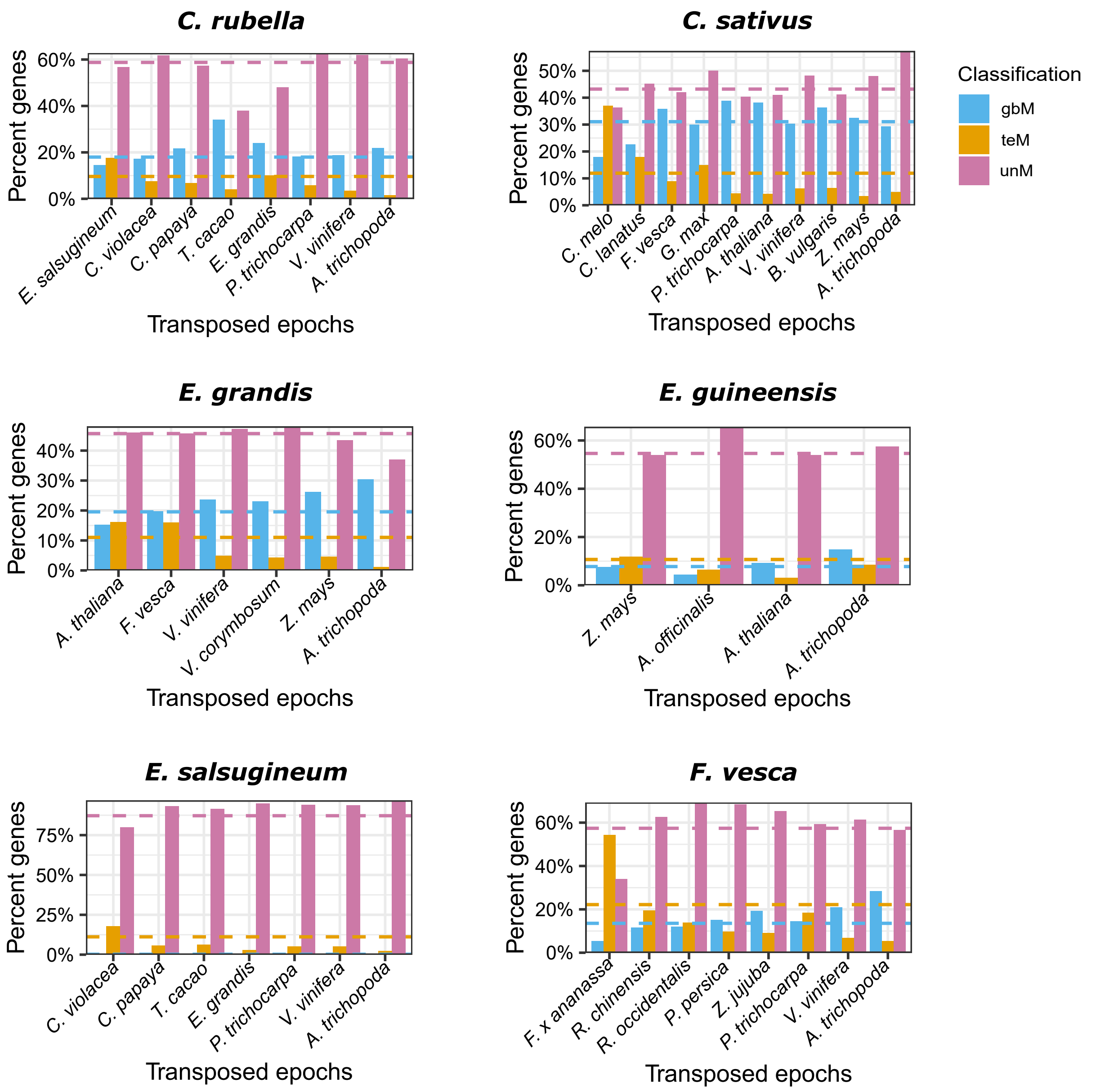

Supplemental Figure S8: Proportion of different genic methylation in transposed duplicates across different epochs. For example, in *A. duranensis* translocated genes that have duplicated since *A. duranensis* diverged from *A. ipaensis* are shown on the x-axis under *A. ipaensis*. Those shown under *G. max*, duplicated in the period since the common ancestor of *A. duranensis* and *A. ipaensis* diverged from their common ancestor with *G. max*, but before the divergence of *A. duranensis* and *A. ipaensis.* Horizontal dotted lines indicate the percentage of each genic methylation class in all translocated duplicates. Bars above this line indicate enrichment, below this line depletion.

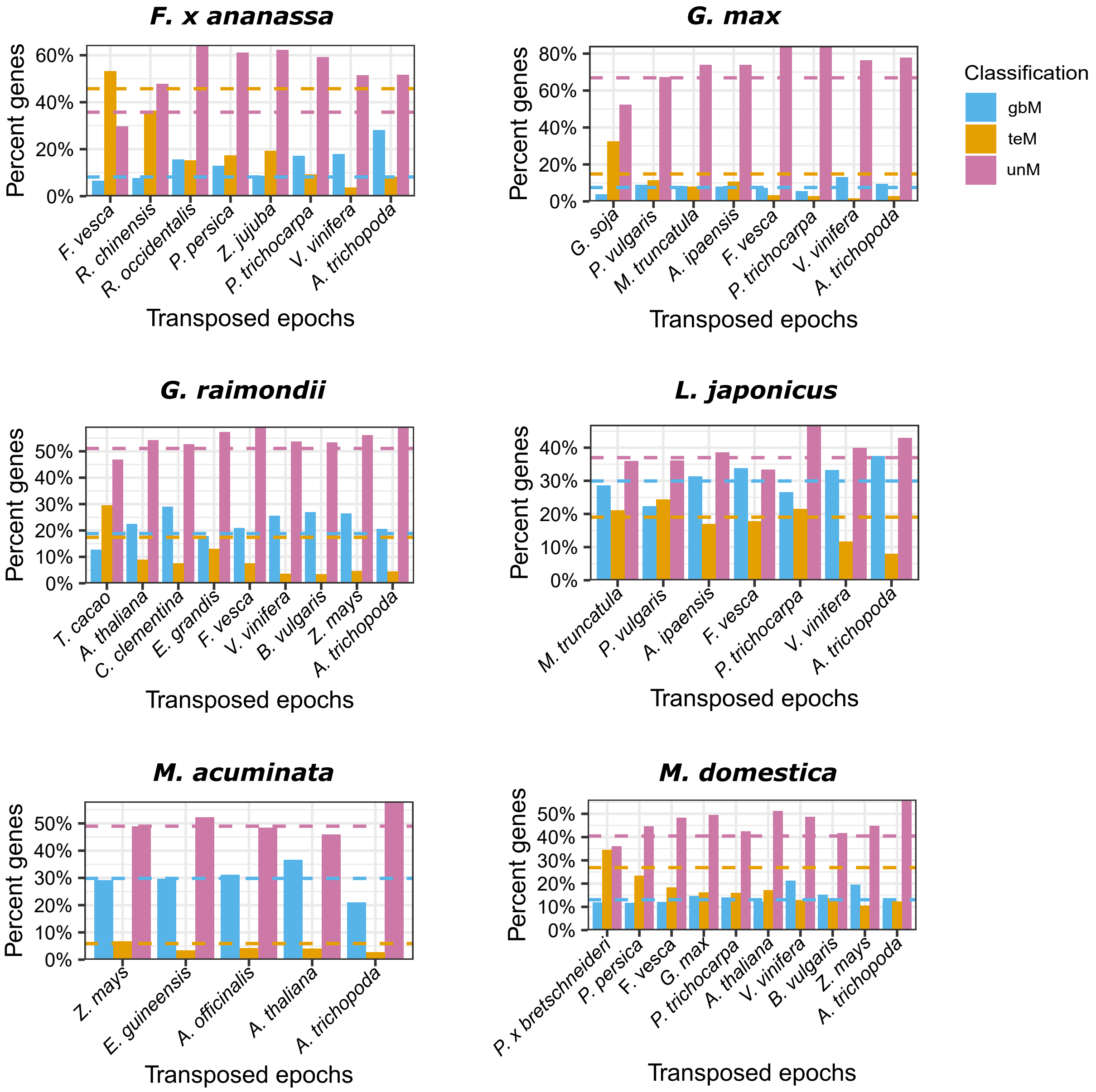

Supplemental Figure S8: Proportion of different genic methylation in transposed duplicates across different epochs. For example, in *A. duranensis* translocated genes that have duplicated since *A. duranensis* diverged from *A. ipaensis* are shown on the x-axis under *A. ipaensis*. Those shown under *G. max*, duplicated in the period since the common ancestor of *A. duranensis* and *A. ipaensis* diverged from their common ancestor with *G. max*, but before the divergence of *A. duranensis* and *A. ipaensis.* Horizontal dotted lines indicate the percentage of each genic methylation class in all translocated duplicates. Bars above this line indicate enrichment, below this line depletion.

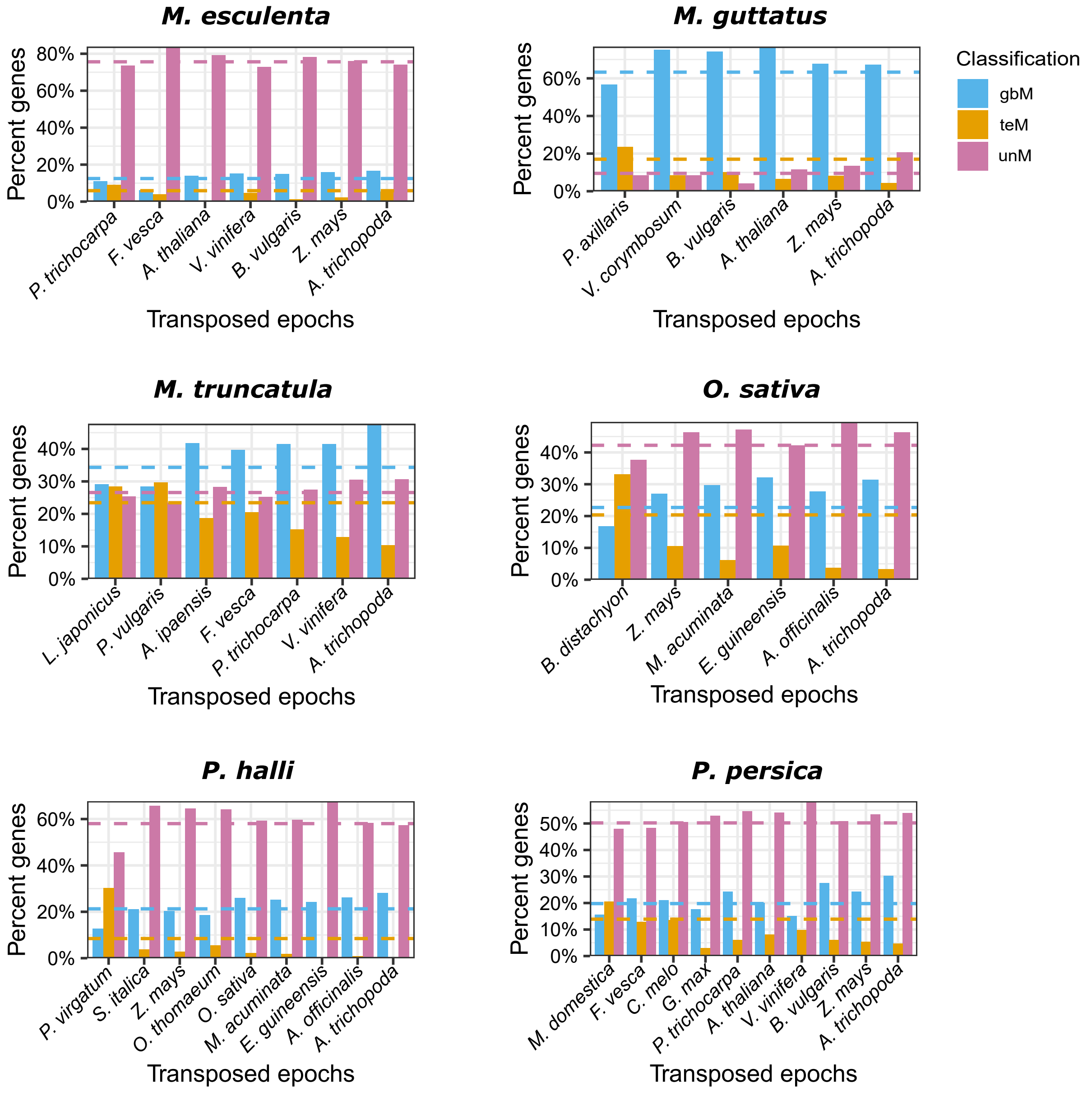

Supplemental Figure S8: Proportion of different genic methylation in transposed duplicates across different epochs. For example, in *A. duranensis* translocated genes that have duplicated since *A. duranensis* diverged from *A. ipaensis* are shown on the x-axis under *A. ipaensis*. Those shown under *G. max*, duplicated in the period since the common ancestor of *A. duranensis* and *A. ipaensis* diverged from their common ancestor with *G. max*, but before the divergence of *A. duranensis* and *A. ipaensis.* Horizontal dotted lines indicate the percentage of each genic methylation class in all translocated duplicates. Bars above this line indicate enrichment, below this line depletion.

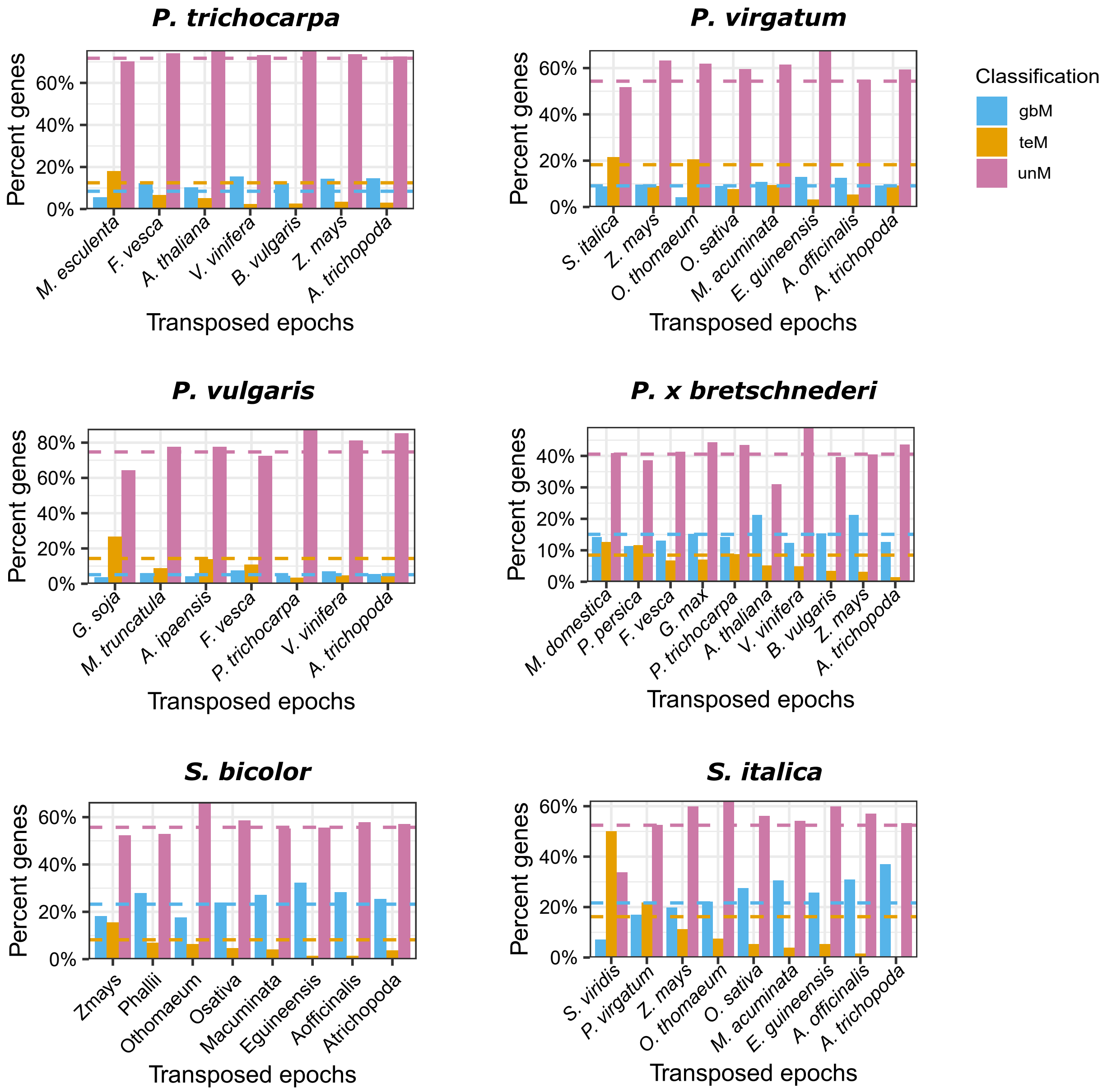

Supplemental Figure S8: Proportion of different genic methylation in transposed duplicates across different epochs. For example, in *A. duranensis* translocated genes that have duplicated since *A. duranensis* diverged from *A. ipaensis* are shown on the x-axis under *A. ipaensis*. Those shown under *G. max*, duplicated in the period since the common ancestor of *A. duranensis* and *A. ipaensis* diverged from their common ancestor with *G. max*, but before the divergence of *A. duranensis* and *A. ipaensis.* Horizontal dotted lines indicate the percentage of each genic methylation class in all translocated duplicates. Bars above this line indicate enrichment, below this line depletion.

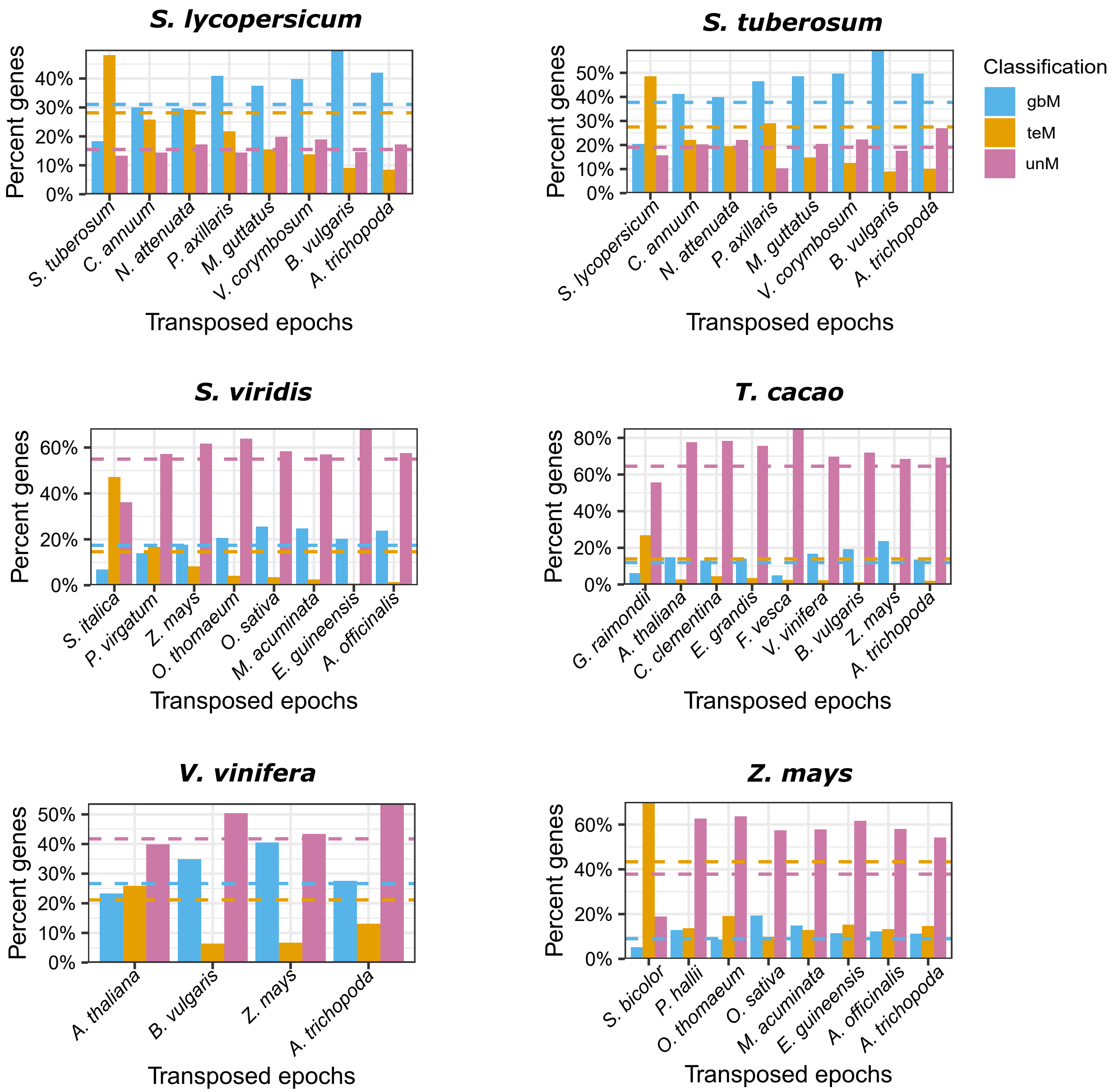

Supplemental Figure S9: **Distribution of core: multi-copy and core: single-copy (intermediate) paralogs based on synonymous substitutions (Ks)**. 'All pairs' represent Ks values of all duplicate gene pairs in the genome, 'Core-MC' represents duplicate pairs among core: multi-copy orthogroup, while 'SC-Int' represent duplicate pairs among the core: single-copy orthogroups. Center line in the boxplot represents the median Ks values, while the box limits represent 25% and 75% percentile of the interquartile range, whiskers represent 1.5 times above or below the interquartile range and dots represents outliers.

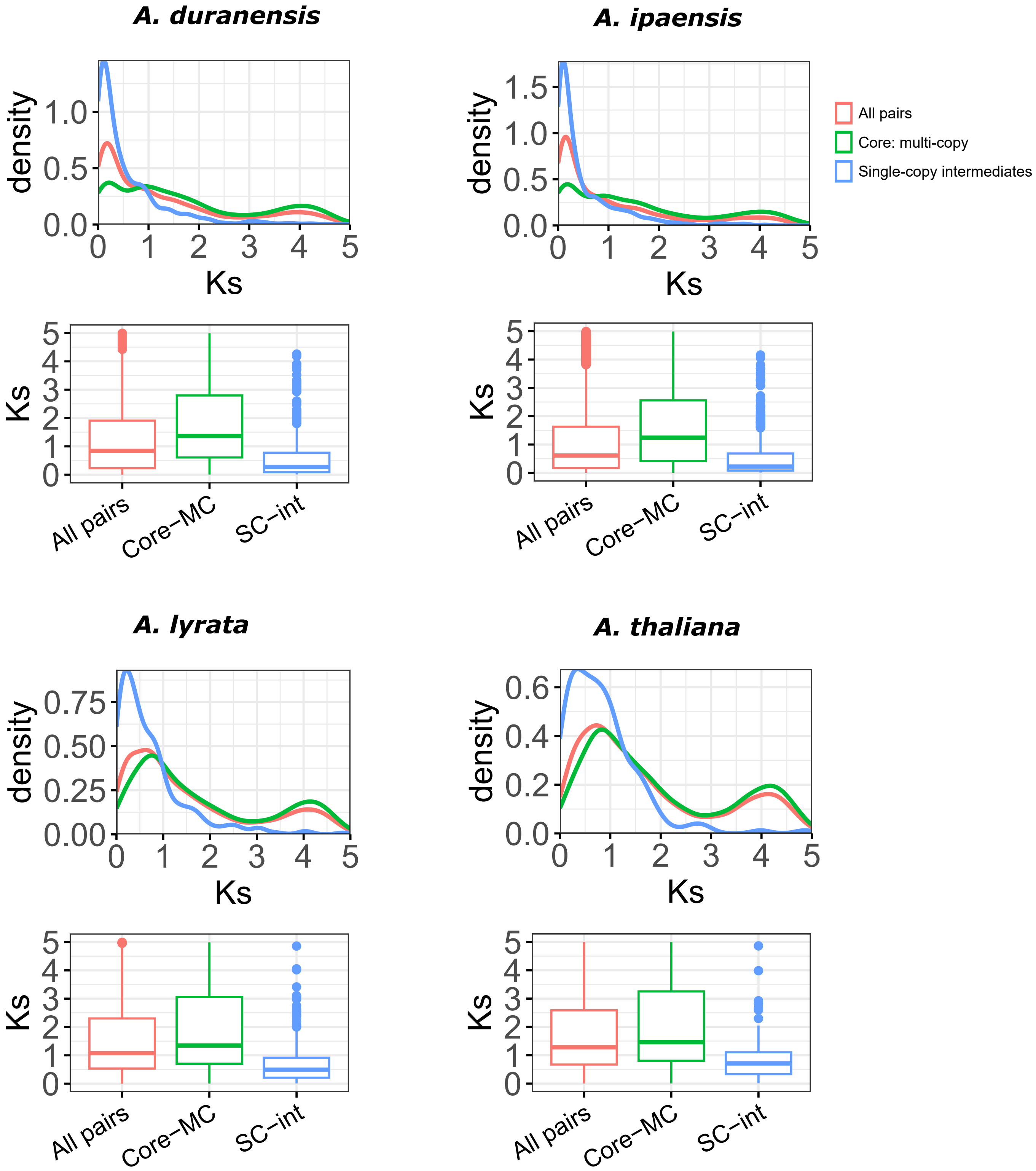

Supplemental Figure S9: **Distribution of core: multi-copy and core: single-copy (intermediate) paralogs based on synonymous substitutions (Ks)**. 'All pairs' represent Ks values of all duplicate gene pairs in the genome, 'Core-MC' represents duplicate pairs among core: multi-copy orthogroup, while 'SC-Int' represent duplicate pairs among the core: single-copy orthogroups. Center line in the boxplot represents the median Ks values, while the box limits represent 25% and 75% percentile of the interquartile range, whiskers represent 1.5 times above or below the interquartile range and dots represents outliers.

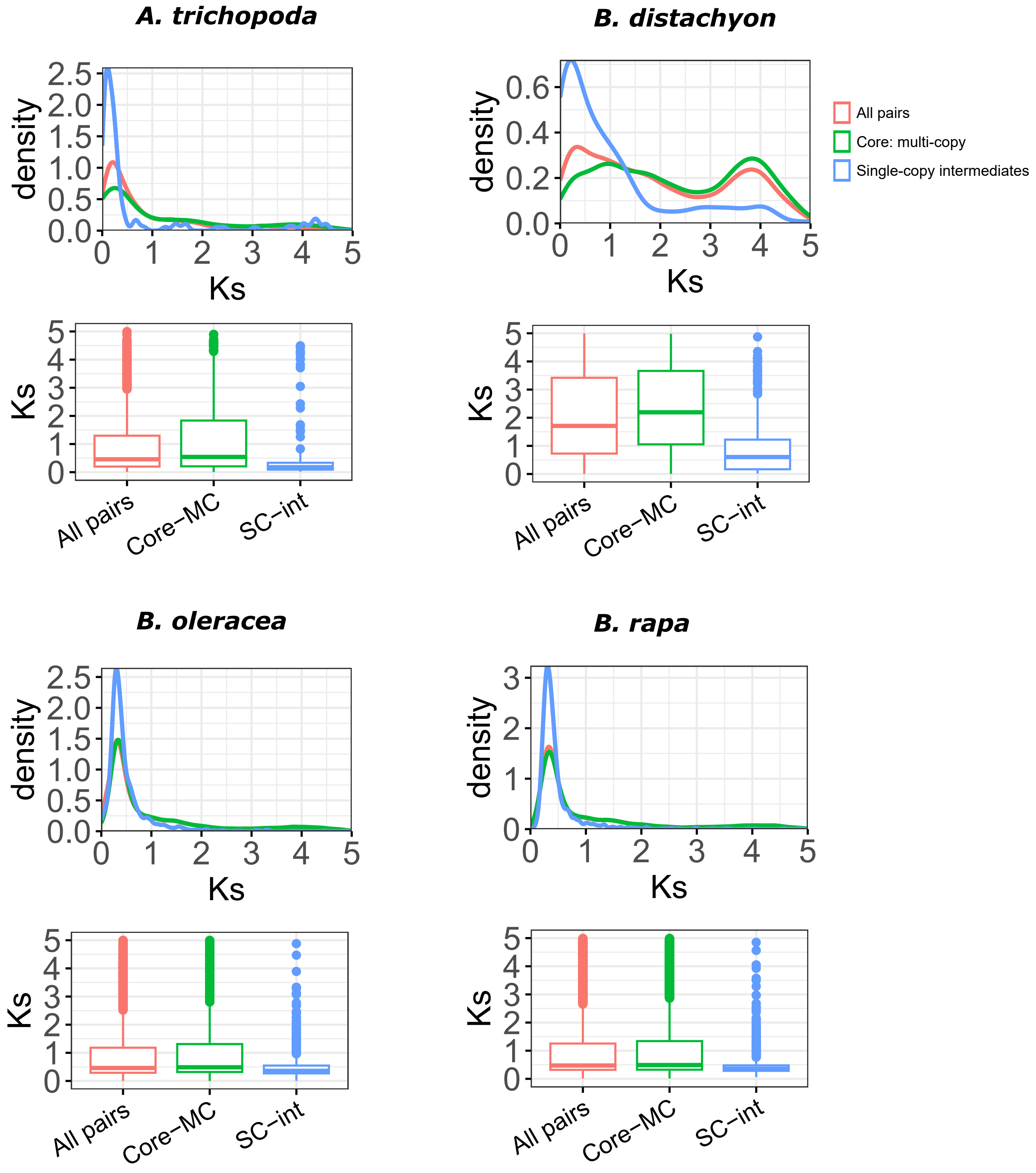

Supplemental Figure S9: **Distribution of core: multi-copy and core: single-copy (intermediate) paralogs based on synonymous substitutions (Ks)**. 'All pairs' represent Ks values of all duplicate gene pairs in the genome, 'Core-MC' represents duplicate pairs among core: multi-copy orthogroup, while 'SC-Int' represent duplicate pairs among the core: single-copy orthogroups. Center line in the boxplot represents the median Ks values, while the box limits represent 25% and 75% percentile of the interquartile range, whiskers represent 1.5 times above or below the interquartile range and dots represents outliers.

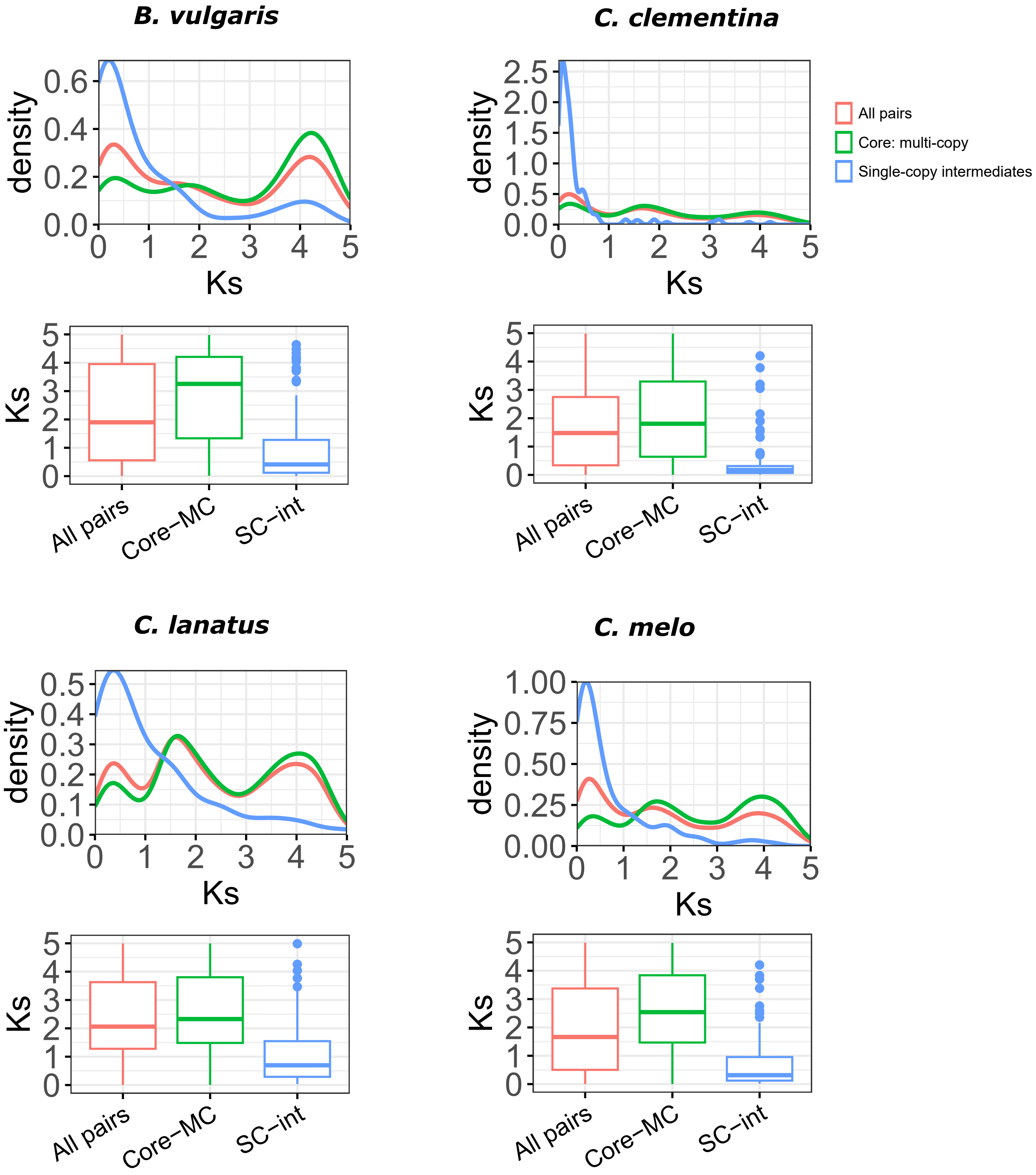

Supplemental Figure S9: **Distribution of core: multi-copy and core: single-copy (intermediate) paralogs based on synonymous substitutions (Ks)**. 'All pairs' represent Ks values of all duplicate gene pairs in the genome, 'Core-MC' represents duplicate pairs among core: multi-copy orthogroup, while 'SC-Int' represent duplicate pairs among the core: single-copy orthogroups. Center line in the boxplot represents the median Ks values, while the box limits represent 25% and 75% percentile of the interquartile range, whiskers represent 1.5 times above or below the interquartile range and dots represents outliers.

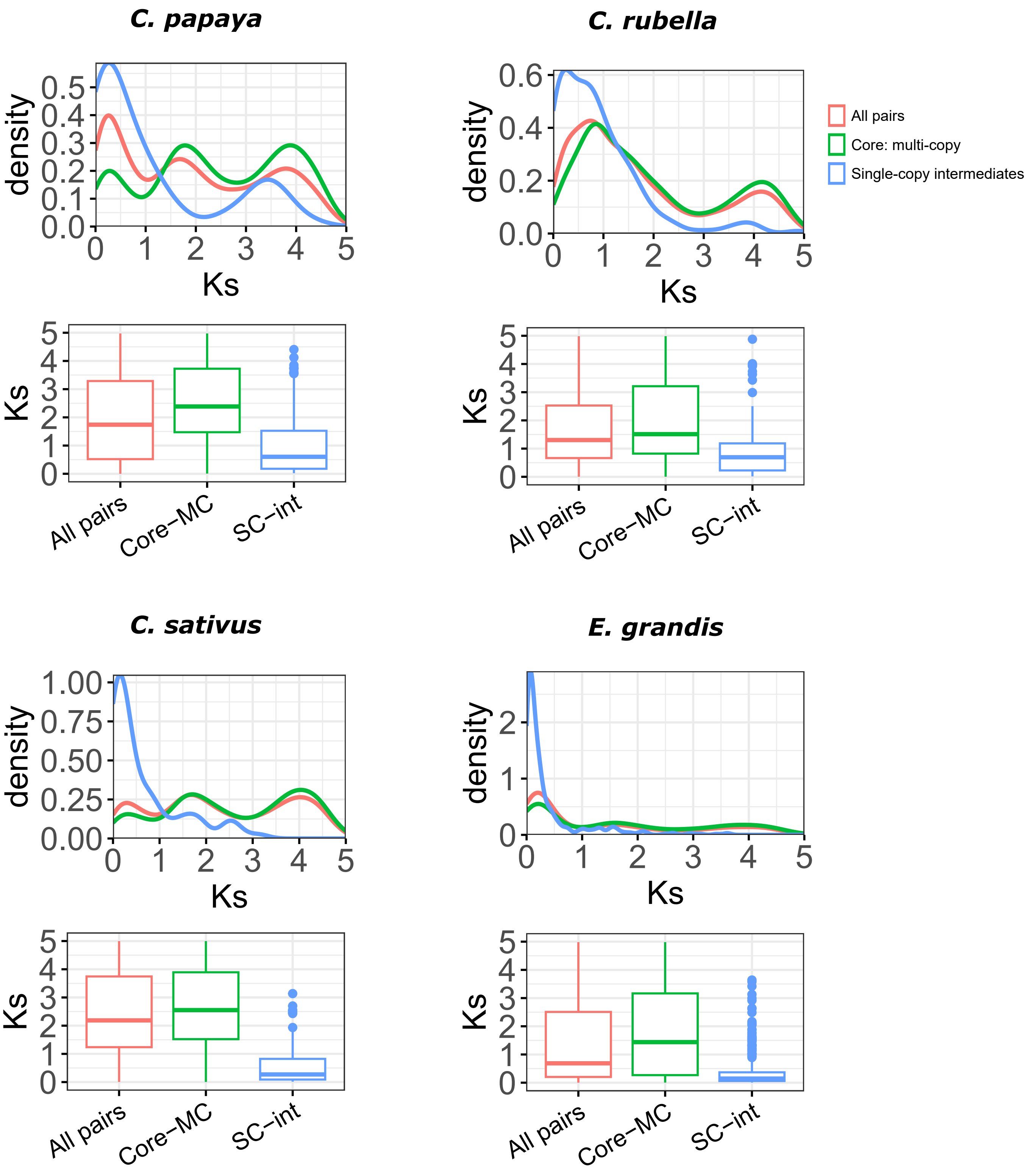

Supplemental Figure S9: **Distribution of core: multi-copy and core: single-copy (intermediate) paralogs based on synonymous substitutions (Ks)**. 'All pairs' represent Ks values of all duplicate gene pairs in the genome, 'Core-MC' represents duplicate pairs among core: multi-copy orthogroup, while 'SC-Int' represent duplicate pairs among the core: single-copy orthogroups. Center line in the boxplot represents the median Ks values, while the box limits represent 25% and 75% percentile of the interquartile range, whiskers represent 1.5 times above or below the interquartile range and dots represents outliers.

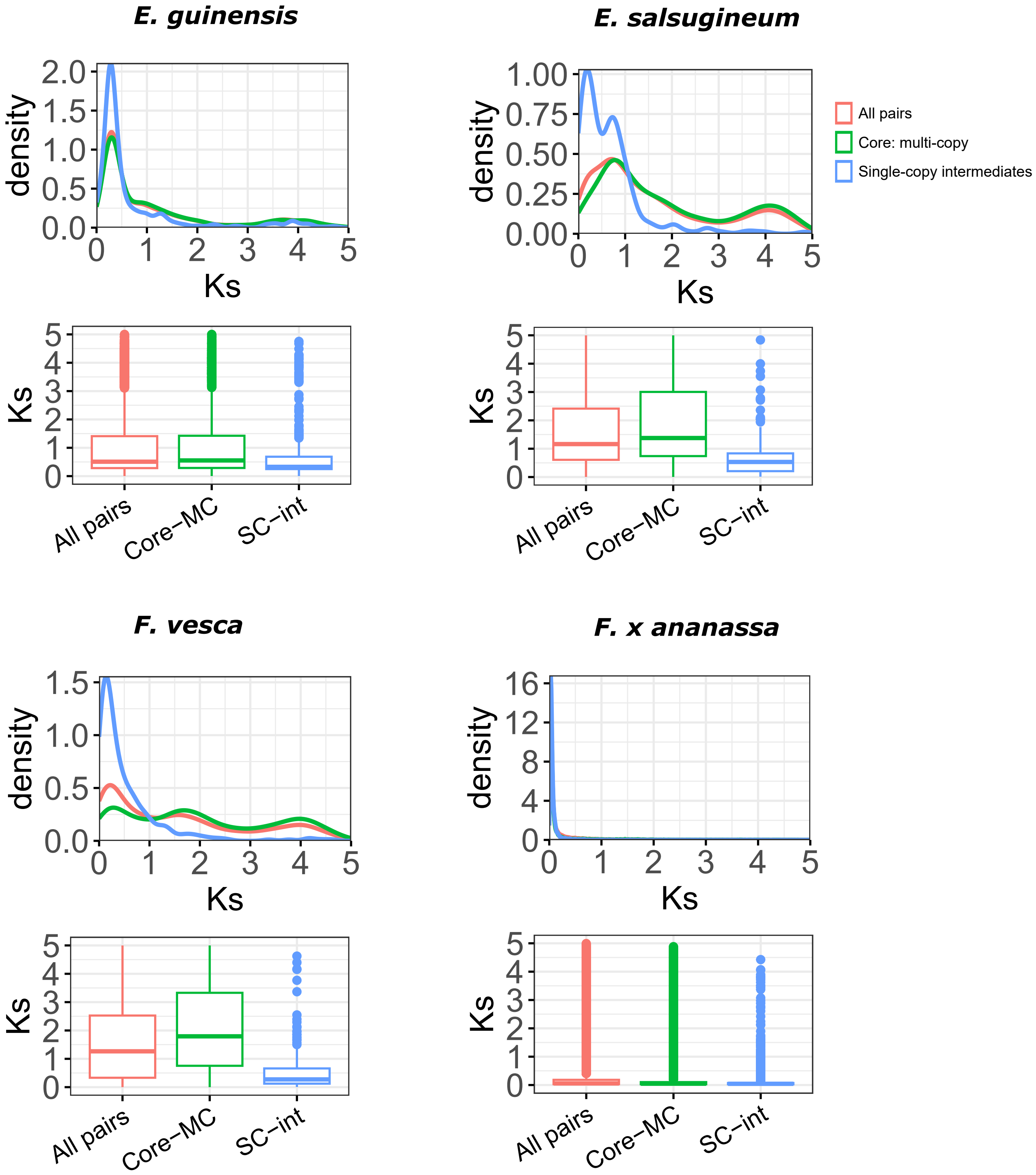

Supplemental Figure S9: **Distribution of core: multi-copy and core: single-copy (intermediate) paralogs based on synonymous substitutions (Ks)**. 'All pairs' represent Ks values of all duplicate gene pairs in the genome, 'Core-MC' represents duplicate pairs among core: multi-copy orthogroup, while 'SC-Int' represent duplicate pairs among the core: single-copy orthogroups. Center line in the boxplot represents the median Ks values, while the box limits represent 25% and 75% percentile of the interquartile range, whiskers represent 1.5 times above or below the interquartile range and dots represents outliers.

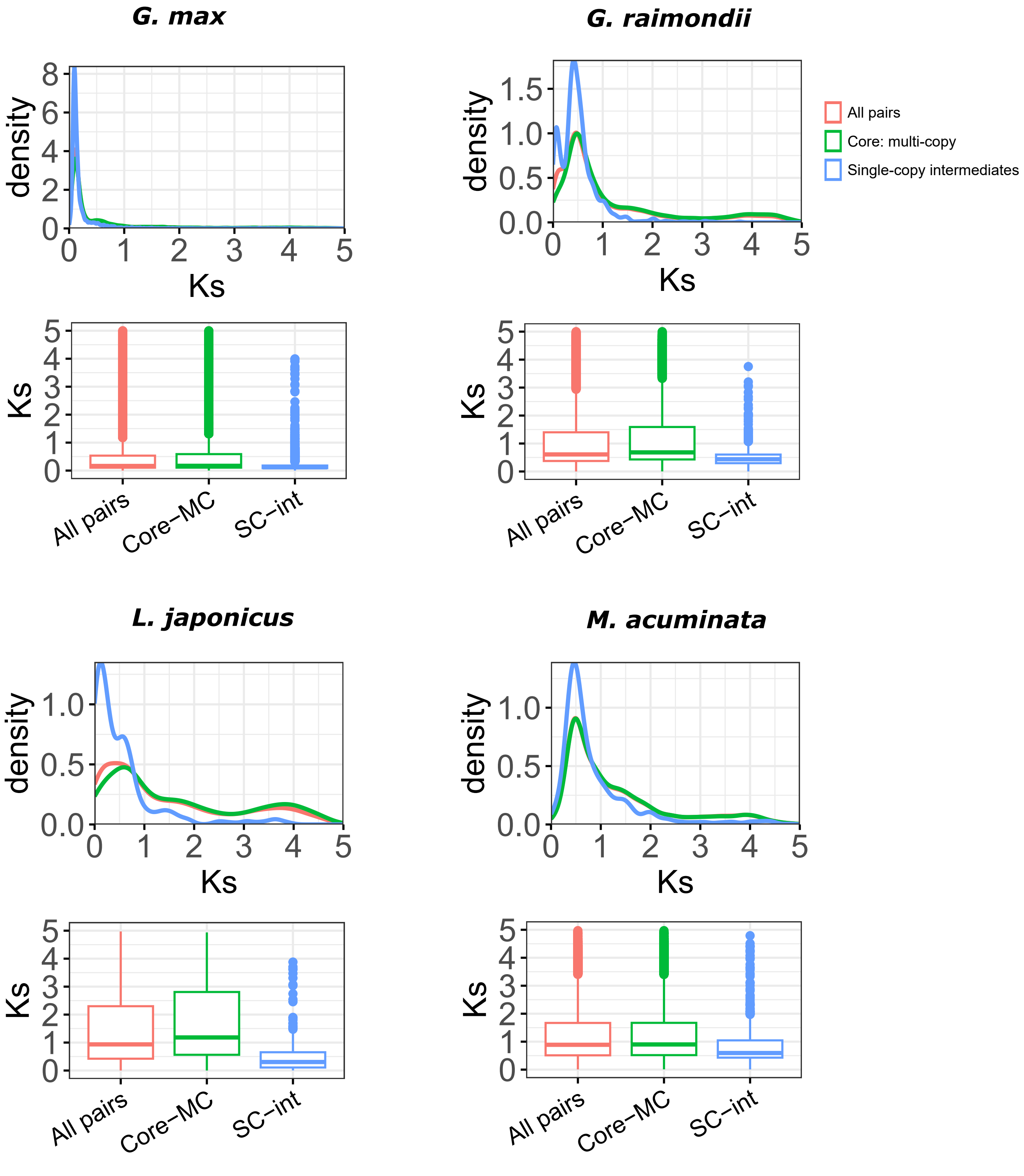

Supplemental Figure S9: **Distribution of core: multi-copy and core: single-copy (intermediate) paralogs based on synonymous substitutions (Ks)**. 'All pairs' represent Ks values of all duplicate gene pairs in the genome, 'Core-MC' represents duplicate pairs among core: multi-copy orthogroup, while 'SC-Int' represent duplicate pairs among the core: single-copy orthogroups. Center line in the boxplot represents the median Ks values, while the box limits represent 25% and 75% percentile of the interquartile range, whiskers represent 1.5 times above or below the interquartile range and dots represents outliers.

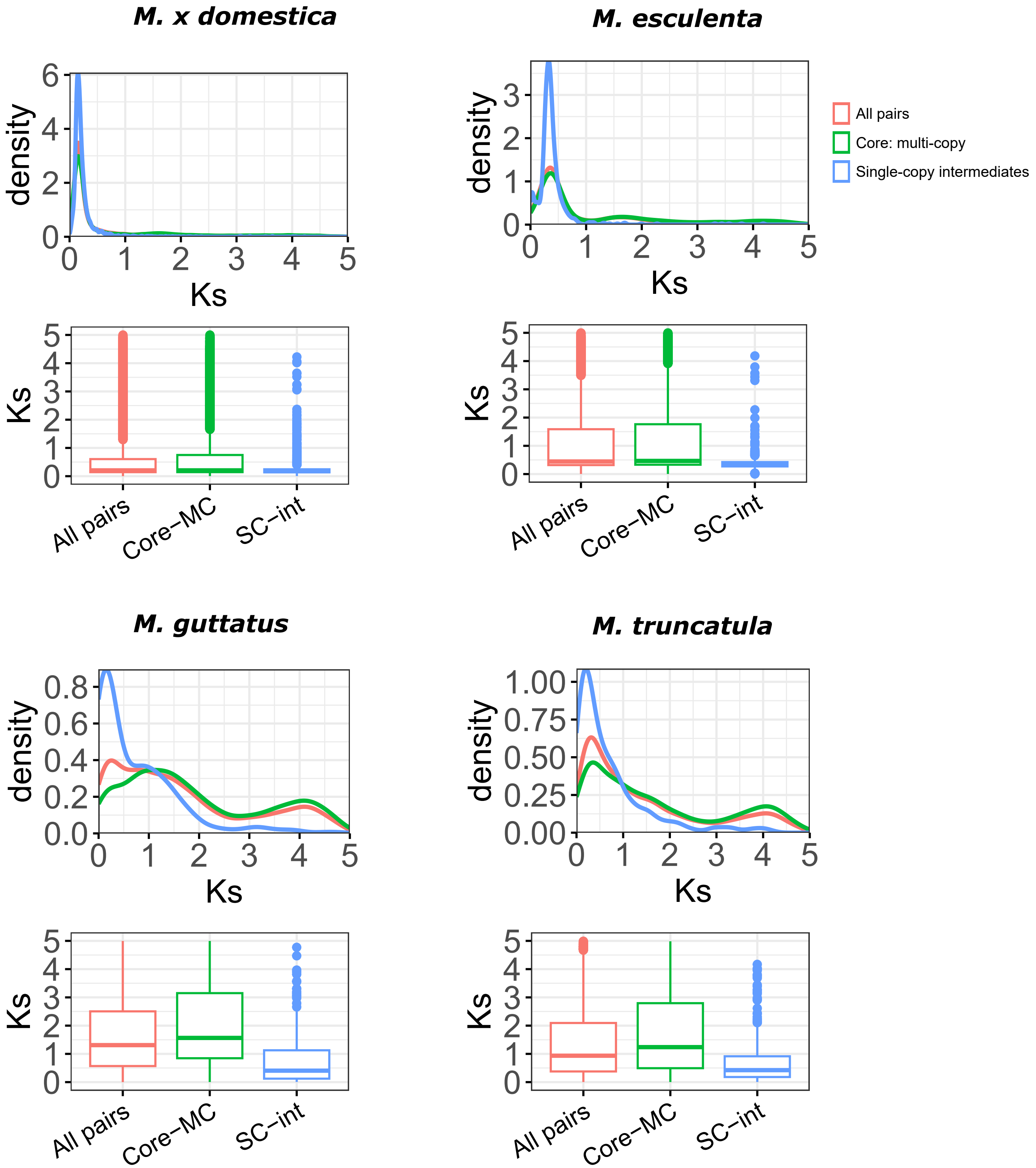

Supplemental Figure S9: **Distribution of core: multi-copy and core: single-copy (intermediate) paralogs based on synonymous substitutions (Ks)**. 'All pairs' represent Ks values of all duplicate gene pairs in the genome, 'Core-MC' represents duplicate pairs among core: multi-copy orthogroup, while 'SC-Int' represent duplicate pairs among the core: single-copy orthogroups. Center line in the boxplot represents the median Ks values, while the box limits represent 25% and 75% percentile of the interquartile range, whiskers represent 1.5 times above or below the interquartile range and dots represents outliers.

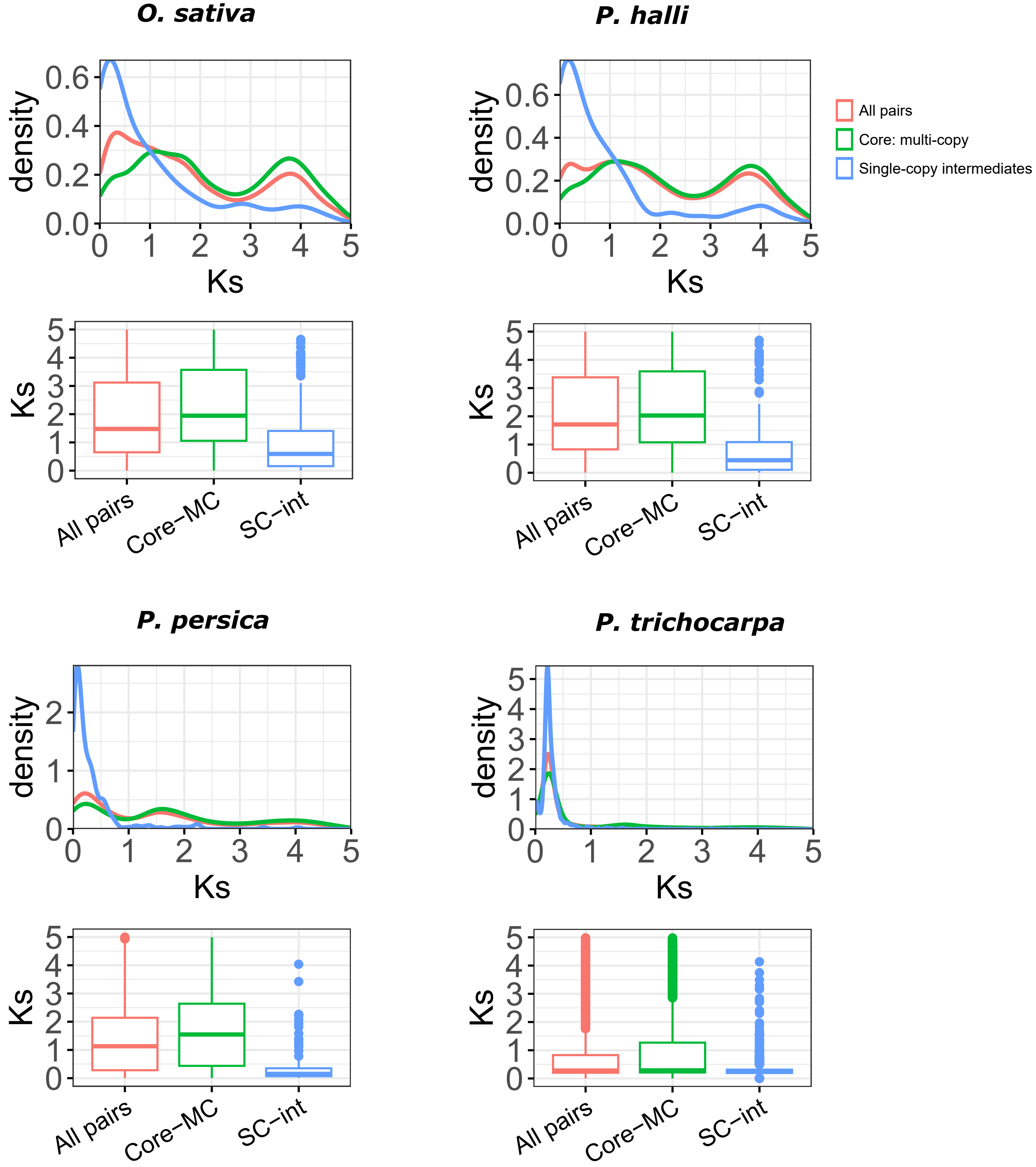

Supplemental Figure S9: **Distribution of core: multi-copy and core: single-copy (intermediate) paralogs based on synonymous substitutions (Ks)**. 'All pairs' represent Ks values of all duplicate gene pairs in the genome, 'Core-MC' represents duplicate pairs among core: multi-copy orthogroup, while 'SC-Int' represent duplicate pairs among the core: single-copy orthogroups. Center line in the boxplot represents the median Ks values, while the box limits represent 25% and 75% percentile of the interquartile range, whiskers represent 1.5 times above or below the interquartile range and dots represents outliers.

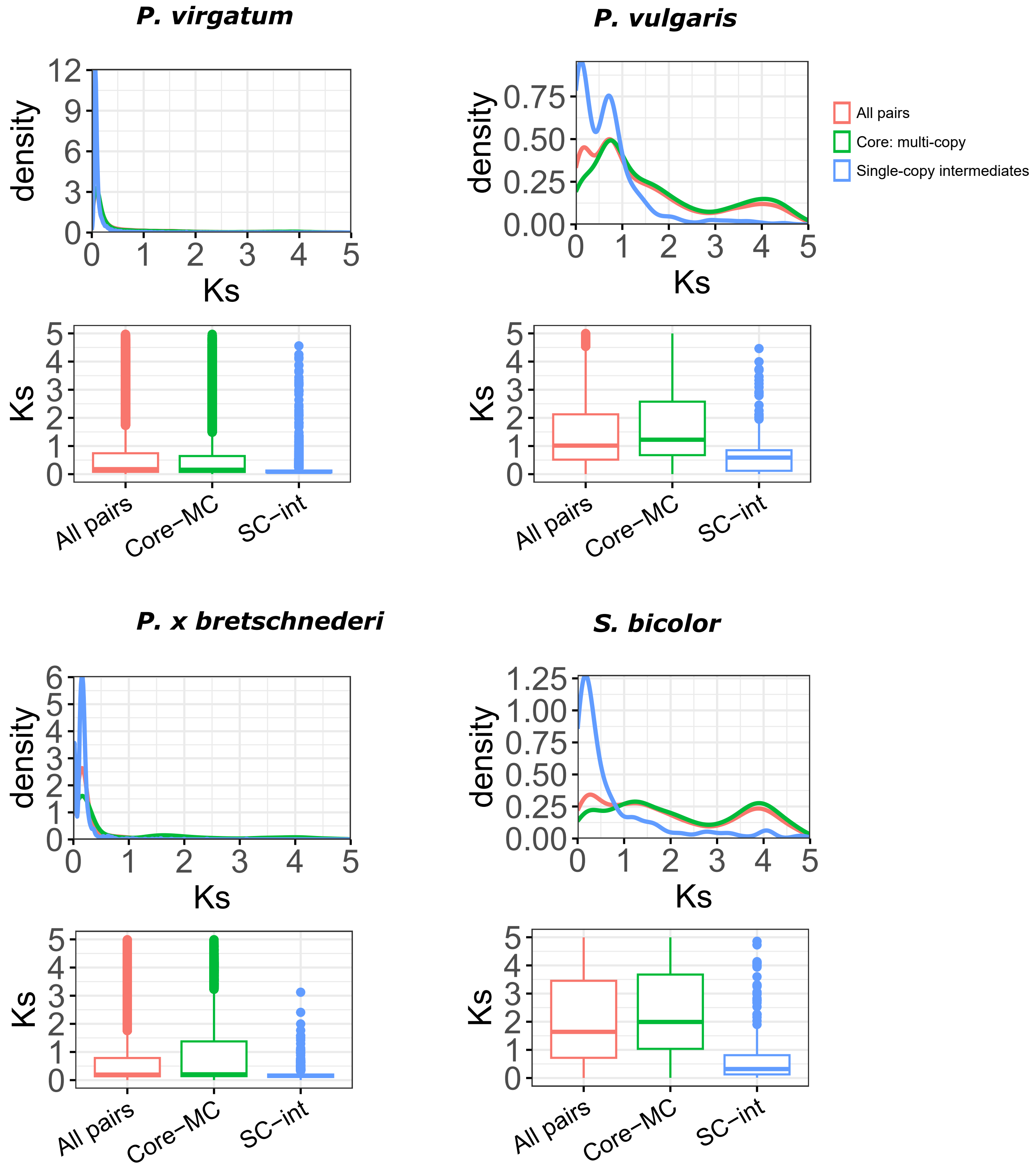

Supplemental Figure S9: **Distribution of core: multi-copy and core: single-copy (intermediate) paralogs based on synonymous substitutions (Ks)**. 'All pairs' represent Ks values of all duplicate gene pairs in the genome, 'Core-MC' represents duplicate pairs among core: multi-copy orthogroup, while 'SC-Int' represent duplicate pairs among the core: single-copy orthogroups. Center line in the boxplot represents the median Ks values, while the box limits represent 25% and 75% percentile of the interquartile range, whiskers represent 1.5 times above or below the interquartile range and dots represents outliers.

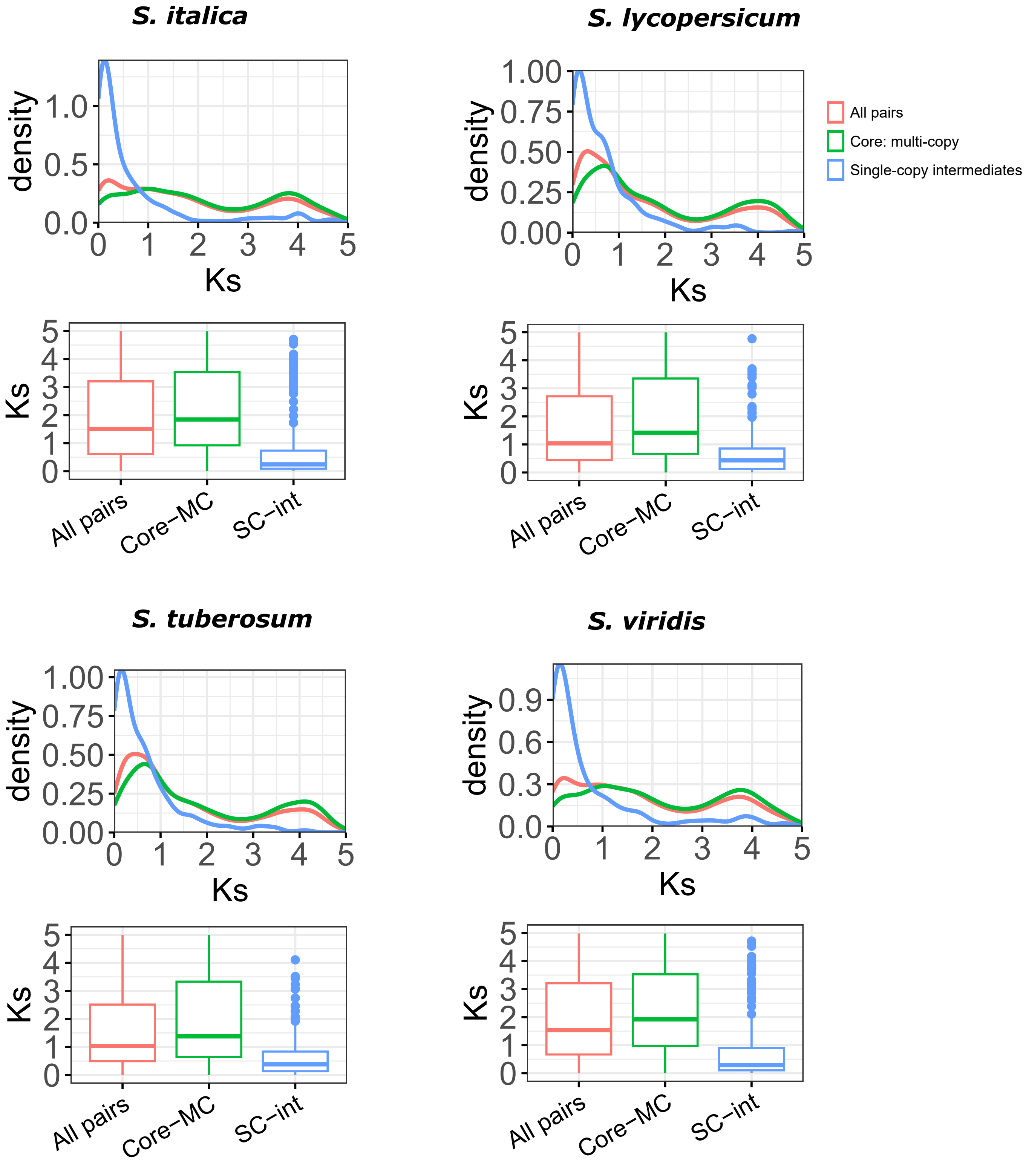

Supplemental Figure S9: **Distribution of core: multi-copy and core: single-copy (intermediate) paralogs based on synonymous substitutions (Ks)**. 'All pairs' represent Ks values of all duplicate gene pairs in the genome, 'Core-MC' represents duplicate pairs among core: multi-copy orthogroup, while 'SC-Int' represent duplicate pairs among the core: single-copy orthogroups. Center line in the boxplot represents the median Ks values, while the box limits represent 25% and 75% percentile of the interquartile range, whiskers represent 1.5 times above or below the interquartile range and dots represents outliers.

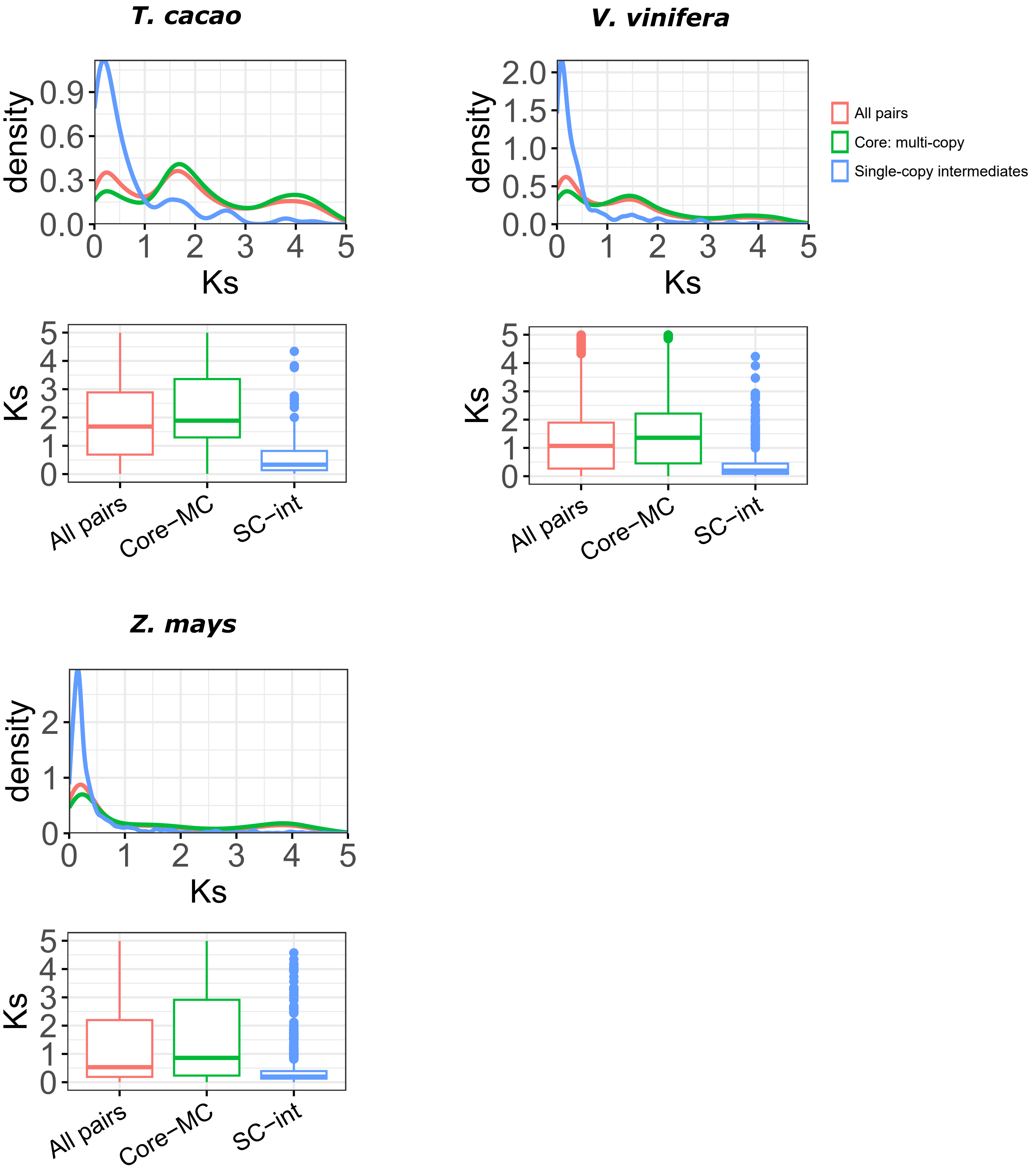

Supplemental Figure S10: **Distribution of genic methylation classified genes based on the ratio of nonsynonymous substitution (Ka), with synonymous substitutions (Ks) across different types of gene duplicate pairs.** Whole-genome duplicates - WGD, Single-gene duplicates - SGD (combined data from tandem, proximal, translocated, and dispersed duplicates). Center line in the boxplot represents the median of Ka/Ks ratios, while the box limits represent 25% and 75% percentile of the interquartile range, whiskers represent 1.5 times above or below the interquartile range and dots represents outliers.

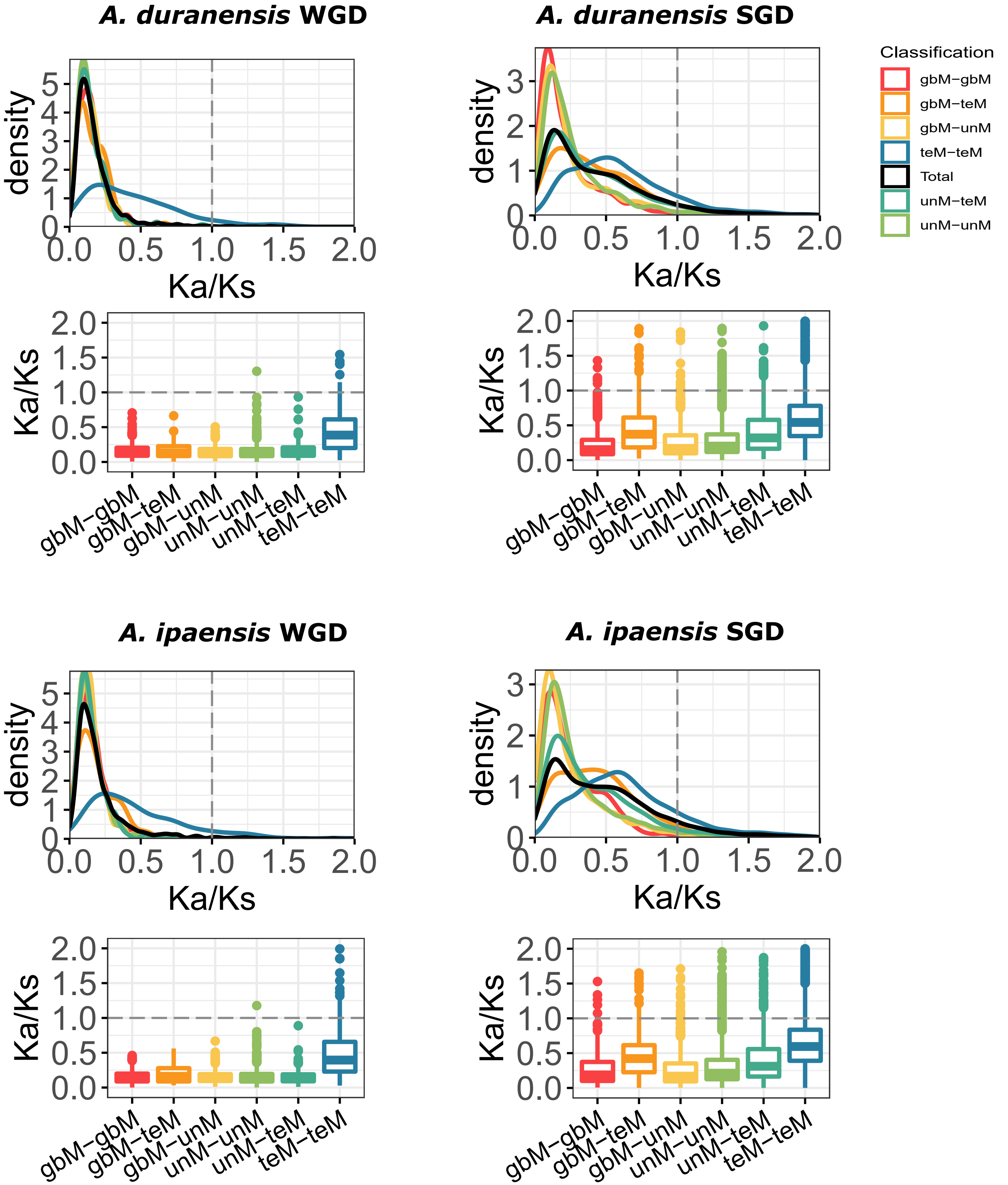

Supplemental Figure S10: **Distribution of genic methylation classified genes based on the ratio of nonsynonymous substitution (Ka), with synonymous substitutions (Ks) across different types of gene duplicate pairs.** Whole-genome duplicates - WGD, Single-gene duplicates - SGD (combined data from tandem, proximal, translocated, and dispersed duplicates). Center line in the boxplot represents the median of Ka/Ks ratios, while the box limits represent 25% and 75% percentile of the interquartile range, whiskers represent 1.5 times above or below the interquartile range and dots represents outliers.

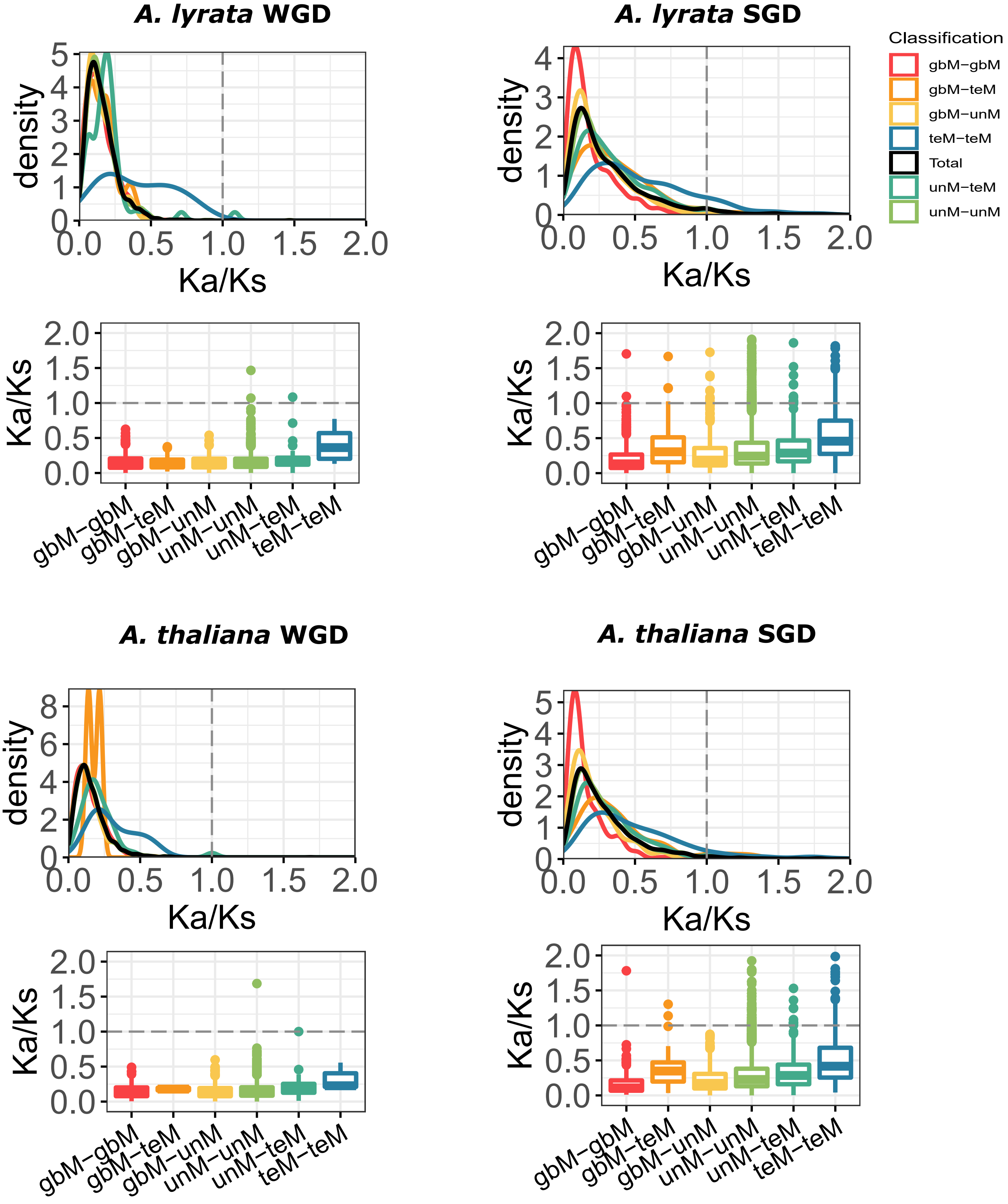

Supplemental Figure S10: **Distribution of genic methylation classified genes based on the ratio of nonsynonymous substitution (Ka), with synonymous substitutions (Ks) across different types of gene duplicate pairs.** Whole-genome duplicates - WGD, Single-gene duplicates - SGD (combined data from tandem, proximal, translocated, and dispersed duplicates). Center line in the boxplot represents the median of Ka/Ks ratios, while the box limits represent 25% and 75% percentile of the interquartile range, whiskers represent 1.5 times above or below the interquartile range and dots represents outliers.

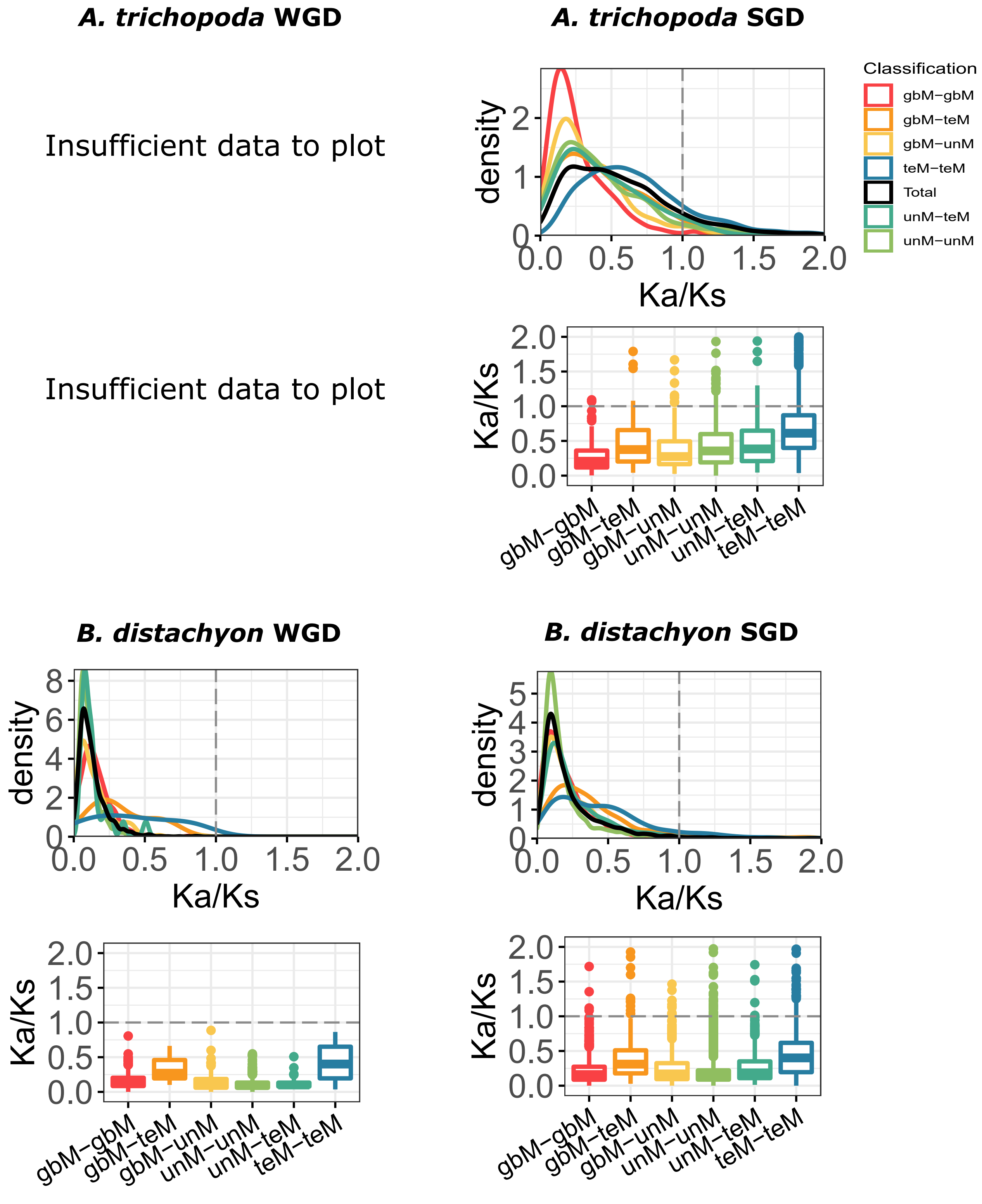

Supplemental Figure S10: **Distribution of genic methylation classified genes based on the ratio of nonsynonymous substitution (Ka), with synonymous substitutions (Ks) across different types of gene duplicate pairs.** Whole-genome duplicates - WGD, Single-gene duplicates - SGD (combined data from tandem, proximal, translocated, and dispersed duplicates). Center line in the boxplot represents the median of Ka/Ks ratios, while the box limits represent 25% and 75% percentile of the interquartile range, whiskers represent 1.5 times above or below the interquartile range and dots represents outliers.

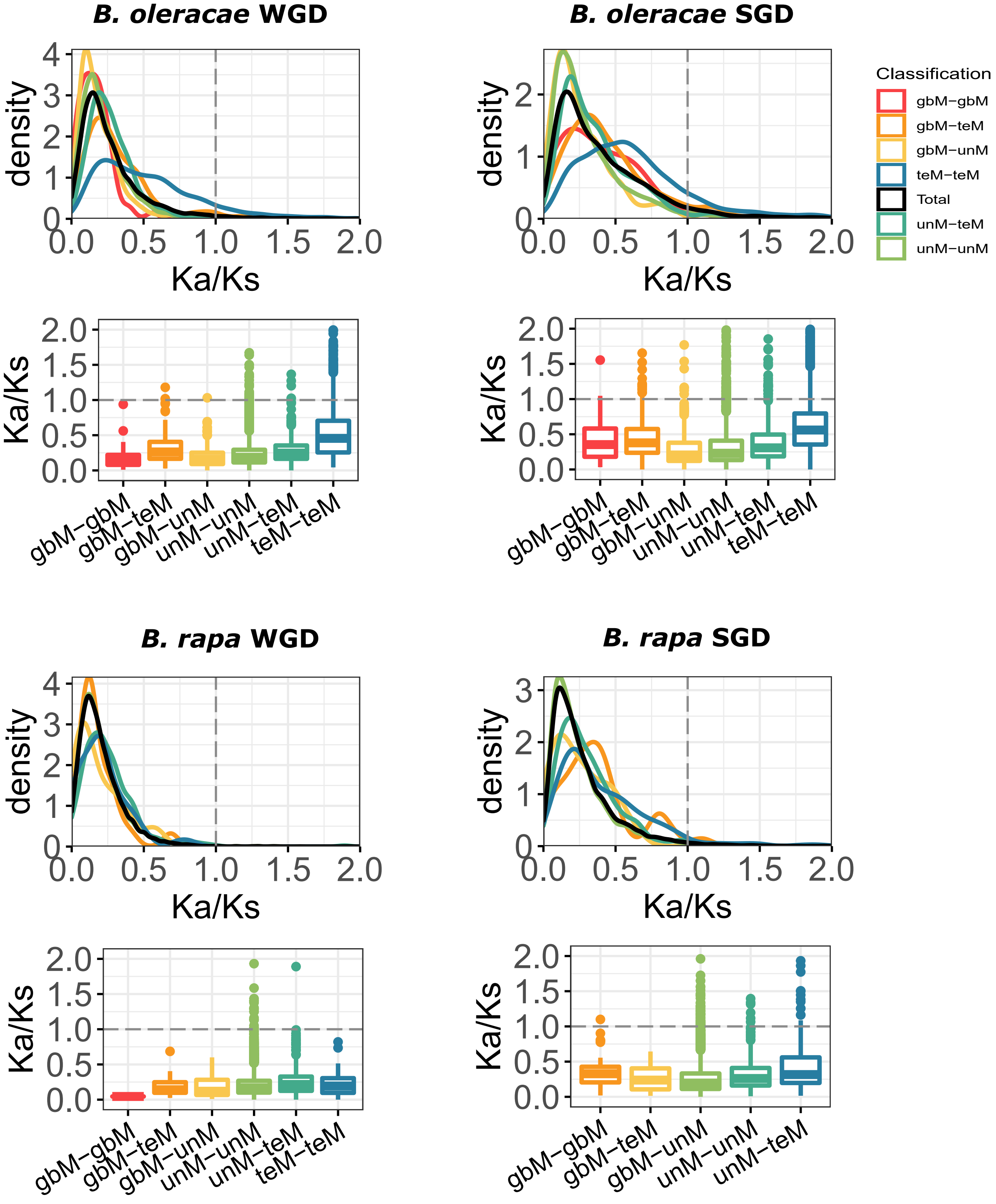

Supplemental Figure S10: **Distribution of genic methylation classified genes based on the ratio of nonsynonymous substitution (Ka), with synonymous substitutions (Ks) across different types of gene duplicate pairs.** Whole-genome duplicates - WGD, Single-gene duplicates - SGD (combined data from tandem, proximal, translocated, and dispersed duplicates). Center line in the boxplot represents the median of Ka/Ks ratios, while the box limits represent 25% and 75% percentile of the interquartile range, whiskers represent 1.5 times above or below the interquartile range and dots represents outliers.

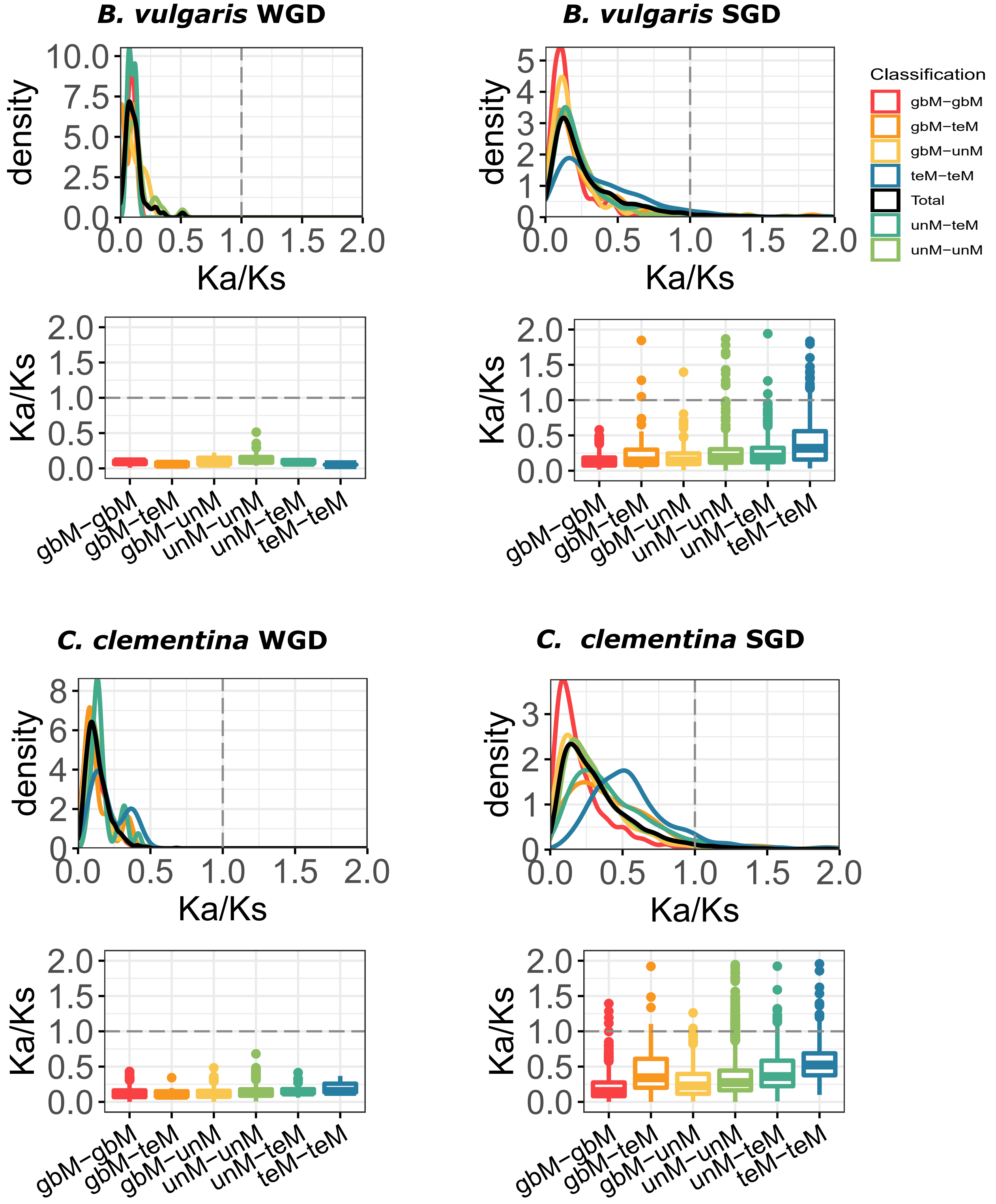

Supplemental Figure S10: **Distribution of genic methylation classified genes based on the ratio of nonsynonymous substitution (Ka), with synonymous substitutions (Ks) across different types of gene duplicate pairs.** Whole-genome duplicates - WGD, Single-gene duplicates - SGD (combined data from tandem, proximal, translocated, and dispersed duplicates). Center line in the boxplot represents the median of Ka/Ks ratios, while the box limits represent 25% and 75% percentile of the interquartile range, whiskers represent 1.5 times above or below the interquartile range and dots represents outliers.

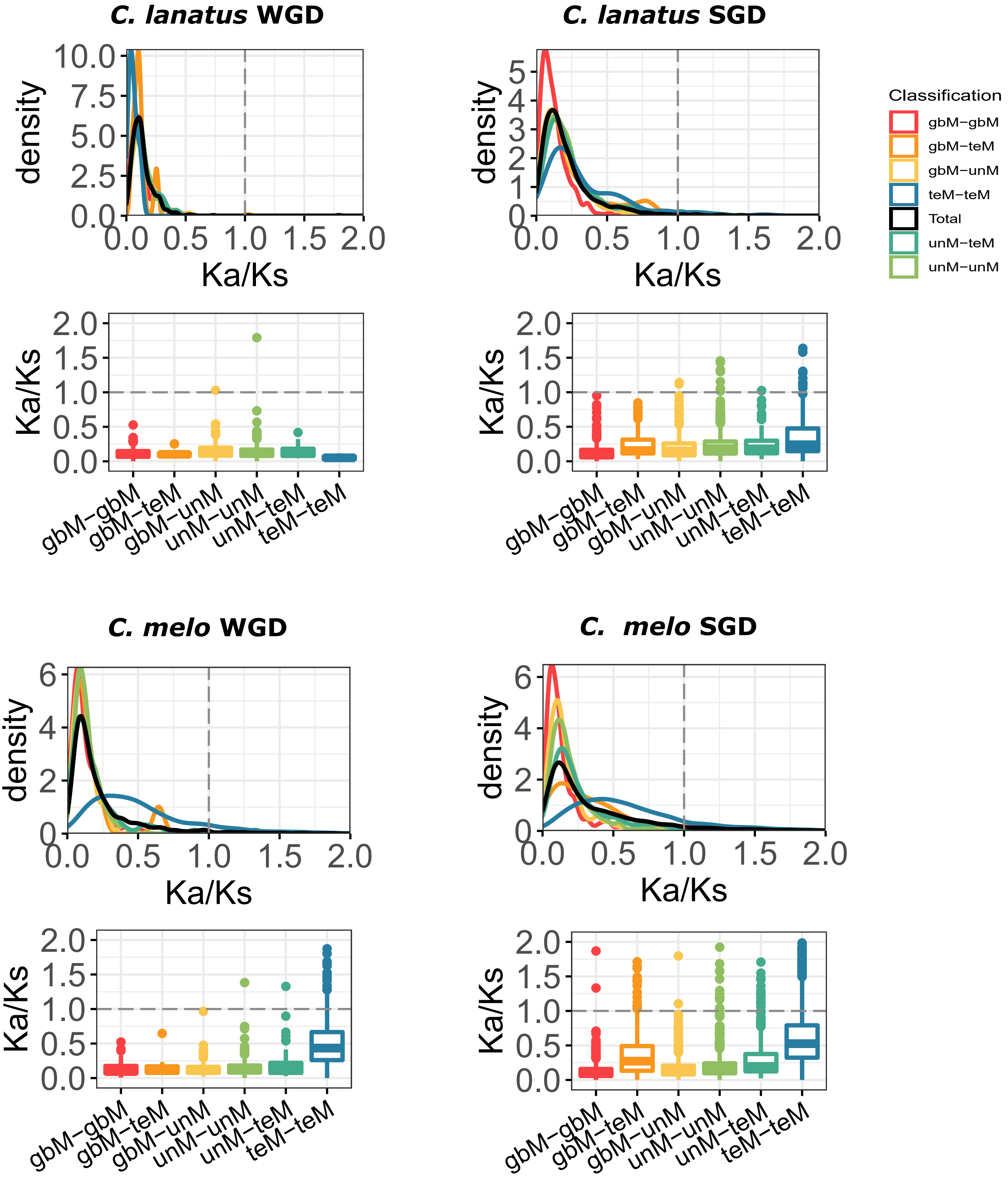

Supplemental Figure S10: **Distribution of genic methylation classified genes based on the ratio of nonsynonymous substitution (Ka), with synonymous substitutions (Ks) across different types of gene duplicate pairs.** Whole-genome duplicates - WGD, Single-gene duplicates - SGD (combined data from tandem, proximal, translocated, and dispersed duplicates). Center line in the boxplot represents the median of Ka/Ks ratios, while the box limits represent 25% and 75% percentile of the interquartile range, whiskers represent 1.5 times above or below the interquartile range and dots represents outliers.

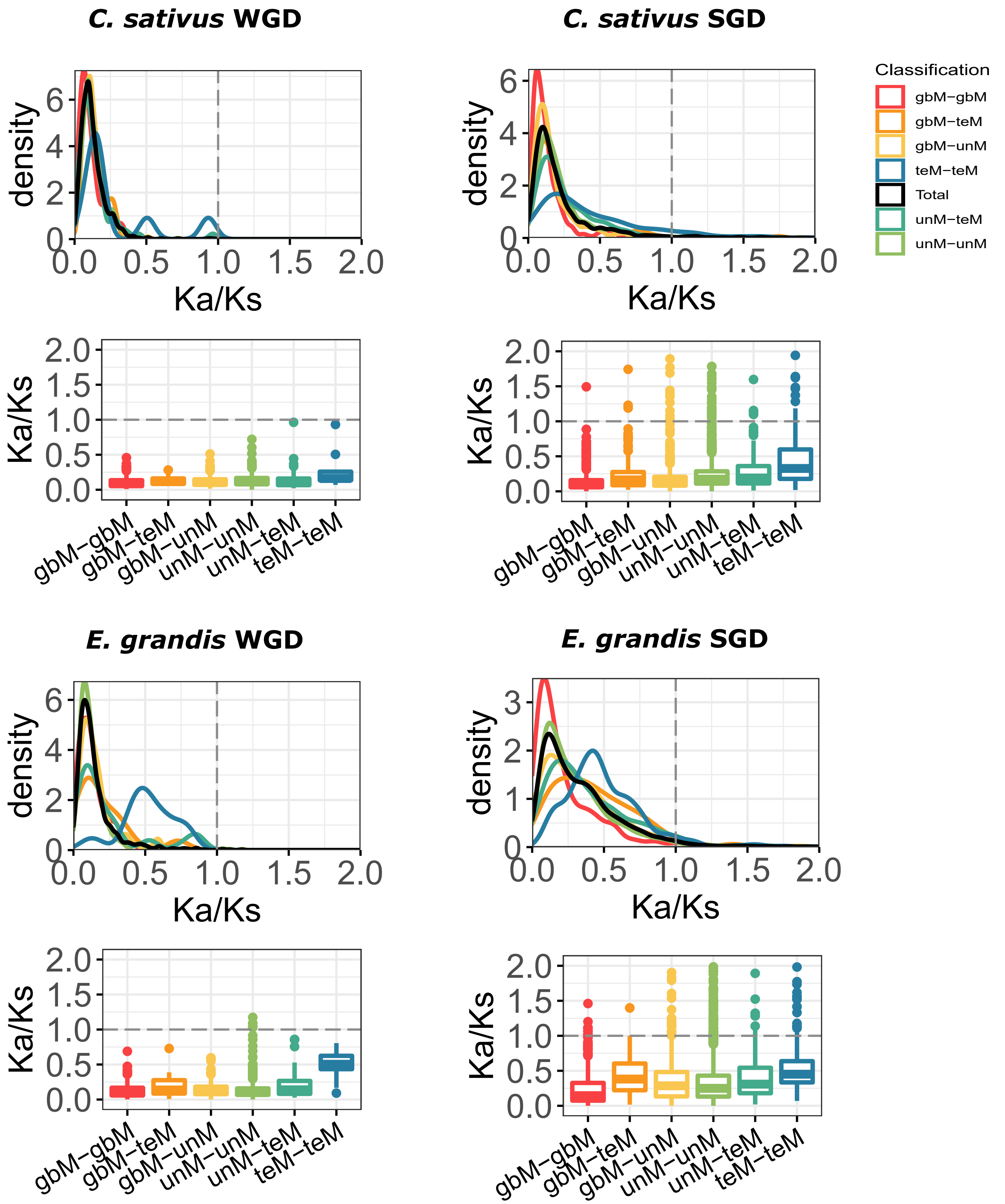

Supplemental Figure S10: **Distribution of genic methylation classified genes based on the ratio of nonsynonymous substitution (Ka), with synonymous substitutions (Ks) across different types of gene duplicate pairs.** Whole-genome duplicates - WGD, Single-gene duplicates - SGD (combined data from tandem, proximal, translocated, and dispersed duplicates). Center line in the boxplot represents the median of Ka/Ks ratios, while the box limits represent 25% and 75% percentile of the interquartile range, whiskers represent 1.5 times above or below the interquartile range and dots represents outliers.

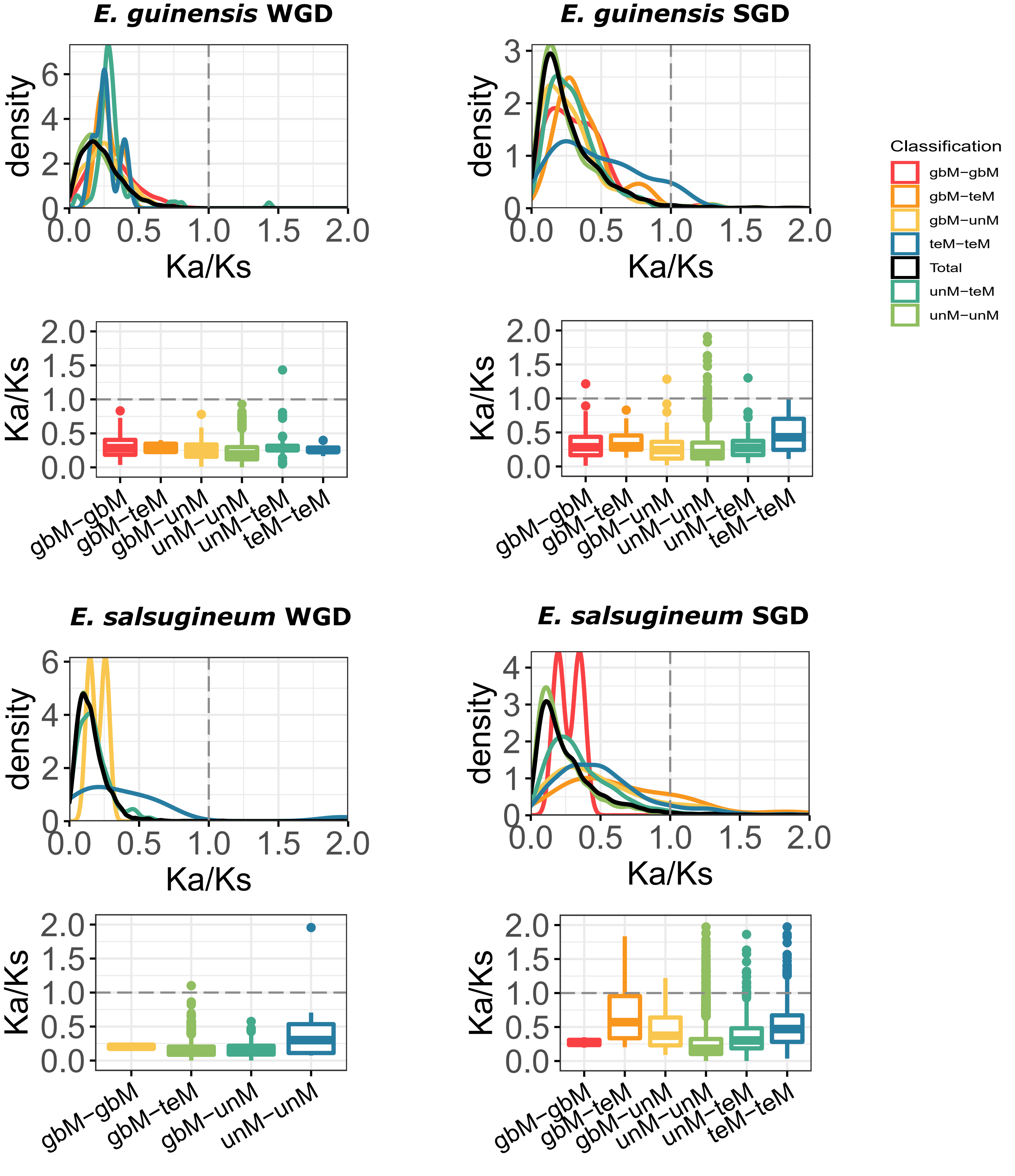

Supplemental Figure S10: **Distribution of genic methylation classified genes based on the ratio of nonsynonymous substitution (Ka), with synonymous substitutions (Ks) across different types of gene duplicate pairs.** Whole-genome duplicates - WGD, Single-gene duplicates - SGD (combined data from tandem, proximal, translocated, and dispersed duplicates). Center line in the boxplot represents the median of Ka/Ks ratios, while the box limits represent 25% and 75% percentile of the interquartile range, whiskers represent 1.5 times above or below the interquartile range and dots represents outliers.

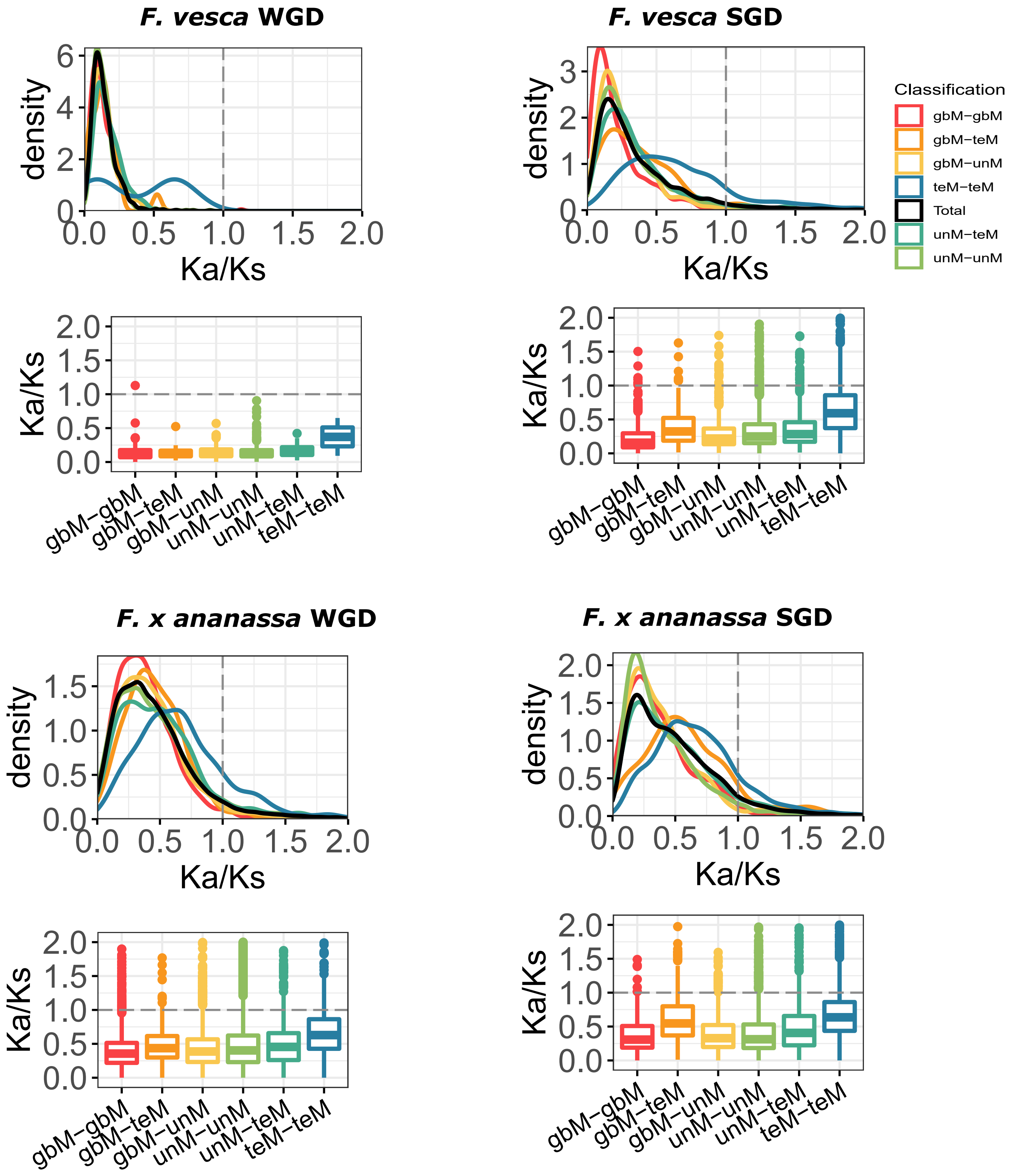

Supplemental Figure S10: **Distribution of genic methylation classified genes based on the ratio of nonsynonymous substitution (Ka), with synonymous substitutions (Ks) across different types of gene duplicate pairs.** Whole-genome duplicates - WGD, Single-gene duplicates - SGD (combined data from tandem, proximal, translocated, and dispersed duplicates). Center line in the boxplot represents the median of Ka/Ks ratios, while the box limits represent 25% and 75% percentile of the interquartile range, whiskers represent 1.5 times above or below the interquartile range and dots represents outliers.

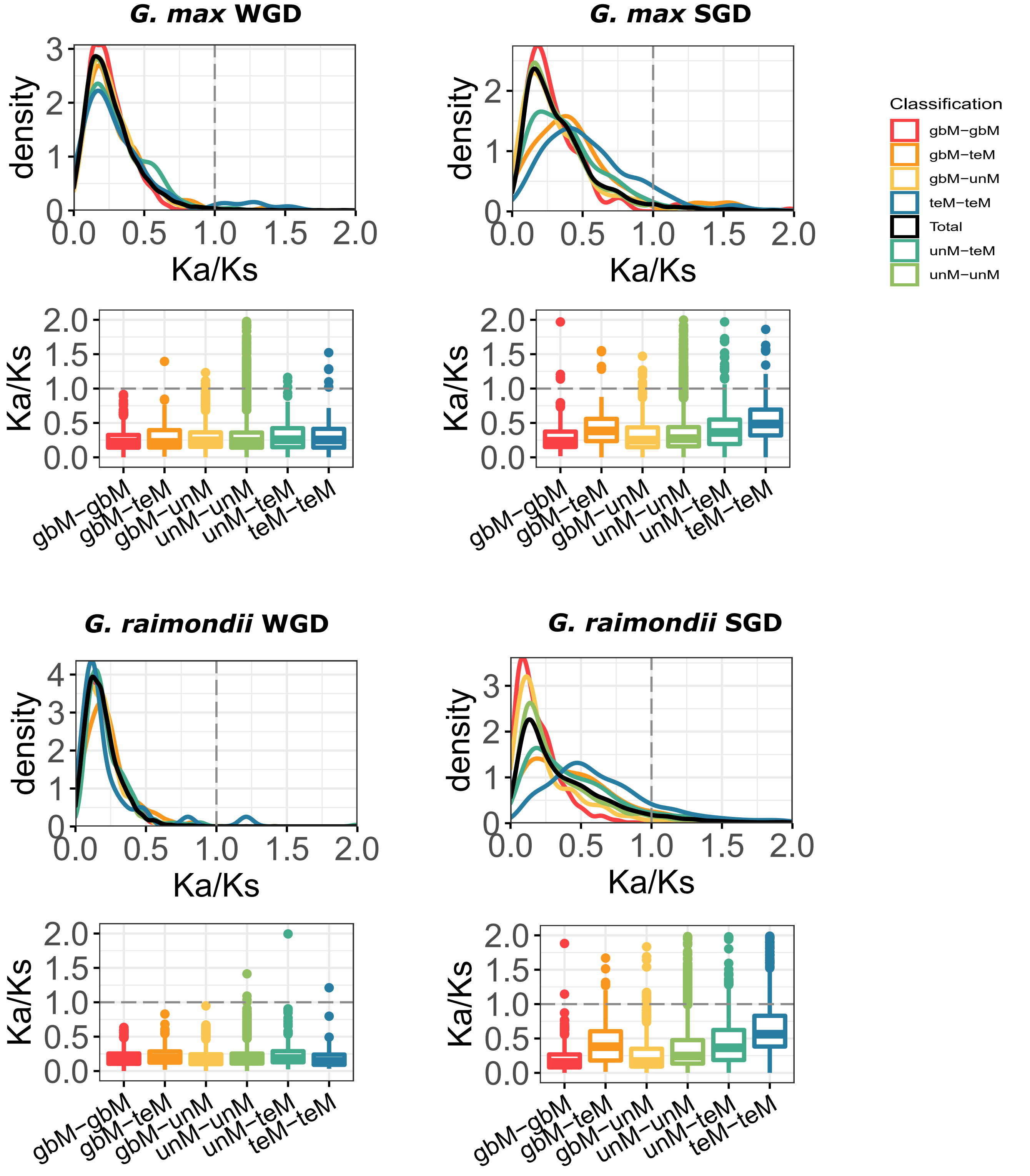

Supplemental Figure S10: **Distribution of genic methylation classified genes based on the ratio of nonsynonymous substitution (Ka), with synonymous substitutions (Ks) across different types of gene duplicate pairs.** Whole-genome duplicates - WGD, Single-gene duplicates - SGD (combined data from tandem, proximal, translocated, and dispersed duplicates). Center line in the boxplot represents the median of Ka/Ks ratios, while the box limits represent 25% and 75% percentile of the interquartile range, whiskers represent 1.5 times above or below the interquartile range and dots represents outliers.

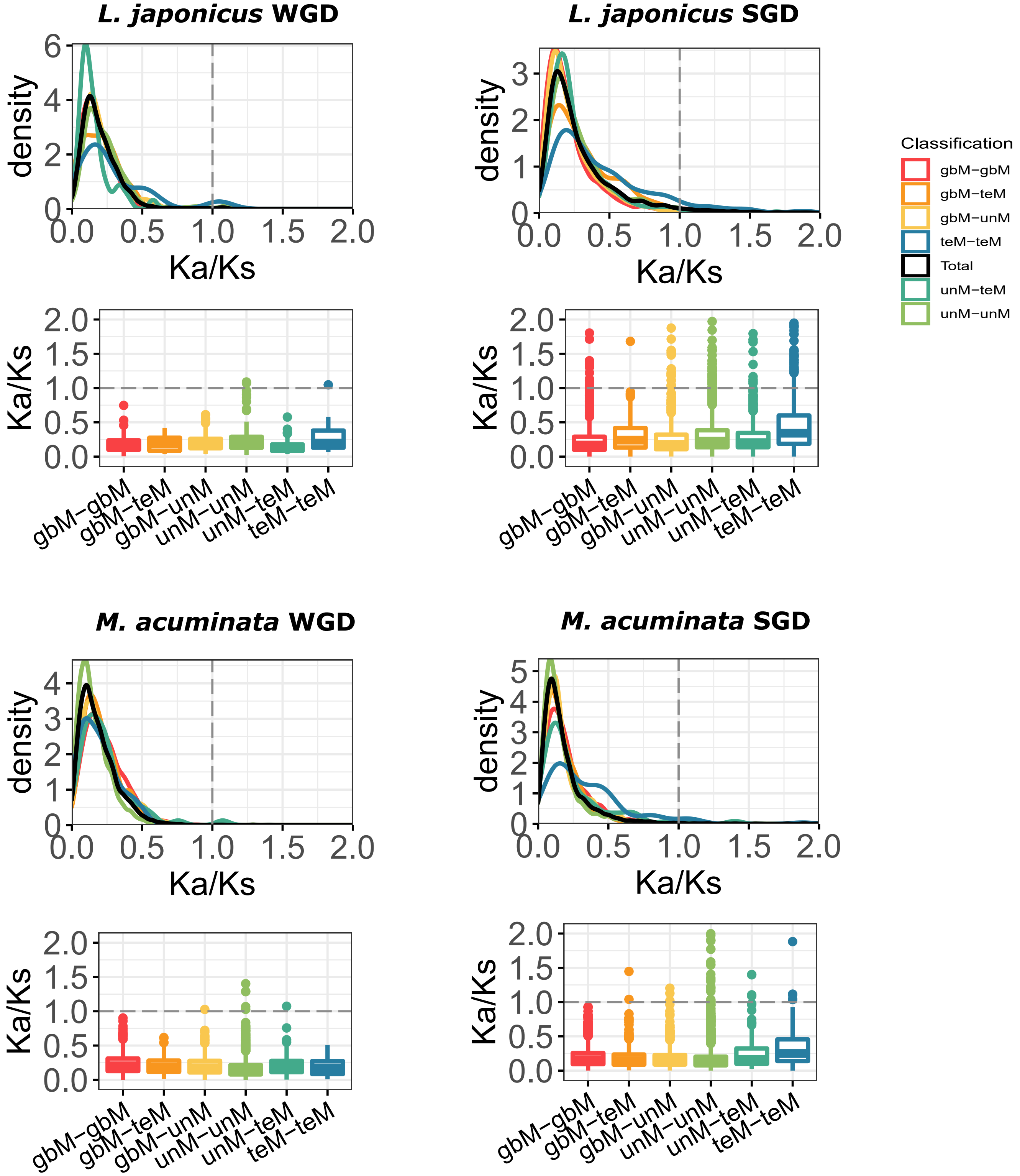

Supplemental Figure S10: **Distribution of genic methylation classified genes based on the ratio of nonsynonymous substitution (Ka), with synonymous substitutions (Ks) across different types of gene duplicate pairs.** Whole-genome duplicates - WGD, Single-gene duplicates - SGD (combined data from tandem, proximal, translocated, and dispersed duplicates). Center line in the boxplot represents the median of Ka/Ks ratios, while the box limits represent 25% and 75% percentile of the interquartile range, whiskers represent 1.5 times above or below the interquartile range and dots represents outliers.

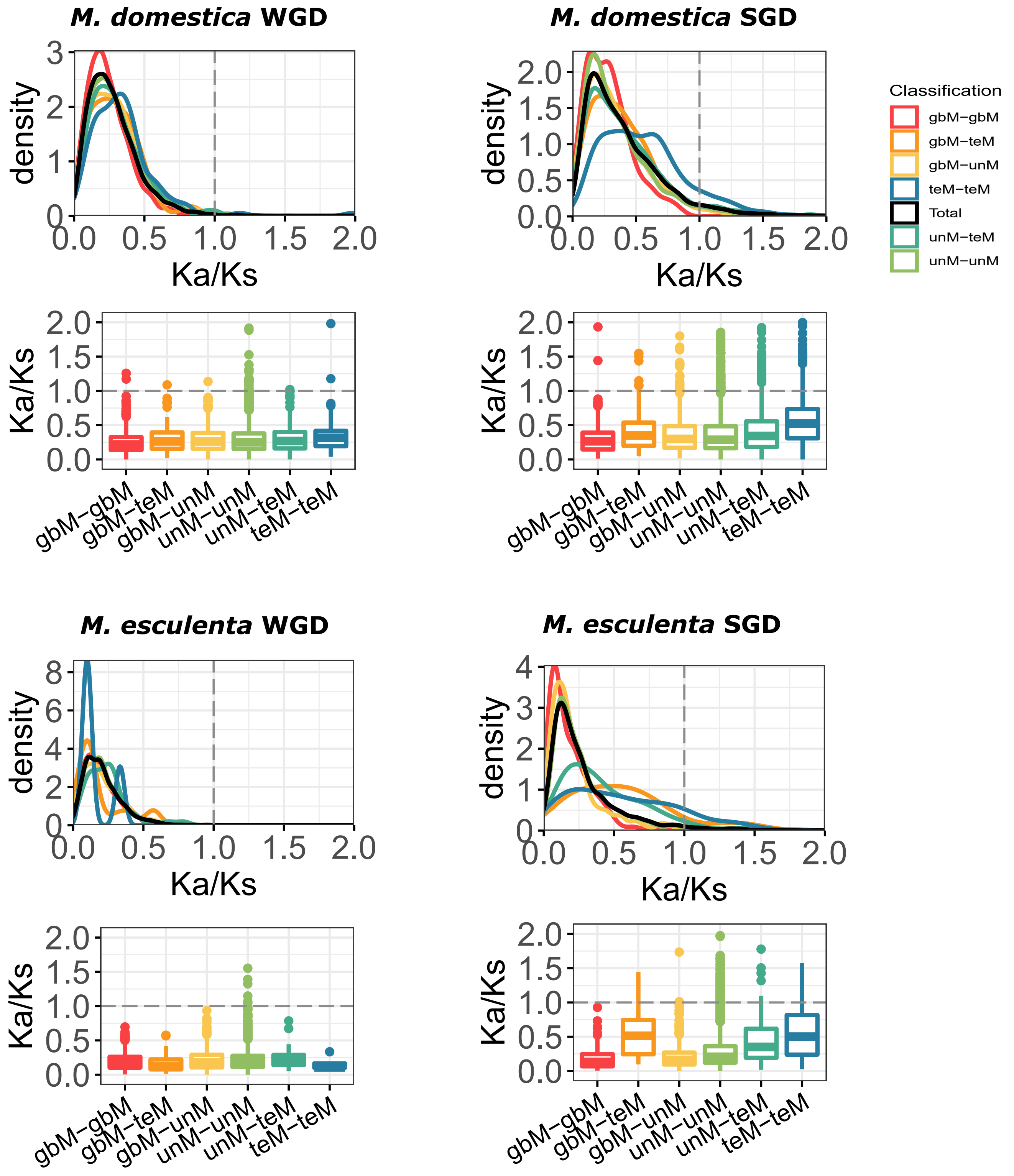

Supplemental Figure S10: **Distribution of genic methylation classified genes based on the ratio of nonsynonymous substitution (Ka), with synonymous substitutions (Ks) across different types of gene duplicate pairs.** Whole-genome duplicates - WGD, Single-gene duplicates - SGD (combined data from tandem, proximal, translocated, and dispersed duplicates). Center line in the boxplot represents the median of Ka/Ks ratios, while the box limits represent 25% and 75% percentile of the interquartile range, whiskers represent 1.5 times above or below the interquartile range and dots represents outliers.

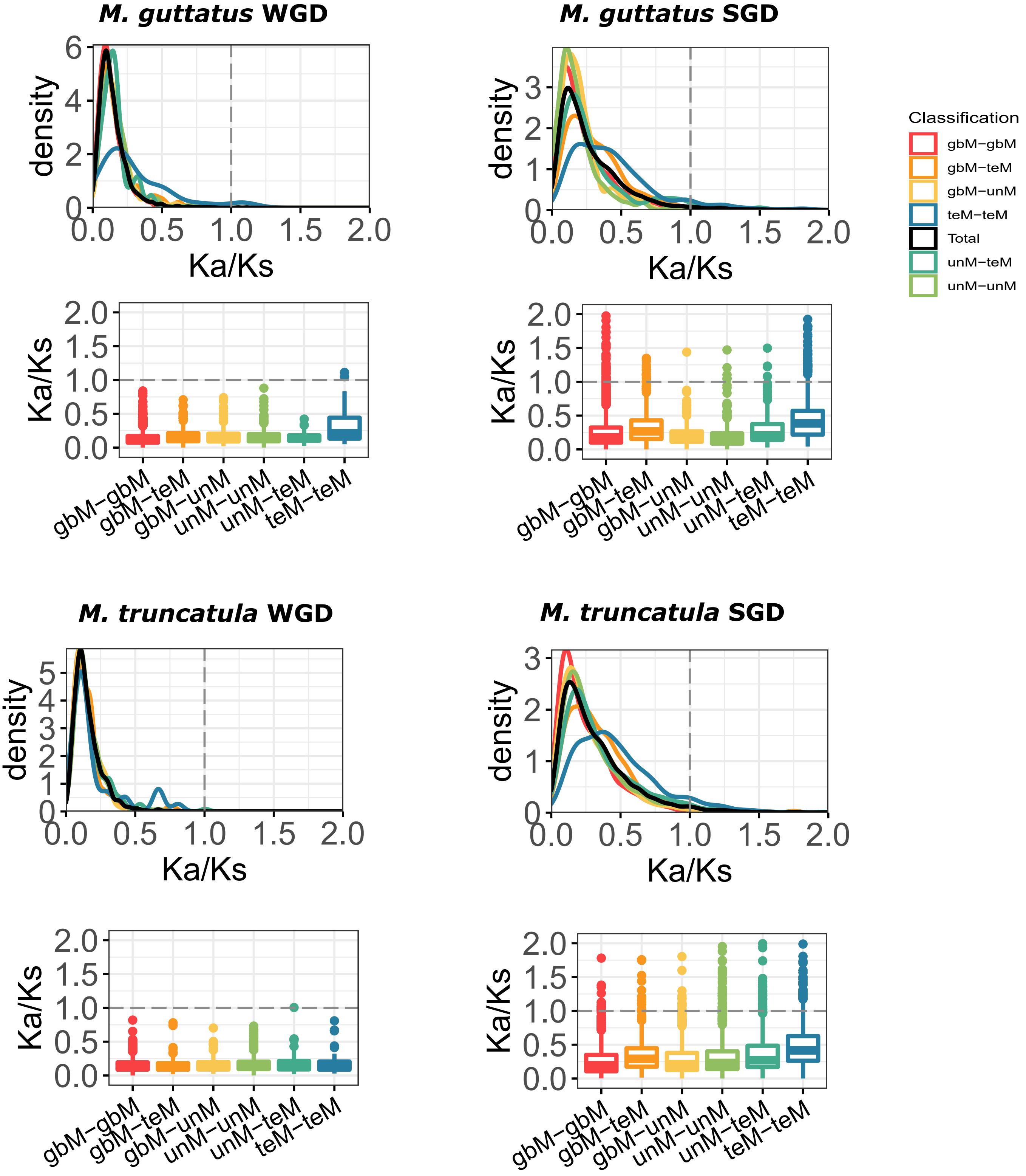

Supplemental Figure S10: **Distribution of genic methylation classified genes based on the ratio of nonsynonymous substitution (Ka), with synonymous substitutions (Ks) across different types of gene duplicate pairs.** Whole-genome duplicates - WGD, Single-gene duplicates - SGD (combined data from tandem, proximal, translocated, and dispersed duplicates). Center line in the boxplot represents the median of Ka/Ks ratios, while the box limits represent 25% and 75% percentile of the interquartile range, whiskers represent 1.5 times above or below the interquartile range and dots represents outliers.

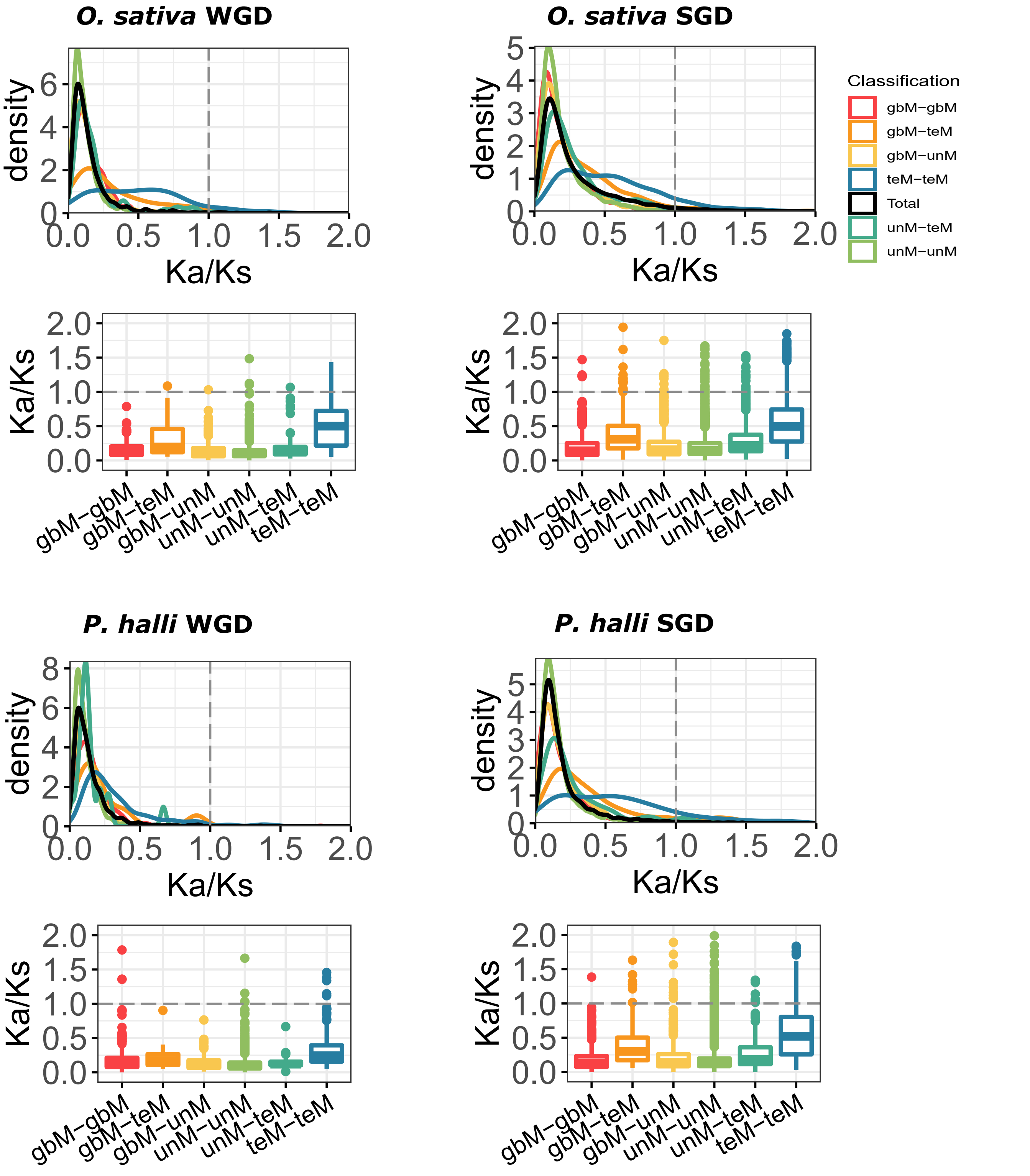

Supplemental Figure S10: **Distribution of genic methylation classified genes based on the ratio of nonsynonymous substitution (Ka), with synonymous substitutions (Ks) across different types of gene duplicate pairs.** Whole-genome duplicates - WGD, Single-gene duplicates - SGD (combined data from tandem, proximal, translocated, and dispersed duplicates). Center line in the boxplot represents the median of Ka/Ks ratios, while the box limits represent 25% and 75% percentile of the interquartile range, whiskers represent 1.5 times above or below the interquartile range and dots represents outliers.

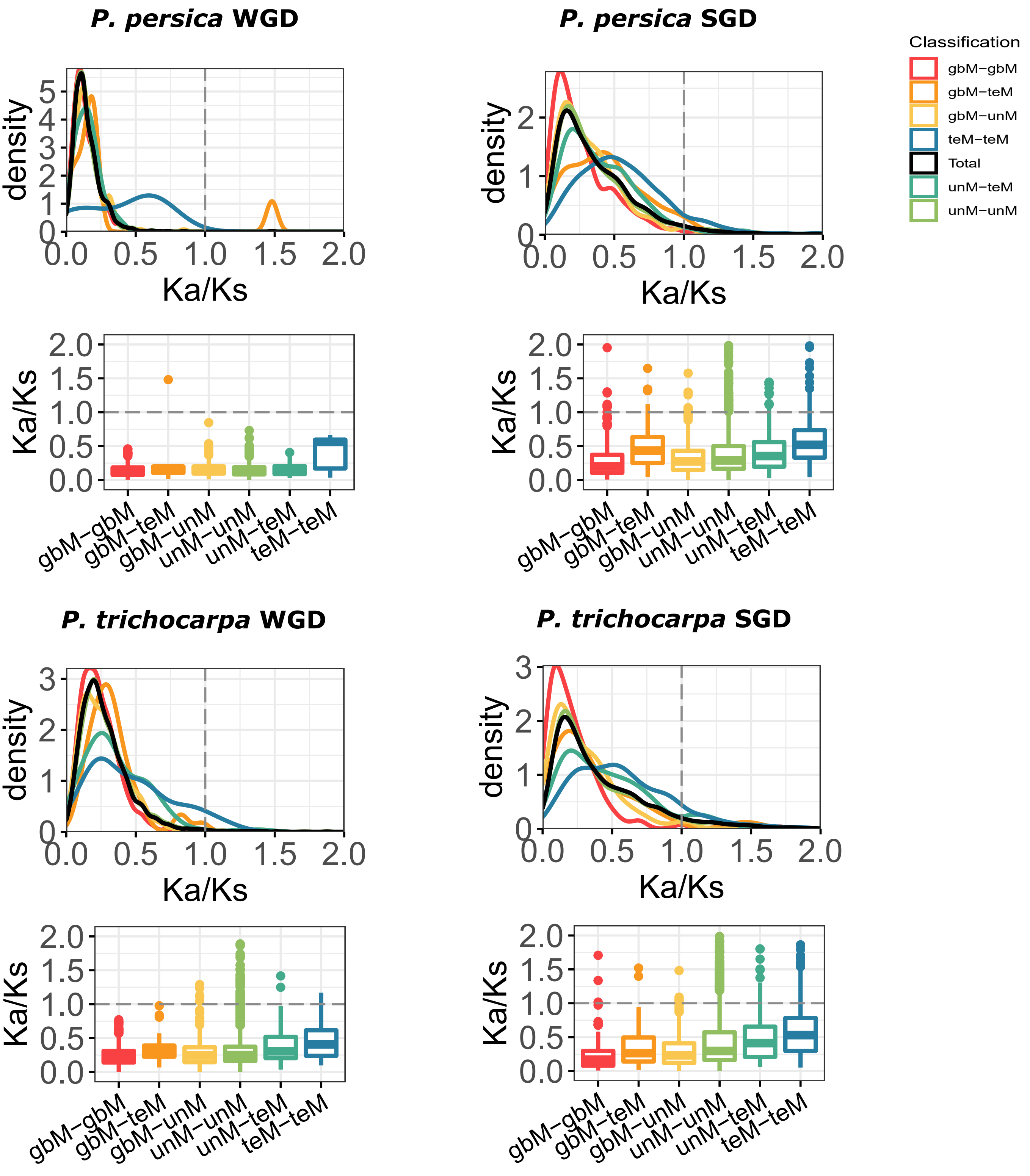

Supplemental Figure S10: **Distribution of genic methylation classified genes based on the ratio of nonsynonymous substitution (Ka), with synonymous substitutions (Ks) across different types of gene duplicate pairs.** Whole-genome duplicates - WGD, Single-gene duplicates - SGD (combined data from tandem, proximal, translocated, and dispersed duplicates). Center line in the boxplot represents the median of Ka/Ks ratios, while the box limits represent 25% and 75% percentile of the interquartile range, whiskers represent 1.5 times above or below the interquartile range and dots represents outliers.

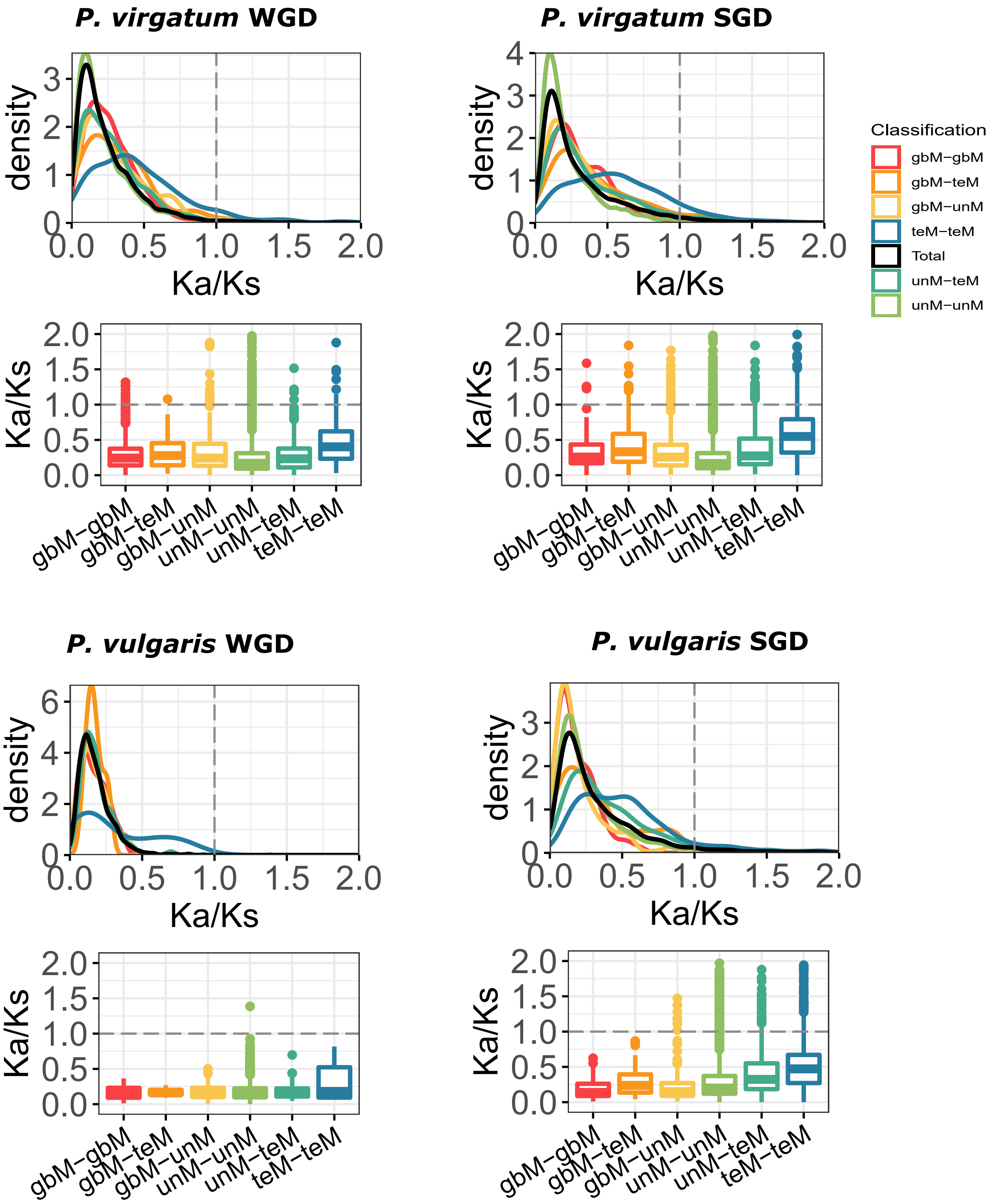

Supplemental Figure S10: **Distribution of genic methylation classified genes based on the ratio of nonsynonymous substitution (Ka), with synonymous substitutions (Ks) across different types of gene duplicate pairs.** Whole-genome duplicates - WGD, Single-gene duplicates - SGD (combined data from tandem, proximal, translocated, and dispersed duplicates). Center line in the boxplot represents the median of Ka/Ks ratios, while the box limits represent 25% and 75% percentile of the interquartile range, whiskers represent 1.5 times above or below the interquartile range and dots represents outliers.

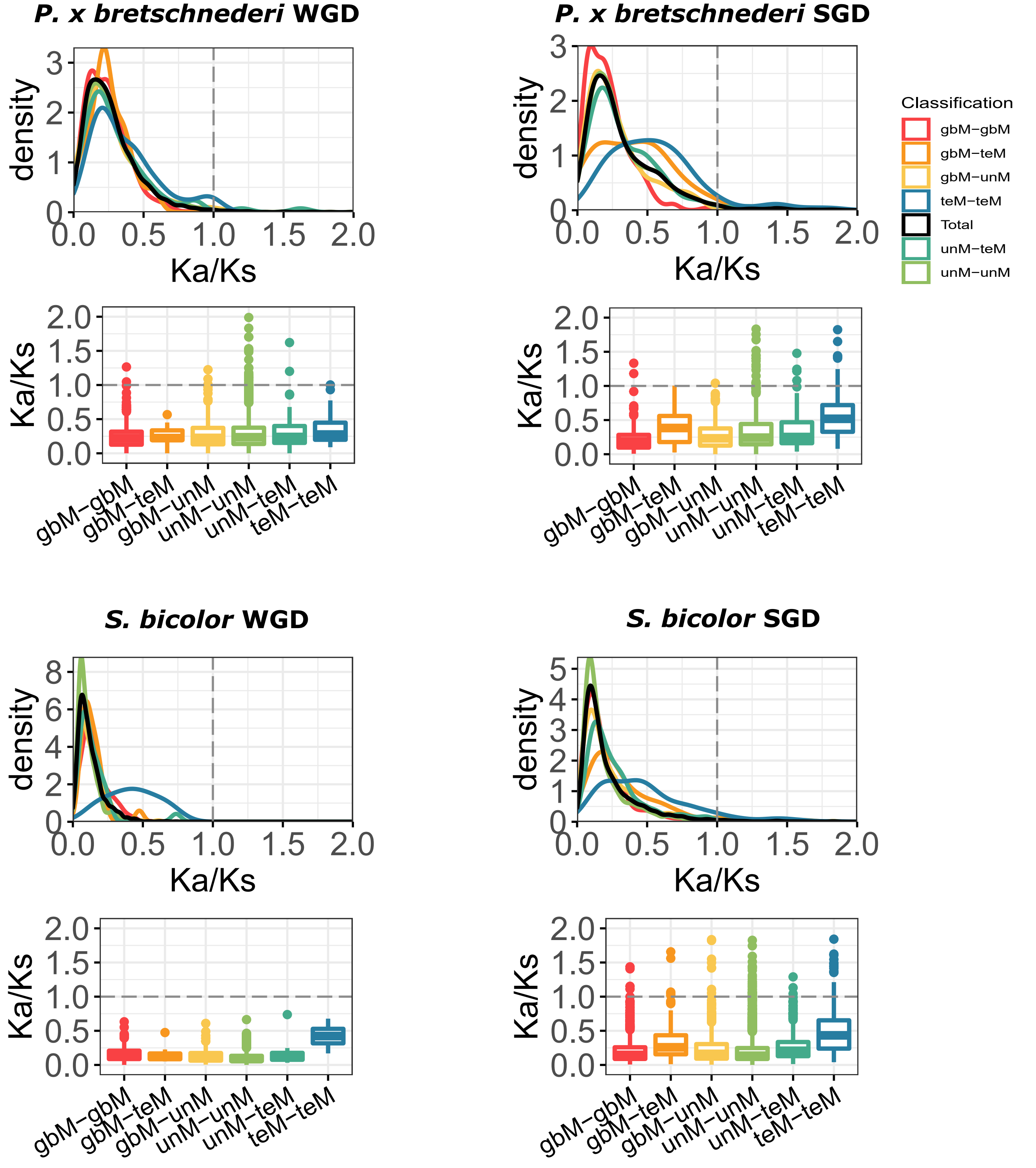

Supplemental Figure S10: **Distribution of genic methylation classified genes based on the ratio of nonsynonymous substitution (Ka), with synonymous substitutions (Ks) across different types of gene duplicate pairs.** Whole-genome duplicates - WGD, Single-gene duplicates - SGD (combined data from tandem, proximal, translocated, and dispersed duplicates). Center line in the boxplot represents the median of Ka/Ks ratios, while the box limits represent 25% and 75% percentile of the interquartile range, whiskers represent 1.5 times above or below the interquartile range and dots represents outliers.

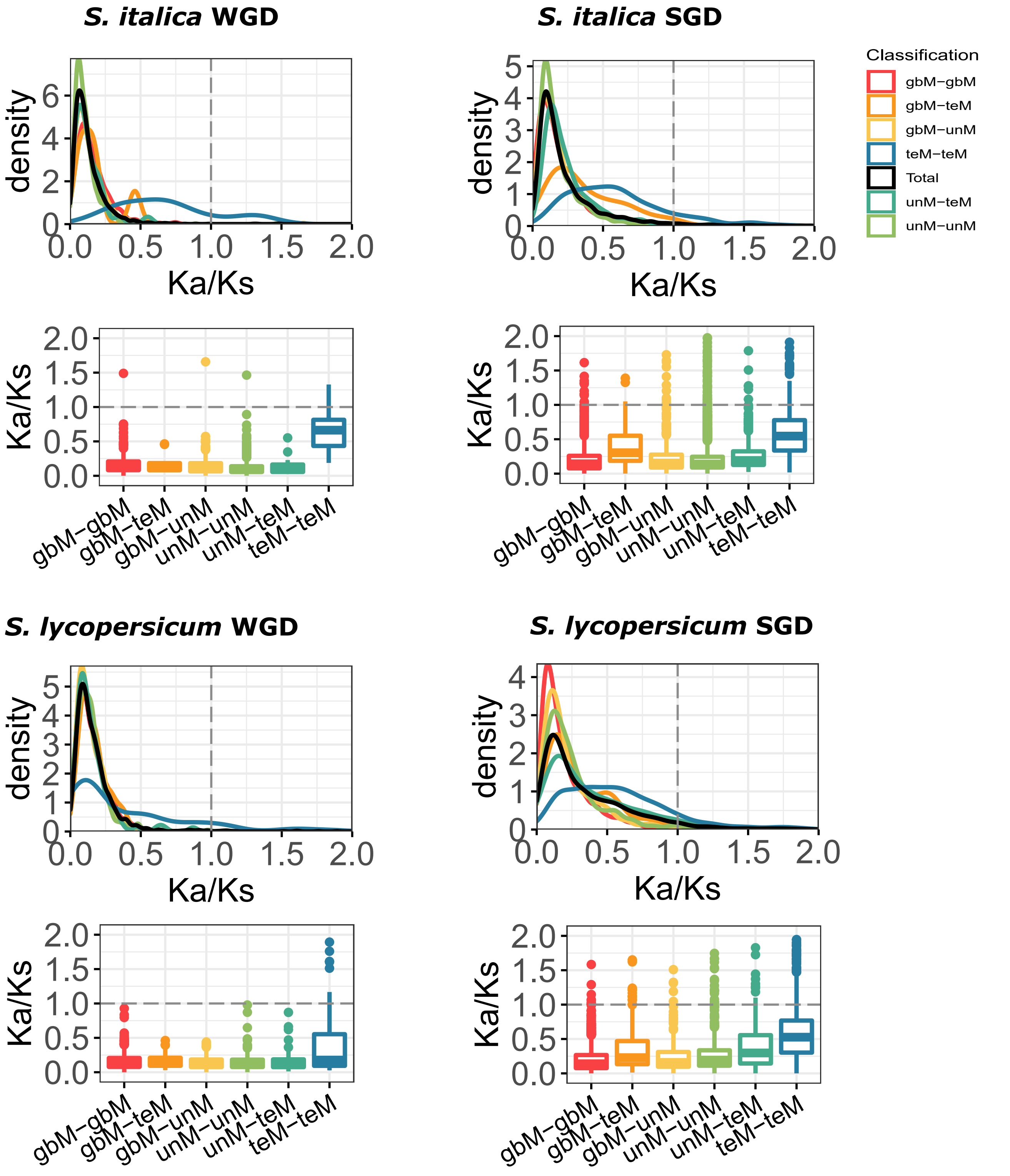

Supplemental Figure S10: **Distribution of genic methylation classified genes based on the ratio of nonsynonymous substitution (Ka), with synonymous substitutions (Ks) across different types of gene duplicate pairs.** Whole-genome duplicates - WGD, Single-gene duplicates - SGD (combined data from tandem, proximal, translocated, and dispersed duplicates). Center line in the boxplot represents the median of Ka/Ks ratios, while the box limits represent 25% and 75% percentile of the interquartile range, whiskers represent 1.5 times above or below the interquartile range and dots represents outliers.

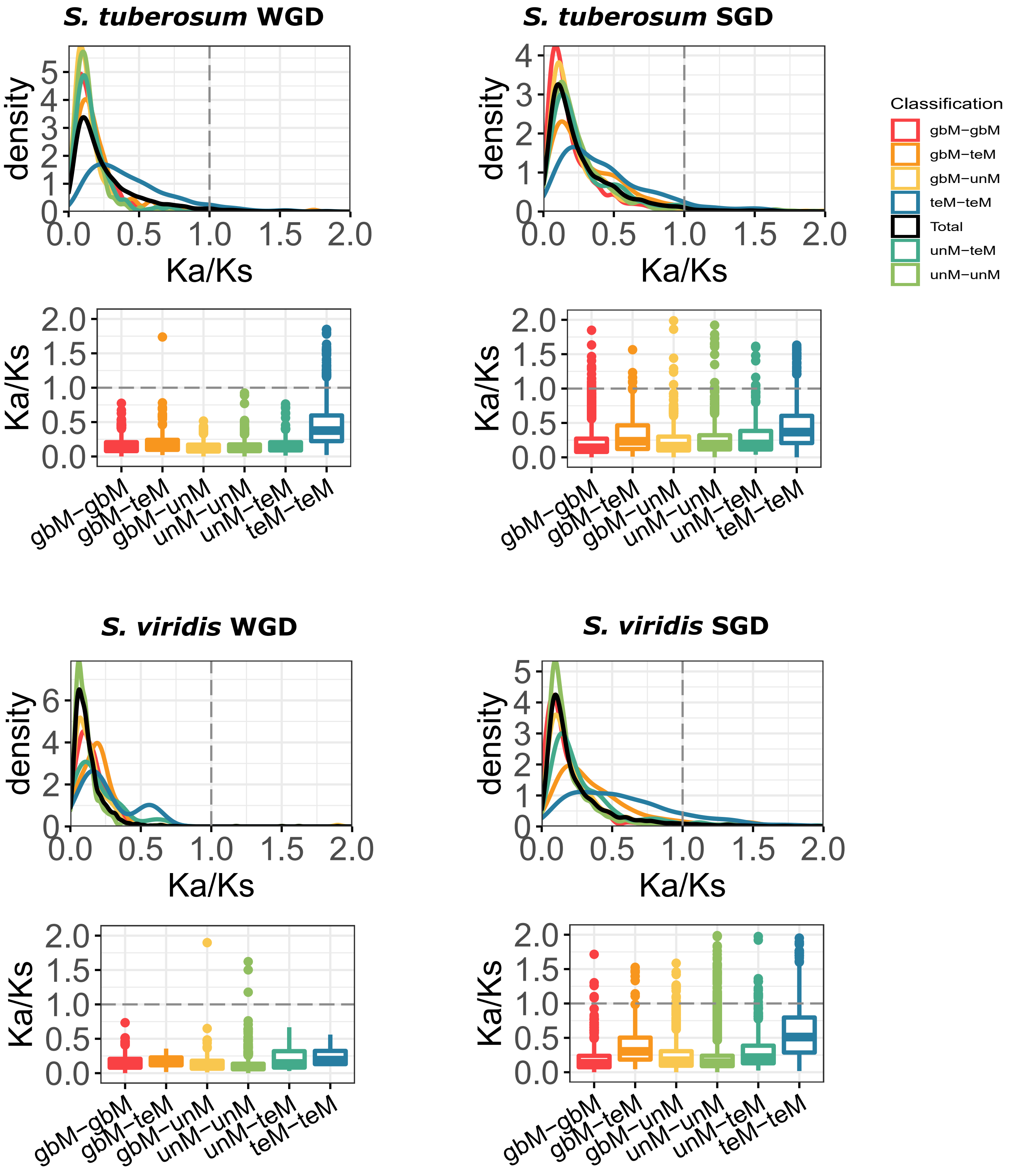

Supplemental Figure S10: **Distribution of genic methylation classified genes based on the ratio of nonsynonymous substitution (Ka), with synonymous substitutions (Ks) across different types of gene duplicate pairs.** Whole-genome duplicates - WGD, Single-gene duplicates - SGD (combined data from tandem, proximal, translocated, and dispersed duplicates). Center line in the boxplot represents the median of Ka/Ks ratios, while the box limits represent 25% and 75% percentile of the interquartile range, whiskers represent 1.5 times above or below the interquartile range and dots represents outliers.

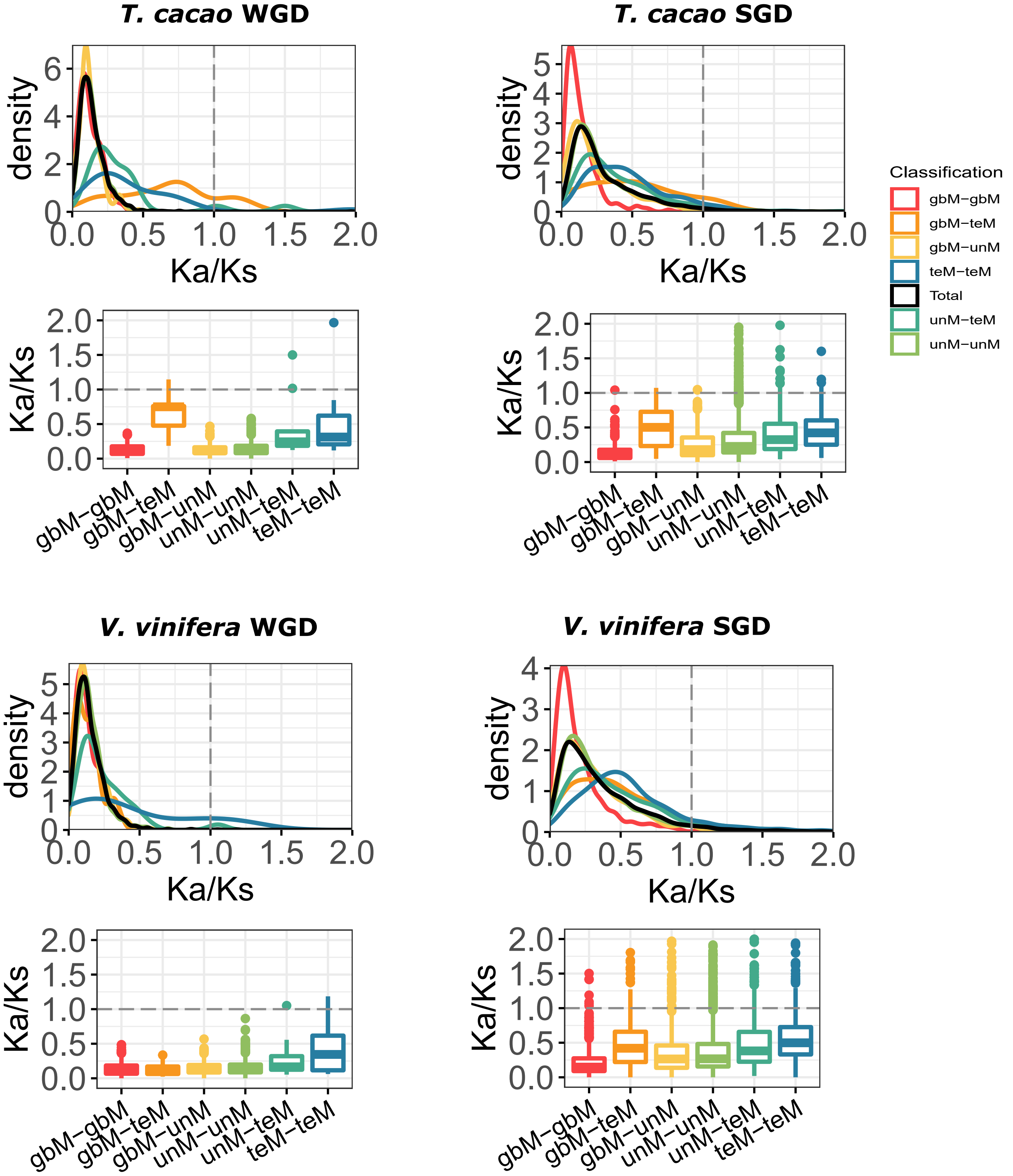

Supplemental Figure S10: **Distribution of genic methylation classified genes based on the ratio of nonsynonymous substitution (Ka), with synonymous substitutions (Ks) across different types of gene duplicate pairs.** Whole-genome duplicates - WGD, Single-gene duplicates - SGD (combined data from tandem, proximal, translocated, and dispersed duplicates). Center line in the boxplot represents the median of Ka/Ks ratios, while the box limits represent 25% and 75% percentile of the interquartile range, whiskers represent 1.5 times above or below the interquartile range and dots represents outliers.

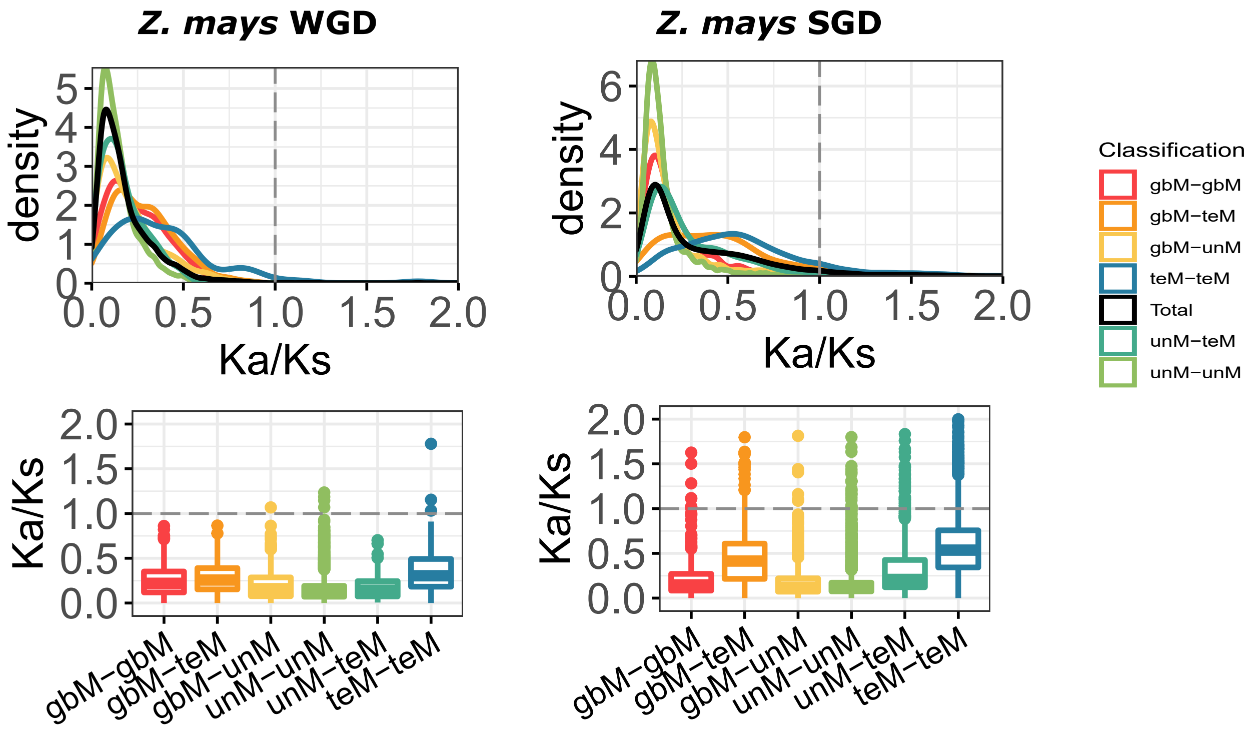

Supplemental Figure S11: **Distribution of core: multi-copy and core: single-copy (intermediate) paralogs based on the ratio of nonsynonymous substitution (Ka), with synonymous substitutions (Ks).** 'All pairs' represent Ka/Ks ratios of all duplicate gene pairs in the genome, 'Core-MC' represents duplicate pairs among core: multi-copy orthogroup, while 'SC-Int' represent duplicate pairs among the core: single-copy orthogroups. Center line in the boxplot represents the median of Ka/Ks ratios, while the box limits represent 25% and 75% percentile of the interquartile range, whiskers represent 1.5 times above or below the interquartile range and dots represents outliers.

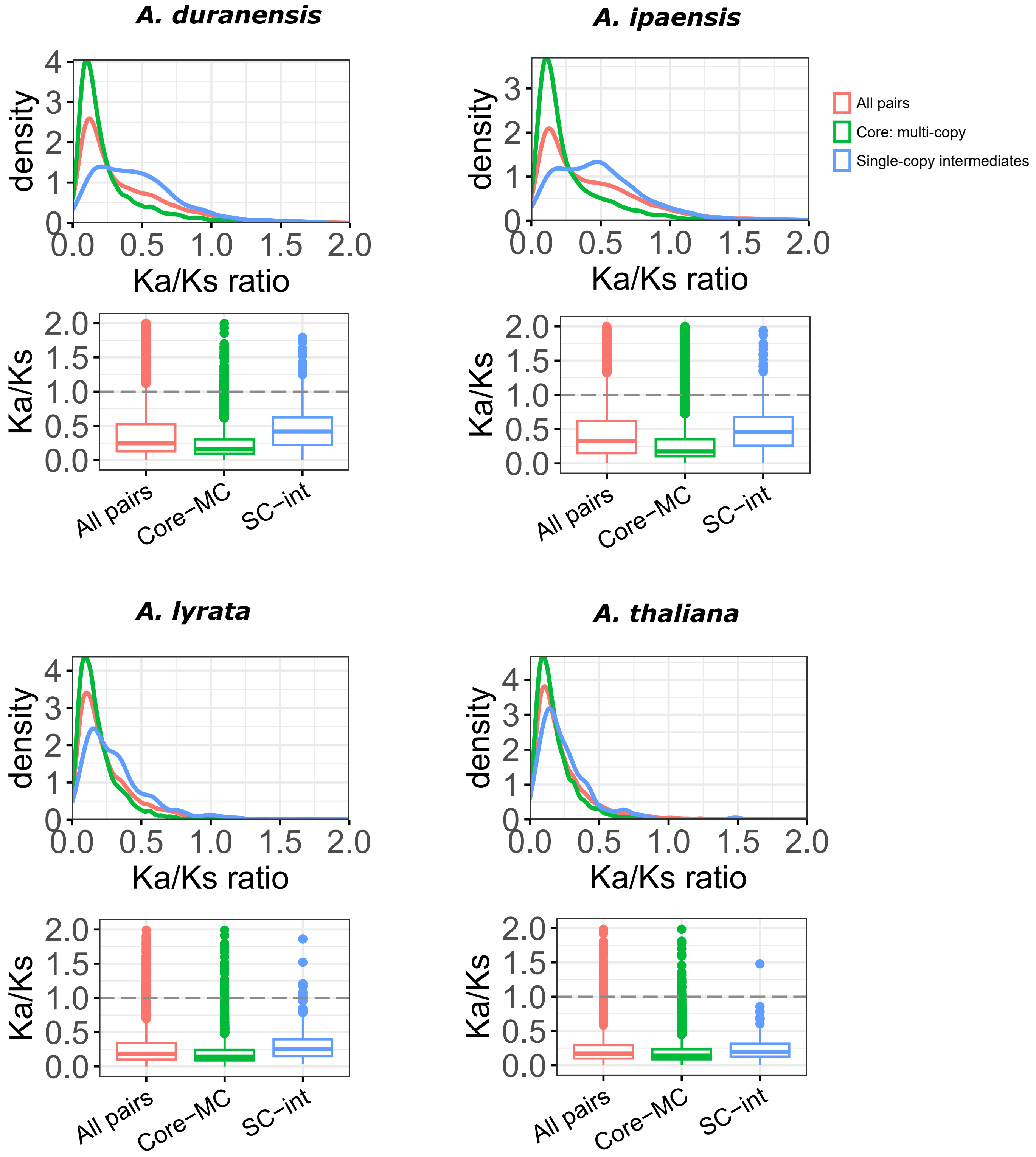

Supplemental Figure S11: Distribution of core: multi-copy and core: single-copy (intermediate) paralogs based on the ratio of nonsynonymous substitution (Ka), with synonymous substitutions (Ks). 'All pairs' represent Ka/Ks ratios of all duplicate gene pairs in the genome, 'Core-MC' represents duplicate pairs among core: multi-copy orthogroup, while 'SC-Int' represent duplicate pairs among the core: single-copy orthogroups. Center line in the boxplot represents the median of Ka/Ks ratios, while the box limits represent 25% and 75% percentile of the interquartile range, whiskers represent 1.5 times above or below the interquartile range and dots represents outliers.

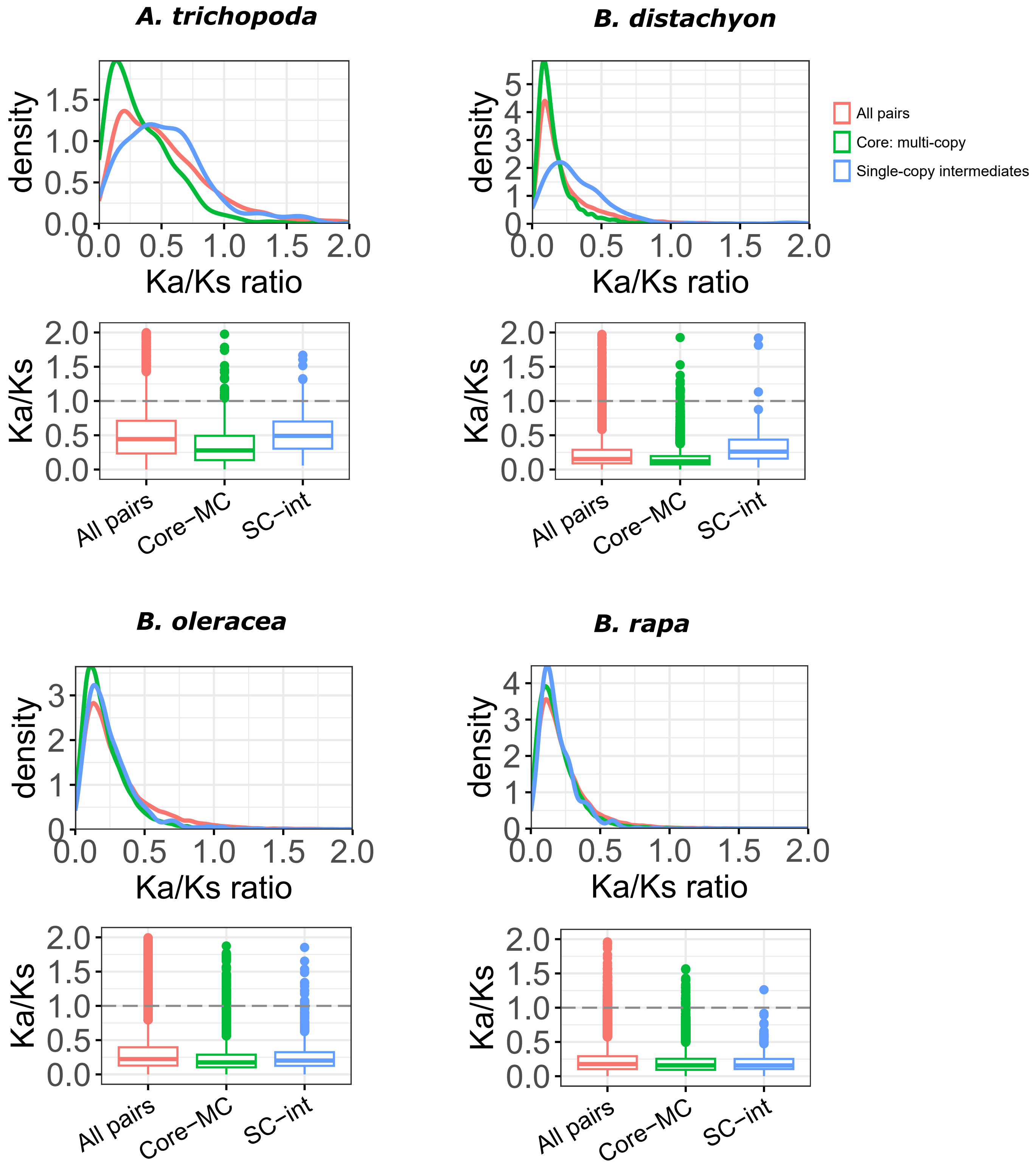

Supplemental Figure S11: **Distribution of core: multi-copy and core: single-copy (intermediate) paralogs based on the ratio of nonsynonymous substitution (Ka), with synonymous substitutions (Ks).** 'All pairs' represent Ka/Ks ratios of all duplicate gene pairs in the genome, 'Core-MC' represents duplicate pairs among core: multi-copy orthogroup, while 'SC-Int' represent duplicate pairs among the core: single-copy orthogroups. Center line in the boxplot represents the median of Ka/Ks ratios, while the box limits represent 25% and 75% percentile of the interquartile range, whiskers represent 1.5 times above or below the interquartile range and dots represents outliers.

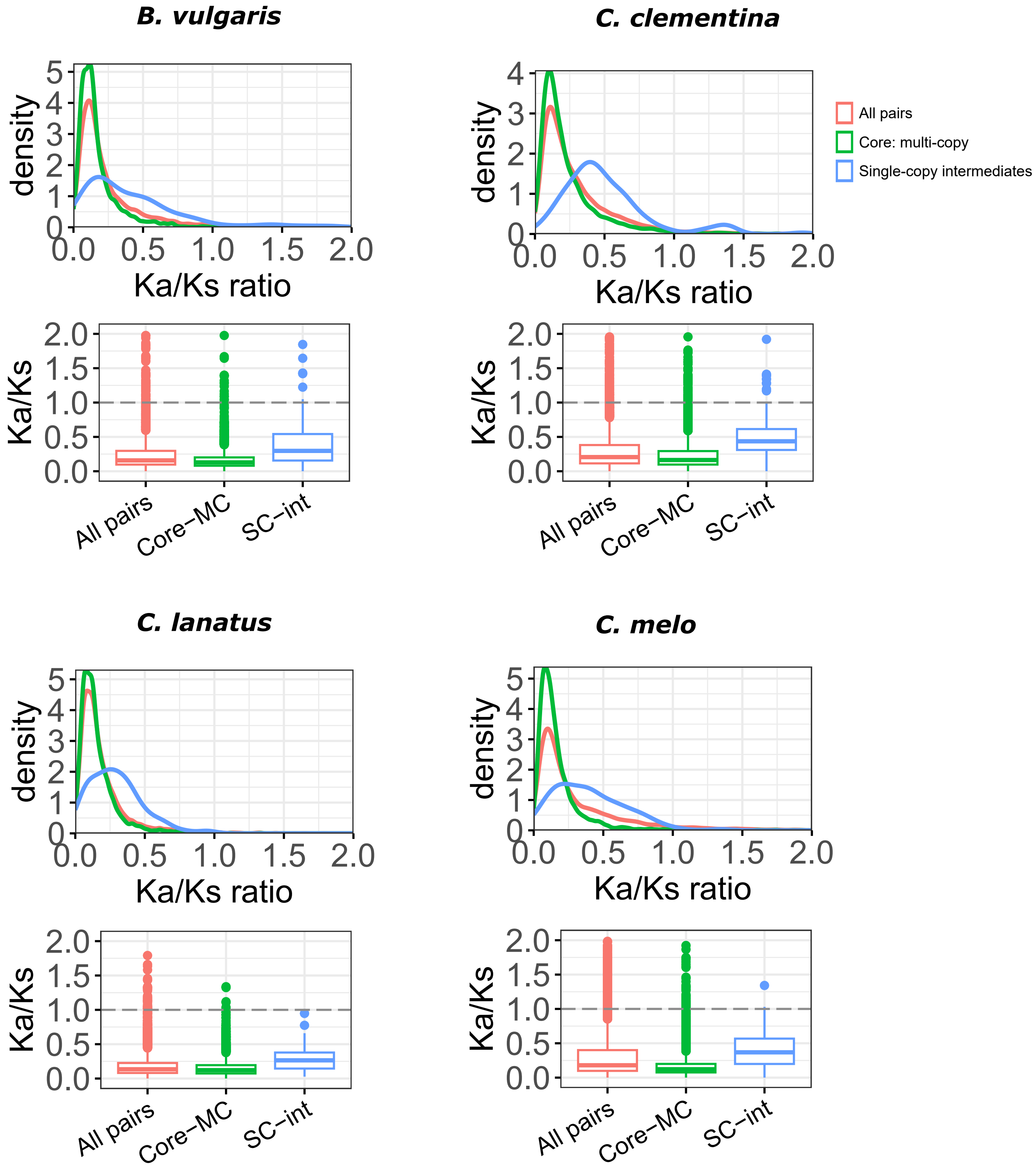

Supplemental Figure S11: **Distribution of core: multi-copy and core: single-copy (intermediate) paralogs based on the ratio of nonsynonymous substitution (Ka), with synonymous substitutions (Ks).** 'All pairs' represent Ka/Ks ratios of all duplicate gene pairs in the genome, 'Core-MC' represents duplicate pairs among core: multi-copy orthogroup, while 'SC-Int' represent duplicate pairs among the core: single-copy orthogroups. Center line in the boxplot represents the median of Ka/Ks ratios, while the box limits represent 25% and 75% percentile of the interquartile range, whiskers represent 1.5 times above or below the interquartile range and dots represents outliers.

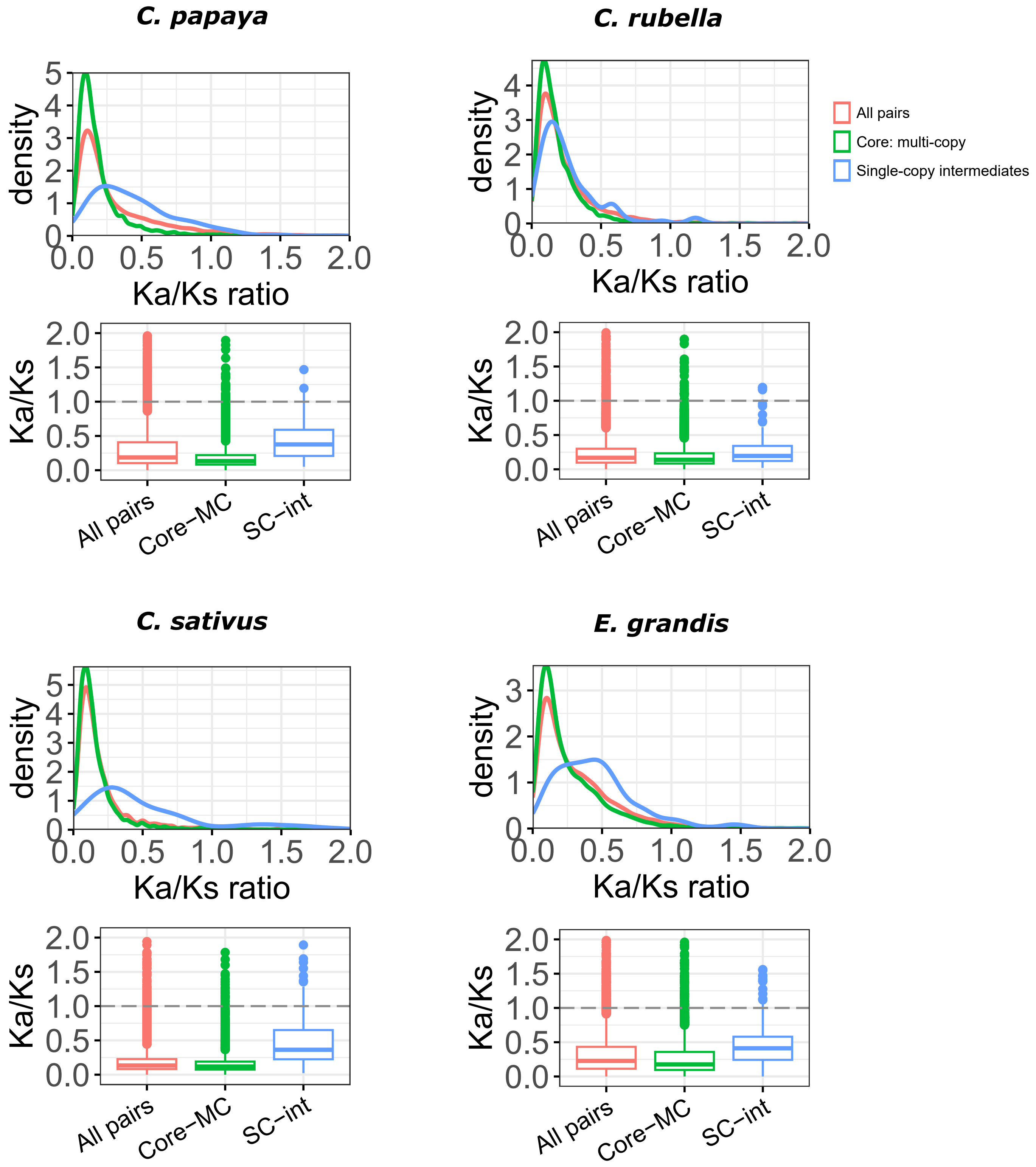

Supplemental Figure S11: **Distribution of core: multi-copy and core: single-copy (intermediate) paralogs based on the ratio of nonsynonymous substitution (Ka), with synonymous substitutions (Ks).** 'All pairs' represent Ka/Ks ratios of all duplicate gene pairs in the genome, 'Core-MC' represents duplicate pairs among core: multi-copy orthogroup, while 'SC-Int' represent duplicate pairs among the core: single-copy orthogroups. Center line in the boxplot represents the median of Ka/Ks ratios, while the box limits represent 25% and 75% percentile of the interquartile range, whiskers represent 1.5 times above or below the interquartile range and dots represents outliers.

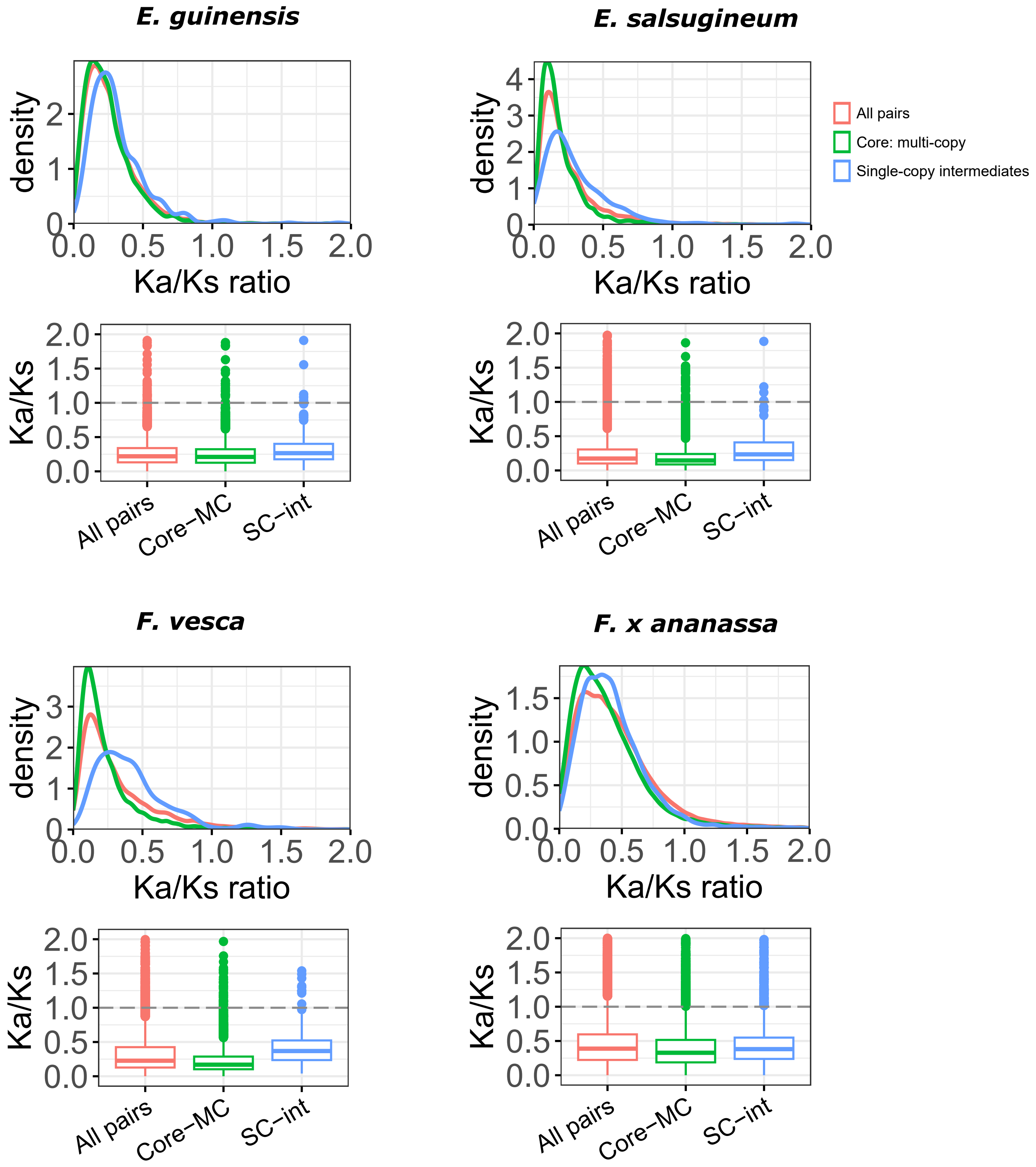

Supplemental Figure S11: **Distribution of core: multi-copy and core: single-copy (intermediate) paralogs based on the ratio of nonsynonymous substitution (Ka), with synonymous substitutions (Ks).** 'All pairs' represent Ka/Ks ratios of all duplicate gene pairs in the genome, 'Core-MC' represents duplicate pairs among core: multi-copy orthogroup, while 'SC-Int' represent duplicate pairs among the core: single-copy orthogroups. Center line in the boxplot represents the median of Ka/Ks ratios, while the box limits represent 25% and 75% percentile of the interquartile range, whiskers represent 1.5 times above or below the interquartile range and dots represents outliers.

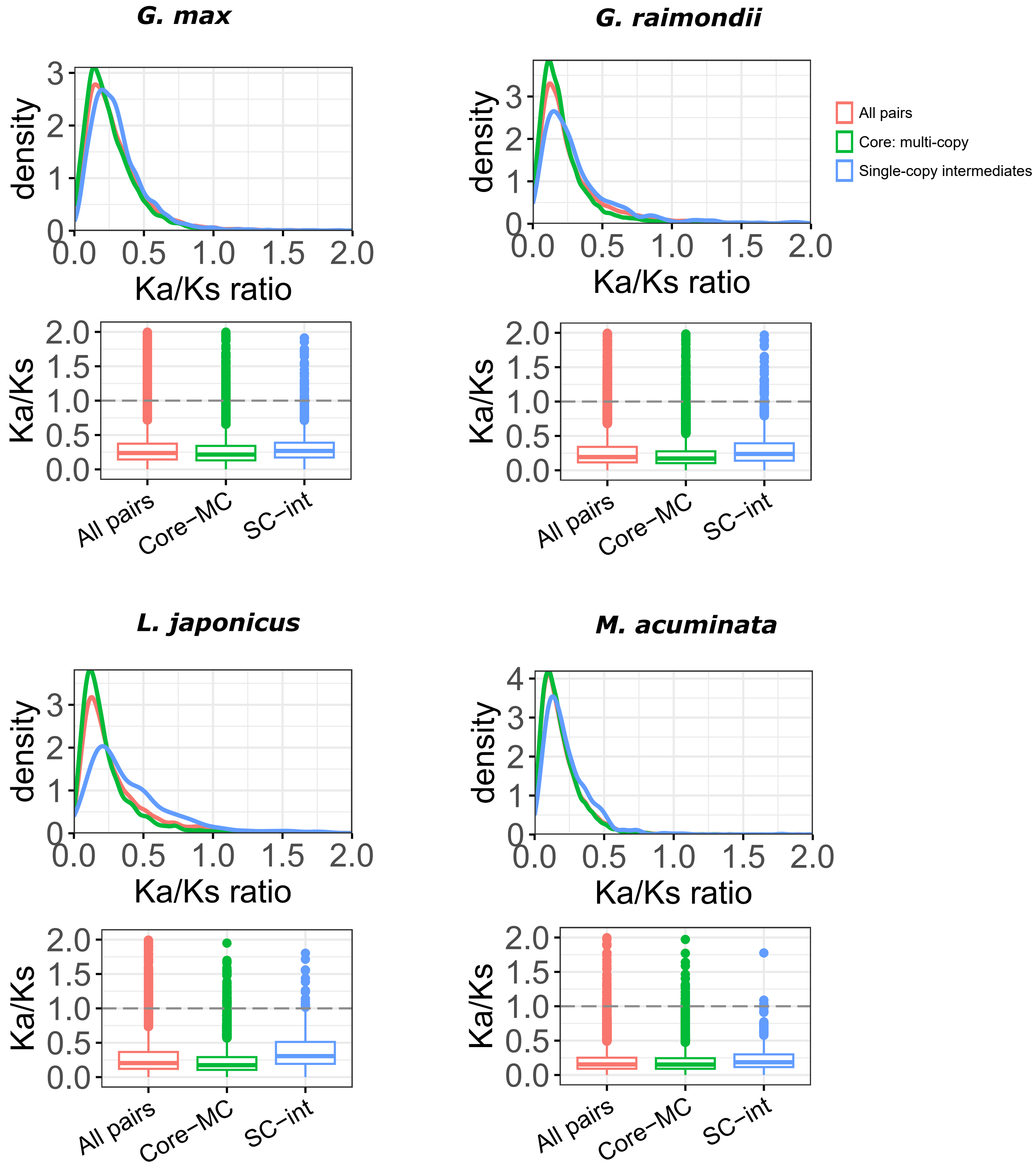

Supplemental Figure S11: **Distribution of core: multi-copy and core: single-copy (intermediate) paralogs based on the ratio of nonsynonymous substitution (Ka), with synonymous substitutions (Ks).** 'All pairs' represent Ka/Ks ratios of all duplicate gene pairs in the genome, 'Core-MC' represents duplicate pairs among core: multi-copy orthogroup, while 'SC-Int' represent duplicate pairs among the core: single-copy orthogroups. Center line in the boxplot represents the median of Ka/Ks ratios, while the box limits represent 25% and 75% percentile of the interquartile range, whiskers represent 1.5 times above or below the interquartile range and dots represents outliers.

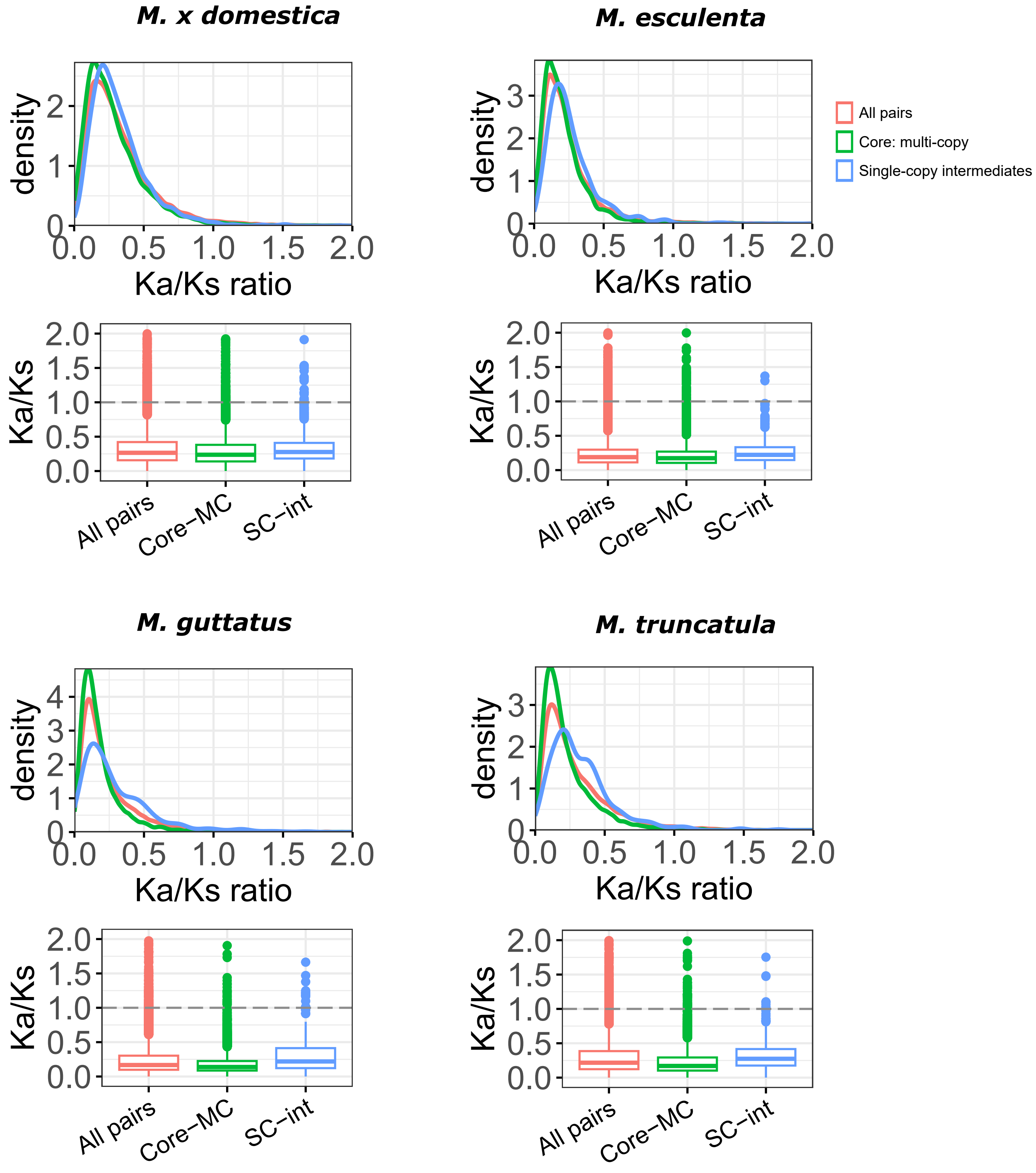

Supplemental Figure S11: **Distribution of core: multi-copy and core: single-copy (intermediate) paralogs based on the ratio of nonsynonymous substitution (Ka), with synonymous substitutions (Ks).** 'All pairs' represent Ka/Ks ratios of all duplicate gene pairs in the genome, 'Core-MC' represents duplicate pairs among core: multi-copy orthogroup, while 'SC-Int' represent duplicate pairs among the core: single-copy orthogroups. Center line in the boxplot represents the median of Ka/Ks ratios, while the box limits represent 25% and 75% percentile of the interquartile range, whiskers represent 1.5 times above or below the interquartile range and dots represents outliers.

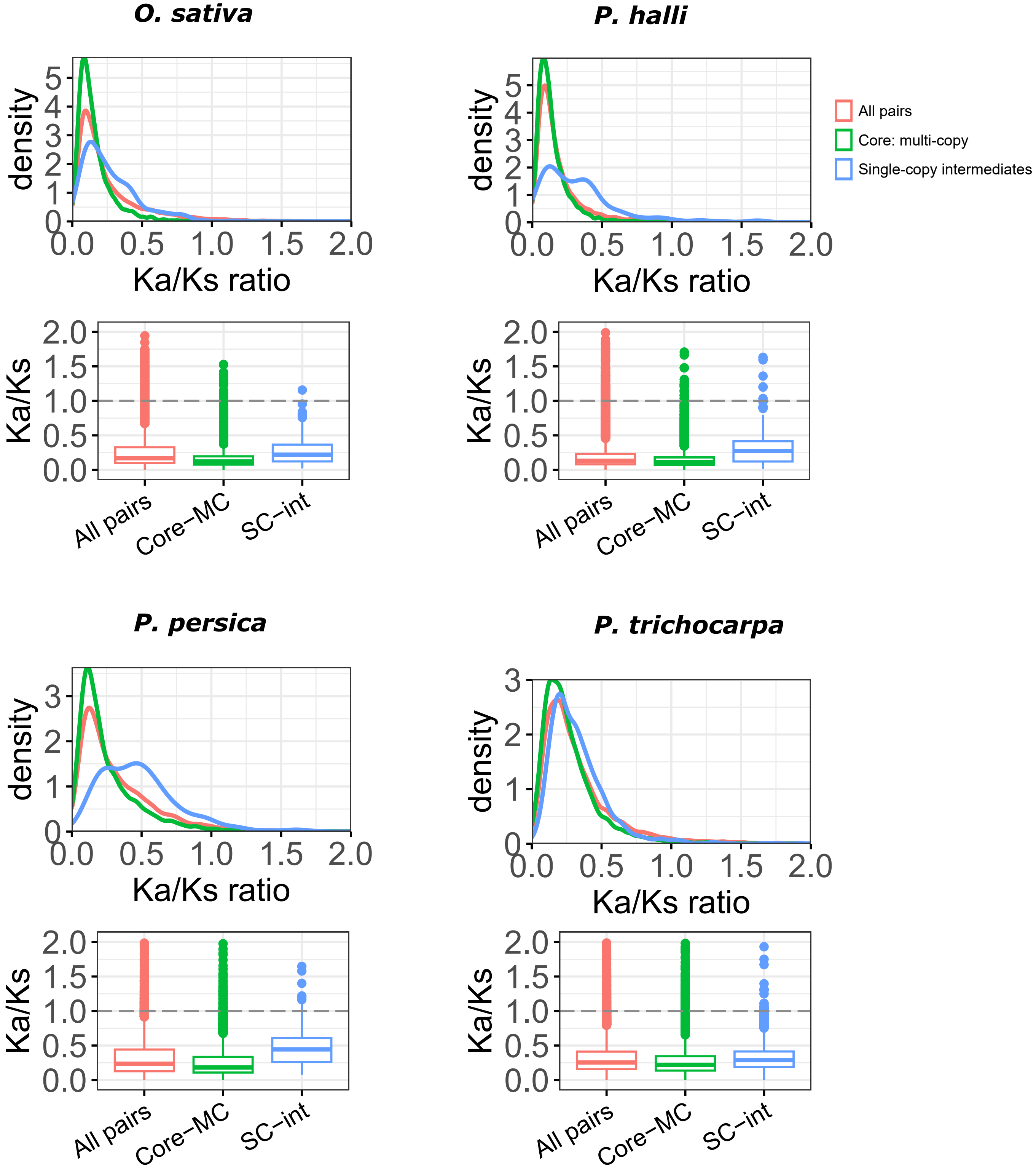

Supplemental Figure S11: **Distribution of core: multi-copy and core: single-copy (intermediate) paralogs based on the ratio of nonsynonymous substitution (Ka), with synonymous substitutions (Ks).** 'All pairs' represent Ka/Ks ratios of all duplicate gene pairs in the genome, 'Core-MC' represents duplicate pairs among core: multi-copy orthogroup, while 'SC-Int' represent duplicate pairs among the core: single-copy orthogroups. Center line in the boxplot represents the median of Ka/Ks ratios, while the box limits represent 25% and 75% percentile of the interquartile range, whiskers represent 1.5 times above or below the interquartile range and dots represents outliers.

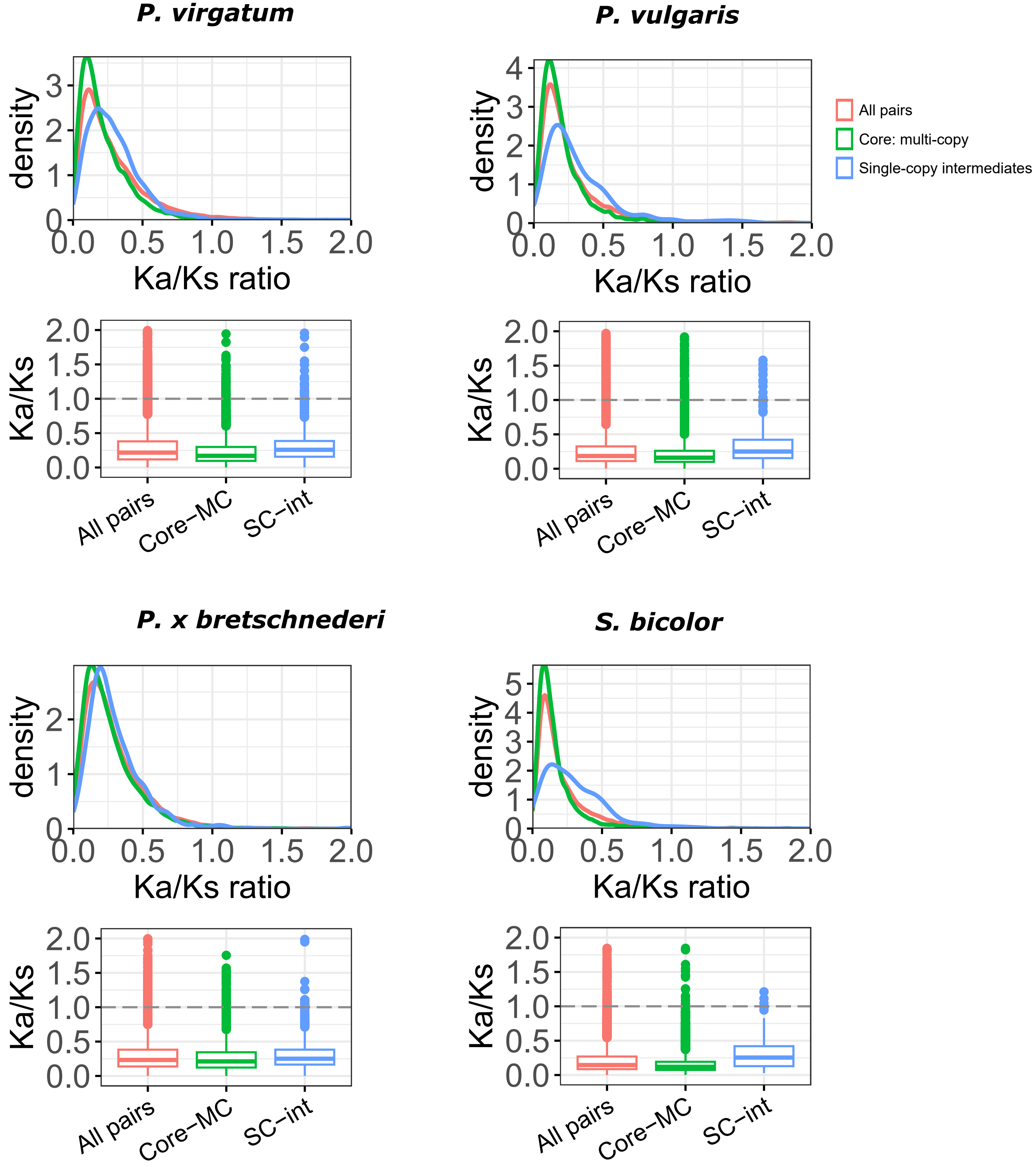

Supplemental Figure S11: **Distribution of core: multi-copy and core: single-copy (intermediate) paralogs based on the ratio of nonsynonymous substitution (Ka), with synonymous substitutions (Ks).** 'All pairs' represent Ka/Ks ratios of all duplicate gene pairs in the genome, 'Core-MC' represents duplicate pairs among core: multi-copy orthogroup, while 'SC-Int' represent duplicate pairs among the core: single-copy orthogroups. Center line in the boxplot represents the median of Ka/Ks ratios, while the box limits represent 25% and 75% percentile of the interquartile range, whiskers represent 1.5 times above or below the interquartile range and dots represents outliers.

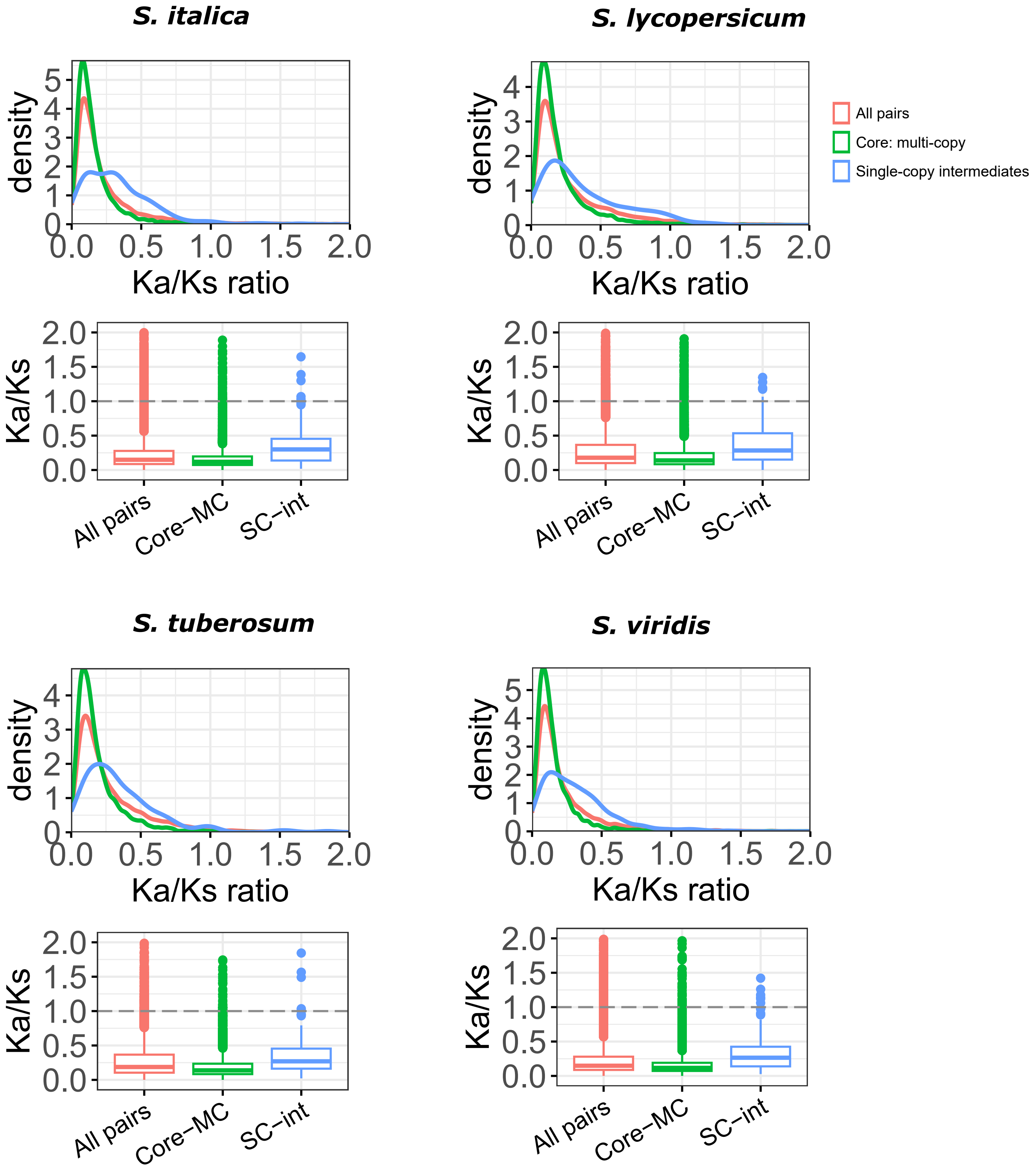

Supplemental Figure S11: **Distribution of core: multi-copy and core: single-copy (intermediate) paralogs based on the ratio of nonsynonymous substitution (Ka), with synonymous substitutions (Ks).** 'All pairs' represent Ka/Ks ratios of all duplicate gene pairs in the genome, 'Core-MC' represents duplicate pairs among core: multi-copy orthogroup, while 'SC-Int' represent duplicate pairs among the core: single-copy orthogroups. Center line in the boxplot represents the median of Ka/Ks ratios, while the box limits represent 25% and 75% percentile of the interquartile range, whiskers represent 1.5 times above or below the interquartile range and dots represents outliers.

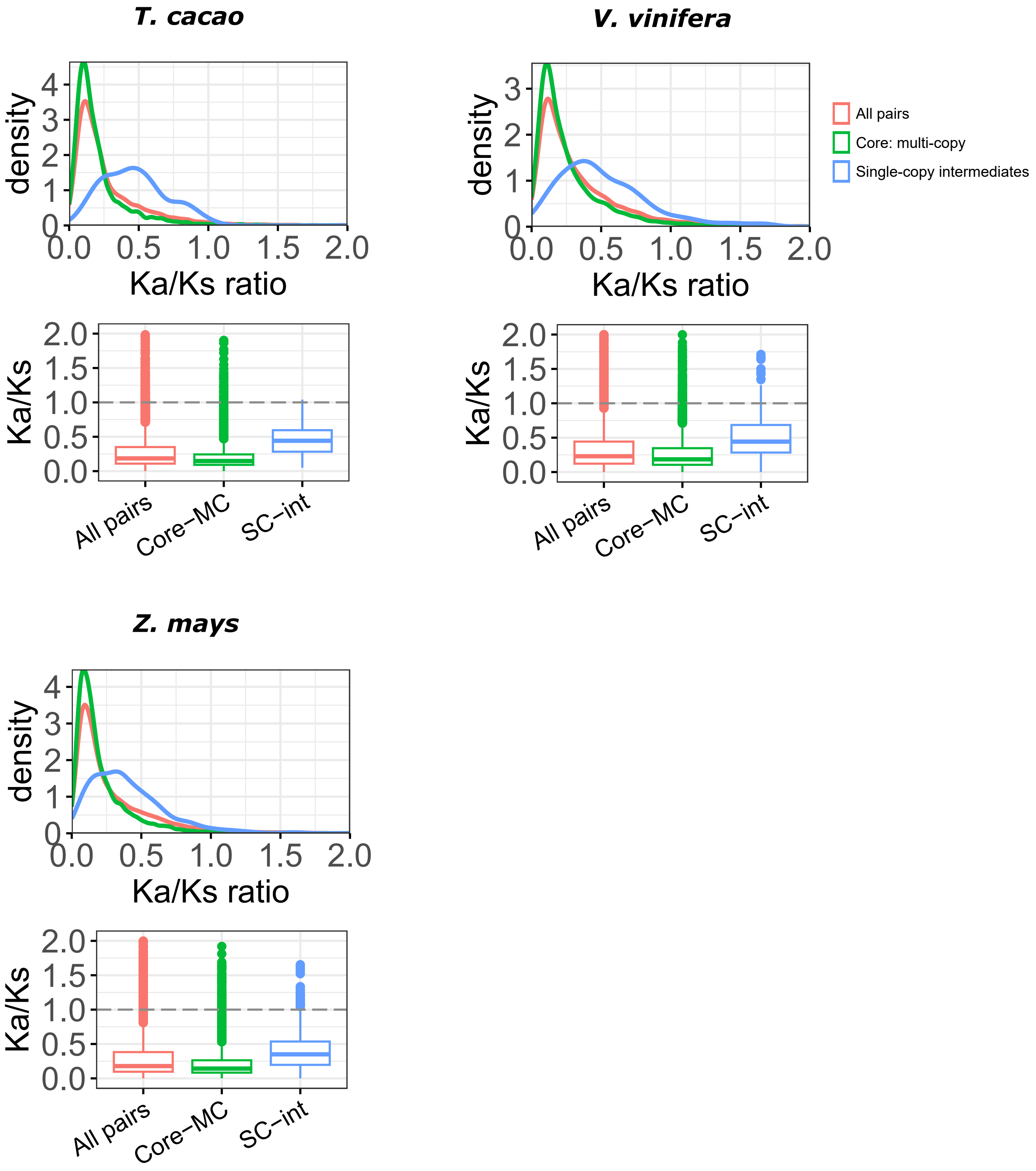

Supplemental Figure S12: Percentage of Total (all genes), gbM, teM, and unM genes with known presence-absence variation. This plot was not restricted to duplicate genes, however the same results were found when limited to duplicates (Table S17). A two-sided Fisher's Exact Test was used to test for depletion or enrichment of PAVs amongst each category of genic methylation. In *B. oleracae,* 151/1816 gbM, 1340/10909 teM, and 1990/29560 unM genes were PAV genes. In *S. lycopersicum,* 262/11387 gbM, 2289/6685 teM, and 1206/5959 unM genes were PAV genes. In *S. tuberosum*, 123/11708 gbM, 920/7551 teM, and 184/6236 unM genes were PAV genes. In *Z. mays,* 224/4760 gbM, 6019/9214 teM, and 2929/16651 unM genes were PAV genes. *FDR corrected p-value < 0.05, **FDR corrected p-value < 0.01, ***FDR corrected p-value < 0.001, NS – Not significantly different.

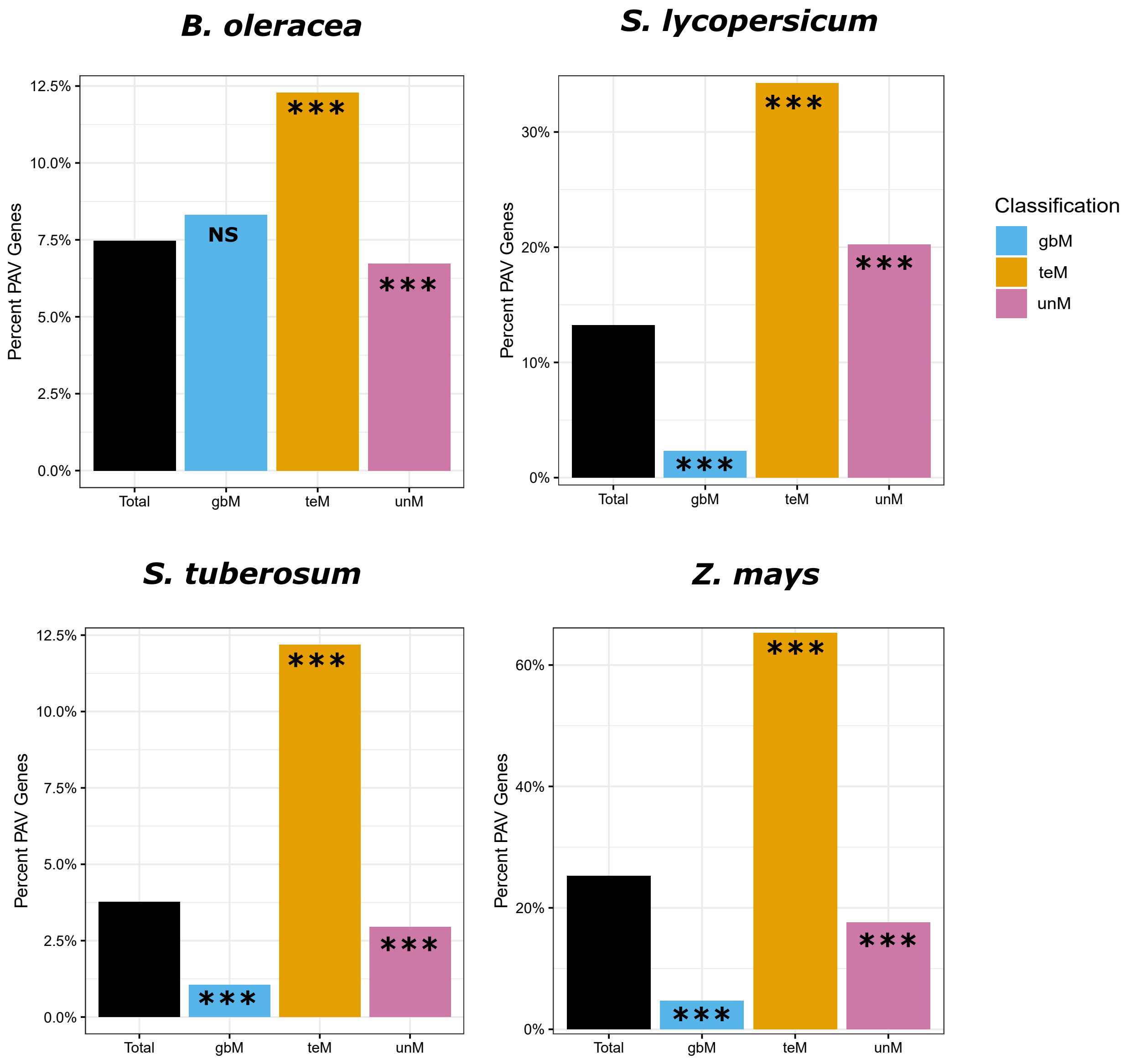

Supplemental Figure S13: Proportion of single-copy singletons and single-copy intermediates in PAV genes in *B. oleracae*, *S. lycopersicum*, *S. tuberosum*, and *Z. mays*. A proportion test was performed to test for differences in proportion of PAVs among single-copy singletons (SCs) and single-copy intermediates (SCi) compared to Core:multi-copy (Core-MC).
In *B. oleracae*, 994/24317 core-MC, 19/2227 SCs and 151/266 Sci were PAV genes.
In *S. lycopersicum*, 1200/15769 core-MC, 27/3131 SCs and 63/726 SCi were PAV genes.
In *S. tuberosum*, 1383/14893 core-MC, 7/2824 SCs and 33/578 SCi were PAV genes.
In *Z. mays*, 3068/18224 core-MC, 61/2692 SCs and 613/1923 SCi were PAV genes.
'***' represent statistical significant difference at p-value < 0.001,
NS – Not significantly different. Proportion of Core:MC, SCs,and SCi were signifcantly different from 'Total'.

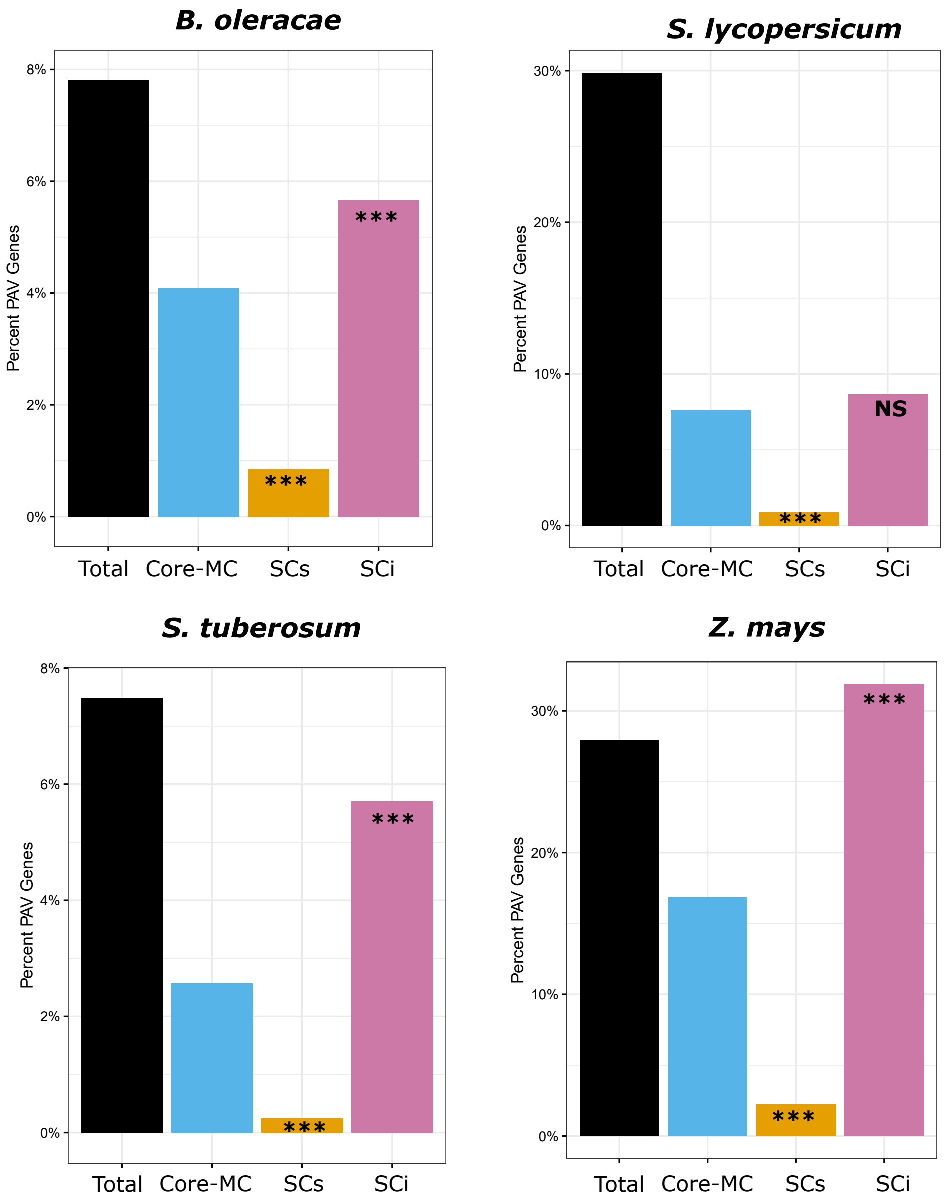

Supplemental Figure S14: **Tau specificity of gbM, teM, and unM genes in *G. max, P. vulgaris,* and *S. bicolor*.** Center line in the boxplot represents the median Tau values, while the box limits represent 25% and 75% percentile of the interquartile range, whiskers represent 1.5 times above or below the interquartile range.

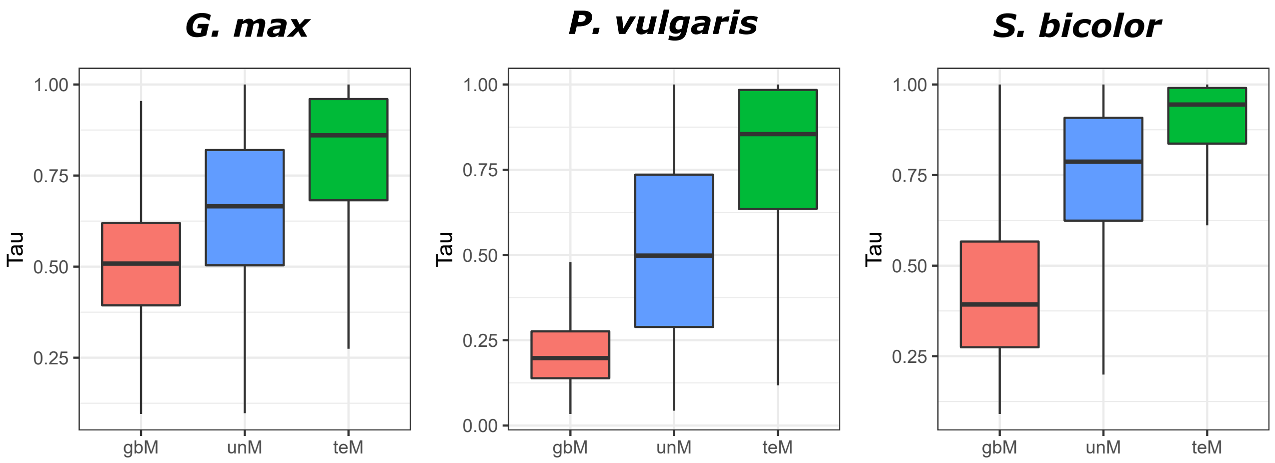

Supplemental Figure S15: **Tau specificity of gbM, teM, and unM genes each orthogroup category in *A. thaliana, G. max, P. vulgaris*, and *S. bicolor*.** Center line in the boxplot represents the median Tau, while the box limits represent 25% and 75% percentile of the interquartile range, whiskers represent 1.5 times above or below the interquartile range and dots represents outliers

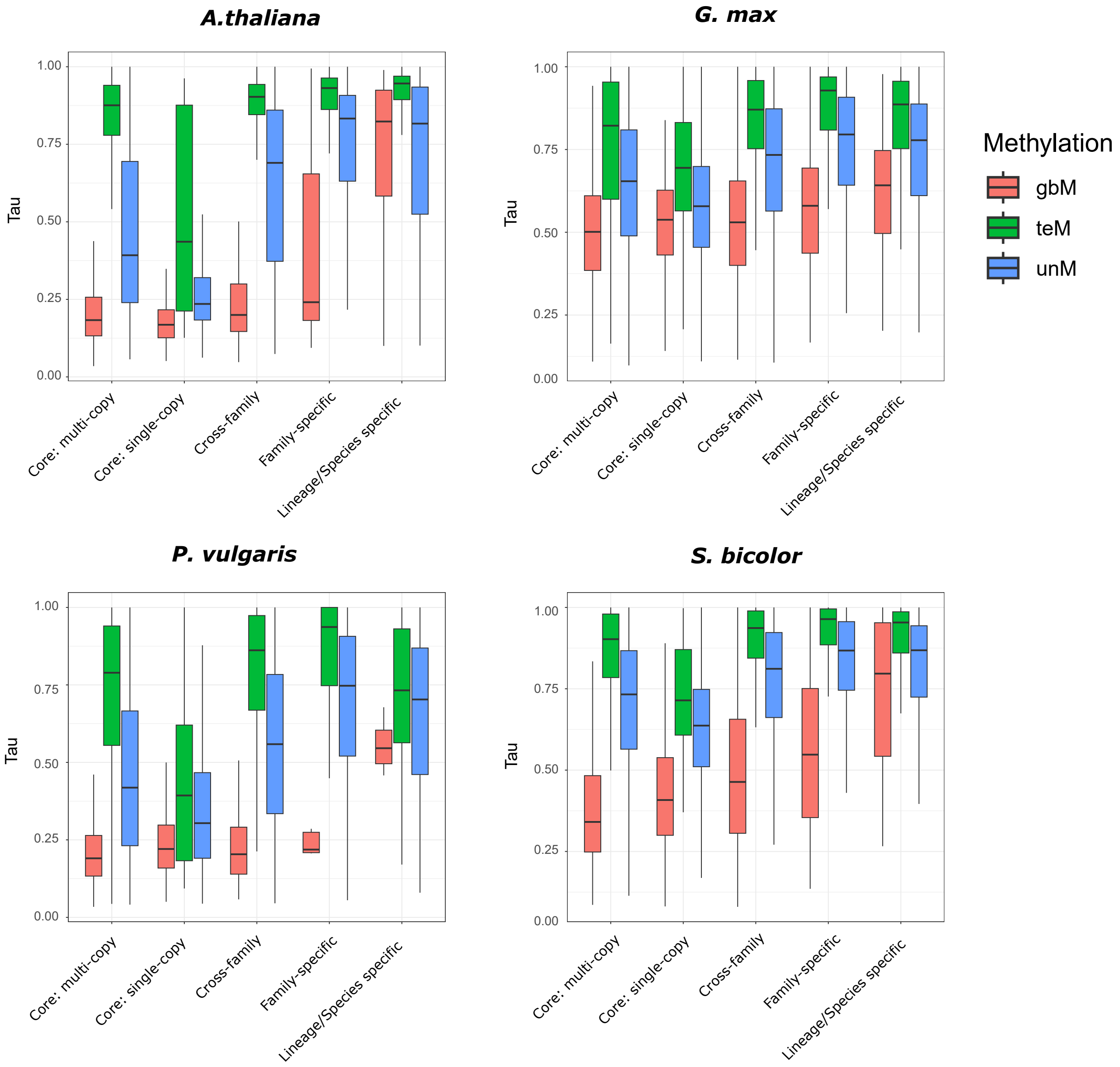

Supplemental Figure S16: **Tau specificities of different types of duplicate genes in *A. thaliana, G. max, P. vulgaris,* and *S. bicolor*.** The distribution of tau for gbM, unM, and teM genes is shown for all duplicates and also broken down based on the type of duplicate gene. Center line in the boxplot represents the median Tau, while the box limits represent 25% and 75% percentile of the interquartile range, whiskers represent 1.5 times above or below the interquartile range.

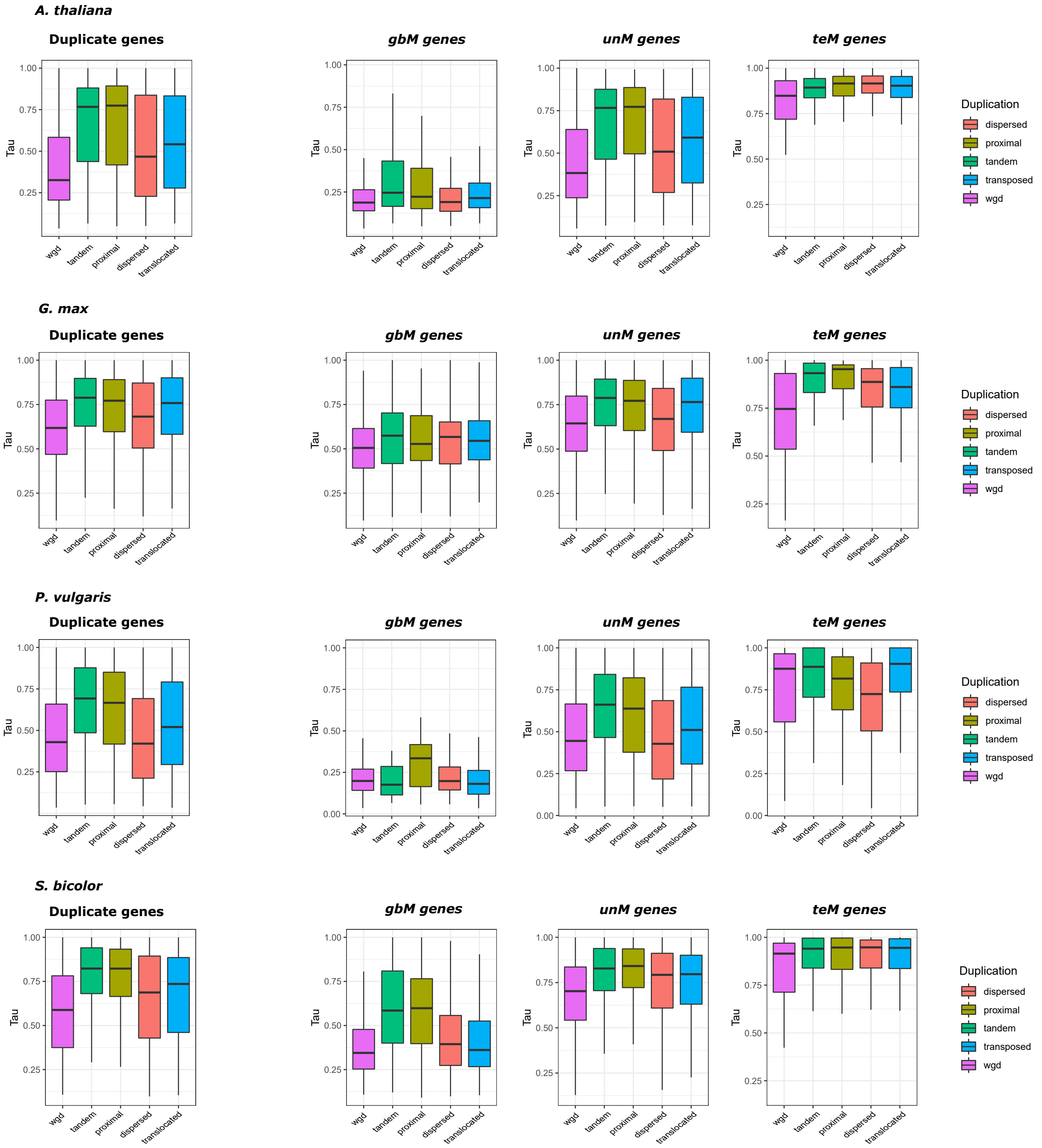

Supplemental Figure S17: Half plots showing gene expression correlations of duplicate pairs based on genic methylation (gbM-gbM, gbM-teM, teM-teM, unM-unM, gbM-unM, and unM-teM) in *A. thaliana, G. max, P. vulgaris,* and *S. bicolor.* Each dot represents gene expression correlation between duplicate genes and the violin halfplot represents the distribution of the correlations.

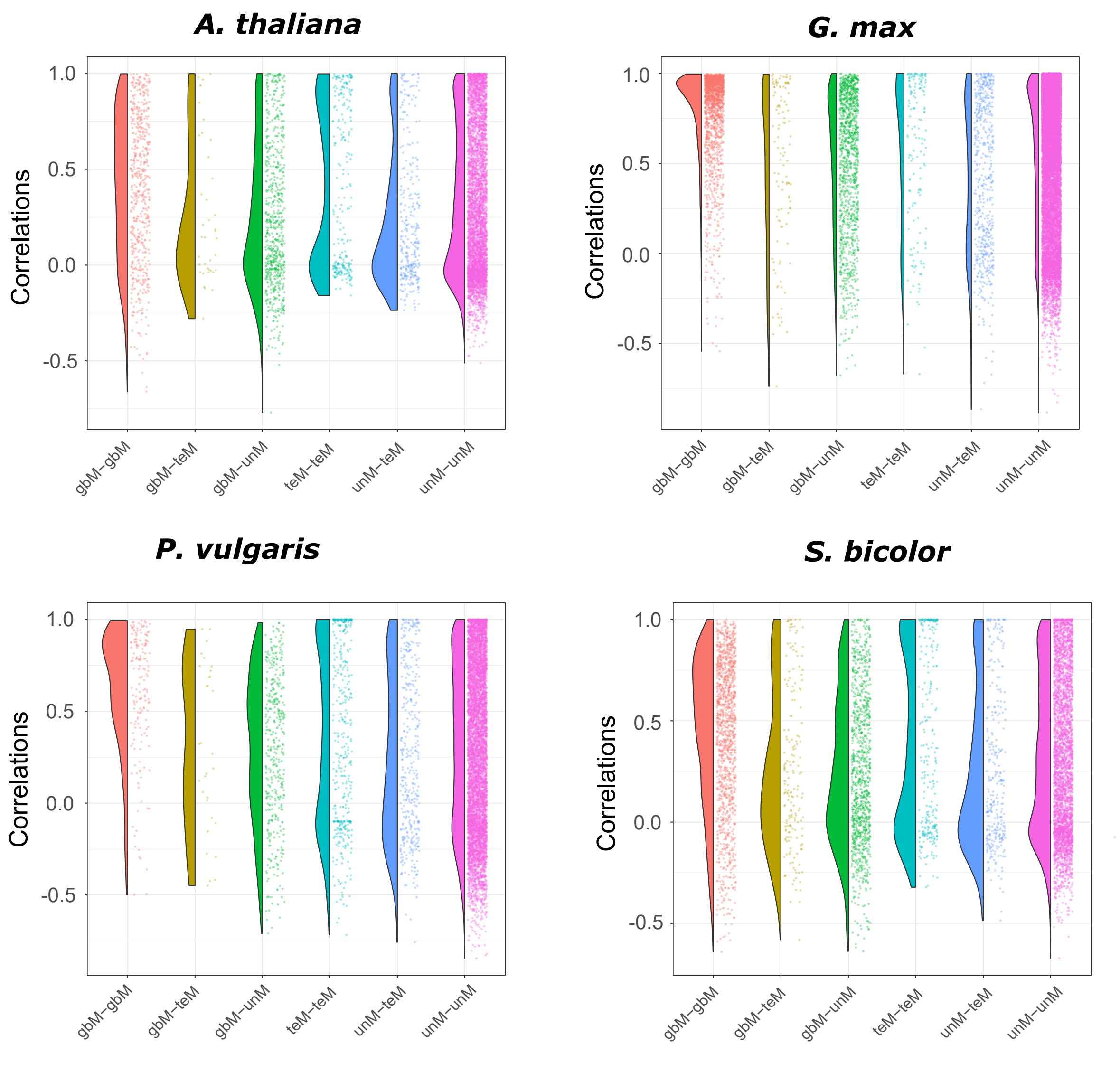

Supplemental Figure S18: Absolute differences in Tau specificity between duplicate pairs in *G. max, P. vulgaris,* and *S. bicolor*. Data is broken down based on the genic methylation of the duplicate pairs (gbM-gbM, gbM-teM, teM-teM, unM-unM, gbM-unM, and unM-teM). Each dot represents adsolute difference in Tau between duplicate genes and the center line in the half boxplot represents the median, while the box limits represent 25% and 75% percentile of the interquartile range, whiskers represent 1.5 times above or below the interquartile range.

G. max

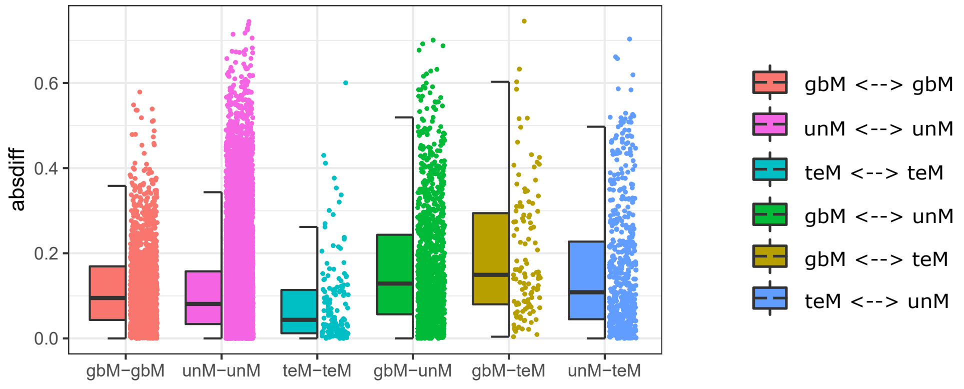

P. vulgaris

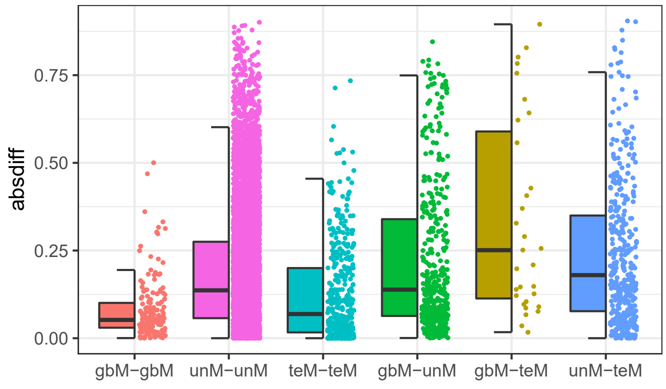

S. bicolor

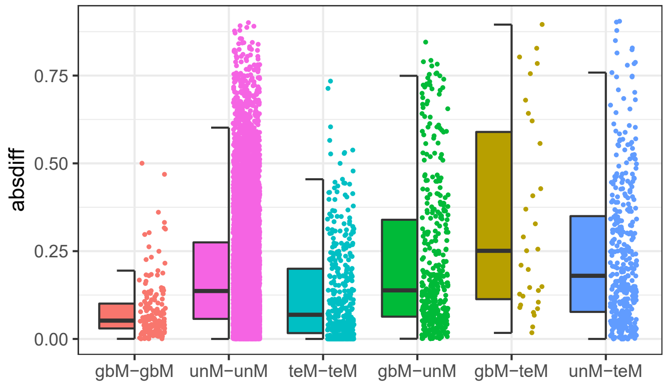

Supplemental Figure S19: Distribution of Tau specificities for gbM, unM, and teM genes separated based on the methylation of their duplicate pair for *G. max, P. vulgaris,* and *S. bicolor.* For example, for gbM genes, the tau specificity was plotted for all gbM genes and the gbM paralog in gbM-gbM, gbM-teM, and gbM-unM pairs. For unM genes, the tau of only the unM paralog is shown and similarly for teM genes, only the tau of the teM paralog is shown. Center line in the boxplot represents the median Tau, while the box limits represent 25% and 75% percentile of the interquartile range, whiskers represent 1.5 times above or below the interquartile range and dots represents outliers

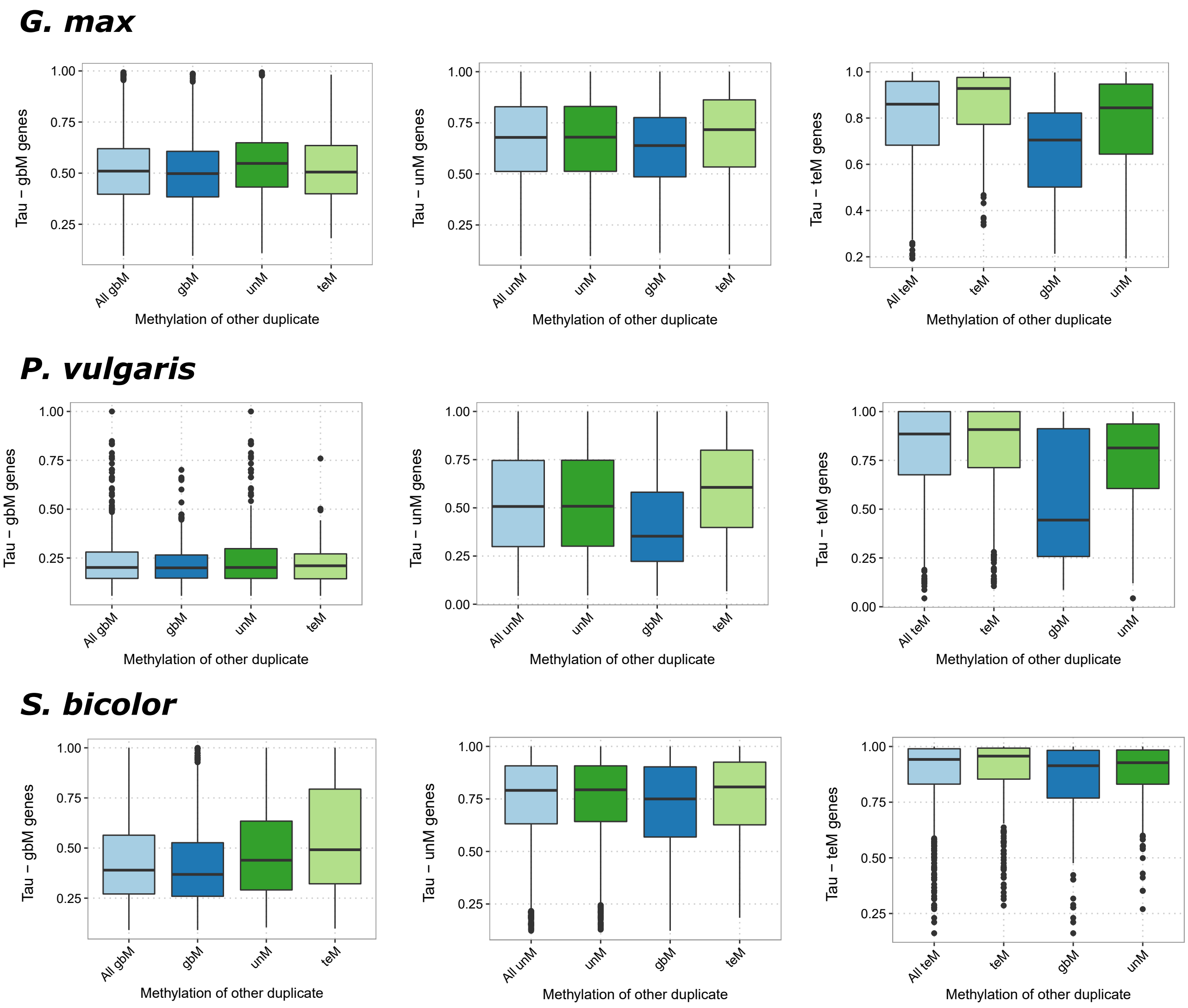

Supplemental Figure S20: Proportion of different orthogroup classification and gbM/unM/teM epiallele frequency within *A. thaliana* population.

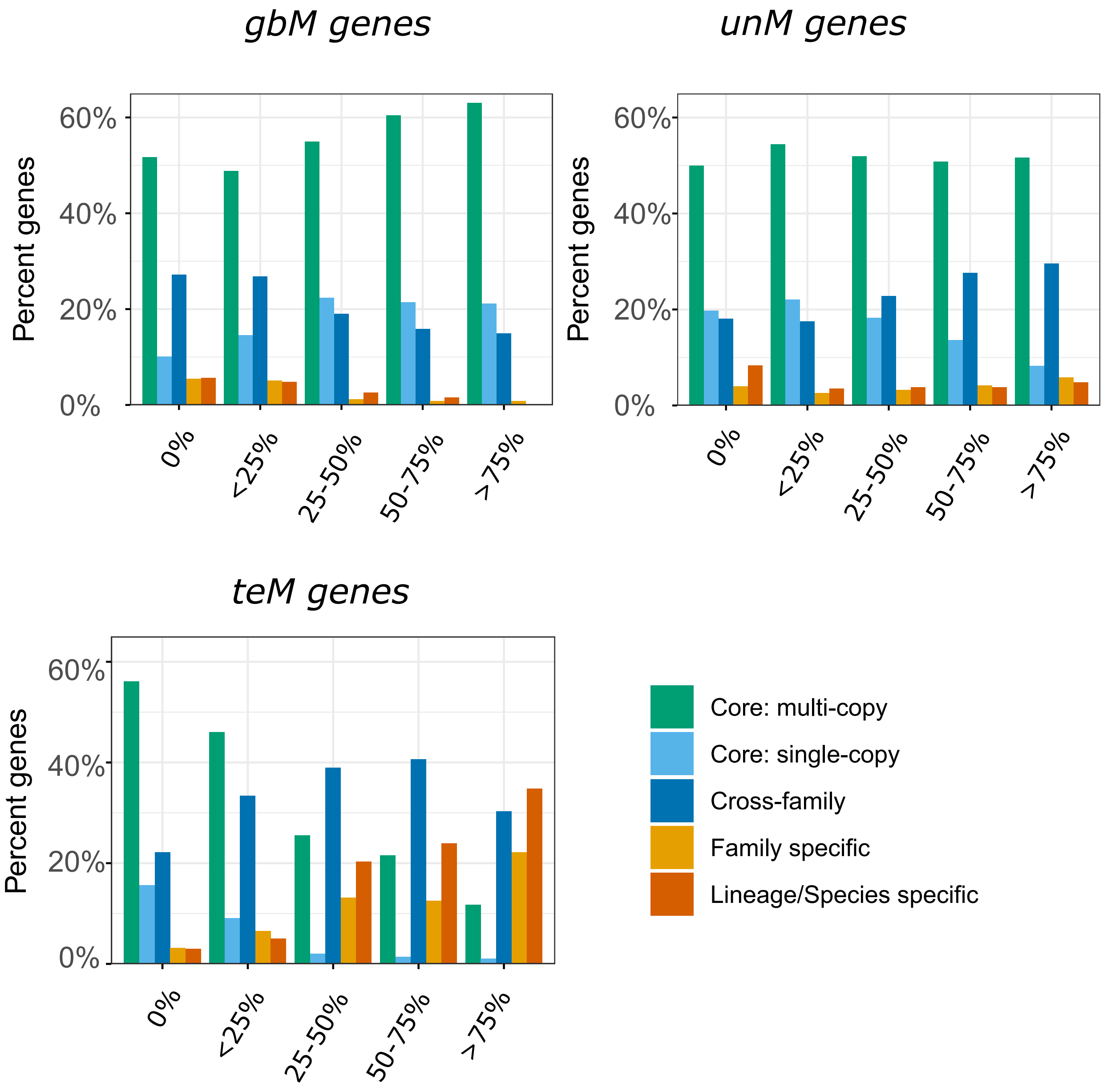

Supplement: kiad220_Supplementary_Data [file kiad220_supplementary_data.zip › Supplemnetary_Figures_120.pdf]
